# Enantio‐ and Regioselective Cascade Hydroboration of Methylenecyclopropanes for Facile Access to Chiral 1,3‐ and 1,4‐Bis(boronates)

**DOI:** 10.1002/advs.202400096

**Published:** 2024-03-13

**Authors:** Jian Zhou, Ling Meng, Ziyi Yang, Jun (Joelle) Wang

**Affiliations:** ^1^ Department of Chemistry Hong Kong Baptist University Kowloon Hong Kong China

**Keywords:** bis(boronates), cascade reaction, C─C bond cleavage, hydroboration

## Abstract

Chiral 1, n‐bis(boronate) plays a crucial role in organic synthesis and medicinal chemistry. However, their catalytic and asymmetric synthesis has long posed a challenge in terms of operability and accessibility from readily available substrates. The recent discovery of the C═C bond formation through β‐C elimination of methylenecyclopropanes (MCP) has provided an exciting opportunity to enhance molecular complexity. In this study, the catalyzed asymmetric cascade hydroboration of MCP is developed. By employing different ligands, various homoallylic boronate intermediate are obtained through the hydroboration ring opening process. Subsequently, the cascade hydroboration with HBpin or B_2_pin_2_ resulted in the synthesis of enantioenriched chiral 1,3‐ and 1,4‐bis(boronates) in high yields, accompanied by excellent chemo‐ and enantioselectivities. The selective transformation of these two distinct C─B bonds also demonstrated their application potential in organic synthesis.

## Graphical abstract

A Cu‐catalyzed asymmetric cascade hydroboration of methylenecyclopropanes allowed for the synthesis of a wide range of chiral 1,3‐ and 1,4‐bis(boronates) via highly selective C─C bond cleavage (up to 98% yield and up to >99% ee). Besides, the application potential of these two distinct C─B bonds in organic synthesis is showcased through their selective transformation.

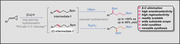

## 1 Introduction

**Scheme 1:**
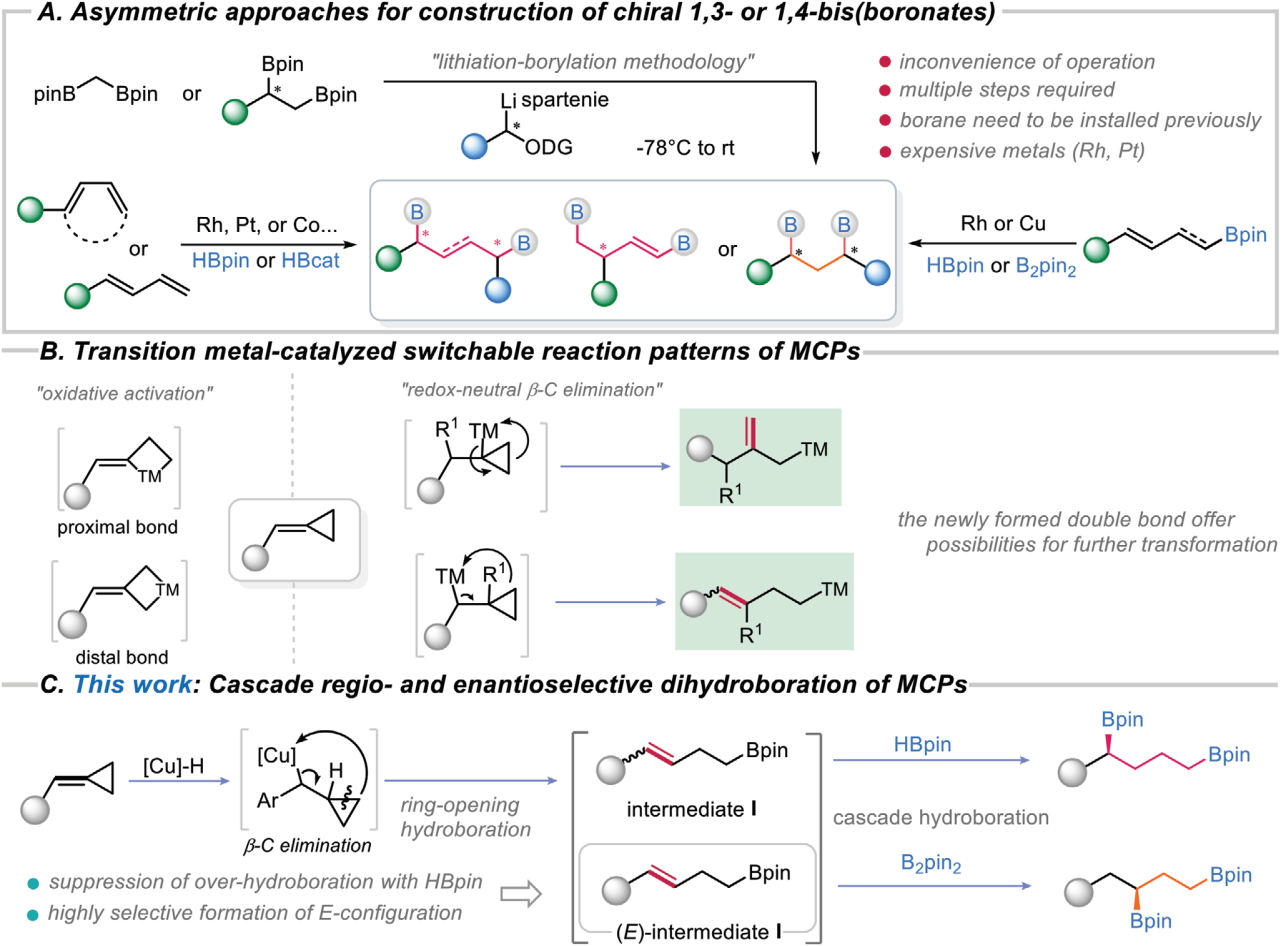
A) Previous methods for synthesis of chiral 1,3‐ and 1,4‐bis(boronates); B) Switchable reaction patterns of MCPs catalyzed by transition metals and C) Research Design: Cu‐catalyzed ring‐opening cascade hydroboration of MCPs.

Chiral 1,n‐ bis(boronate) compounds, known for their potential in the asymmetric construction of complex structures through selective and multiple conversions of two C‐B bonds,^[^ ^1^ ^]^ are recognized as highly versatile and valuable synthetic modules in chemical biology, material science and organic synthesis.^[^ ^2^ ^]^ To date, transition metal‐catalyzed hydroboration, diboration, hydrogenation and cross‐coupling reactions have been developed for the synthesis of chiral geminal^[^ ^3^ ^]^ and vicinal diboronates.^[^ ^4^ ^]^ However, the efficient and straightforward approaches for preparation of chiral 1,3‐ and 1,4‐bis(boronate) compounds are very limited, and only a few advancements have been made (**Scheme** **1**A). Based on lithiation‐borylation methodology,^[^ ^5^ ^]^ Aggarwal and co‐workers realized sparteine‐ligated lithiated carbamate‐promoted asymmetric homologation of optically pure 1,2‐bis(boronic esters) and double homologation of diborylmethane to generate chiral 1,3‐bis(boronic esters).^[^ ^6^ ^]^ Besides, dienes were applied into the synthesis of such structures.^[^ ^7^ ^]^ Morken and co‐workers developed an enantioconvergent 1,4‐diboration of diene catalyzed by Pt/TADDOL or OxaPhos.^[^ ^7a‐c^ ^]^ Other alternative methods include asymmetric hydroboration/boration of designed unsaturated substrates that require prior attachment of borane groups.^[^ ^8^ ^]^ Indeed, there is still a strong desire for the development of a practical method that is capable of constructing 1,3‐ or 1,4‐diboronates from readily accessible starting materials, providing a more efficient and accessible route to these valuable chiral compounds in an enantio‐ and regioselective manner.

Methylenecyclopropanes (MCPs), a type of highly versatile reagent commonly used in organic synthesis, can undergo various switchable reaction patterns through C─C bond cleavage facilitated by transition metals (Scheme 1B).^[^ ^9^ ^]^ For example, the insertion of transition metals into proximal C2─C3 single bond or the distal C3─C4 single bond results in the formation of characteristic metallacyclobutane species.^[^ ^10^ ^]^ While the addition of transition metal complexes to the exo C═C bond happened, the redox‐neutral β‐C elimination occurs, leading to the generation of various ring‐opening structures and the formation of new C═C bond.^[^ ^11^ ^]^ The newly formed double bond in MCPs has the potential for further transformation, especially in asymmetric version. Nevertheless, the merger of ring‐opening process and the cascade enantioselective hydrofunctionalization of MCPs, which could lead to the synthesis of complex chiral molecules with high enantioselectivity is very rare. Recently, we reported the Cu/DTBM‐SegPhos‐catalyzed cascade hydroamination reaction of MCPs and chiral 1,4‐diamines were obtained with high efficiency.^[^ ^12^ ^]^

On the other hand, copper‐catalyzed asymmetric hydroboration of unsaturated bonds has emerged as a powerful tool for the construction of chiral organoborons, due to the mild reaction conditions, high efficiency, and the advantage of using low‐cost of Cu catalysts.^[^ ^13^ ^]^ In 2019, Engle and co‐workers reported a copper‐catalyzed hydroboration of benzylidenecyclopropanes with B_2_pin_2_, affording access to cyclopropylboronic esters and alkenylboronates in one step granted by BINAP and DPPE.^[^ ^14^ ^]^ Very recently, the cobalt‐catalyzed dihydroboration of arylidenecyclopropanes was disclosed by Ge group.^[^ ^15^ ^]^ The 1,4‐diboronate was obtained via a homoallylic boronate intermediate and the 1,3‐diboronate was derived from a diene intermediate, both in racemic form.

Based on our ongoing interests in developing asymmetric hydrofunctionalization reactions of unsaturated bonds, particularly those involving P‐containing species addition reactions and cascade reactions catalyzed by transition metals,^[^ ^12^, ^16^ ^]^ we have made further progress in understanding the characteristic reaction patterns of MCPs. Specifically, we have discovered that the characteristic reaction pattern of MCPs involves a process of β‐C elimination followed by a sequential cascade hydroamination of the newly formed double bond, facilitated by Cu‐H species. This discovery inspired us to explore the possibility of employing chiral copper catalyst with HBpin or B_2_Pin_2_ reagents, which may enable a new type of cascade reaction of MCPs (Scheme 1C). In our investigation, we envisioned that the first hydroboration of MCPs with HBpin involves the regioselective migratory insertion of L*Cu‐H and subsequent β‐C elimination, leading to the formation of a homoallylic boronate intermediate I. This intermediate then undergoes a cascade hydroboration of the double bond, resulting in the formation of a chiral 1,4‐bis(boronate) compound. The switch to the construction of chiral 1,3‐bis(boronate) compounds by asymmetric hydroboration of intermediate I with B_2_pin_2_ is appealing, but there are several challenges that need to be addressed to achieve the high chemo‐, enantio‐ and regioselectivity. For instance, the suppression of over‐hydroboration of intermediate I in the ring‐opening process to ensure the regioselective of cascade hydroboration is necessary. Besides, controlling the highly selective formation of (*E*)‐intermediate **I** via β‐C elimination is crucial for the cascade hydroboration with L*Cu‐Bpin species. In response to this scenario, herein, we disclose a copper‐catalyzed asymmetric ring‐opening cascade hydroboration of MCPs driven by C─C bond cleavage, presenting a highly enantio‐ and regioselective access to structurally diverse and synthetically versatile chiral 1,3‐ and 1,4‐bis(boronates).

## 2 Results and Discussion

We commenced our study by using (cyclopropylidenemeth‐yl)‐benzene **1a** and HBpin **2** as benchmark substrates with Cu(OAc)_2_ in toluene at room temperature to investigate the efficiency of various commercially available chiral ligands (**Table** **1**). To easily determine the enantioselectivities, 1,4‐bis(boronate) product was oxidized to the corresponding 1,4‐diol in the presence of NaBO_3_·H_2_O. Excellent enantioselectivities were observed when ligand **L5**, **L6**, and **L7** were used, but the yields of target compound were only moderate (entry 5–7). (*S,S*)‐Ph‐BPE (**L8**) was found to be the best ligand, delivering cascade hydroboration product in 91% yield and 98% ee at room temperature (entry 8). In comparison to Cu(OAc)_2_, the use of CuCl maintained the ee value, but caused a slight drop in yield (entry 9). Besides, no increase in yield was observed by raising the reaction temperature (entry 10).

**Table 1:** Optimization of the reaction conditions.

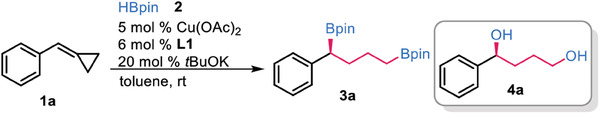				
entry^a)^	ligand	time [h]	yield [%]^b)^	ee^c)^
1	**L1**	24	trace	NA
2	**L2**	24	trace	NA
3	**L3**	24	18	NA
4	**L4**	24	21	NA
5	**L5**	36	72	96
6	**L6**	36	65	99
7	**L7**	36	79	96
8	**L8**	36	91	98
9^d)^	**L8**	36	78	96
10^e)^	**L8**	36	85	98
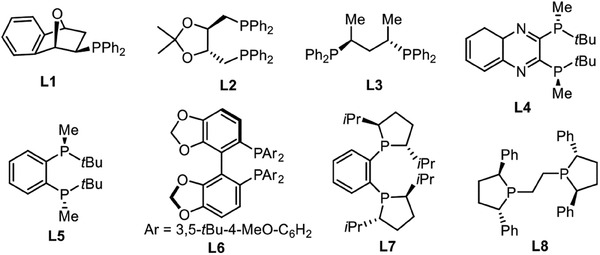				

^a)^ Unless otherwise noted, all reactions were carried out with (cyclopropylidenemeth‐yl)‐benzene **1a** (0.1 mmol), **2** (0.22 mmol), *t*BuOk (20 mol %) and metal/ligand (1:1.2, 5 mol %) in toluene 0.5 mL;

^b)^ Isolated yields for **3a**;

^c)^ ee was determined by its derivative **4a**;

^d)^ 5 mol % CuCl was used;

^e)^ at 40 °C.

Having optimized reaction conditions in hand, a scope study of various MCP derivatives was conducted and outlined in **Table** **2**. Generally, a range of substituents, including both electron‐donating and electron‐withdrawing groups at the para‐, meta‐, and ortho‐ positions on the aryl group of MCPs, were well‐tolerated, affording corresponding products in good to excellent yields (46−97% yields) with 90−98% ee. Notably, products **4d**, **4e**, **4i,** and **4k** were generated with >99% ees. Additionally, substrates containing other aromatic rings, such as aryl, naphthalene, and benzofuran, were also compatible in this transformation and delivered products **4n**, **4o**, **4p**, and **4q** in good yields and high level of enantioselectivies. MCPs with two or three substitutes on aryl ring could be dihydroborated to products **4r** and **4s** smoothly. The attempt to employ aliphatic substituted MCP was unsuccessful (see Supporting Information). Furthermore, the absolute configuration of **4a** was unambiguously determined to be *S* by comparing with the literature.^[^ ^17^ ^]^

**Table 2:** Substrate scope of MCPs in synthesis of chiral 1,4‐bis(boronates).^a)^

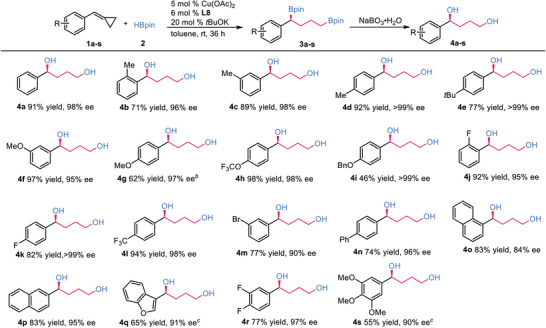

^a)^ Unless otherwise noted, the reaction conditions were conducted with **1** (0.1 mmol, 1.0 equiv), **2** (0.22 mmol, 2.2 equiv), Cu(OAc)_2_ (5 mol %), **L8** (6 mol %), *t*BuOK (20 mol %) in toluene 0.5 mL, rt, 36 h. Isolated yields of compounds **4**;

^b)^ reacted at 40 °C for 48 h;

^c)^ 10 mol % catalyst was used and reacted for 60 h.

**Scheme 2:**
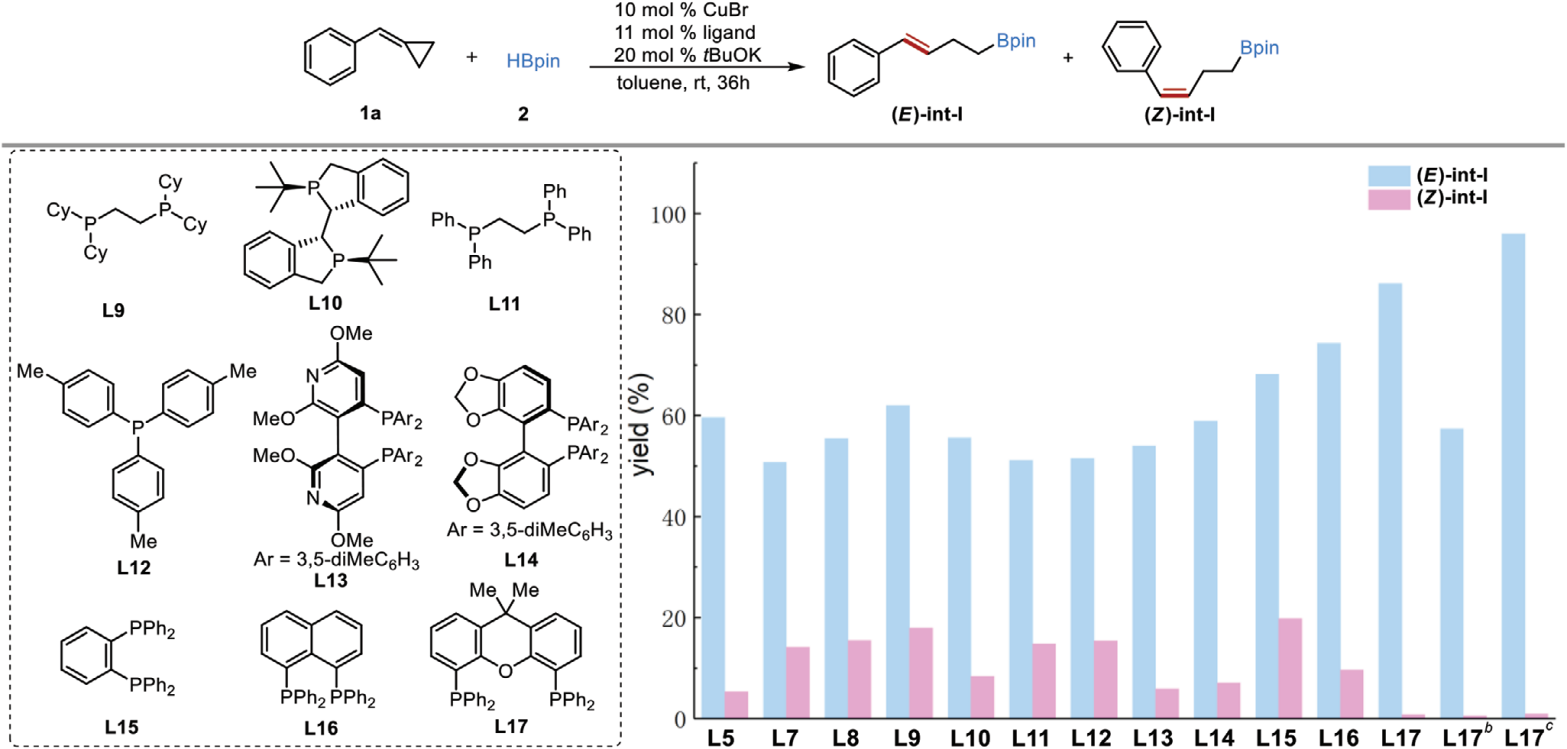
Optimization of formation of **(*E)*‐int‐I** ^[a]^. a) Unless otherwise noted, the reaction conditions were conducted with **1a** (0.1 mmol, 1.0 equiv), **2** (0.1 mmol, 1.0 equiv), CuBr/Ligand (1/1.1, 10 mol %), and *t*BuOK (20 mol %) in toluene 0.5 mL, rt, 36 h. All yields and E/Z ratio were evaluated by ^1^H NMR. b) THF as solvent. c) 2.5 mol % catalyst was used and ratio of **1a**/**2** was 1.2/1.

Next, our attention turned toward the formation of chiral 1,3‐bis(boronates), where a cascade asymmetric hydroboration of intermediate **I** with B_2_pin_2_ was designed. To prevent over‐hydroboration of intermediate **I** during the ring‐opening process of MCP, modification of reaction conditions was conducted (see Table S1, Supporting Information). Under the CuBr/**L8**‐catalyzed condition, the **int‐I** was obtained in 71% yield but with E/Z ratio of 78/22 and no observation of over‐hydroborated product. Unlike the protocol that reacted with HBpin, the formation of E/Z isomers with different configurations would complicate the construction of carbon chirality of C─B bond from addition of Cu‐Bpin species to the double bond in a one‐pot sequential protocol. It becomes crucial to control and obtain the highly selective **(*E*)‐int‐I** to ensure the next controlling of the C‐B bond formation. After extensive optimization, it was found that Cu precursors, solvent and base were not the crucial factors in achieving E/Z selectivity (see Tables S1 and S2, Supporting Information). Then, various ligands were screened using CuBr as the precursor, *t*BuOK as the base, and toluene as the solvent. In **Scheme** **2**, P‐stereogenic ligands **L5** and **L10** could slightly increase the E/Z ratio of **int‐I**, and similar results were also observed for axial ligands **L13** and **L14**, in which isomers of **int‐I** were obtained with E/Z ratio of 90/10 and 89/11 respectively. Monodentate ligand **L12** was also capable of facilitating the ring‐opening process, but with poor selectivity. To our delight, the use of **L17** enabled the generation of nearly pure **(*E*)‐int‐I** with an E/Z ratio of 99/1 in 87% yield. This finding provided a highly selective approach for the desired **(*E*)‐int‐I**. The use of THF as solvent dropped the yield to 58% while maintaining the high E/Z selectivity. The DFT calculation was conducted, and it was shown that the larger bite angle of **L17**, which allows a larger catalyst pocket, was considered as a major factor for the high selectivity (see Supporting Information). Subsequently, efforts toward the optimization of asymmetric hydroboration of **(*E*)‐int‐I** with B_2_pin_2_ were made. Of note, the failure of **L17** as ligand in the cascade transformation guarantees the construction of chirality (see Table S3, Supporting Information). By successfully combining the optimized reaction conditions for each step, a sequential one‐pot process was achieved, in which intermediate **(*E*)‐int‐I** was prepared without isolated and directly subjected to the CuBr/**L10** catalytic condition, leading to a generation of chiral 1,3‐bis(boronate) **6a** in 87% yield with 90% ee (see Table S3, Supporting Information).

With optimized reaction conditions in hands, we next focused on examining the generality of this sequential cascade protocol. As described in **Table** **3**, various MCPs bearing with different substitutes (Me, *t*Bu, Ph, OMe, OCF_3_, F, and Br) at orth‐, meta‐ or para‐ position of the phenyl group were efficiently transformed into desired products with moderate to good yields (41‐82%) and high levels of enantio‐controlling (74–91% ees). Additionally, fused ring substrates (**7j** and **7k**) and benzofuran ring (**7l**) were tolerated. Multi‐substituted MCPs with 3,4‐diF and 3,4,5‐triOMe also afforded products **7m** and **7n** in good yields and enantioselectivities. Finally, the absolute configuration of **7a** was determined to be *S* by comparison with the literature.^[^ ^18^ ^]^

**Table 3:** Substrate scope of MCPs in synthesis of chiral 1,3‐bis(boronates).^a)^

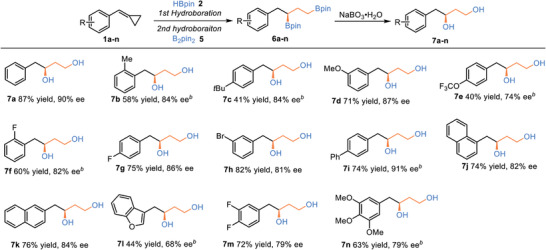

^a)^ Unless otherwise noted, the reaction conditions were conducted with **1** (0.12 mmol, 1.2 equiv), **2** (0.1 mmol, 1.0 equiv), CuBr (2.5 mol %), **L17** (12 mol %), *t*BuOK (20 mol %) in toluene 0.4 mL, rt, 36 h. Then, the prepared mixture of CuBr/**L10** (1/1.1, 10 mol %), *t*BuOK (20 mol %) in THF 0.8 mL was added, followed by the addition of **5** (0.12 mmol, 1.2 equiv). The mixture was cooled to −25 °C, 3.0 equiv MeOH was added and reacted at −25 °C for 48 h. Isolated yields of compounds **7**;

^b)^ reacted at −25 °C for 52 h.

**Scheme 3:**
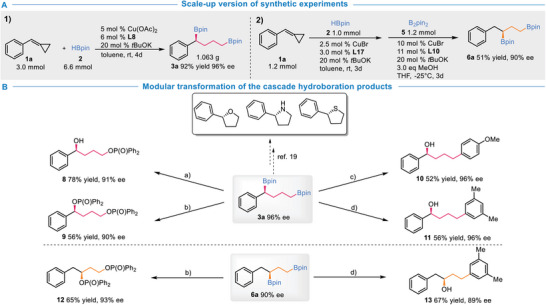
Scale‐up reactions and product diversification. a) NaBO_3_·H_2_O (4.0 equiv), THF/H_2_O, rt, 4 h, then Ph_2_P(O)Cl (3.0 equiv), TEA (3.0 equiv), CH_3_Cl, rt. 12 h. b) NaBO_3_·H_2_O (4.0 equiv), THF/H_2_O, rt, 4 h, then Ph_2_P(O)Cl (4.5 equiv), TEA (4.5 equiv), CH_2_Cl_2_, rt. 12 h. c) 1‐bromo‐4‐methoxybenzene (2.0 equiv), Pd_2_(dba)_3_ (2.5 mol %), RuPhos (5 mol %), *t*BuONa (4.0 eq), toluene/H_2_O, 100 °C 12 h, then, NaBO_3_·H_2_O (2.0 equiv), THF/H_2_O, rt, 4 h. d) 1‐bromo‐3,5‐dimethylbenzene (2.0 equiv), Pd_2_(dba)_3_ (2.5 mol %), RuPhos (5 mol %), *t*BuONa (4.0 eq), toluene/H_2_O, 100 °C, 12 h, then, NaBO_3_·H_2_O (2.0 equiv), THF/H_2_O, rt, 4 h.

To further demonstrate the practical utility of this approach, cascade Cu‐catalyzed dihydroboration reactions on a larger scale were performed. The product **3a** was obtained in 92% yield and 96% ee in the gram‐scale synthetic experiments. MCP **1a** at 1.2 mmol scale in the one‐pot protocol was transformed to **6a** with no loss of enantioselectivity (**Scheme** **3**a). Furthermore, we also conducted the synthetic transformations of these structurally diverse and synthetically versatile chiral 1,3‐ and 1,4‐bis(boronates). Optically pure five‐membered rings, such as tetrahydrofuran, pyrrolidine, and tetrahydrothiophene, could be easily synthesized through several convenient transformations of **3a**.^[^ ^19^ ^]^ Particularly, the presence of two C‐B bonds provides extensive opportunities for modular manipulations. For instance, chiral phosphate **8** with a hydroxyl group could be furnished, and chiral 1,4‐ and 1,3‐diphosphate **9** and **12** were modularly synthesized with good enantiocontrolling from **3a** and **6a** respectively. Moreover, the involvement of **3a** and **6a** in Pd‐catalyzed Suzuki−Miyaura cross‐couplings and sequential oxidation reaction led to the synthesis of chiral butanols **10**, **11** with different substitutes, and chiral propanol **13** (Scheme 3b).

**Scheme 4:**
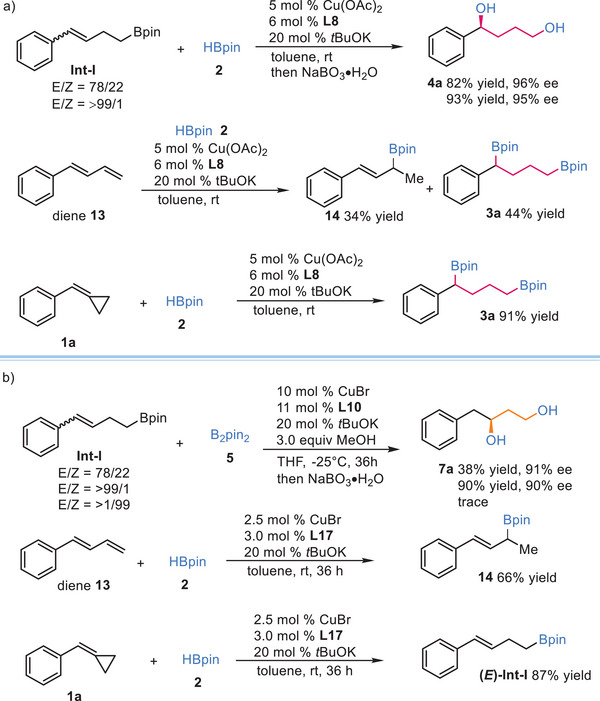
Control experiments of cascade hydroboration.

Several experiments were conducted to gain insights of mechanism of this cascade reaction. the **int‐I** obtained with an E/Z ratio of 78/22, and pure (*E*)‐**int‐I**, were subjected to the standard conditions, product **4a** was observed in 82% yield, and 93% yield with similar enantioselectivities respectively. Besides, diene **13** was prepared and subjected to Cu‐catalyzed cascade hydroboration, under catalyzed by Cu(OAc)_2_/**L8**, a mixture of allylboronate **14** (34% yield) and 1,4‐boronate **3a** (44% yield) were produced with moderate regioselectivity (**Scheme** **4**a). Furthermore, as shown in Scheme 4b, the **int‐I** with E/Z ratio of 78/22 was subjected to the standard conditions and product **7a** was observed in 38% yield, and 91% yield, which demonstrate the **(*Z*)‐int‐I** delayed the cascade transformation. Also, only trace amount of **7a** was observed when **(*Z*)‐int‐I** was employed in standard condition. Additionally, when CuBr/**L17** was used as the catalyst, only the formation of allylboronate **14** was observed. This observation suggests a distinct reaction pathway which exclude the possibility of diene as intermediate in comparison to the Co‐catalyzed hydroboration of MCPs.^[^ ^15^ ^]^

**Figure 1:**
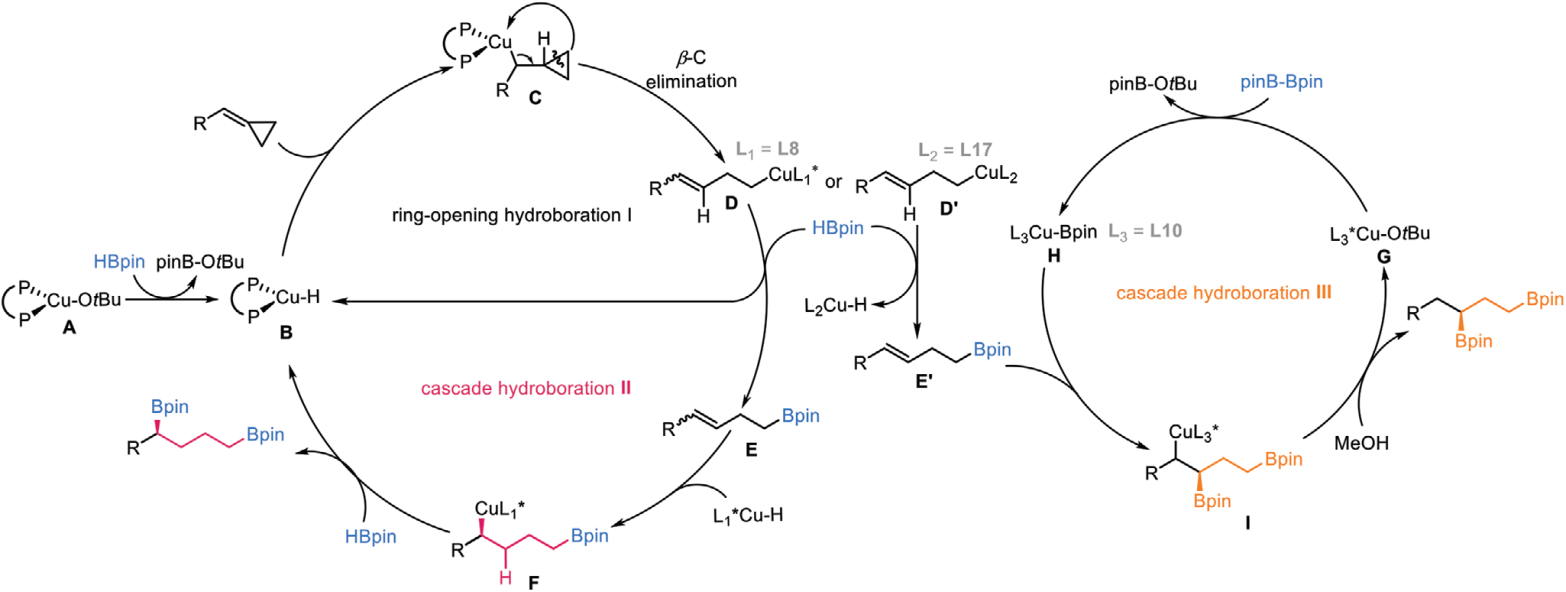
Proposed catalytic cycle.

Based on the previous reports on Cu‐catalyzed hydroboration and considering the control experiments above,^[^ ^3^, ^12^, ^20^ ^]^ we proposed a plausible mechanism for the hydroboration where HBpin or B_2_pin_2_ were involved (**Figure** **1**). The reaction is initiated by Cu‐H species **B,** which is generated through the reaction of Cu‐alkoxide **A** with pinacolborane. Cu‐H species **B** then adds to the *exo*‐double bond of MCP, leading to the formation of complex **C**. The subsequent β‐C elimination of complex **C** releases complex **D**, which undergoes reductive elimination to form intermediate **E** and liberate Cu‐H species **B**. Notably, the use of **L17** enables the selective formation of trans‐intermediate **D'**, and then affords trans‐intermediate **E’**. Intermediate **E** then proceeds through the cascade hydroboration **II** procedure with HBpin, providing chiral 1,4‐bis(boronate). Alternatively, as shown in cascade hyroboration **III**, chiral nucleophile Cu‐Bpin **H**, formed through transmetalation between Cu‐alkoxide **G** and B_2_pin_2,_ coordinates and adds to double bond of intermediate **E’**. Then, the protonation of alkyl‐Cu intermediate **I** via MeOH produces chiral 1,3‐bis(boronate) and regenerates Cu‐alkoxide **G**.

## 3 Conclusion

In this work, we have disclosed an efficient, straightforward, and versatile cascade hydroboration protocol of MCPs, which involves a ring‐opening hydroboration procedure through β‐C elimination, followed by a cascade hydroboration using different type of boron reagents. This methodology allows the facile access to a range of chiral 1,4‐ and 1,3‐bis(boronates) in a highly chemo‐ and enantioselective manner. The modularity of diversifying two C‐B bonds of products to obtain various optically pure 1,3‐ and 1,4‐diphosphnates, butanol, and propanol with different substitutes highlights the practicality of this novel and easily operated protocol. We anticipate that this protocol will inspire further exploration of its potential applications in asymmetric catalysis and organic synthesis.

## Conflict of Interest

The authors declare no conflict of interest.

## Supporting information

Supporting Information

## Data Availability

The data that support the findings of this study are available in the supplementary material of this article.
