# Supplementary material for: Enantio‐ and Regioselective Cascade Hydroboration of Methylenecyclopropanes for Facile Access to Chiral 1,3‐ and 1,4‐Bis(boronates)
Source: Adv Sci (Weinh). 2024 Mar 13;11(21):2400096. doi: 10.1002/advs.202400096 (PMC11151016; doi:10.1002/advs.202400096)
Supplement: Supplementary file 1 — Supporting Information [file ADVS-11-2400096-s001.pdf]

ADVANCED SCIENCE
Open Access

Supporting Information

for *Adv. Sci.*, DOI 10.1002/advs.202400096

Enantio- and Regioselective Cascade Hydroboration of Methylenecyclopropanes for Facile Access to Chiral 1,3- and 1,4-Bis(boronates)

*Jian Zhou, Ling Meng, Ziyi Yang and Jun (Joelle) Wang**

Supporting Information

Enantio- and Regioselective Cascade Hydroboration of Methylenecyclopropanes for Facile Access to Chiral 1,3- and 1,4-bis(boronates)

Jian Zhou,[a] Ling Meng,[a] Ziyi Yang[a] and Jun (Joelle) Wang[a]*

[a]Department of Chemistry, Hong Kong Baptist University, Kowloon, Hong Kong, China;

Contents

1.General information 2

2. Optimization for synthesis of (*E*)-int-I 2

3. Optimization for synthesis of chiral 1,3-bis(boronate) 6a[a] 4

4. Typical procedure for copper-catalyzed cascade asymmetric hydroboration 5

5. Gram-scale reaction and synthetic transformation of the products 8

6. Synthesis of (*Z*)-int-I 13

7. Control experiments 14

8. Calculation details 16

9. Analytic data for the products 21

10. NMR Spectrum 42

11. HPLC Spectrum 89

12. Reference 128

1.General information

NMR Spectra were recorded on a Bruker DPX-500 (400) spectrometer at 600 MHz or 400 MHz for ^{1}H NMR, 376 MHz for ^{19}F NMR and 101 MHz or 125 MHz for ^{13}C NMR in $CDCl_3$ with tetramethylsilane (TMS) or the residual deuterated solvent peaks as internal standard. Chemical shifts (δ) are reported in ppm, and coupling constants (J) are in Hertz (Hz). Flash column chromatograph was carried out using 200-300 mesh silica gel at medium pressure. High resolution mass spectra (HRMS) were recorded on a LC-TOF spectrometer. ESI-HRMS data were acquired using a Thermo LTQ Orbitrap XL Instrument equipped with an ESI source. Optical rotation was obtained on a Rudolph Research Analytical (Atopol I). HPLC analysis was performed on Agilent 1260 series. Unless otherwise noted, all reagents were purchased from commercial suppliers and used without purification. All air- and moisture-sensitive manipulations were carried out with standard Schlenk techniques under nitrogen or in a glove box under argon. Anhydrous THF (Tetrahydrofuran), toluene were distilled from sodium benzophenone prior to use. Methylenecyclopropanes are known compounds and were synthesized according to the literature procedure.[1]

2. Optimization for synthesis of (*E*)-int-I

Table S1[a]

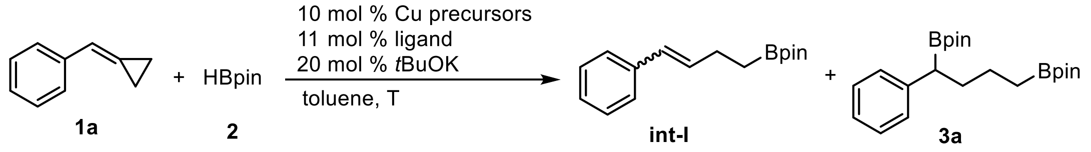

Entry	Ratio (1a/2)	Cu precursor	Ligand	T/°C	Time(h)	Yield for int-I(%)[b]	E/Z ratio[c]
1	1/1.5	$Cu(OAc)_2$	**L8**	rt	36	55 (20 for **3a**)	-
2	1/1.2	CuBr	**L8**	rt	36	71	78/22
3	1/1	CuBr	**L8**	rt	36	68	77/23
4	1/1	CuBr	**L8**	4	48	74	78/22
5	1/1	CuBr	**L8**	50	36	68	79/21
6	1/1	$Cu(MeCN)BF_4$	**L8**	rt	36	70	77/23
7	1/1	CuI	**L8**	rt	36	72	76/24
8	1/1	$Cu(MeCN)PF_6$	**L8**	rt	36	65	78/22
9	1/1	CuCl	**L8**	rt	36	68	78/22
10	1/1	CuOAc	**L8**	rt	36	62	78/22

11	1/1	CuBr	**L5**	rt	36	65	92/08
12	1/1	CuOAc	**L5**	rt	36	71	92/08

[a]Reaction conditions: MCP **1a** (0.1 mmol), Hbpin **2** (0.1 mmol), Cu precursor (10 mol %), Ligand (11 mol %) and *t*BuOK (20 mol %) in 0.5 mL toluene, stirred at indicated temperature. [b,c]All yields and E/Z ratio were evaluated by ^{1}H NMR.

^{1}H NMR (400 MHz, Chloroform-*d*) δ 7.35 – 7.25 (m, 4H), 7.22 – 7.11 (m, 1H), 6.42 – 6.33 (m, 1H), 6.34 – 6.23 (m, 1H), 2.38 – 2.27 (m, 2H), 1.24 (s, 12H), 0.98 (t, *J* = 7.8 Hz, 2H).

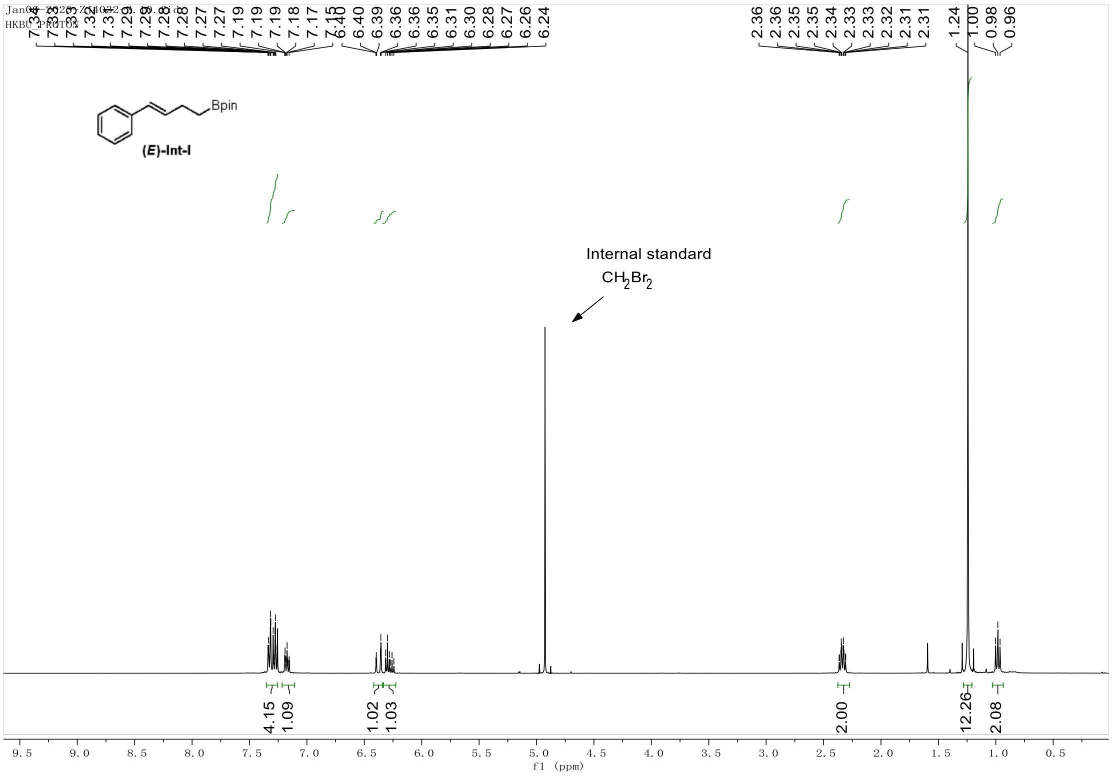

Table S2[a]

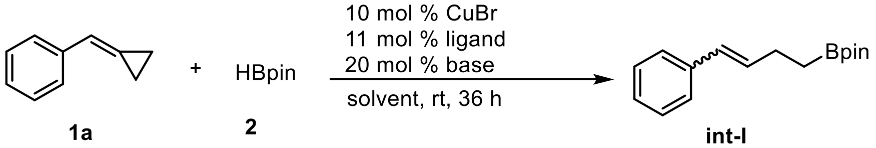

Entry	Ligand	Sovlent	Base	Yield for int-I (%)[b]	E/Z ratio[c]
1	**L8**	toluene	*t*BuOLi	65	78/22
2	**L8**	toluene	*t*BuONa	59	78/22
3	**L8**	toluene	MeOK	74	81/19
4[d]	**L8**	toluene	*t*BuOK	62	77/23
5	**L8**	mesitylene	*t*BuOK	55	79/21
6	**L8**	1,4-dioxane	*t*BuOK	48	79/21
7	**L8**	DMF	*t*BuOK	33	71/29
8	**L8**	DCE	*t*BuOK	63	81/19
9	**L8**	EA	*t*BuOK	11	76/24
10	**L8**	MeCN	*t*BuOK	58	79/21

11	**L8**	CPME	*t*BuOK	43	78/22
12	**L5**	THF	*t*BuOK	55	92/08
13	**L5**	Hex	*t*BuOK	66	92/08

[a]Reaction conditions: MCP **1a** (0.1 mmol), Hbpin **2** (0.1 mmol), CuBr (10 mol %), Ligand (11 mol %) and base (20 mol %) in 0.5 mL solvent, stirred at rt for indicated time. [b,c]All yields and E/Z ratio were evaluated by ^{1}H NMR. [d]10 mol % PPh_3 was added.

3. Optimization for synthesis of chiral 1,3-bis(boronate) 6a[a]

Table S3[a]

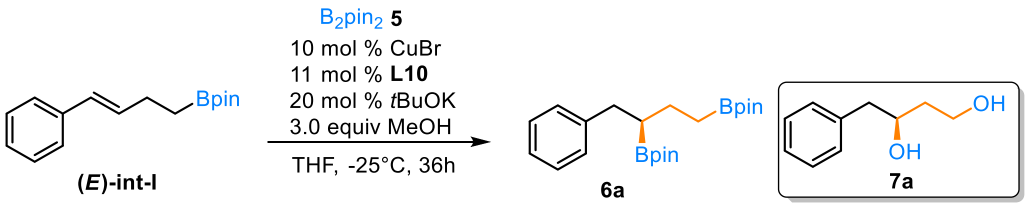

Entry	variation from optimized condition	yield[b]	ee[c]
1	none	91	90
2	**L2** instead of **L10**	trace	NA
3[d]	**L5** instead of **L10**	65	65
4[e]	**L5** instead of **L10**	78	73
5	**L6** instead of **L10**	trace	NA
6[d]	**L8** instead of **L10**	60	66
7[e]	**L8** instead of **L10**	70	66
8	**L13** instead of **L10**	trace	NA
9	**L17** instead of **L10**	trace	NA
10	**L18** instead of **L10**	trace	NA
11	**L19** instead of **L10**	trace	NA
12	**L20** instead of **L10**	trace	NA
13[d]	**L21** instead of **L10**	35	31
14[f]	**L21** instead of **L10**	46	37
15	-35℃	54	86
16	rt	80	70
17	toluene as solvent	70	70
18[g]	**(*E*)-3a** was prepared without purificaiton	87	90

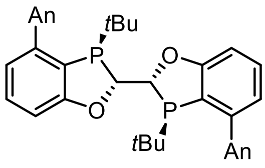

L18

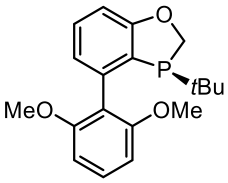

L19

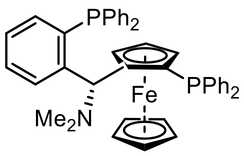

L20

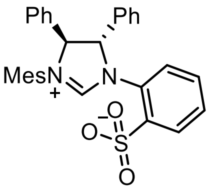

L21

[a]Unless otherwisenoted, all reactions were carried out with (cyclopropylidenemeth-yl)-benzene **1a** (0.1mmol), B_2pin_2 **5** (0.12 mmol), *t*BuOk (20 mol %) and metal/ligand (1:1.2, 5 mol %) in

THF 0.5 mL. [b]Isolated yields. [c] ee value of **7a** was determined by HPLC on a chiral stationary phase. [d]at rt. [e]at -35°C. [f]at 2°C. [g] reacted for 48h,

An endeavor towards the optimization of asymmetric hydroboration of isolated ***(E)*-int-I** with B_2pin_2 **5** was made. After extensive investigation on various reaction parameters, chiral 1,3-bis(boronate) **6a** that was oxidized to 1,3-diol **7a** could be obtained in 91% yield with 90% ee at -25°C in the presence of 10 mol % CuBr, **L10** (11 mol %), *t*BuOK (20 mol %), and 3.0 equiv MeOH in THF (entry 1). Screening of different commercial ligands, such as **L2**, **L6**, **L13**, **L18**, **L19** and **L20** could not afford product. The employment of **L5** at room temperature led to formation of **6a** in 65% yield with 65% ee, lowering reacted temperature to -35 °C improved both yield and ee, albeit, to 78% yield and 73% ee slightly (entry 3 and entry 4). Similar phenomenon was observed when NHC-**L21** was used. **L8** that demonstrated highly catalytic efficiency in construction of chiral 1,4-bis(boronate) only gave product in moderate yield and enantiocontrol (60% yield and 66% ee), reacting at -35°C could slightly increase yield, nevertheless, have no impact on enantioselectivity of product (entry 6 and entry 7). Of note, the failure of **L17** as ligand in the cascade transformation guaranteed the construction of chirality (entry 9). Besides, no matter being stirred at more lowering temperature (-35°C) or at room temperature, varying degree of reduction in both yields and ee values were observed (entry 15 and 16). Using toluene as solvent instead of THF lowered reaction efficiency with no diminishment of enantioselectivity (entry 17). Lastly, the sequential one-pot cascade protocol was developed for synthesis of chiral 1,3-bis(boronate) using HBpin **2** and B_2pin_2 **5**. Gratifyingly, the success of combining optimized reaction conditions for each step realize this sequential process, in which intermediate ***(E)*-int-I** was prepared without isolated and directly subjected to the CuBr/**L10** catalytic condition, allowed the access to a generation of product **6a** in 87% yield with 90% ee (entry 18).

4. Typical procedure for copper-catalyzed cascade asymmetric hydroboration

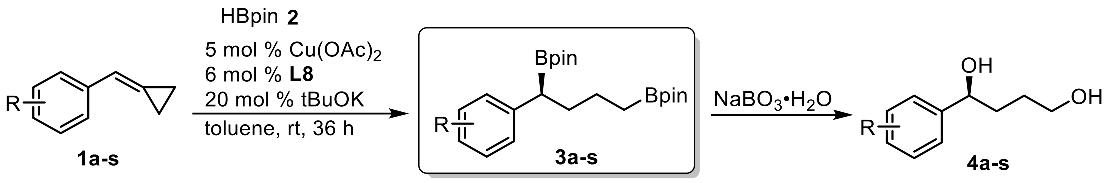

General procedure A: An oven-dried Schlenk tube with a stirred bar was charged with 5 mol % $Cu(OAc)_2$ (1.0 mg, 0.0050 mmol), 6 mol % **L8** (3.0 mg, 0.0060 mmol) and 0.5 mL toluene in argon atmosphere. The resulting solution was stirred at room temperature for 20 min, then *t*BuOK (2.2 mg, 0.0200 mmol) was allowed to add and stirred for additional 10 min, then 0.22 mmol HBpin **2** and 0.1 mmol methylenecyclopropanes **1** were added, the mixture was stirred at room temperature under argon atmosphere for 36 h. Then the resulting solution was added 5 mL of DCM and filtered through a pad of silica gel, washed by PE/EA = 10/1 (10 mL x 3). The combined filtrates were concentrated and dissloved in THF/H_2O (2 mL, 1/1 v/v), then $NaBO_3$ ·H_2O (0.4000 mmol, 40.0 mg) and was added. The resulting mixture was allowed to stir at room temperature for 4 h. The reaction mixture was diluted with EtOAc (5 mL) and H_2O (2 mL). The aqueous layer was extracted with EtOAc (5×2 mL). The organic layer was dried over anhydrous Na_2SO_4, filtered, and concentrated under vacuum. The residue was purified by column chromatography on silica gel to get the corresponding product **4.** The ee values was determined by HPLC.

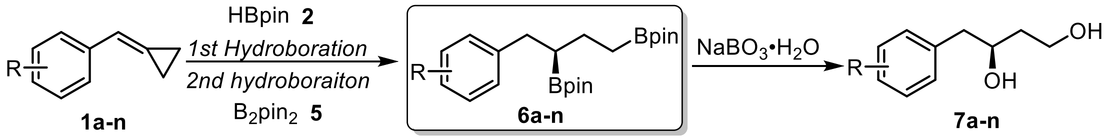

General procedure B: An oven-dried Schlenk tube with a stirred bar was charged with 2.5 mol % CuBr (0.36 mg, 0.0025 mmol), 3 mol % **L17** (1.7 mg, 0.0030 mmol) and 0.4 mL toluene in argon atmosphere. The resulting solution was stirred at room temperature for 20 min, then *t*BuOK (2.2 mg, 0.0200 mmol) was allowed to add and stirred for additional 10 min, then 0.1 mmol HBpin **2** and 0.12 mmol methylenecyclopropanes **1** were added, the mixture **A** was stirred at room temperature under argon atmosphere for 36 h. On the other hand, 10 mol % CuBr (1.4 mg, 0.0010 mmol), 11 mol % **L10** (4.2 mg, 0.0011 mmol) and 0.8 mL THF in argon atmosphere. The resulting solution was

stirred at room temperature for 20 min, then *t*BuOK (2.2 mg, 0.0200 mmol) was allowed to add and stirred for additional 10 min, resulting in the formation of mixture **B**. Afterward, mixture **B** was transferred to mixture **A**, followed by the addition of 0.12 mmol of B_2pin_2 **5** to the resulting solution. The final solution was then cooled to -25 ℃, followed by introduction of 3.0 equivalents of MeOH, and stirred for 48 h. Then the resulting solution was added 5 mL of DCM and filtered through a pad of silica gel, washed by PE/EA = 10/1 (10 mL x 3). The combined filtrates were concentrated and dissloved in THF/H_2O (2 mL, 1/1 v/v), then $NaBO_3 \cdot H_2O$ (0.4000 mmol, 40.0 mg) and was added. The resulting mixture was allowed to stir at room temperature for 4 h. The reaction mixture was diluted with EtOAc (5 mL) and H_2O (2 mL). The aqueous layer was extracted with EtOAc (5×2 mL). The organic layer was dried over anhydrous Na_2SO_4, filtered, and concentrated under vacuum. The residue was purified by column chromatography on silica gel to get the corresponding product **7.** The ee values was determined by HPLC.

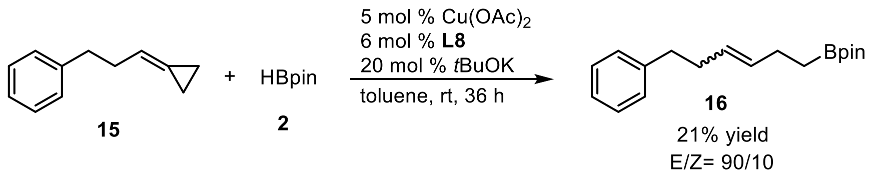

An oven-dried Schlenk tube with a stirred bar was charged with 5 mol % $Cu(OAc)_2$ (1.0 mg, 0.0050 mmol), 6 mol % **L8** (3.0 mg, 0.0060 mmol) and 0.5 mL toluene in argon atmosphere. The resulting solution was stirred at room temperature for 20 min, then *t*BuOK (2.2 mg, 0.0200 mmol) was allowed to add and stirred for additional 10 min, then 0.1 mmol HBpin **2** and 0.1 mmol (3-cyclopropylidenepropyl)benzene **15** were added, the mixture was stirred at room temperature under argon atmosphere for 36 h. The reaction mixture was purified by column chromatography on silica gel to get the corresponding product **16**. ^{1}H NMR (400 MHz, Chloroform-*d*) δ 7.31-.26 (m, 2H), 7.20-7.16 (m, 3H), 5.44-5.30 (m, 2H), 2.65 (dd, *J* = 9.0, 6.7 Hz, 2H), 2.40-2.32 (m, 2H), 2.11 (q, *J* = 7.4 Hz, 2H), 1.24 (s, 13H), 0.80 (t, *J* = 7.9 Hz, 2H).

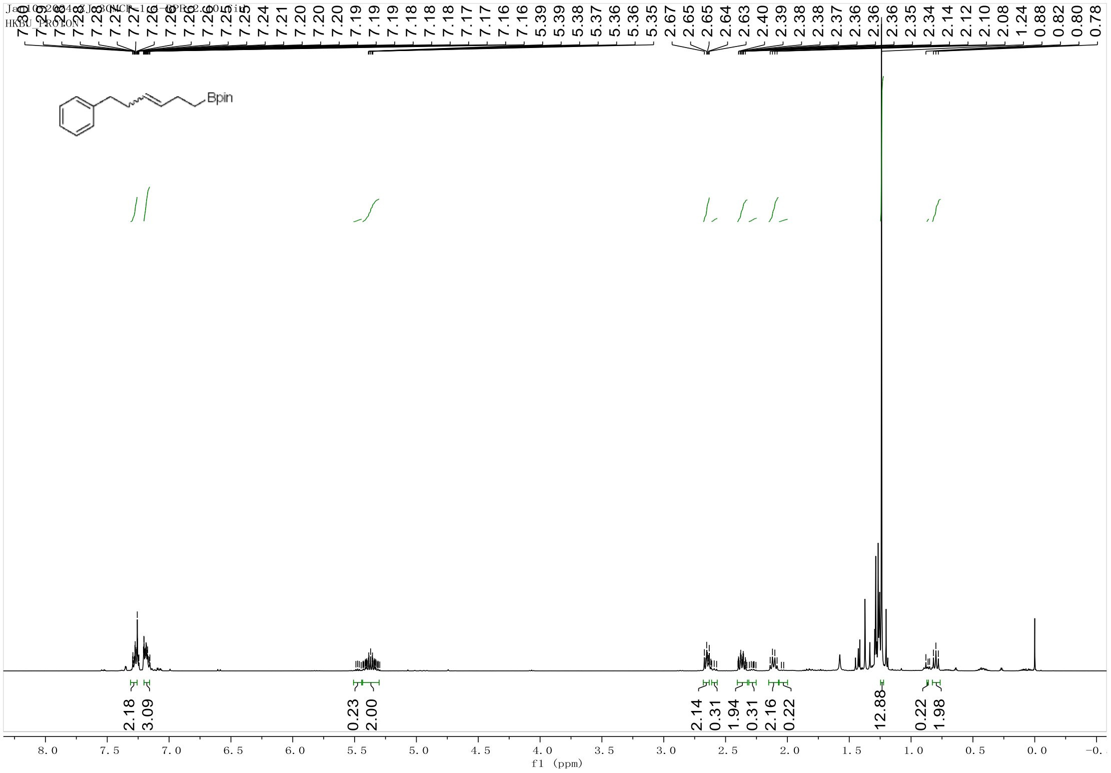

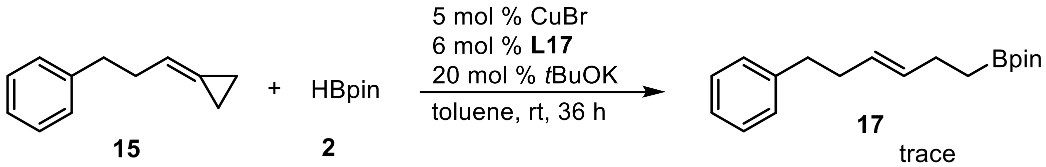

An oven-dried Schlenk tube with a stirred bar was charged with 5 mol % CuBr (0.72mg, 0.005 mmol), 6 mol % **L17** (3.4 mg, 0.006 mmol) and 0.5 mL toluene in argon atmosphere. The resulting solution was stirred at room temperature for 20 min, then *t*BuOK (2.2 mg, 0.0200 mmol) was allowed to add and stirred for additional 10 min, then 0.1 mmol HBpin **2** and 0.1 mmol (3-cyclopropylidenepropyl)benzene **15** were added, the mixture was stirred at room temperature under argon atmosphere for 36 h. Only trace amount of 17 was observed and (3-cyclopropylidenepropyl)benzene **15** was decomposed.

5. Gram-scale reaction and synthetic transformation of the products

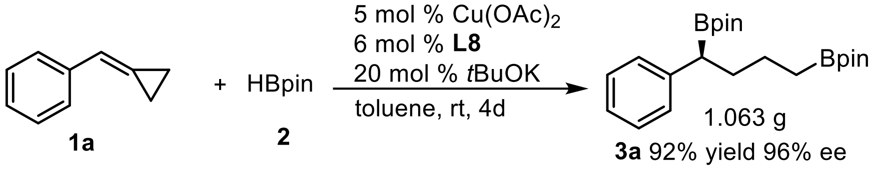

An oven-dried Schlenk tube with a stirred bar was charged with 5 mol % $Cu(OAc)_2$ (27.2 mg, 0.1500 mmol), 6 mol % **L8** (91.1 mg, 0.1800 mmol) and 15.0 mL toluene in argon atmosphere. The resulting solution was stirred at room temperature for 30 min, then *t*BuOK (67.2 mg, 0.6000 mmol) was allowed to add and stirred for additional 10 min, then 6.6 mmol HBpin **2** and 3.0 mmol methylenecyclopropanes **1a** were added, the mixture was stirred at room temperature under argon atmosphere for 4 d. Then the reaction mixture was purified by column chromatography on silica gel to give product **3a** (1.06 g, 96% ee) .

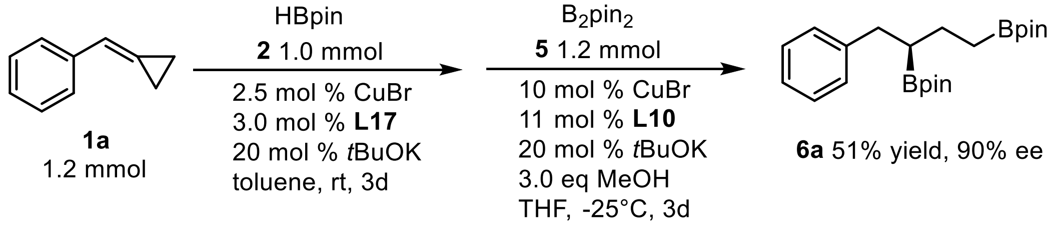

An oven-dried Schlenk tube with a stirred bar was charged with 2.5 mol % CuBr (3.6 mg, 0.0250 mmol), 3 mol % **L17** (18 mg, 0.0300 mmol) and 4 mL toluene in argon atmosphere. The resulting solution was stirred at room temperature for 30 min, then *t*BuOK (22.0 mg, 0.2000 mmol) was allowed to add and stirred for additional 10 min, then 1 mmol HBpin **2** and 1.2 mmol methylenecyclopropanes **1** were added, the mixture **A** was stirred at room temperature under argon atmosphere for 3 d. On the other hand, 10 mol % CuBr (14.3 mg, 0.0100 mmol), 11 mol % **L10** (42 mg, 0.1100 mmol) and 8 mL THF in argon atmosphere. The resulting solution was stirred at room temperature for 30 min, then *t*BuOK (22.0 mg, 0.2000 mmol) was allowed to add and stirred for additional 10 min, resulting in the formation of mixture **B**. Afterward, mixture **B** was transferred to mixture **A**, followed by the addition of 1.2 mmol of B_2pin_2 **5** to the resulting solution. The final solution was then cooled to -25 ℃, followed by introduction of 3.0 equivalents of MeOH, and stirred for 48 h. Then the reaction mixture

was purified by column chromatography on silica gel to deliver product **6a** (196.0 mg, 90% ee).

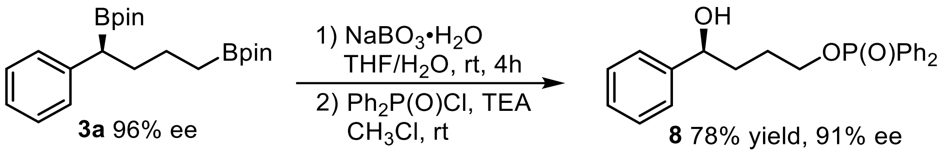

To a mixture of **3a** (38.6 mg, 0.1000 mmol, 96% ee) in THF/H_2O (2 mL, 1/1 v/v) was added $NaBO_3 \cdot H_2O$ (0.4000 mmol, 40.0 mg). The resulting mixture was allowed to stir at room temperature for 4 h. The reaction mixture was diluted with EtOAc (5 mL) and H_2O (2 mL). The aqueous layer was extracted with EtOAc (5×2 mL). The organic layer was dried over anhydrous Na_2SO_4, filtered, and concentrated under vacuum. After purification by using silica gel flash column chromatography, the intermediate was dissolved in 2 mL CH_3Cl, followed by sequential addition of 3.0 equivalents of $Ph_2P(O)Cl$ (70.9 mg, 0.3000 mmol) and 3.0 equivalents of TEA (30.3 mg, 0.3000 mmol). The resulting mixture was allowed to stir at room temperature under argon atmosphere for 12 h. After evaporation of reacting mixture, the crude product was purified silica gel flash column chromatography to give product **8** (28.5 mg, 78% yield, 91% ee).

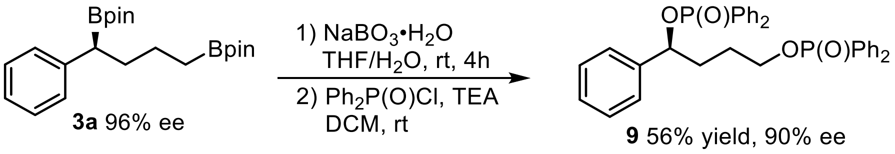

To a mixture of **3a** (77.2 mg, 0.2000 mmol, 96% ee) in THF/H_2O (4 mL, 1/1 v/v) was added $NaBO_3 \cdot H_2O$ (0.8000 mmol, 80.0 mg). The resulting mixture was allowed to stir at room temperature for 4 h. The reaction mixture was diluted with EtOAc (5 mL) and H_2O (2 mL). The aqueous layer was extracted with EtOAc (5×2 mL). The organic layer was dried over anhydrous Na_2SO_4, filtered, and concentrated under vacuum. After purification by using silica gel flash column chromatography, the intermediate was dissolved in 4 mL CH_3Cl, followed by sequential addition of 4.5 equivalents of $Ph_2P(O)Cl$ (212.7 mg, 0.9000 mmol) and 3.0 equivalents of TEA (90.6

mg, 0.9000 mmol). The resulting mixture was allowed to stir at room temperature under argon atmosphere for 12 h. After evaporation of reacting mixture, the crude product was purified silica gel flash column chromatography to give product **9** (63.0 mg, 78% yield, 90% ee).

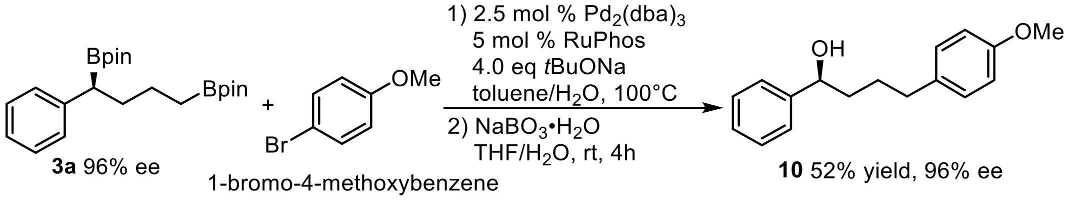

An oven-dried Schlenk tube with a stirred bar was charged with 2.5 mol % $Pd_2(dba)_3$ (4.6 mg, 0.0050 mmol), 5 mol % **L8** (4.7 mg, 0.0050 mmol) and 1 mL toluene in argon atmosphere. The resulting solution was stirred at room temperature for 10 min, then *t*BuONa (76.9 mg, 0.8000 mmol), **3a** (76.8 mg, 0.2000 mmol, 96% ee), 1-bromo-4-methoxybenzene (74.8 mg, 0.4000 mmol), and DI water (60 μL) were allowed to add and stirred at 100°C for 12h. Then, the crude product was purified using silica gel flash column chromatography (120:1 to 100:1 hexane/EtOAc). Subsequently, the crude product was dissloved in THF/H_2O (2 mL, 1/1), then $NaBO_3 \cdot H_2O$ (0.8000 mmol, 80.0 mg) and was added. The resulting mixture was allowed to stir at room temperature for 4 h. Then, the reaction mixture was diluted with EtOAc (5 mL) and H_2O (2 mL). The aqueous layer was extracted with EtOAc (5×2 mL). The organic layer was dried over anhydrous Na_2SO_4, filtered, and concentrated under vacuum. The residue was purified by column chromatography on silica gel to give **10** (26.6 mg, 52% yield, 96% ee).

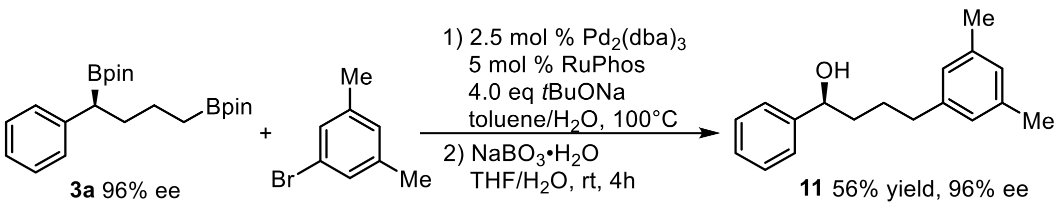

An oven-dried Schlenk tube with a stirred bar was charged with 2.5 mol % $Pd_2(dba)_3$ (4.6 mg, 0.0050 mmol), 5 mol % **L8** (4.7 mg, 0.0050 mmol) and 1 mL toluene in argon atmosphere. The resulting solution was stirred at room temperature for 10 min, then *t*BuONa (76.9 mg, 0.8000 mmol), **3a** (76.8 mg, 0.2000 mmol, 96% ee), 1-bromo-

3,5-dimethylbenzene (73.2 mg, 0.4000 mmol), and DI water (60 μL) were allowed to add and stirred at 100°C for 12h. Then, the crude product was purified using silica gel flash column chromatography (120:1 to 100:1 hexane/EtOAc). Subsequently, the crude product was dissloved in THF/H_2O (2 mL, 1/1), then $NaBO_3 \cdot H_2O$ (0.8000 mmol, 80.0 mg) and was added. The resulting mixture was allowed to stir at room temperature for 4 h. The reaction mixture was diluted with EtOAc (5 mL) and H_2O (2 mL). The aqueous layer was extracted with EtOAc (5×2 mL). The organic layer was dried over anhydrous Na_2SO_4, filtered, and concentrated under vacuum. The residue was purified by column chromatography on silica gel to give **11** (28.4 mg, 56% yield, 96% ee).

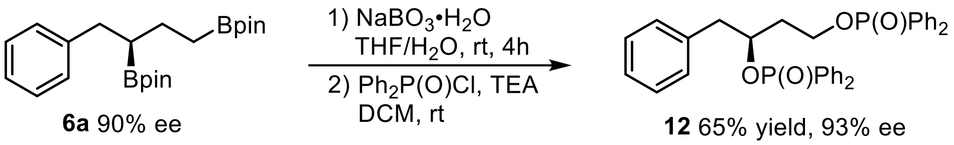

To a mixture of **6a** (38.6 mg, 0.1000 mmol, 90% ee) in THF/H_2O (2 mL, 1/1 v/v) was added $NaBO_3 \cdot H_2O$ (0.4000 mmol, 40.0 mg). The resulting mixture was allowed to stir at room temperature for 4 h. The reaction mixture was diluted with EtOAc (5 mL) and H_2O (2 mL). The aqueous layer was extracted with EtOAc (5×2 mL). The organic layer was dried over anhydrous Na_2SO_4, filtered, and concentrated under vacuum. After purification by using silica gel flash column chromatography, the intermediate was dissolved in 2 mL CH_3Cl, followed by sequential addition of 4.5 equivalents of $Ph_2P(O)Cl$ (106.5 mg, 0.4500 mmol) and 4.5 equivalents of TEA (45.3 mg, 0.9000 mmol). The resulting mixture was allowed to stir at room temperature under argon atmosphere for 12 h. After evaporation of reacting mixture, the crude product was purified silica gel flash column chromatography to give product **9** (36.0 mg, 65% yield, 90% ee).

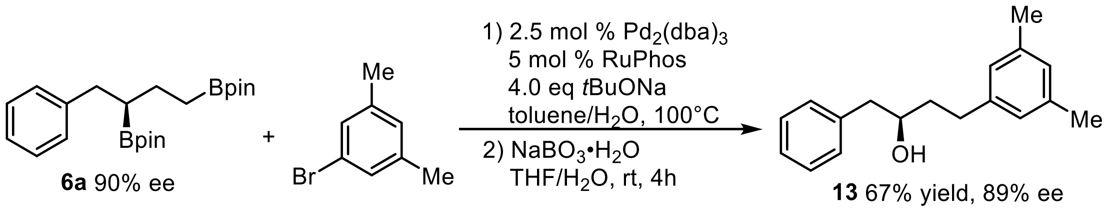

An oven-dried Schlenk tube with a stirred bar was charged with 2.5 mol % $Pd_2(dba)_3$ (2.3 mg, 0.0025 mmol), 5 mol % **L8** (2.3 mg, 0.0025 mmol) and 0.5 mL toluene in argon atmosphere. The resulting solution was stirred at room temperature for 10 min, then *t*BuONa (38.5 mg, 0.4000 mmol), **6a** (38.4 mg, 0.1000 mmol, 90% ee), 1-bromo-3,5-dimethylbenzene (36.6 mg, 0.2000 mmol), and DI water (30 μL) were allowed to add and stirred at 100°C for 12h. Then, the crude product was purified using silica gel flash column chromatography (120:1 to 100:1 hexane/EtOAc). Subsequently, the crude product was dissloved in THF/H_2O (2 mL, 1/1), then $NaBO_3\cdot H_2O$ (0.4000 mmol, 40.0 mg) and was added. The resulting mixture was allowed to stir at room temperature for 4 h. The reaction mixture was diluted with EtOAc (5 mL) and H_2O (2 mL). The aqueous layer was extracted with EtOAc (5×2 mL). The organic layer was dried over anhydrous Na_2SO_4, filtered, and concentrated under vacuum. The residue was purified by column chromatography on silica gel to give **13** (17.0 mg, 67% yield, 89% ee).

6. Synthesis of (*Z*)-int-I [3]

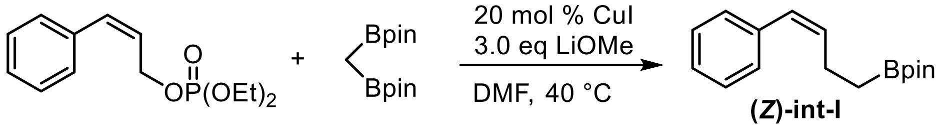

In air, CuI (4 mg, 0.02 mmol), MeOLi (24 mg, 0.6 mmol), diborylmethane (107 mg 0.4 mmol) were added to a Schlenk tube equipped with a stir bar. The vessel was evacuated and filled with argon (this process was repeated three times). DMF(0.5 mL) were added in turn by syringe under argon atmosphere at room temperature, and then stirred at 60 °C for 10 min and cooled to 40 °C. (Z)-diethyl (3-phenylallyl) phosphate (0.2 mmol) was added in turn by syringe under argon atmosphere. The resulting reaction mixture was stirred vigorously at 40 °C for 24 hours. The reaction mixture was then diluted with EtOAc, filtered through silica gel with copious washings (EtOAc), concentrated, and purified by column chromatography to give (Z)-Int-I (43 mg, 86% yield, pale liquid). ^{1}H NMR (400 MHz,

Chloroform-*d*) δ 7.36 – 7.27 (m, 4H), 7.23 – 7.18 (m, 1H), 6.35 (dd, *J* = 11.7, 2.0 Hz, 1H), 5.72 – 5.62 (m, 1H), 2.50 – 2.39 (m, 2H), 1.24 (s, 12H), 0.94 (t, *J* = 7.8 Hz, 2H).

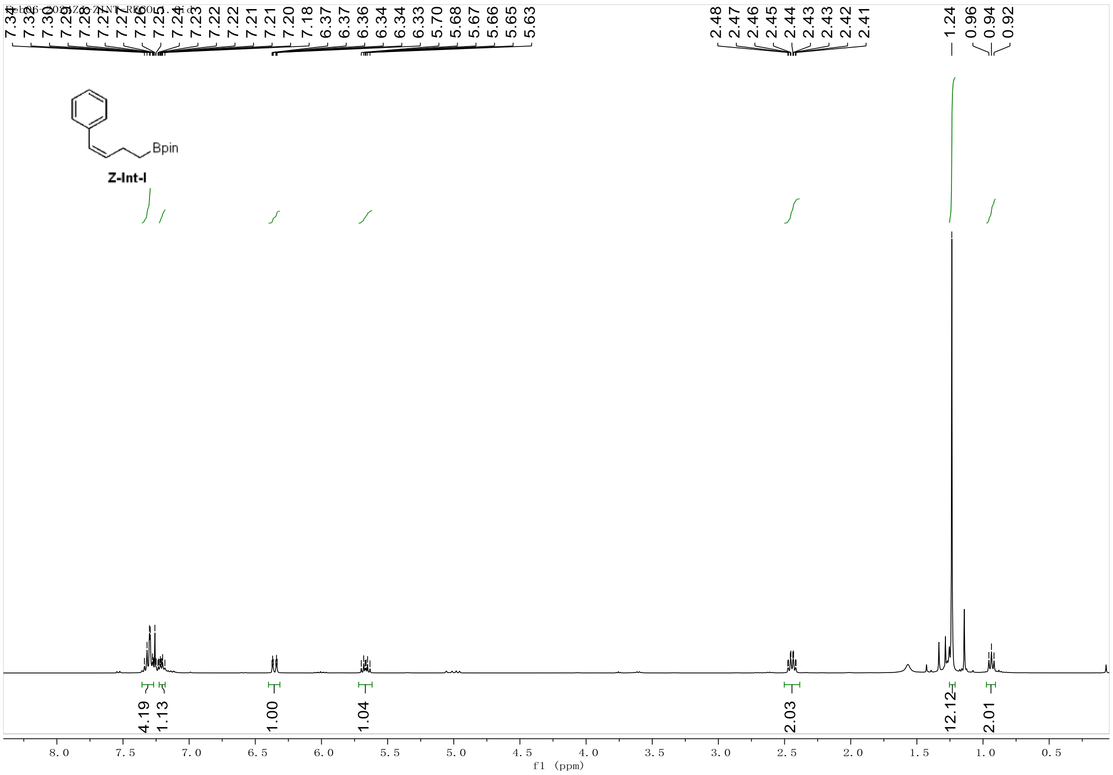

7. Control experiments

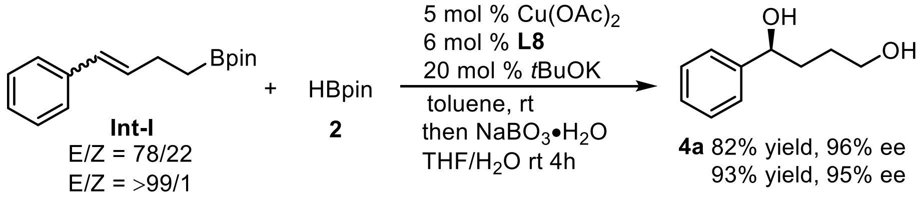

An oven-dried Schlenk tube with a stirred bar was charged with 5 mol % $Cu(OAc)_2$ (1.0 mg, 0.0050 mmol), 6 mol % **L8** (3.0 mg, 0.0060 mmol) and 0.5 mL toluene in argon atmosphere. The resulting solution was stirred at room temperature for 20 min, then *t*BuOK (2.2 mg, 0.0200 mmol) was allowed to add and stirred for additional 10 min, then 0.1 mmol **int-I** with E/Z ratio of 78/22 or >99/1 and 0.22 mmol HBpin **2** was added, the mixture was stirred at room temperature under argon atmosphere for 36 h. Then the resulting solution was added 5 mL of DCM and filtered through a pad of silica gel, washed by PE/EA = 10/1 (10 mL x 3). The combined filtrates were concentrated

and dissloved in THF/H_2O (2 mL, 1/1 v/v), then $NaBO_3 \cdot H_2O$ (0.4000 mmol, 40.0 mg) and was added. The resulting mixture was allowed to stir at room temperature for 4 h. The reaction mixture was diluted with EtOAc (5 mL) and H_2O (2 mL). The aqueous layer was extracted with EtOAc (5×2 mL). The organic layer was dried over anhydrous Na_2SO_4, filtered, and concentrated under vacuum. The residue was purified by column chromatography on silica gel to get the corresponding product **4a** (82% yield, 96% ee or 93% yield, 95% ee).

(*E*)-buta-1,3-dien-1-ylbenzene **13** was synthesized according to previous general procedure.[2]

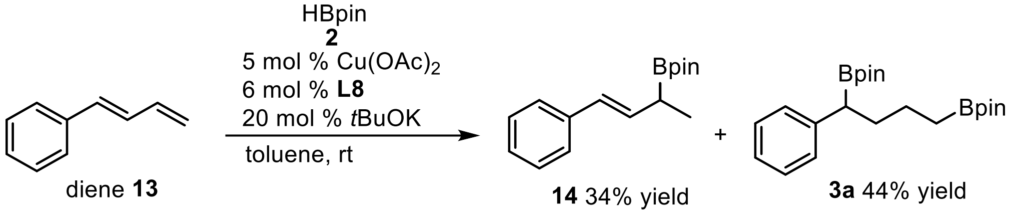

An oven-dried Schlenk tube with a stirred bar was charged with 5 mol % $Cu(OAc)_2$ (1.0 mg, 0.0050 mmol), 6 mol % **L8** (3.0 mg, 0.0060 mmol) and 0.5 mL toluene in argon atmosphere. The resulting solution was stirred at room temperature for 20 min, then *t*BuOK (2.2 mg, 0.0200 mmol) was allowed to add and stirred for additional 10 min, then 0.1 mmol diene **11** and 0.22 mmol HBpin **2** was added, the mixture was stirred at room temperature under argon atmosphere for 36 h. Then the reaction mixture was purified by column chromatography on silica gel to afford **14** (8.8 mg, 34% yield) and **3a** (16.9 mg, 44% yield).

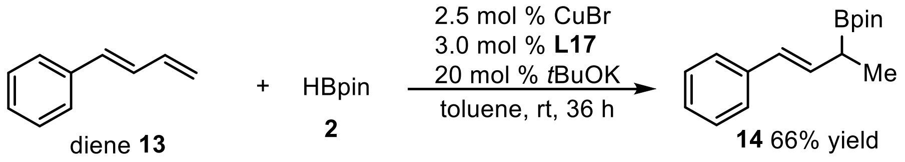

An oven-dried Schlenk tube with a stirred bar was charged with 2.5 mol % CuBr (0.36 mg, 0.0025 mmol), 6 mol % **L17** (1.7 mg, 0.0030 mmol) and 0.4 mL toluene in argon atmosphere. The resulting solution was stirred at room temperature for 20 min, then *t*BuOK (2.2 mg, 0.0200 mmol) was allowed to add and stirred for additional 10

min, then 0.12 mmol diene **13** and 0.1 mmol HBpin **2** was added, the mixture **A** was stirred at room temperature under argon atmosphere for 36 h.Then the reaction mixture was purified by column chromatography on silica gel to afford **14** (16.9 mg, 66% yield). Colorless oil, ^{1}H NMR (400 MHz, Chloroform-*d*) δ 7.36-7.32 (m, 2H), 7.29-7.24 (m, 2H), 7.18-7.13 (m, 1H), 6.37-6.31 (m, 2H), 1.24 (s, 12H), 1.19 (d, *J* = 7.3 Hz, 3H). ^{13}C NMR (101 MHz, Chloroform-*d*) δ 138.33, 133.35, 128.40, 127.65, 126.49, 125.92, 83.30, 24.75, 24.68, 14.86.

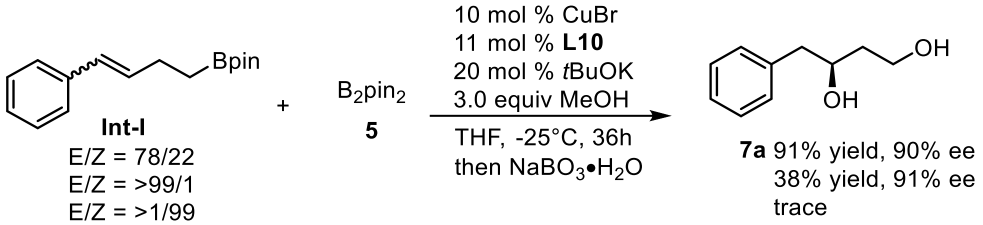

An oven-dried Schlenk tube with a stirred bar was charged with 10 mol % CuBr (1.4 mg, 0.0010 mmol), 11 mol % **L10** (4.2 mg, 0.0011 mmol) and 0.5 mL THF in argon atmosphere. The resulting solution was stirred at room temperature for 20 min, then *t*BuOK (2.2 mg, 0.0200 mmol) was allowed to add and stirred for additional 10 min, then 0.1 mmol **int-I** with E/Z ratio of 78/22 , >99/1 or >1/99 and 0.12 mmol B_2pin_2 **5** was added, The final solution was then cooled to -25 °C, followed by introduction of 3.0 equivalents of MeOH, and stirred for 36 h. Then the resulting solution was added 5 mL of DCM and filtered through a pad of silica gel, washed by PE/EA = 10/1 (10 mL x 3). The combined filtrates were concentrated and dissloved in THF/H_2O (2 mL, 1/1 v/v), then $NaBO_3 \cdot H_2O$ (0.4000 mmol, 40.0 mg) and was added. The resulting mixture was allowed to stir at room temperature for 4 h. The reaction mixture was diluted with EtOAc (5 mL) and H_2O (2 mL). The aqueous layer was extracted with EtOAc (5×2 mL). The organic layer was dried over anhydrous Na_2SO_4, filtered, and concentrated under vacuum. The residue was purified by column chromatography on silica gel to get the corresponding product **7a** (91% yield, 90% ee, 38% yield, 91% ee, and trace).

8. Calculation details

All the calculations were performed with the Gaussian 09 program.[4] All of the intermediates and transition states were optimized using ωB97X-D functional,[5] and the Lanl2DZ basis set[6] for Cu with polarization functions for Cu (ζf = 3.525)[7] and 6-31g(d) basis set for all other atoms. Frequency analysis was calculated at the same level of theory to verify the nature of stationary points. For each transition state, the intrinsic reaction coordinates (IRC)[8] analysis was also conducted to ensure that it indeed connects two relevant local minima.

Results and Discussion of Calculation

As shown in Figure 1, the step of *β*-C elimination involves the formation of **D** intermediates in E-/Z-configurations. Therefore, we calculated the energy differences of two transition states lead to intermediates **D** in E- or Z-configurations and tried to explain the reason why ligand **L17** shows better selectivity than other ligands by analysis catalytic pockets with Topographic Steric Map (Figure S1).[9] It was found that **L17Cu** catalyst has a relatively large $\%V_{Bur}$ of 55.4% while that of **L16Cu**, **L11Cu**, **L15Cu** are 51.7%, 49.3% and 48% respectively. The peculiar behavior of **L17Cu** catalysts has been ascribed to the large bite angle characterizing this ligand. The large bridge of **L17Cu** forces the ligand to assume a folded structure with the two phenyl substituents pointing up and facing each other, forming a hindered catalytic pocket, while other catalysts form flat catalytic pockets. Although **L15Cu** form a catalytic pocket with phenyl groups in similar orientation, its $\%V_{Bur}$ is smaller than that of **L17Cu**. In other words, **L15Cu** has a smaller bite angle, forming a flatter catalytic pocket and opening a larger gate for the reactant, which means the phenyl groups on phosphorous atoms do not perform blocking function effectively in catalysis during the reaction.

(a)

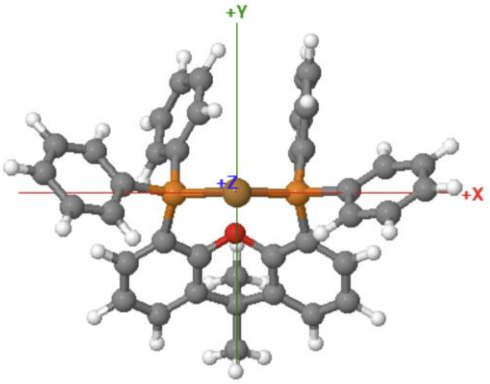

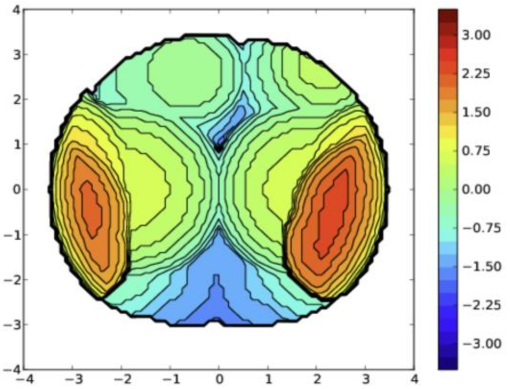

The orientation of **Cu_L17_E** Computed steric map of **Cu_L17_E**

%V Free	**%V Buried**	% V Tot/V Ex
44.6	**55.4**	99.9

bite angle (**LCuL**) = 111.06

(b)

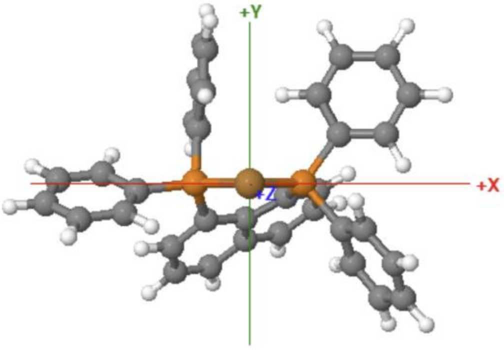

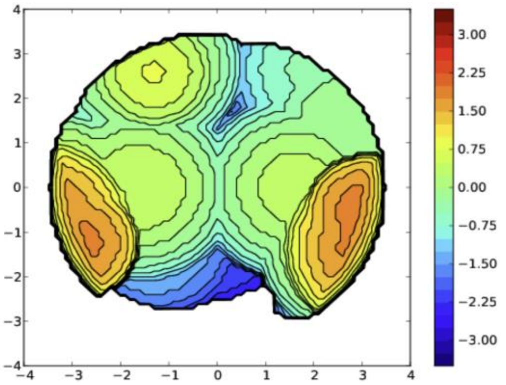

The orientation of **Cu_L16_E** Computed steric map of **Cu_L16_E**

%V Free	**%V Buried**	% V Tot/V Ex
47.8	**52.2**	99.9

bite angle (**LCuL**) = 90.34

(c)

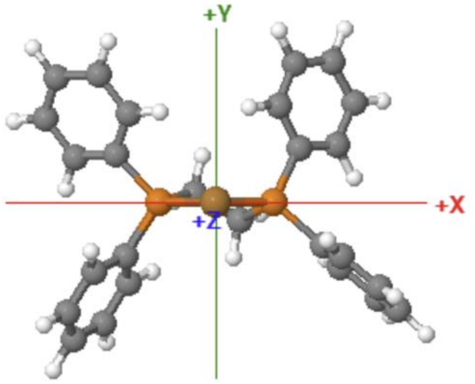

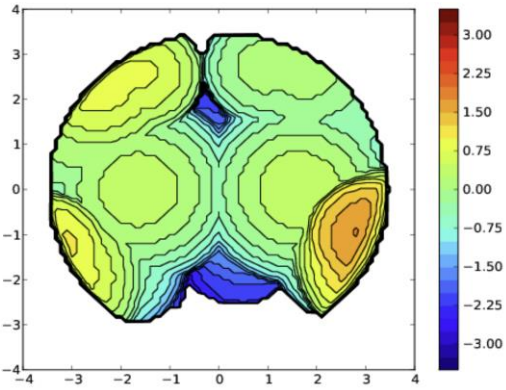

The orientation of **Cu_L11_E** Computed steric map of **Cu_L11_E**

%V Free	%V Buried	% V Tot/V Ex
50.7	**49.3**	99.9

bite angle (**LCuL**) = 89.72

(d)

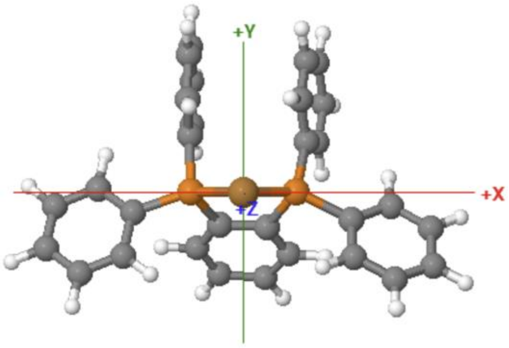

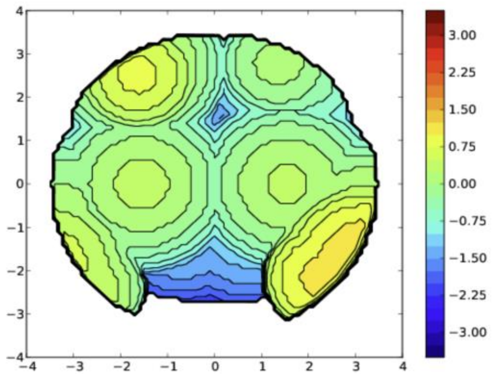

The orientation of **Cu_L15_E** Computed steric map of **Cu_L15_E**

%V Free	%V Buried	% V Tot/V Ex
52.0	**48.0**	99.9

bite angle (**LCuL**) = 88.33

Figure 1S. Computed steric maps **(a)-(d)** of metal-ligand complexes based on their optimized structures. The steric maps are viewed along the *z* axis. $\%V_{Bur}$, highlights a difference in steric bulkiness between these ligands. The orientation of complexes is indicated in the left panel.

In Figure 2S, the calculation shown that **L17** provides a 98% of calculated E/Z ratio, which is in perfect accordance with experimental result of 95%. Additionally, we conducted a carefully analysis of the structures of these transition states. It was found

that there are some short H···H contacts of 2.016 Å and 2.333 Å in transition state **ts_Cu_L17_Z** while these interaction distances in **ts_Cu_L17_E** are 2.197Å and 2.355Å, respectively, which indicates that the MCP part in **ts_Cu_L17_Z** has stronger repulsive interactions with phenyl groups of **L17**, resulting in it being disfavored.

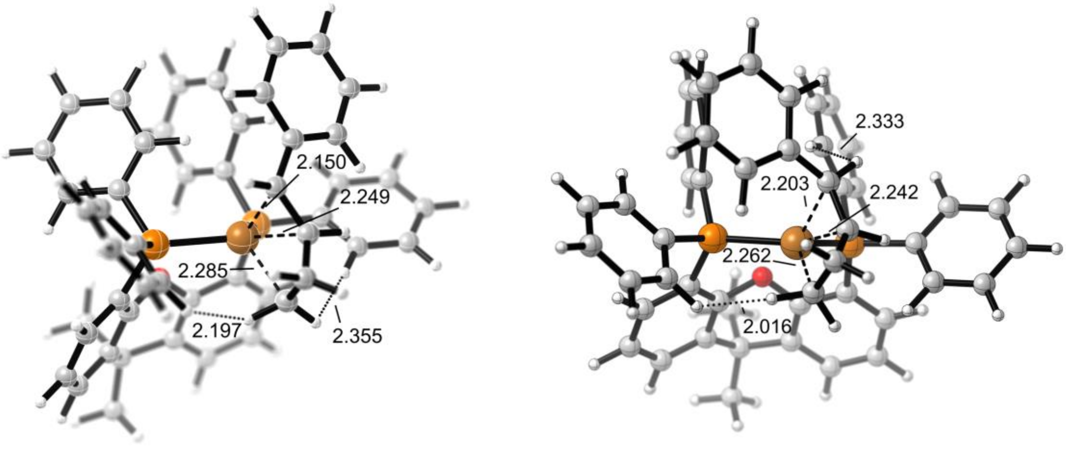

**ts_Cu_L17_E**	**ts_Cu_L17_Z**
ΔΔ**G** =0 kcal/mol	ΔΔ**G** =3.28 kcal/mol
exp. ratio (E/Z) = >19:1	cal. ratio (E/Z) = >19:1

Figure 2S. Comparison between ring-opening transition states leading to E- or Z-configurations.

In conclusion, **L17Cu** catalyst forms a relatively hindered catalytic pocket due to large bite angle characterizing **L17** ligand. The less repulsive interactions between MCP reactant and phenyl groups of ligands make intermediate **D** with E-configuration be favored in the reaction. Therefore, a high E/Z ratio of intermediate **D** during β-C elimination of MCP is formed by using **L17** as ligand.

9. Analytic data for the products

(S)-2,2'-(1-phenylbutane-1,4-diyl)bis(4,4,5,5-tetramethyl-1,3,2-dioxaborolane) (3a)

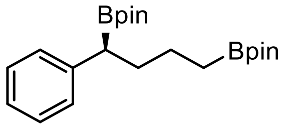

^{1}H NMR (400 MHz, Chloroform-*d*) δ 7.26-7.18 (m, 4H), 7.14-7.08 (m, 1H), 2.31 (t, *J* = 7.8 Hz, 1H), 1.88-1.78 (m, 1H), 1.72-1.65 (m, 1H), 1.43-1.35 (m, 2H), 1.22 (s, 12H), 1.19 (s, 6H), 1.18 (s, 6H), 0.82-0.72 (m, 2H). ^{13}C NMR (101 MHz, Chloroform-*d*) δ 143.4, 128.4, 128.18, 125.0, 83.2, 82.8, 35.3, 24.8, 24.82, 24.6, 24.6, 23.7. HRMS (ESI-ion trap) m/z: [M+Na]+ calcd for $C_{22}H_{36}B_2O_4Na$ 409.2692; found 409.2691.

(*S*)-1-phenylbutane-1,4-diol (4a)

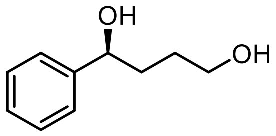

General procedure was used with methylenecyclopropane **1a** (13.0 mg, 0.1 mmol, 1.0 equiv.) and HBpin **2** (27.5 mg, 0.22 mmol, 2.2 equiv.) at rt for 36 h to afford **4a** as colorless oil (15.0 mg, 91% yield, 98% ee). $[\alpha]^{20}_{D}$= -39.7 (c 0.43, $CHCl_3$). ^{1}H NMR (400 MHz, Chloroform-*d*) δ 7.39-7.30 (m, 4H), 7.31-7.23 (m, 1H), 4.72 (t, *J* = 6.3 Hz, 1H), 3.75-3.61 (m, 2H), 2.36 (s, 2H), 1.86 (q, *J* = 7.2 Hz, 2H), 1.74-1.61 (m, 2H).^{13}C NMR (101 MHz, Chloroform-*d*) δ 144.7, 128.5, 127.5, 125.8, 74.4, 62.9, 36.2, 29.2. The enantiomeric excess was determined by Daicel Chiralcel IC (0.46 cm x 25 cm), Hexanes /IPA = 92 / 08, 1.0 mL/min, λ = 210 nm, t (major) = 25.3 min, t (minor) = 28.1 min. HRMS (ESI-ion trap) m/z: [M+H]+ calcd for $C_{10}H_{15}O_2$ 167.1067; found 167.1064.

(*S*)-1-(o-tolyl)butane-1,4-diol (4b)

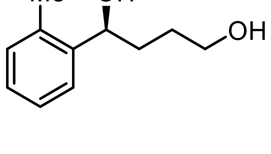

General procedure was used with methylenecyclopropane **1b** (14.4 mg, 0.1 mmol, 1.0 equiv.) and HBpin **2** (27.5 mg, 0.22 mmol, 2.2 equiv.) at rt for 36 h to afford **4b** as colorless oil (12.5 mg, 71% yield, 96% ee). $[\alpha]^{20}_{D}$= -71.5 (c 0.36, $CHCl_3$). ^{1}H NMR (400 MHz, Chloroform-*d*) δ 7.52-7.46 (m, 1H), 7.25-7.20 (m, 1H), 7.19-7.10 (m, 2H), 4.96 (dd, *J* = 7.7, 4.0 Hz, 1H), 3.77-3.63 (m, 2H), 2.56 (s, 2H), 2.33 (s, 3H), 1.84-1.71 (m, 4H). ^{13}C NMR (101 MHz, Chloroform-*d*) δ 142.9, 134.3, 130.4, 127.2, 126.3, 125.1, 70.7, 63.0, 35.2, 29.5, 19.1. The enantiomeric excess was determined by Daicel Chiralcel IC (0.46 cm x 25 cm), Hexanes /IPA = 93 / 07, 1.0 mL/min, λ = 210 nm, t (major) = 25.7 min, t (minor) = 29.8 min. HRMS (ESI-ion trap) m/z: [M+H]+ calcd for $C_{11}H_{17}O_2$ 181.1223; found 181.1221.

(*S*)-1-(m-tolyl)butane-1,4-diol (4c)

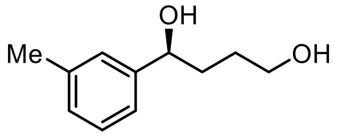

General procedure was used with methylenecyclopropane **1c** (14.4 mg, 0.1 mmol, 1.0 equiv.) and HBpin **2** (27.5 mg, 0.22 mmol, 2.2 equiv.) at rt for 36 h to afford **4c** as white solid (16.0 mg, 89% yield, 98% ee). m.p. 51.2-52.0 °C. $[\alpha]^{20}_{D}$= -47.8 (c 0.47, $CHCl_3$). ^{1}H NMR (400 MHz, Chloroform-*d*) δ 7.26-7.20 (m, 1H), 7.18-7.11 (m, 2H), 7.10-7.04 (m, 1H), 4.67 (t, *J* = 6.3 Hz, 1H), 3.72-3.61 (m, 2H), 2.46 (s, 2H), 2.35 (s, 3H), 1.84 (q, *J* = 6.9 Hz, 1H), 1.73-1.61 (m, 2H). ^{13}C NMR (101 MHz, Chloroform-*d*) δ 144.6, 138.0, 128.3, 128.2, 126.4, 122.8, 74.4, 62.8, 36.2, 29.2, 21.4. The enantiomeric excess was determined by Daicel Chiralcel IC (0.46 cm x 25 cm), Hexanes /IPA = 93 / 07, 1.0 mL/min, λ = 210 nm, t (major) = 28.5 min, t (minor) = 32.9 min. HRMS (ESI-ion trap) m/z: [M+H]+ calcd for $C_{11}H_{17}O_2$ 181.1223; found 181.1221.

(*S*)-1-(p-tolyl)butane-1,4-diol (4d)

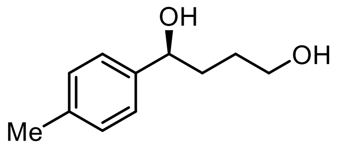

General procedure was used with methylenecyclopropane **1d** (14.4 mg, 0.1 mmol, 1.0 equiv.) and HBpin **2** (27.5 mg, 0.22 mmol, 2.2 equiv.) at rt for 36 h to afford **4d** as colorless oil (16.5 mg, 92% yield, >99% ee). $[\alpha]^{20}_{D}$= -33.9 (c 0.48, $CHCl_3$). ^1H NMR (400 MHz, Chloroform-*d*) δ 7.24 (d, *J* = 8.1 Hz, 2H) 7.15 (d, *J* = 7.9 Hz, 2H), 4.68 (t, *J* = 6.3 Hz, 1H), 3.73-3.61 (m, 2H), 2.53-2.40 (m, 1H), 2.34 (s, 3H), 1.87-1.80 (m, 2H), 1.72-1.62 (m, 2H). ^{13}C NMR (101 MHz, Chloroform-*d*) δ 141.7, 137.2, 129.2, 125.8, 7.31, 62.9, 36.2, 29.3, 21.2. The enantiomeric excess was determined by Daicel Chiralcel IC (0.46 cm x 25 cm), Hexanes /IPA = 93 / 07, 1.0 mL/min, λ = 210 nm, t (major) = 35.9 min, t (minor) = 38.3 min. HRMS (ESI-ion trap) m/z: [M+H]+ calcd for $C_{11}H_{17}O_2$ 181.1223; found 181.1221.

(*S*)-1-(4-(tert-butyl)phenyl)butane-1,4-diol (4e)

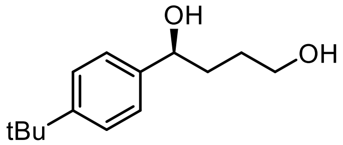

General procedure was used with methylenecyclopropane **1e** (18.6 mg, 0.1 mmol, 1.0 equiv.) and HBpin **2** (27.5 mg, 0.22 mmol, 2.2 equiv.) at rt for 36 h to afford **4e** as white solid (17.0 mg, 77% yield, >99% ee). m.p. 60.5-61.3 °C. $[\alpha]^{20}_{D}$= -34.2 (c 0.50, $CHCl_3$). ^1H NMR (400 MHz, Chloroform-*d*) δ 7.37-7.31 (m, 2H), 7.26-7.23 (m, 2H), 4.66 (t, *J* = 6.3 Hz, 1H), 3.71-3.57 (m, 2H), 2.43 (s, 2H), 1.88-1.75 (m, 2H), 1.71-1.59 (m, 2H), 1.29 (s, 9H). ^{13}C NMR (101 MHz, Chloroform-*d*) δ 150.5, 141.7, 125.5, 125.4, 74.3, 62.9, 36.1, 34.6, 31.4, 29.4. The enantiomeric excess was determined by Daicel Chiralcel IB (0.46 cm x 25 cm), Hexanes /IPA = 93 / 07, 1.0 mL/min, λ = 210 nm, t (major) = 35.2 min, t (minor) = 39.7 min. HRMS (ESI-ion trap) m/z: [M+H]+ calcd for $C_{14}H_{23}O_2$ 223.1693; found 223.1690.

(*S*)-1-(3-methoxyphenyl)butane-1,4-diol (4f)

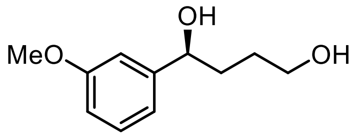

General procedure was used with methylenecyclopropane **1f** (16.0 mg, 0.1 mmol, 1.0 equiv.) and HBpin **2** (27.5 mg, 0.22 mmol, 2.2 equiv.) at rt for 36 h to afford **4f** as white solid (19.0 mg, 97% yield, 95% ee). m.p. 66.0-67.0 °C. $[\alpha]^{20}_{D}$= -30.9 (c 0.57, $CHCl_3$). ^1H NMR (400 MHz, Chloroform-*d*) δ 7.26-7.22 (m, 1H), 6.94-6.87 (m, 2H), 6.81-6.78 (m, 1H), 4.67 (t, *J* = 6.2 Hz, 1H), 3.79 (s, 3H), 3.70-3.57 (m, 2H), 2.75 (s, 2H), 1.87-1.79 (m, 2H), 1.71-1.59 (m, 2H). ^{13}C NMR (101 MHz, Chloroform-*d*) δ 159.7, 146.5, 129.5, 118.1, 112.8, 111.3, 74.3, 62.8, 55.3, 36.3, 29.2. The enantiomeric excess was determined by Daicel Chiralcel IC (0.46 cm x 25 cm), Hexanes /IPA = 85 / 15, 1.0 mL/min, λ = 210 nm, t (major) = 35.9 min, t (minor) = 38.3 min. HRMS (ESI-ion trap) m/z: [M+Na]+ calcd for $C_{11}H_{16}O_3Na$ 219.0992; found 219.0989.

(*S*)-1-(4-methoxyphenyl)butane-1,4-diol (4g)

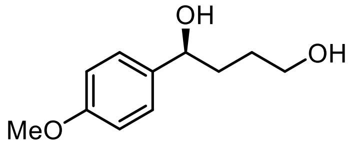

General procedure was used with methylenecyclopropane **1g** (16.0 mg, 0.1 mmol, 1.0 equiv.) and HBpin **2** (27.5 mg, 0.22 mmol, 2.2 equiv.) at 40 °C for 48 h to afford **4g** as white solid (12.2 mg, 62% yield, 97% ee). m.p. 48.1-49.3 °C. $[\alpha]^{20}_{D}$= -24.4 (c 0.34, $CHCl_3$). ^1H NMR (400 MHz, Chloroform-*d*) δ 7.28-7.25 (m, 2H), 6.89-6.85 (m, 2H), 4.66 (dd, *J* = 7.4, 5.4 Hz, 1H), 3.80 (s, 3H), 3.66 (q, *J* = 6.3 Hz, 2H), 2.32 (s, 2H), 1.89-1.78 (m, 2H), 1.70-1.58 (m, 2H). ^{13}C NMR (101 MHz, Chloroform-*d*) δ 159.0, 136.9, 127.1, 113.8, 74.1, 62.9, 55.3, 36.2, 29.3. The enantiomeric excess was determined by Daicel Chiralcel ID (0.46 cm x 25 cm), Hexanes /IPA = 85 / 15, 1.0 mL/min, λ = 214 nm, t (minor) = 16.3 min, t (major) = 17.2 min. HRMS (ESI-ion trap) m/z: [M+H]+ calcd for $C_{11}H_{17}O_3$ 197.1172; found 197.1167.

(*S*)-1-(4-(trifluoromethoxy)phenyl)butane-1,4-diol (4h)

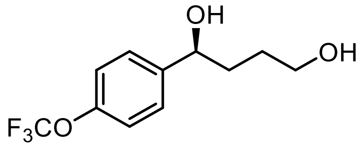

General procedure was used with methylenecyclopropane **1h** (21.4 mg, 0.1 mmol, 1.0 equiv.) and HBpin **2** (27.5 mg, 0.22 mmol, 2.2 equiv.) at rt for 36 h to afford **4h** as colorless oil (24.5 mg, 98% yield, 98% ee). $[\alpha]^{20}_{D}$= -14.0 (c 0.75, $CHCl_3$). ^1H NMR (400 MHz, Chloroform-*d*) δ 7.41-7.35 (m, 2H), 7.22-7.15 (m, 2H), 4.74 (dd, *J* = 7.4, 5.1 Hz, 1H), 3.75-3.63 (m, 2H), 2.55 (s, 2H), 1.90-1.79 (m, 2H), 1.75-1.61 (m, 2H). ^{19}F NMR (377 MHz, Chloroform-*d*) δ -57.89. ^{13}C NMR (151 MHz, Chloroform-*d*) δ 148.4, 143.4, 127.2, 120.9, 120.5 (q, *J* = 257.0 Hz), 73.6, 62.8, 36.5, 29.0. The enantiomeric excess was determined by Daicel Chiralcel IA (0.46 cm x 25 cm), Hexanes /IPA = 99 / 01, 1.0 mL/min, λ = 214 nm, t (major) = 37.9 min, t (minor) = 40.7 min. HRMS (ESI-ion trap) m/z: [M+H]+ calcd for $C_{11}H_{14}F_3O_3$ 251.0890; found 251.0885.

(*S*)-1-(4-(benzyloxy)phenyl)butane-1,4-diol (4i)

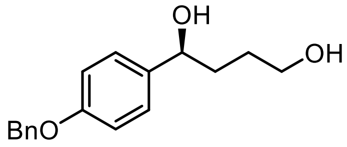

General procedure was used with methylenecyclopropane **1i** (23.6mg, 0.1 mmol, 1.0 equiv.) and HBpin **2** (27.5 mg, 0.22 mmol, 2.2 equiv.) at rt for 36 h to afford **4i** as white solid (12.5 mg, 46% yield, >99% ee). m.p. 115.3-116.2 °C. $[\alpha]^{20}_{D}$= -30.3 (c 0.35, $CHCl_3$). ^1H NMR (400 MHz, Chloroform-*d*) δ 7.47-7.35 (m, 4H), 7.37-7.30 (m, 1H), 7.31-7.24 (m, 3H), 6.99-6.92 (m, 2H), 5.06 (s, 2H), 4.68 (dd, *J* = 7.3, 5.4 Hz, 1H), 3.75-3.61 (m, 2H), 2.25 (br, H)1.90-1.79 (m, 2H), 1.72-1.61 (m, 2H). ^{13}C NMR (101 MHz, Chloroform-*d*) δ 158.4, 137.2, 137.1, 128.7, 128.1, 127.6, 127.2, 114.9, 74.2, 70.2, 63.1, 36.2, 29.5. The enantiomeric excess was determined by Daicel Chiralcel IA (0.46 cm x 25 cm), Hexanes /IPA = 90 / 10, 1.0 mL/min, λ = 210 nm, t (major) = 22.1 min, t (minor) = 24.0 min. HRMS (ESI-ion trap) m/z: [M+Na]+ calcd for $C_{17}H_{20}O_3Na$ 295.1305; found 295.1301.

(*S*)-1-(2-fluorophenyl)butane-1,4-diol (4j)

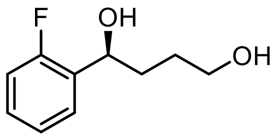

General procedure was used with methylenecyclopropane **1j** (14.8 mg, 0.1 mmol, 1.0 equiv.) and HBpin **2** (27.5 mg, 0.22 mmol, 2.2 equiv.) at rt for 36 h to afford **4j** as colorless oil (17.0 mg, 92% yield, 95% ee). $[\alpha]^{20}_{D}$= -32.6 (c 0.5, $CHCl_3$). ^1H NMR (600 MHz, Chloroform-*d*) δ 7.52-7.43 (m, 1H), 7.25-7.19 (m, 1H), 7.18-7.09 (m, 1H), 7.04-6.96 (m, 1H), 5.04 (dd, *J* = 7.8, 4.6 Hz, 1H), 3.75-3.60 (m, 2H), 2.76 (s, 2H), 1.92-1.83 (m, 2H), 1.75-1.64 (m, 2H). ^{19}F NMR (565 MHz, Chloroform-*d*) δ -119.68. ^{13}C NMR (151 MHz, Chloroform-*d*) δ 159.6 (d, *J* = 245.2 Hz), 131.6 (d, *J* = 13.1 Hz), 128.7 (d, *J* = 8.2 Hz), 127.2 (d, *J* = 4.4 Hz), 124.3 (d, *J* = 3.3 Hz), 115.2 (d, *J* = 21.9 Hz), 68.1 (d, *J* = 2.5 Hz), 62.8, 35.2, 29.0. The enantiomeric excess was determined by Daicel Chiralcel ID (0.46 cm x 25 cm), Hexanes /IPA = 95 / 05, 1.0 mL/min, λ = 214 nm, t (minor) = 27.8 min, t (major) = 29.0 min. HRMS (ESI-ion trap) m/z: [M+Na]+ calcd for $C_{10}H_{13}FO_2Na$ 207.0792; found 207.0792.

(*S*)-1-(4-fluorophenyl)butane-1,4-diol (4k)

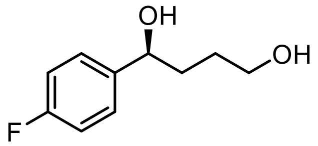

General procedure was used with methylenecyclopropane **1k** (14.8 mg, 0.1 mmol, 1.0 equiv.) and HBpin **2** (27.5 mg, 0.22 mmol, 2.2 equiv.) at rt for 36 h to afford **4k** as white solid (15.0 mg, 82% yield, >99% ee). m.p. 44.0-45.0 °C. $[\alpha]^{20}_{D}$= -31.4 (c 0.43, $CHCl_3$). ^1H NMR (400 MHz, Chloroform-*d*) δ 7.36-7.27 (m, 2H), 7.07-6.97 (m, 2H), 4.71 (t, *J* = 6.3 Hz, 1H), 3.75-3.61 (m, 2H), 2.43 (s, 2H), 1.83 (q, *J* = 7.1 Hz, 2H), 1.73-1.59 (m, 2H). ^{19}F NMR (377 MHz, Chloroform-*d*) δ -115.26. ^{13}C NMR (101 MHz, Chloroform-*d*) δ 162.1 (d, *J* = 245.1 Hz), 140.5 (d, *J* = 3.0 Hz), 127.4 (d, *J* = 8.0 Hz), 115.3 (d, *J* = 21.3 Hz), 73.8, 62.9, 36.5, 29.1. The enantiomeric excess was determined by Daicel Chiralcel IC (0.46 cm x 25 cm), Hexanes /IPA = 93 / 07, 0.8 mL/min, λ =

214 nm, t (major) = 22.0 min, t (minor) = 23.8 min. HRMS (ESI-ion trap) m/z: [M+H]+ calcd for $C_{10}H_{14}FO_2$ 185.0972; found 185.0970.

(*S*)-1-(4-(trifluoromethyl)phenyl)butane-1,4-diol (4l)

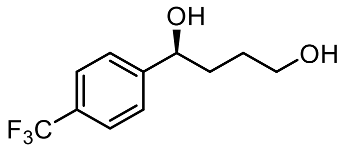

General procedure was used with methylenecyclopropane **1l** (19.8 mg, 0.1 mmol, 1.0 equiv.) and HBpin **2** (27.5 mg, 0.22 mmol, 2.2 equiv.) at rt for 36 h to afford **4** as colorless oil (22.0 mg, 94% yield, 98% ee). $[\alpha]^{20}_{D}$= -37.9 (c 0.57, $CHCl_3$). ^1H NMR (600 MHz, Chloroform-*d*) δ 7.63-7.53 (m, 2H), 7.49-7.40 (m, 2H), 4.76 (dd, *J* = 8.1, 4.4 Hz, 1H), 3.72-3.61 (m, 2H), 2.89 (s, 2H), 1.89-1.79 (m, 2H), 1.71-1.61 (m, 2H). ^{19}F NMR (565 MHz, Chloroform-*d*) δ -62.44. ^{13}C NMR (151 MHz, Chloroform-*d*) δ 148.7, 129.6 (q, *J* = 32.4 Hz), 126.0, 125.3 (q, *J* = 3.8 Hz), 124.2 (q, *J* = 271.9 Hz), 73.6, 62.7, 36.6, 28.8. The enantiomeric excess was determined by Daicel Chiralcel IF (0.46 cm x 25 cm), Hexanes /IPA = 96 / 04, 0.5 mL/min, λ = 210 nm, t (major) = 37.9 min, t (minor) = 40.7 min. HRMS (ESI-ion trap) m/z: [M+H]+ calcd for $C_{11}H_{14}F_3O_2$ 235.0940; found 235.0937.

(*S*)-1-(3-bromophenyl)butane-1,4-diol (4m)

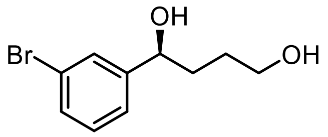

General procedure was used with methylenecyclopropane **1m** (20.7 mg, 0.1 mmol, 1.0 equiv.) and HBpin **2** (27.5 mg, 0.22 mmol, 2.2 equiv.) at rt for 36 h to afford **4m** as white solid (18.8 mg, 77% yield, 90% ee). m.p. 59.2-60.6 °C. $[\alpha]^{20}_{D}$= -30.5 (c 0.56, $CHCl_3$). ^1H NMR (400 MHz, Chloroform-*d*) δ 7.52-7.48 (m, 1H), 7.41-7.35 (m, 1H), 7.26-7.16 (m, 2H), 4.66 (dd, *J* = 7.6, 4.8 Hz, 1H), 3.72-3.60 (m, 2H), 2.85 (s, 2H), 1.87-1.77 (m, 2H), 1.70-1.61 (m, 2H). ^{13}C NMR (101 MHz, Chloroform-*d*) δ 147.1, 130.4, 130.0, 128.9, 124.4, 122.6, 73.6, 62.8, 36.5, 29.0. The enantiomeric excess was

determined by Daicel Chiralcel IC (0.46 cm x 25 cm), Hexanes /IPA = 93 / 07, 1.0 mL/min, λ = 210 nm, t (major) = 23.0 min, t (minor) = 26.5 min. HRMS (ESI-ion trap) m/z: [M+H]+ calcd for $C_{10}H_{14}BrO_2$ 245.0172; found 245.0166.

(*S*)-1-([1,1'-biphenyl]-4-yl)butane-1,4-diol (4n)

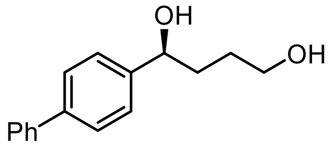

General procedure was used with methylenecyclopropane **1n** (20.6 mg, 0.1 mmol, 1.0 equiv.) and HBpin **2** (27.5 mg, 0.22 mmol, 2.2 equiv.) at rt for 36 h to afford **4n** as white solid (18.0 mg, 74% yield, 96% ee). m.p. 73.0-74.0 °C. $[\alpha]^{20}_{D}$= -42.9 (c 0.53, $CHCl_3$). ^{1}H NMR (400 MHz, Chloroform-*d*) δ 7.61-7.53 (m, 4H), 7.46-7.38 (m, 4H), 7.37-7.32 (m, 1H), 4.74 (t, *J* = 6.3 Hz, 1H), 3.75-3.61 (m, 2H), 3.00 (s, 2H), 1.88 (q, *J* = 6.9 Hz, 2H), 1.76-1.64 (m, 2H). ^{13}C NMR (101 MHz, Chloroform-*d*) δ 143.8, 140.8, 140.4, 128.8, 127.3, 127.2, 127.1, 126.3, 74.1, 62.8, 36.4, 29.2. The enantiomeric excess was determined by Daicel Chiralcel ID (0.46 cm x 25 cm), Hexanes /IPA = 90 / 10, 1.0 mL/min, λ = 214 nm, t (major) = 23.7 min, t (minor) = 26.3 min. HRMS (ESI-ion trap) m/z: [M+H]+ calcd for $C_{16}H_{19}O_2$ 243.1380; found 243.1374.

(*S*)-1-(naphthalen-1-yl)butane-1,4-diol (4o)

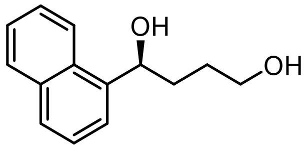

General procedure was used with methylenecyclopropane **1o** (18.0 mg, 0.1 mmol, 1.0 equiv.) and HBpin **2** (27.5 mg, 0.22 mmol, 2.2 equiv.) at rt for 36 h to afford **4o** as white solid (18.0 mg, 83% yield, 84% ee). m.p. 85.0-86.0 °C. $[\alpha]^{20}_{D}$= -60.6 (c 0.53, $CHCl_3$). ^{1}H NMR (400 MHz, Chloroform-*d*) δ 8.08-8.00 (m, 1H), 7.90-7.82 (m, 1H), 7.79-7.74 (m, 1H), 7.67-7.62 (m, 1H), 7.54-7.41 (m, 3H), 5.49 (dd, *J* = 8.3, 3.7 Hz, 1H), 3.77-3.63 (m, 2H), 2.65 (s, 2H), 2.12-2.04 (m, 1H), 1.98-1.89 (m, 1H), 1.82-1.72 (m, 2H). ^{13}C NMR (101 MHz, Chloroform-*d*) δ 140.3, 133.8, 130.2, 129.0, 127.9, 126.0, 125.5,

125.5, 123.0, 122.8, 71.1, 63.0 35.6, 29.5. The enantiomeric excess was determined by Daicel Chiralcel IC (0.46 cm x 25 cm), Hexanes /IPA = 85 / 15, 1.0 mL/min, λ = 214 nm, t (major) = 11.8 min, t (minor) = 15.4 min. HRMS (ESI-ion trap) m/z: [M+Na]+ calcd for $C_{14}H_{16}O_2Na$ 239.1043; found 239.1042.

(*S*)-1-(naphthalen-2-yl)butane-1,4-diol (4p)

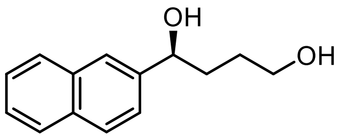

General procedure was used with methylenecyclopropane **1p** (18.0 mg, 0.1 mmol, 1.0 equiv.) and HBpin **2** (27.5 mg, 0.22 mmol, 2.2 equiv.) at rt for 36 h to afford **4p** as white solid (18.0 mg, 83% yield, 95% ee). m.p. 58.0-59.0 °C. $[\alpha]^{20}_{D}$= -38.1 (c 0.53, $CHCl_3$). ^{1}H NMR (600 MHz, Chloroform-*d*) δ 7.86-7.78 (m, 3H), 7.77 (s, 1H), 7.51-7.41 (m, 3H), 4.85 (t, *J* = 6.3 Hz, 1H), 3.71-3.61 (m, 2H), 2.62 (s, 2H), 1.92 (q, *J* = 6.9 Hz, 2H), 1.74-1.62 (m, 2H). ^{13}C NMR (151 MHz, Chloroform-*d*) δ 142.1, 133.3, 132.9, 128.3, 127.9, 127.7, 126.2, 125.8, 124.5, 124.1, 74.4, 62.8, 36.2, 29.2. The enantiomeric excess was determined by Daicel Chiralcel ID (0.46 cm x 25 cm), Hexanes /IPA = 90 / 10, 1.0 mL/min, λ = 254 nm, t (minor) = 29.7 min, t (major) = 32.2 min. HRMS (ESI-ion trap) m/z: [M+Na]+ calcd for $C_{14}H_{16}O_2Na$ 239.1043; found 239.1042.

(*S*)-1-(benzofuran-3-yl)butane-1,4-diol (4q)

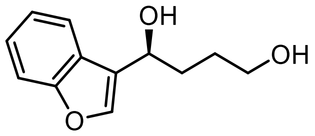

General procedure was used with methylenecyclopropane **1q** (17.0 mg, 0.1 mmol, 1.0 equiv.) and HBpin **2** (27.5 mg, 0.22 mmol, 2.2 equiv.) at rt for 60 h to afford **4q** as white solid (13.3 mg, 65% yield, 91% ee). m.p. 54.0-55.0 °C. $[\alpha]^{20}_{D}$= -29.2 (c 0.38, $CHCl_3$). ^{1}H NMR (400 MHz, Chloroform-*d*) δ 7.69-7.64 (m, 1H), 7.56 (s, 1H), 7.50-7.45 (m, 1H), 7.33-7.26 (m, 1H), 7.26-7.20 (m, 1H), 4.98 (dd, *J* = 7.6, 4.8, Hz, 1H), 3.79-3.59 (m, 2H), 2.50 (s, 2H), 2.12-1.98 (m, 2H), 1.80-1.66 (m, 2H). ^{13}C NMR (101

MHz, Chloroform-*d*) δ 155.7, 141.3, 126.0, 124.5, 124.0, 122.6, 120.5, 111.7, 67.1, 62.8, 34.4, 29.1. The enantiomeric excess was determined by Daicel Chiralcel ID (0.46 cm x 25 cm), Hexanes /IPA = 92 / 08, 1.0 mL/min, λ = 220 nm, t (major) = 33.2 min, t (minor) = 39.6 min. HRMS (ESI-ion trap) m/z: [M+H]+ calcd for $C_{12}H_{15}O_3$ 207.1016; found 207.1013.

(*S*)-1-(3,4-difluorophenyl)butane-1,4-diol (4r)

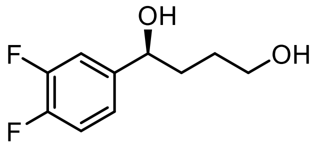

General procedure was used with methylenecyclopropane **1r** (16.6 mg, 0.1 mmol, 1.0 equiv.) and HBpin **2** (27.5 mg, 0.22 mmol, 2.2 equiv.) at rt for 36 h to afford **4r** as white solid (15.6 mg, 77% yield, 97% ee). m.p. 66.0-67.0 °C. $[\alpha]^{20}_{D}$= -39.3 (c 0.45, $CHCl_3$). ^{1}H NMR (400 MHz, Chloroform-*d*) δ 7.23-7.02 (m, 3H), 4.70 (dd, *J* = 7.7, 4.7 Hz, 1H), 3.77-3.64 (m, 2H), 2.35 (s, H), 1.87-1.78 (m, 2H), 1.71-1.62 (m, 2H). ^{19}F NMR (377 MHz, Chloroform-*d*) δ -137.62 (d, *J* = 21.1 Hz), -139.98 (d, *J* = 21.4 Hz). ^{13}C NMR (101 MHz, Chloroform-*d*) δ 151.2 (dd, *J* = 83.9, 12.6 Hz), 148.7 (dd, *J* = 83.0, 12.6 Hz), 142.0 (dd, *J* = 4.7, 3.8 Hz), 121.7 (dd, *J* = 6.3, 3.5 Hz), 117.2 (d, *J* = 17.2 Hz), 114.8 (d, *J* = 17.5 Hz), 73.2, 62.8, 36.8, 29.0. The enantiomeric excess was determined by Daicel Chiralcel ID (0.46 cm x 25 cm), Hexanes /IPA = 95 / 05, 1.0 mL/min, λ = 210 nm, t (minor) = 37.6 min, t (major) = 40.4 min. HRMS (ESI-ion trap) m/z: [M+H]+ calcd for $C_{10}H_{13}F_2O_2$ 203.0878; found 203.0878.

(*S*)-1-(3,4,5-trimethoxyphenyl)butane-1,4-diol (4s)

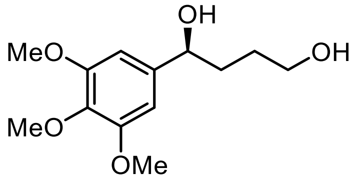

General procedure was used with methylenecyclopropane **1s** (22.0 mg, 0.1 mmol, 1.0 equiv.) and HBpin **2** (27.5 mg, 0.22 mmol, 2.2 equiv.) at rt for 60 h to afford **4s** as colorless oil (14.0 mg, 55% yield, 91% ee). $[\alpha]^{20}_{D}$= -27.5 (c 0.4, $CHCl_3$). ^{1}H NMR (400

MHz, Chloroform-*d*) δ 6.58 (s, 2H), 4.70-4.62 (m, 1H), 3.86 (s, 6H), 3.83 (s, 3H), 3.75-3.65 (m, 2H), 2.04 (s, 2H), 1.88-1.80 (m, 2H), 1.76-1.63 (m, 2H). ^{13}C NMR (101 MHz, Chloroform-*d*) δ 152.2, 139.6, 136.1, 101.6, 73.6, 61.9, 59.8, 55.1, 35.4, 28.3. The enantiomeric excess was determined by Daicel Chiralcel ID (0.46 cm x 25 cm), Hexanes /IPA = 75 / 25, 1.0 mL/min, λ = 214 nm, t (major) = 16.8 min, t (minor) = 21.0 min. HRMS (ESI-ion trap) m/z: [M+Na]+ calcd for $C_{13}H_{20}O_5Na$ 279.1203; found 279.1198.

(*R*)-2,2'-(4-phenylbutane-1,3-diyl)bis(4,4,5,5-tetramethyl-1,3,2-dioxaborolane) (6a)

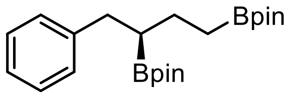

^{1}H NMR (400 MHz, Chloroform-*d*) δ 7.25-7.17 (m, 4H), 7.15-7.09 (m, 1H), 2.76-2.61 (m, 2H), 1.61-1.51 (m, 2H), 1.40-1.32 (m, 1H), 1.23 (s, 12H), 1.14 (s, 6H), 1.10 (s, 6H), 0.87-0.77 (m, 2H). ^{13}C NMR (101 MHz, Chloroform-*d*) δ 142.4, 128.9, 128.0, 125.5, 82.9, 82.9, 37.3, 25.5, 24.8, 24.8, 24.8. HRMS (ESI-ion trap) m/z: [M+H]+ calcd for $C_{22}H_{37}B_2O_4$ 387.2872; found 387.2864.

(*S*)-4-phenylbutane-1,3-diol (7a)

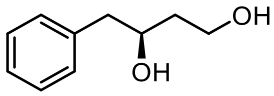

General procedure was used with methylenecyclopropane **1a** (15.6 mg, 0.12 mmol, 1.2 equiv.) and HBpin **2** (12.8 mg, 0.1 mmol, 1.0 equiv.) at rt for 36 h and B_2pin_2 **5** (30.5 mg, 0.12 mmol, 1.2 equiv.) at -25 °C for 48 h to afford **7a** as colorless oil (14.5 mg, 87% yield, 90% ee). $[\alpha]^{20}_{D}$= -18.8 (c, 0.73 $CHCl_3$). ^{1}H NMR (400 MHz, Chloroform-*d*) δ 7.37-7.29 (m, 2H), 7.26-7.18 (m, 3H), 4.14-4.05 (m, 1H), 3.95-3.78 (m, 2H), 2.86-2.73 (m, 2H), 2.42 (s, 2H), 1.86-1.72 (m, 2H). ^{13}C NMR (101 MHz, Chloroform-*d*) δ 138.0, 129.4, 128.7, 126.7, 73.1, 61.8, 44.4, 37.8. The enantiomeric excess was determined by Daicel Chiralcel IC (0.46 cm x 25 cm), Hexanes /IPA = 92 / 08, 1.0

mL/min, λ = 210 nm, t (major) = 21.9 min, t (minor) = 27.0 min. HRMS (ESI-ion trap) m/z: [M+H]+ calcd for $C_{10}H_{15}O_2$ 167.1067; found 167.1065.

(*S*)-4-(o-tolyl)butane-1,3-diol (7b)

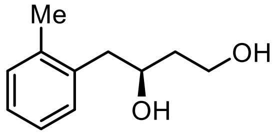

General procedure was used with methylenecyclopropane **1b** (17.3mg, 0.12 mmol, 1.2 equiv.) and HBpin **2** (12.8 mg, 0.1 mmol, 1.0 equiv.) at rt for 36 h and B_2pin_2 **5** (30.5 mg, 0.12 mmol, 1.2 equiv.) at -25 °C for 52 h to afford **7b** as colorless oil (10.5 mg, 58% yield, 85% ee). $[\alpha]^{20}_{D}$= -16.2 (c 0.28, $CHCl_3$). ^{1}H NMR (400 MHz, Chloroform-*d*) δ 7.18-7.11 (m, 4H), 4.14-4.03 (m, 1H), 3.97-3.74 (m, 2H), 2.86-2.74 (m, 2H), 2.34 (s, 3H), 1.84-1.74 (m, 2H). ^{13}C NMR (101 MHz, Chloroform-*d*) δ 136.7, 136.3, 130.6, 130.2, 126.8, 126.1, 72.2, 61.9, 41.5, 38.0, 19.7. The enantiomeric excess was determined by Daicel Chiralcel IC (0.46 cm x 25 cm), Hexanes /IPA = 93 / 07, 1.0 mL/min, λ = 254 nm, t (major) = 19.2 min, t (minor) = 22.6 min. HRMS (ESI-ion trap) m/z: [M+H]+ calcd for $C_{11}H_{17}O_2$ 181.1223; found 181.1222.

(*S*)-4-(4-(tert-butyl)phenyl)butane-1,3-diol (7c)

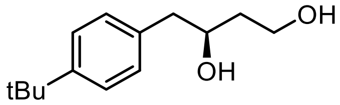

General procedure was used with methylenecyclopropane **1c** (22.3 mg, 0.12 mmol, 1.2 equiv.) and HBpin **2** (12.8 mg, 0.1 mmol, 1.0 equiv.) at rt for 36 h and B_2pin_2 **5** (30.5 mg, 0.12 mmol, 1.2 equiv.) at -25 °C for 52 h to afford **7c** as white solid (9.0 mg, 41% yield, 85% ee). m.p. 50.0-51.0 °C. $[\alpha]^{20}_{D}$= -12.4 (c 0.23, $CHCl_3$). ^{1}H NMR (400 MHz, Chloroform-*d*) δ 7.38-7.30 (m, 2H), 7.19-7.09 (m, 2H), 4.13-4.04 (m, 1H), 3.94-3.77 (m, 2H), 2.84-2.67 (m, 2H), 2.41 (s, 2H), 1.82-1.74 (m, 2H), 1.31 (s, 9H). ^{13}C NMR (101 MHz, Chloroform-*d*) δ 149.5, 134.8, 129.1, 125.6, 73.2, 61.9, 43.8, 37.8, 34.5, 31.4. The enantiomeric excess was determined by Daicel Chiralcel ID (0.46 cm x 25 cm), Hexanes /IPA = 95 / 05, 1.0 mL/min, λ = 214 nm, t (major) = 12.2 min, t (minor)

= 13.6 min. HRMS (ESI-ion trap) m/z: [M+H]+ calcd for $C_{14}H_{23}O_2$ 223.1693; found 223.1691.

(*S*)-4-(3-methoxyphenyl)butane-1,3-diol (7d)

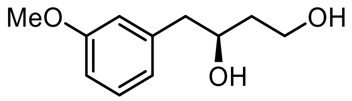

General procedure was used with methylenecyclopropane **1d** (19.2 mg, 0.12 mmol, 1.2 equiv.) and HBpin **2** (12.8 mg, 0.1 mmol, 1.0 equiv.) at rt for 36 h and B_2pin_2 **5** (30.5 mg, 0.12 mmol, 1.2 equiv.) at -25 °C for 48 h to afford **7d** as colorless oil (14.0 mg, 71% yield, 87% ee). $[\alpha]^{20}_{D}$= -11.0 (c 0.4, $CHCl_3$). ^1H NMR (400 MHz, Chloroform-*d*) δ 7.26-7.20 (m, 1H), 6.82-6.75 (m, 3H), 4.13-4.04 (m, 1H), 3.92-3.75 (m, 5H), 2.82-2.69 (m, 2H), 2.46 (s, 2H), 1.81-1.71 (m, 2H).^{13}C NMR (101 MHz, Chloroform-*d*) δ 159.8, 139.6, 129.7, 121.7, 115.1, 111.9, 73.0, 61.7, 55.2, 44.4, 37.8. The enantiomeric excess was determined by Daicel Chiralcel IC (0.46 cm x 25 cm), Hexanes /IPA = 92 / 1508, 1.0 mL/min, λ = 210 nm, t (major) = 33.9 min, t (minor) = 42.4 min. HRMS (ESI-ion trap) m/z: [M+H]+ calcd for $C_{11}H_{17}O_3$ 197.1172; found 197.1167.

(*S*)-4-(4-(trifluoromethoxy)phenyl)butane-1,3-diol (7e)

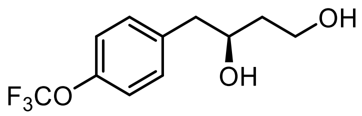

General procedure was used with methylenecyclopropane **1e** (25.7 mg, 0.12 mmol, 1.2 equiv.) and HBpin **2** (12.8 mg, 0.1 mmol, 1.0 equiv.) at rt for 36 h and B_2pin_2 **5** (30.5 mg, 0.12 mmol, 1.2 equiv.) at -25 °C for 52 h to afford **7e** as colorless oil (10.0 mg, 40% yield, 74% ee). $[\alpha]^{20}_{D}$= -6.8 (c 0.26, $CHCl_3$). ^1H NMR (400 MHz, Chloroform-*d*) δ 7.26-7.22 (m, 2H), 7.18-7.14 (m, 2H), 4.12-4.05 (m, 1H), 3.95-3.88 (m, 1H), 3.88-3.80 (m, 1H), 2.84-2.75 (m, 2H), 2.33 (s, 2H), 1.80-1.73 (m, 2H). ^{19}F NMR (377 MHz, Chloroform-*d*) δ -57.89. ^{13}C NMR (101 MHz, Chloroform-*d*) δ 147.9 (d, *J* = 2.0 Hz), 136.9, 130.7, 121.1, 120.5 (q, *J* = 256.7 Hz), 72.9, 61.8, 43.6, 37.7. The enantiomeric excess was determined by Daicel Chiralcel IA (0.46 cm x 25 cm), Hexanes /IPA = 95 /

05, 1.0 mL/min, λ = 214 nm, t (minor) = 18.3 min, t (major) = 19.7 min. HRMS (ESI-ion trap) m/z: [M+H]+ calcd for $C_{11}H_{14}F_3O_3$ 251.0890; found 251.0890.

(*S*)-4-(2-fluorophenyl)butane-1,3-diol (7f)

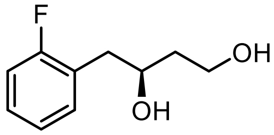

General procedure was used with methylenecyclopropane **1f** (17.8 mg, 0.12 mmol, 1.2 equiv.) and HBpin **2** (12.8 mg, 0.1 mmol, 1.0 equiv.) at rt for 36 h and B_2pin_2 **5** (30.5 mg, 0.12 mmol, 1.2 equiv.) at -25 °C for 52 h to afford **7f** as colorless oil (11.0 mg, 60% yield, 81% ee). $[\alpha]^{20}_D$= -12.3 (c 0.30, $CHCl_3$). ^{1}H NMR (400 MHz, Chloroform-*d*) δ 7.26-7.18 (m, 2H), 7.12-7.00 (m, 2H), 4.17-4.09 (m, 1H), 3.92-3.78 (m, 2H), 2.91-2.77 (m, 2H), 2.60 (s, 2H), 1.80-1.71 (m, 2H). ^{19}F NMR (377 MHz, Chloroform-*d*) δ -117.64.^{13}C NMR (101 MHz, Chloroform-*d*) δ 161.3 (d, *J* = 244.9 Hz), 131.9 (d, *J* = 4.8 Hz), 128.4 (d, *J* = 8.1 Hz), 125.1 (d, *J* = 15.8 Hz), 124.1 (d, *J* = 3.7 Hz), 115.4 (d, *J* = 22.4 Hz), 72.0, 61.7, 37.8, 37.5. The enantiomeric excess was determined by Daicel Chiralcel IC (0.46 cm x 25 cm), Hexanes /IPA = 93 / 07, 1.0 mL/min, λ = 210 nm, t (major) = 20.4 min, t (minor) = 25.6 min. HRMS (ESI-ion trap) m/z: [M+H]+ calcd for $C_{10}H_{14}FO_2$ 185.0972; found 185.0971.

(*S*)-4-(4-fluorophenyl)butane-1,3-diol (7g)

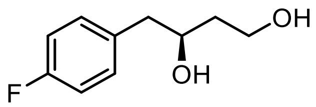

General procedure was used with methylenecyclopropane **1g** (17.8 mg, 0.12 mmol, 1.2 equiv.) and HBpin **2** (12.8 mg, 0.1 mmol, 1.0 equiv.) at rt for 36 h and B_2pin_2 **5** (30.5 mg, 0.12 mmol, 1.2 equiv.) at -25 °C for 48 h to afford **7g** as colorless oil (14.0 mg, 75% yield, 86% ee). $[\alpha]^{20}_D$= -2.8 (c 0.40, $CHCl_3$).^{1}H NMR (400 MHz, Chloroform-*d*) δ 7.23-7.12 (m, 2H), 7.04-6.93 (m, 2H), 4.10-4.01 (m, 1H), 3.93-3.76 (m, 2H), 2.83-2.69 (m, 2H), 2.20 (s, 2H), 1.80-1.67 (m, 2H). ^{19}F NMR (377 MHz, Chloroform-*d*) δ -116.58. ^{13}C NMR (101 MHz, Chloroform-*d*) δ 161.8 (d, *J* = 244.6 Hz), 133.8 (d, *J* =

3.3 Hz), 130.8 (d, *J* = 7.8 Hz), 115.4 (d, *J* = 21.2 Hz), 72.9, 61.7, 43.4, 37.7. The enantiomeric excess was determined by Daicel Chiralcel IC (0.46 cm x 25 cm), Hexanes /IPA = 92 / 08, 1.0 mL/min, λ = 210 nm, t (major) = 16.2 min, t (minor) = 18.5 min. HRMS (ESI-ion trap) m/z: [M+H]+ calcd for $C_{10}H_{13}FO_2Na$ 207.0792; found 207.0793.

(*S*)-4-(3-bromophenyl)butane-1,3-diol (7h)

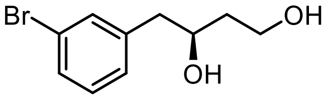

General procedure was used with methylenecyclopropane **1h** (24.8 mg, 0.12 mmol, 1.2 equiv.) and HBpin **2** (12.8 mg, 0.1 mmol, 1.0 equiv.) at rt for 36 h and B_2pin_2 **5** (30.5 mg, 0.12 mmol, 1.2 equiv.) at -25 °C for 48 h to afford **7h** as colorless oil (20.0 mg, 82% yield, 81% ee). $[\alpha]^{20}_{D}$= -11.3 (c 0.60, $CHCl_3$). ^{1}H NMR (400 MHz, Chloroform-*d*) δ 7.40-7.34 (m, 2H), 7.19-7.11 (m, 2H), 4.13-4.02 (m, 1H), 3.93-3.74 (m, 2H), 2.78-2.70 (m, 2H), 2.29 (s, 2H), 1.80-1.67 (m, 2H). ^{13}C NMR (101 MHz, Chloroform-*d*) δ 140.6, 132.4, 130.1, 129.7, 128.1, 122.6, 72.7, 61.6, 43.9, 37.7. The enantiomeric excess was determined by Daicel Chiralcel IC (0.46 cm x 25 cm), Hexanes /IPA = 93 / 07, 1.0 mL/min, λ = 210 nm, t (major) = 20.7 min, t (minor) = 25.6 min. HRMS (ESI-ion trap) m/z: [M+H]+ calcd for $C_{10}H_{14}BrO_2$ 245.0172; found 245.0169.

(*S*)-4-([1,1'-biphenyl]-4-yl)butane-1,3-diol (7i)

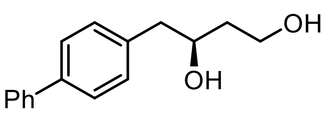

General procedure was used with methylenecyclopropane **1i** (24.7 mg, 0.12 mmol, 1.2 equiv.) and HBpin **2** (12.8 mg, 0.1 mmol, 1.0 equiv.) at rt for 36 h and B_2pin_2 **5** (30.5 mg, 0.12 mmol, 1.2 equiv.) at -25 °C for 52 h to afford **7i** as white solid (17.8 mg, 74% yield, 91% ee). m.p. 100.0-101.3 °C. $[\alpha]^{20}_{D}$= -13.9 (c 0.53, $CHCl_3$). ^{1}H NMR (400 MHz, Chloroform-*d*) δ 7.61-7.52 (m, 4H), 7.44 (dd, *J* = 8.4, 6.8 Hz, 2H), 7.37-7.32 (m, 1H), 7.31-7.27 (m, 2H), 4.17-4.09 (m, 1H), 3.95-3.80 (m, 2H), 2.90-2.77 (m, 2H), 2.39 (s, 2H), 1.87-1.71 (m, 2H). ^{13}C NMR (101 MHz, Chloroform-*d*) δ 140.8, 139.6, 137.1,

129.9, 128.8, 127.4, 127.2, 127.0, 73.1, 61.8, 44.0, 37.8. The enantiomeric excess was determined by Daicel Chiralcel ID (0.46 cm x 25 cm), Hexanes /IPA = 90 / 10, 1.0 mL/min, λ = 214 nm, t (major) = 16.1 min, t (minor) = 19.4 min. HRMS (ESI-ion trap) m/z: [M+H]+ calcd for $C_{16}H_{19}O_2$ 243.1380; found 243.1376.

(*S*)-4-(naphthalen-1-yl)butane-1,3-diol (7j)

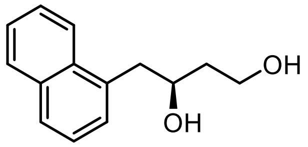

General procedure was used with methylenecyclopropane **1j** (21.6 mg, 0.12 mmol, 1.2 equiv.) and HBpin **2** (12.8 mg, 0.1 mmol, 1.0 equiv.) at rt for 36 h and B_2pin_2 **5** (30.5 mg, 0.12 mmol, 1.2 equiv.) at -25 °C for 48 h to afford **7j** as white solid (16.0 mg, 74% yield, 82% ee). m.p. 77.7-78.7 °C. $[\alpha]^{20}_D$= -24.6 (c 0.47, $CHCl_3$).^{1}H NMR (400 MHz, Chloroform-*d*) δ 8.07-8.02 (m, 1H), 7.89-7.84 (m, 1H), 7.76 (d, *J* = 7.9 Hz, 1H), 7.56-7.46 (m, 2H), 7.45-7.39 (m, 1H), 7.38-7.33 (m, 1H), 4.29-4.17 (m, 1H), 3.93-3.74 (m, 2H), 3.34-3.15 (m, 2H), 2.36 (s, 2H), 1.90-1.76 (m, 2H). ^{13}C NMR (101 MHz, Chloroform-*d*) δ 134.1, 134.0, 132.1, 128.9, 127.8, 127.5, 126.1, 125.8, 125.5, 123.8, 72.4, 61.8, 41.5, 38.1. The enantiomeric excess was determined by Daicel Chiralcel IC (0.46 cm x 25 cm), Hexanes /IPA = 92 / 08, 1.0 mL/min, λ = 210 nm, t (minor) = 22.3 min, t (major) = 25.6 min. HRMS (ESI-ion trap) m/z: [M+H]+ calcd for $C_{14}H_{17}O_2$ 217.1223; found 217.1220.

(*S*)-4-(naphthalen-2-yl)butane-1,3-diol (7k)

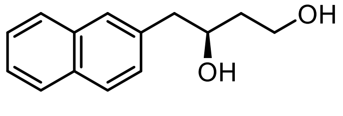

General procedure was used with methylenecyclopropane **1k** (21.6 mg, 0.12 mmol, 1.2 equiv.) and HBpin **2** (12.8 mg, 0.1 mmol, 1.0 equiv.) at rt for 36 h and B_2pin_2 **5** (30.5 mg, 0.12 mmol, 1.2 equiv.) at -25 °C for 48 h to afford **7k** as white solid (16.4 mg, 76% yield, 84% ee). m.p. 63.0-64.0 °C. $[\alpha]^{20}_D$= -6.3 (c 0.48, $CHCl_3$).^{1}H NMR (400 MHz, Chloroform-*d*) δ 7.89-7.74 (m, 3H), 7.66 (s, 1H), 7.51-7.41 (m, 2H), 7.35 (dd, *J* = 8.4,

1.8 Hz, 1H), 4.22-4.11 (m, 1H), 3.94-3.76 (m, 2H), 3.01-2.86 (m, 2H), 2.62 (s, 2H), 1.84-1.71 (m, 2H). ^{13}C NMR (101 MHz, Chloroform-*d*) δ 135.6, 133.6, 132.3, 128.3, 128.0, 127.7, 127.7, 127.5, 126.2, 125.6, 72.9, 61.7, 44.5, 37.8. The enantiomeric excess was determined by Daicel Chiralcel IC (0.46 cm x 25 cm), Hexanes /IPA = 92 / 08, 1.0 mL/min, λ = 210 nm, t (major) = 27.1 min, t (minor) = 29.8 min. HRMS (ESI-ion trap) m/z: [M+H]+ calcd for $C_{14}H_{16}O_2Na$ 239.1043; found 239.1040.

(*S*)-4-(benzofuran-3-yl)butane-1,3-diol (7l)

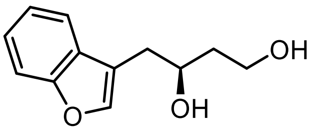

General procedure was used with methylenecyclopropane **1l** (20.4 mg, 0.12 mmol, 1.2 equiv.) and HBpin **2** (12.8 mg, 0.1 mmol, 1.0 equiv.) at rt for 36 h and B_2pin_2 **5** (30.5 mg, 0.12 mmol, 1.2 equiv.) at -25 °C for 52 h to afford **7l** as colorless oil (9.0 mg, 44% yield, 68% ee). $[\alpha]^{20}_{D}$= -6.9 (c 0.23, $CHCl_3$).^{1}H NMR (400 MHz, Chloroform-*d*) δ 7.60-7.56 (m, 1H), 7.52 (s, 1H), 7.51-7.46 (m, 1H), 7.34-7.28 (m, 1H), 7.27-7.23 (m, 1H), 4.26-4.16 (m, 1H), 3.94-3.81 (m, 2H), 2.91-2.82 (m, 2H), 2.23 (s, 2H), 1.86-1.74 (m, 2H). ^{13}C NMR (101 MHz, Chloroform-*d*) δ 155.4, 142.6, 128.1, 124.5, 122.6, 119.7, 116.4, 111.6, 71.2, 61.7, 38.0, 32.1. The enantiomeric excess was determined by Daicel Chiralcel IA (0.46 cm x 25 cm), Hexanes /IPA = 95 / 05, 1.0 mL/min, λ = 214 nm, t (major) = 29.6 min, t (minor) = 32.6 min. HRMS (ESI-ion trap) m/z: [M+Na]+ calcd for $C_{12}H_{14}O_3Na$ 229.0835; found 229.0830.

(*S*)-4-(3,4-difluorophenyl)butane-1,3-diol (7m)

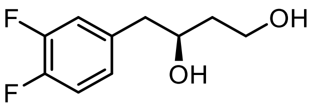

General procedure was used with methylenecyclopropane **1m** (19.9 mg, 0.12 mmol, 1.2 equiv.) and HBpin **2** (12.8 mg, 0.1 mmol, 1.0 equiv.) at rt for 36 h and B_2pin_2 **5** (30.5 mg, 0.12 mmol, 1.2 equiv.) at -25 °C for 48 h to afford **7m** as colorless oil (14.5 mg, 72% yield, 79% ee). $[\alpha]^{20}_{D}$= -9.8 (c 0.42, $CHCl_3$).^{1}H NMR (400 MHz, Chloroform-

d) δ 7.14-7.00 (m, 2H), 6.95-6.87 (m, 1H), 4.10-4.00 (m, 1H), 3.94-3.77 (m, 2H), 2.77-2.68 (m, 2H), 2.43 (s, 2H), 1.78-1.64 (m, 2H). ^{19}F NMR (377 MHz, Chloroform-*d*) δ -137.93 (d, *J* = 21.4 Hz), -141.24 (d, *J* = 21.2 Hz).^{13}C NMR (101 MHz, Chloroform-*d*) δ 150.9 (dd, *J* = 99.1, 12.5 Hz), 148.5 (dd, *J* = 97.7, 12.6 Hz), 135.2 (dd, *J* = 5.6, 3.9 Hz), 125.3 (dd, *J* = 6.1, 3.5 Hz), 118.2 (d, *J* = 16.8 Hz), 117.2 (d, *J* = 16.9 Hz), 72.7, 61.6, 43.3, 37.6. The enantiomeric excess was determined by Daicel Chiralcel ID (0.46 cm x 25 cm), Hexanes /IPA = 95 / 05, 1.0 mL/min, λ = 214 nm, t (major) = 17.7min, t (minor) = 24.9 min. HRMS (ESI-ion trap) m/z: [M+H]+ calcd for $C_{10}H_{13}F_2O_2$ 203.0878; found 203.0877.

(*S*)-4-(3,4,5-trimethoxyphenyl)butane-1,3-diol (7n)

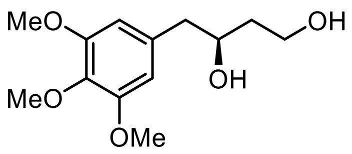

General procedure was used with methylenecyclopropane **1n** (26.4 mg, 0.12 mmol, 1.2 equiv.) and HBpin **2** (12.8 mg, 0.1 mmol, 1.0 equiv.) at rt for 36 h and B_2pin_2 **5** (30.5 mg, 0.12 mmol, 1.2 equiv.) at -25 °C for 52 h to afford **7n** as colorless oil (16.0 mg, 63% yield, 79% ee). $[\alpha]^{20}_D$= -6.0 (c 0.47, $CHCl_3$). ^{1}H NMR (400 MHz, Chloroform-*d*) δ 6.43 (s, 2H), 4.11-4.04 (m, 1H), 3.92-3.79 (m, 11H), 2.79-2.65 (m, 2H), 2.27 (s, 2H), 1.83-1.72 (m, 2H). ^{13}C NMR (101 MHz, Chloroform-*d*) δ 153.3, 136.6, 133.8, 106.2, 72.9, 61.7, 60.9, 56.1, 44.7, 37.9. The enantiomeric excess was determined by Daicel Chiralcel ID (0.46 cm x 25 cm), Hexanes /IPA = 75 / 25, 1.0 mL/min, λ = 214 nm, t (minor) = 26.3 min, t (major) = 28.6 min. HRMS (ESI-ion trap) m/z: [M+Na]+ calcd for $C_{13}H_{20}O_5Na$ 279.1203; found 279.1199.

(*S*)-4-hydroxy-4-phenylbutyl diphenylphosphinate (8)

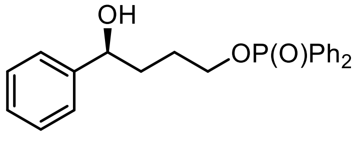

Colorless oil (28.5 mg, 78% yield, 91% ee). ^{1}H NMR (400 MHz, Chloroform-*d*) δ 7.82-7.73 (m, 4H), 7.54-7.47 (m, 2H), 7.47-7.40 (m, 4H), 7.34-7.30 (m, 4H), 7.27-7.23 (m, 1H), 4.74-4.66 (m, 1H), 4.09-4.00 (m, 2H), 2.36 (s, 1H), 1.97-1.76 (m, 4H). ^{31}P NMR (162 MHz, Chloroform-*d*) δ 31.75. ^{13}C NMR (101 MHz, Chloroform-*d*) δ 144.6, 132.2 (d, *J* = 2.8 Hz), 131.6 (d, *J* = 10.2 Hz), 131.4 (d, *J* = 137.2 Hz), 128.6 (d, *J* = 13.2 Hz), 128.5, 127.5, 125.9, 73.9, 65.0 (d, *J* = 6.0 Hz), 34.9, 27.0 (d, *J* = 6.2 Hz). The enantiomeric excess was determined by Daicel Chiralcel IC (0.46 cm x 25 cm), Hexanes /IPA = 60 / 40, 1.0 mL/min, λ = 220 nm, t (major) = 9.6 min, t (minor) = 12.2 min. HRMS (ESI-ion trap) m/z: [M+H]+ calcd for $C_{22}H_{24}O_3P$ 367.1458; found 367.1450.

(*S*)-1-phenylbutane-1,4-diyl bis(diphenylphosphinate) (9)

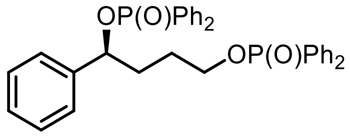

Colorless oil (63.0 mg, 78% yield, 90% ee). ^{1}H NMR (600 MHz, Chloroform-*d*) δ 7.83-7.78 (m, 2H), 7.77-7.71 (m, 4H), 7.58-7.52 (m, 2H), 7.51-7.46 (m, 3H), 7.44-7.35 (m, 7H), 7.25-7.21 (m, 5H), 7.20-7.16 (m, 2H), 5.36 (dt, *J* = 9.6, 6.6 Hz, 1H), 4.01-3.90 (m, 2H), 2.19-2.13 (m, 1H), 2.07-2.01 (m, 1H), 1.83-1.73 (m, 1H), 1.73-1.62 (m, 1H).^{31}P NMR (243 MHz, Chloroform-*d*) δ 31.41, 31.36. ^{13}C NMR (151 MHz, Chloroform-*d*) δ 140.2 (d, *J* = 3.5 Hz), 132.7, 132.1 (d, *J* = 2.7 Hz), 131.9, 131.7 (dd, *J* = 76.7, 10.3 Hz), 131.6 (d, *J* = 10.3 Hz), 131.3 (d, *J* = 143.1 Hz), 131.0 (d, *J* = 3.3 Hz), 128.5 (d, *J* = 13.1 Hz), 128.4, 128.3 (dd, *J* = 49.5, 13.1 Hz), 128.1, 126.5, 77.7 (d, *J* = 5.9 Hz), 64.4 (d, *J* = 5.8 Hz), 34.5 (d, *J* = 4.1 Hz), 26.3 (d, *J* = 6.7 Hz).The enantiomeric excess was determined by Daicel Chiralcel IA (0.46 cm x 25 cm), Hexanes /IPA = 60 / 40, 1.0 mL/min, λ = 210 nm, t (minor) = 12.7 min, t (major) = 14.3 min. HRMS (ESI-ion trap) m/z: [M+H]+ calcd for $C_{34}H_{33}O_4P_2$ 567.1849; found 567.1838.

(*S*)-4-(4-methoxyphenyl)-1-phenylbutan-1-ol (10)

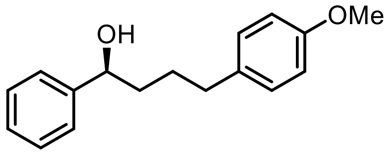

Colorless oil (26.6 mg, 52% yield, 96% ee). ^{1}H NMR (400 MHz, Chloroform-*d*) δ 7.38-7.31 (m, 4H), 7.29-7.25 (m, 1H), 7.10-7.04 (m, 2H), 6.84-6.78 (m, 2H), 4.71-4.65 (m, 1H), 3.78 (s, 3H), 2.58 (t, *J* = 7.3 Hz, 2H), 1.85-1.69 (m, 4H). ^{13}C NMR (101 MHz, Chloroform-*d*) δ 157.7, 144.8, 134.4, 129.3, 128.5, 127.6, 125.9, 113.7, 74.6, 55.3, 38.6, 34.8, 27.8. The enantiomeric excess was determined by Daicel Chiralcel ID (0.46 cm x 25 cm), Hexanes /IPA = 98 / 02, 1.0 mL/min, λ = 210 nm, t (major) = 32.5 min, t (minor) = 36.1 min. HRMS (ESI-ion trap) m/z: [M+Na]+ calcd for $C_{17}H_{20}O_2Na$ 279.1356; found 279.1352.

(*S*)-4-(3,5-dimethylphenyl)-1-phenylbutan-1-ol (11)

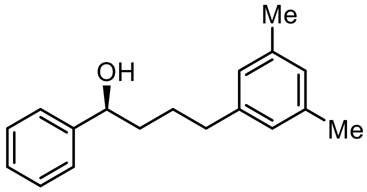

White solid, (28.4 mg, 56% yield, 96% ee). m.p. 35.0-36.2 °C. ^{1}H NMR (400 MHz, Chloroform-*d*) δ 7.38-7.28 (m, 4H), 7.31-7.22 (m, 1H), 6.83-6.73 (m, 3H), 4.67 (dd, *J* = 7.4, 5.3 Hz, 1H), 2.54 (t, *J* = 7.4 Hz, 2H), 2.27 (s, 6H), 1.90-1.66 (m, 4H). ^{13}C NMR (101 MHz, Chloroform-*d*) δ 144.8, 142.2, 137.8, 128.5, 127.6, 127.4, 126.3, 126.0, 74.6, 38.7, 35.6, 27.7, 21.3. The enantiomeric excess was determined by Daicel Chiralcel ID (0.46 cm x 25 cm), Hexanes /IPA = 99 / 01, 1.0 mL/min, λ = 210 nm, t (major) = 17.2 min, t (minor) = 19.5 min. HRMS (ESI-ion trap) m/z: [M+Na]+ calcd for $C_{18}H_{22}ONa$ 277.1563; found 277.1558.

(*S*)-4-phenylbutane-1,3-diyl bis(diphenylphosphinate) (12)

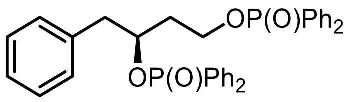

Colorless oil (36.0 mg, 65% yield, 90% ee). ^{1}H NMR (600 MHz, Chloroform-*d*) δ 7.79-7.70 (m, 6H), 7.61-7.56 (m, 2H), 7.54-7.44 (m, 4H), 7.45-7.32 (m, 8H), 7.27-7.21 (m,

3H), 7.10-7.06 (m, 2H), 4.80-4.72 (m, 1H), 4.24-4.15 (m, 1H), 4.15-4.08 (m, 1H), 3.10-2.95 (m, 2H), 2.22-2.07 (m, 2H). ^{31}P NMR (243 MHz, Chloroform-*d*) δ 31.88, 30.75. ^{13}C NMR (151 MHz, Chloroform-*d*) δ 136.8, 132.2, 132.2 (t, *J* = 2.5 Hz), 132.0 (dd, *J* = 14.8, 2.8 Hz), 131.7 (d, *J* = 10.3 Hz), 131.6 (dd, *J* = 10.2, 5.0 Hz), 131.4 (dd, *J* = 136.5, 8.8 Hz), 131.0 (d, *J* = 91.5 Hz), 129.7, 128.6 (dd, *J* = 12.8, 2.9 Hz), 128.5, 128.4 (d, *J* = 9.0 Hz), 126.7, 74.8 (d, *J* = 6.5 Hz), 61.2 (d, *J* = 5.8 Hz), 42.0 (d, *J* = 4.0 Hz), 35.8 (dd, *J* = 6.4, 3.8 Hz). The enantiomeric excess was determined by Daicel Chiralcel IA (0.46 cm x 25 cm), Hexanes /IPA = 80 / 20, 0.8 mL/min, λ = 210 nm, t (minor) = 40.4 min, t (major) = 43.3 min. HRMS (ESI-ion trap) m/z: [M+H]+ calcd for $C_{34}H_{33}O_4P_2$ 567.1849; found 567.1839.

(*R*)-4-(3,5-dimethylphenyl)-1-phenylbutan-2-ol (13)

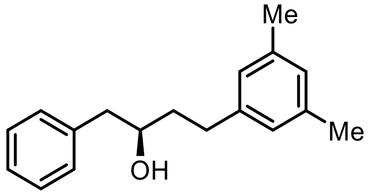

Colorless oil (17.0 mg, 67% yield, 89% ee). ^{1}H NMR (400 MHz, Chloroform-*d*) δ 7.34-7.28 (m, 2H), 7.25-7.19 (m, 3H), 6.82 (s, 3H), 3.88-3.80 (m, 1H), 2.85 (dd, *J* = 13.6, 4.3 Hz, 1H), 2.81-2.73 (m, 1H), 2.72-2.59 (m, 2H), 2.28 (d, *J* = 0.7 Hz, 6H), 1.91-1.71 (m, 2H). ^{13}C NMR (101 MHz, Chloroform-*d*) δ 142.0 138.5, 137.9, 129.5, 128.6, 127.5, 126.5, 126.3, 72.1, 44.2, 38.6, 32.0, 21.3.

The enantiomeric excess was determined by Daicel Chiralcel IC (0.46 cm x 25 cm), Hexanes /IPA = 99 / 01, 1.0 mL/min, λ = 214 nm, t (minor) = 18.9 min, t (major) = 21.4 min. HRMS (ESI-ion trap) m/z: [M+H]+ calcd for [M+Na]+ calcd for $C_{18}H_{22}ONa$ 277.1563; found 277.1555.

10. NMR Spectrum

(S)-2,2'-(1-phenylbutane-1,4-diyl)bis(4,4,5,5-tetramethyl-1,3,2-dioxaborolane) (3a)

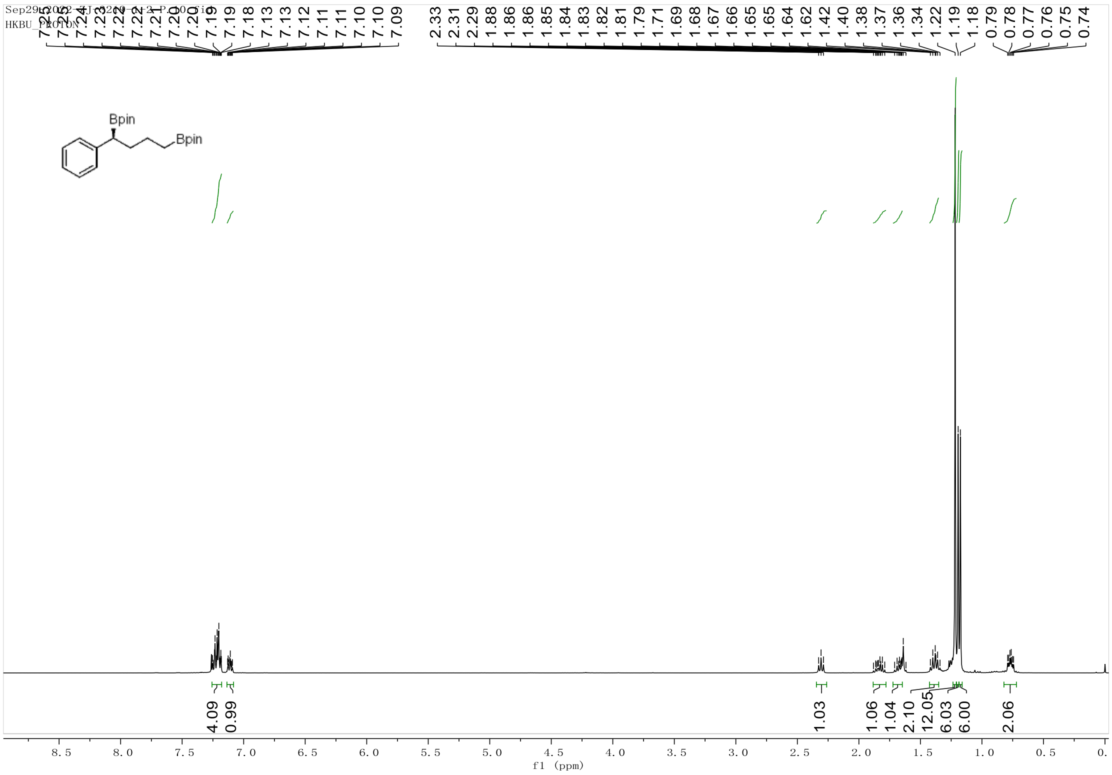

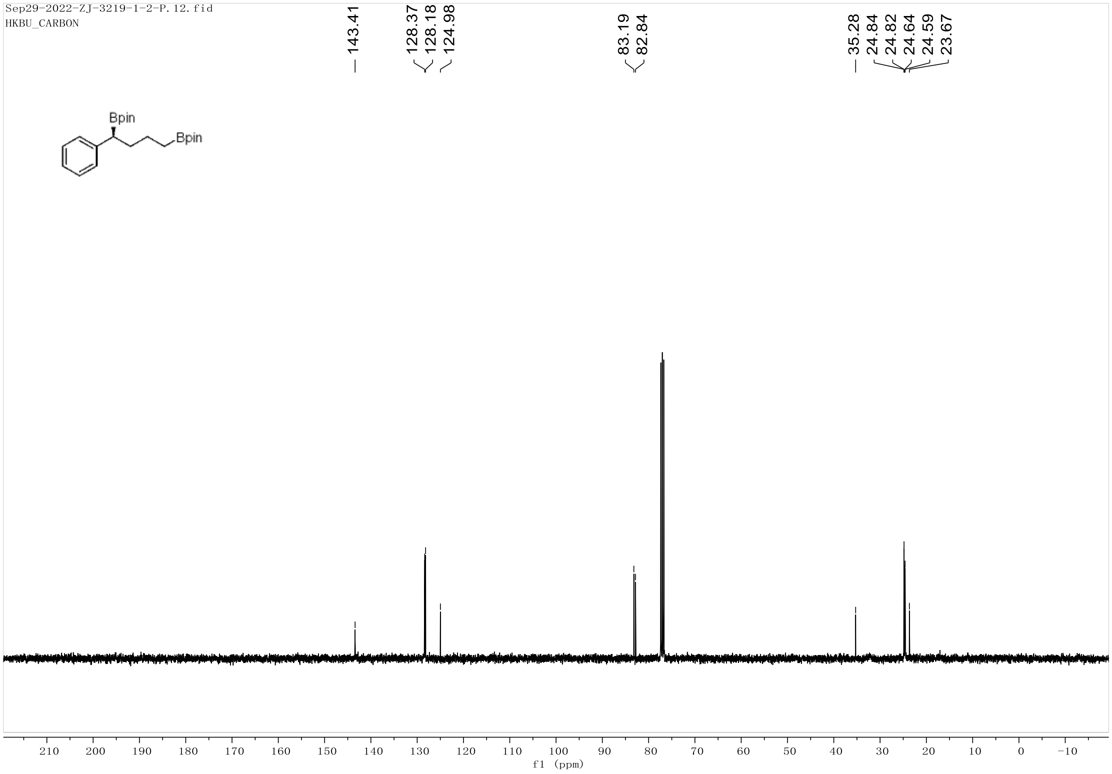

(*S*)-1-phenylbutane-1,4-diol (4a)

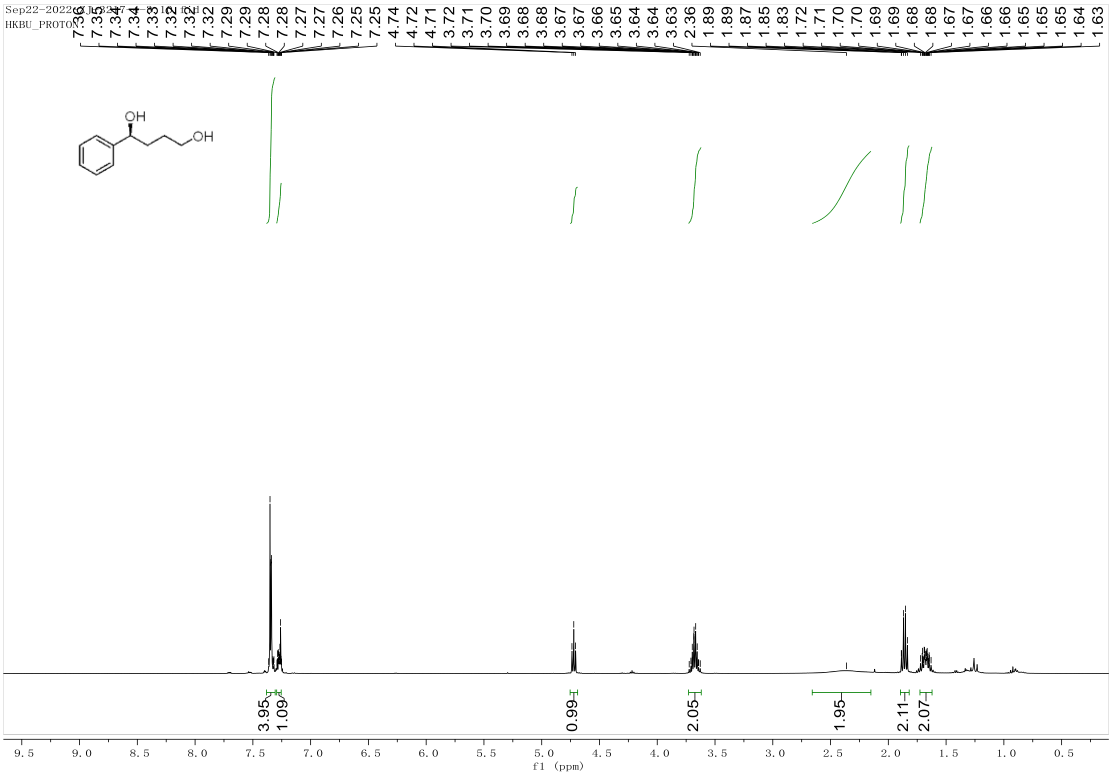

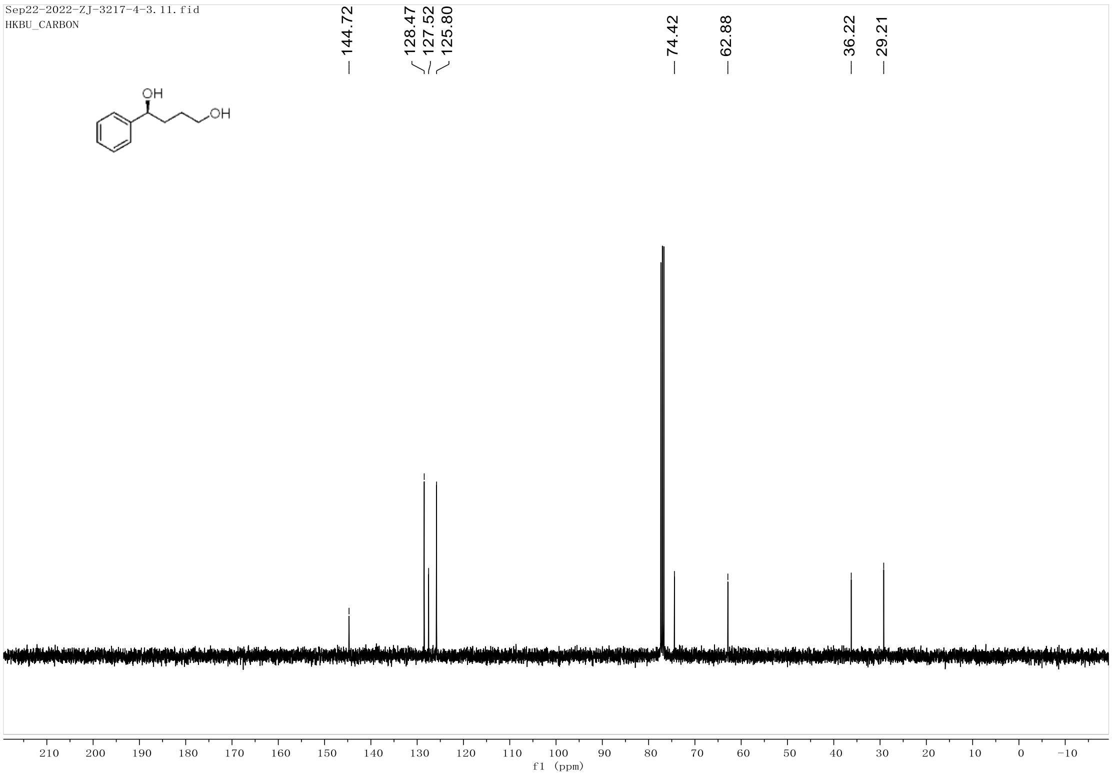

(*S*)-1-(o-tolyl)butane-1,4-diol (4b)

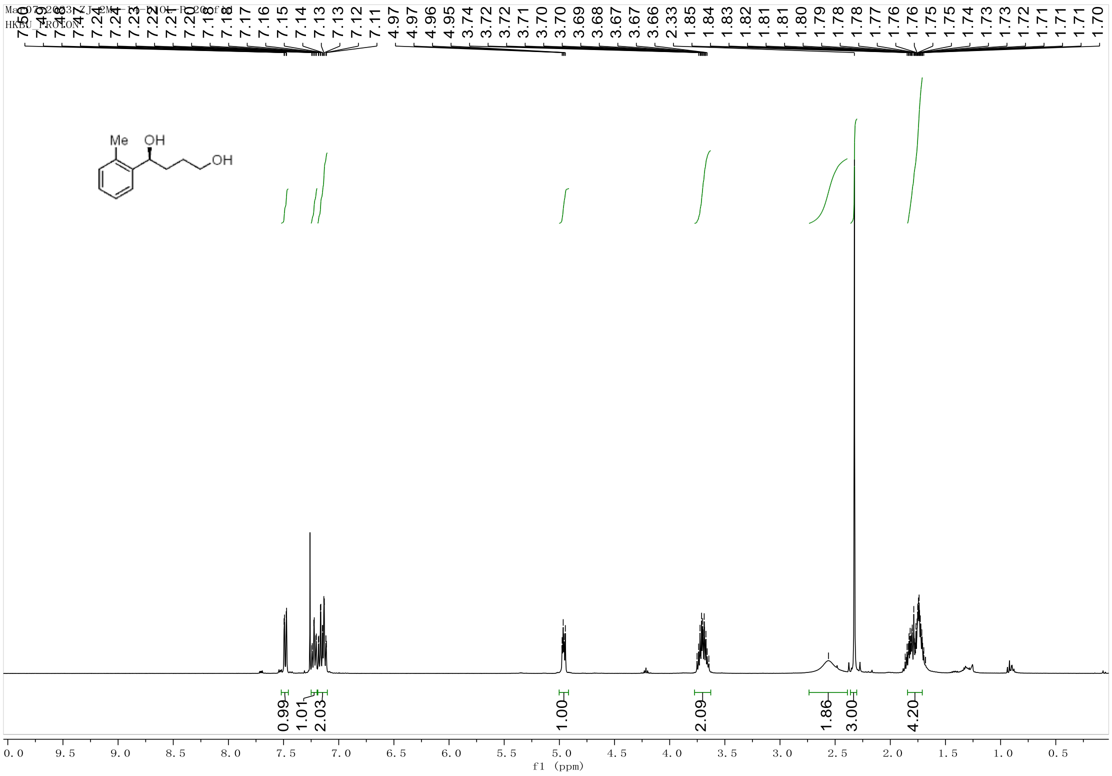

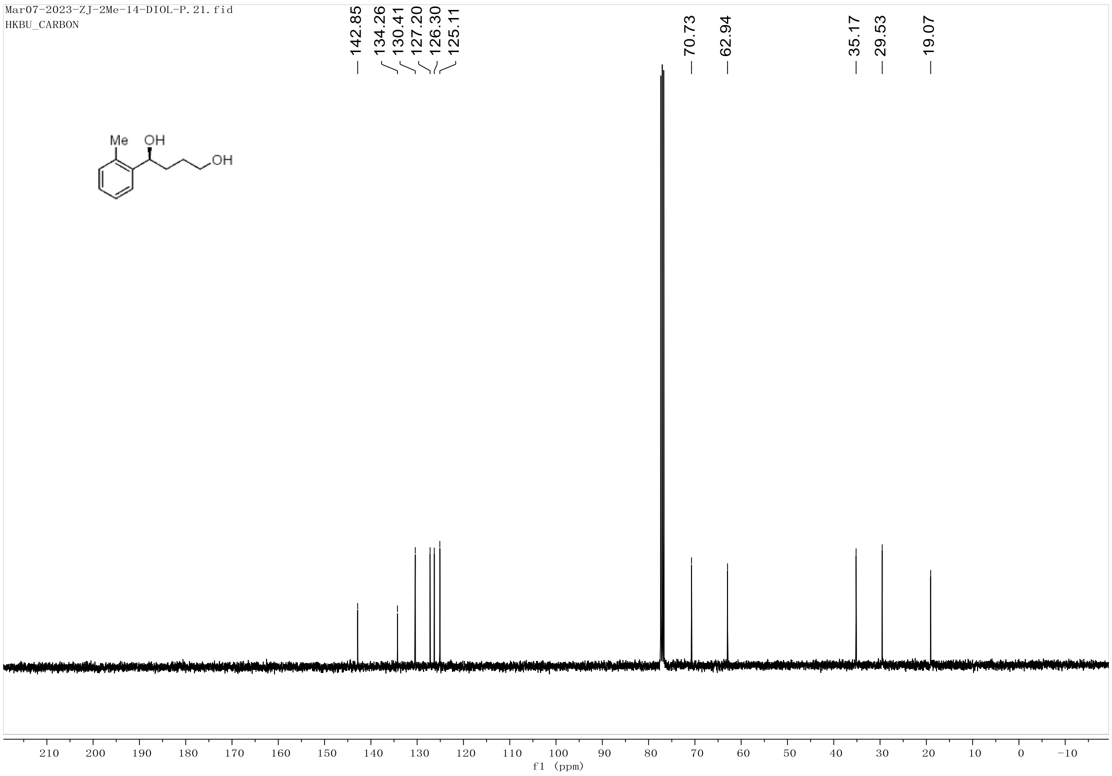

(*S*)-1-(m-tolyl)butane-1,4-diol (4c)

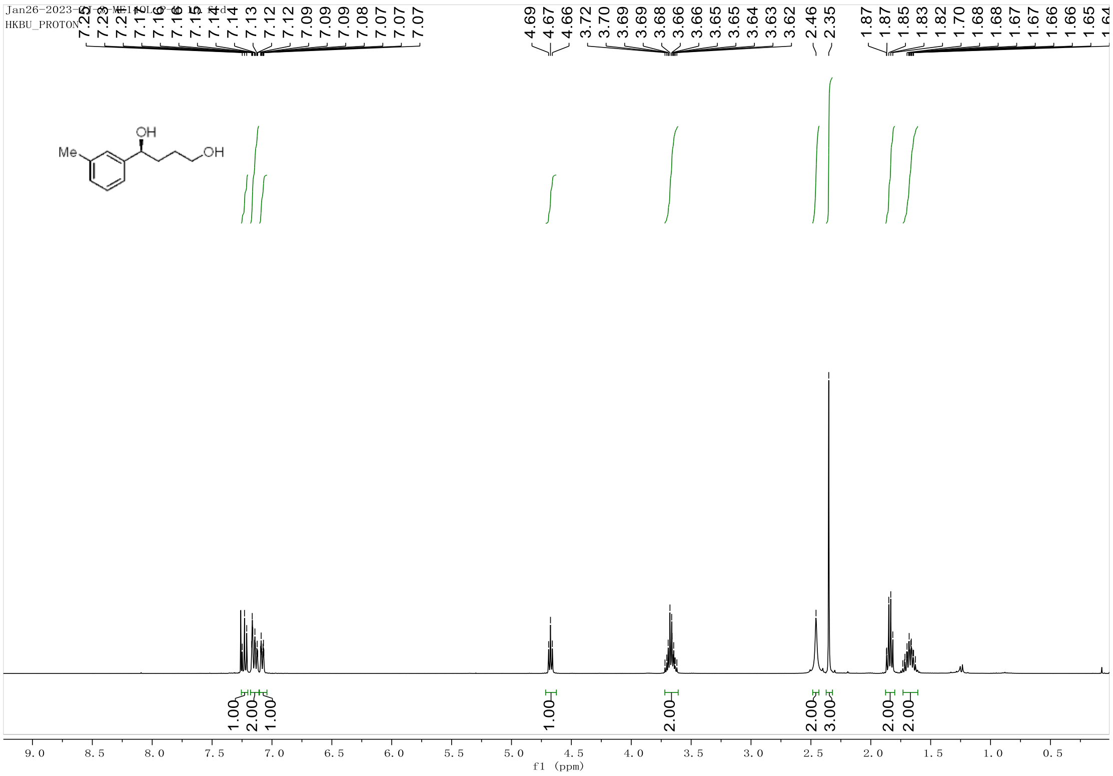

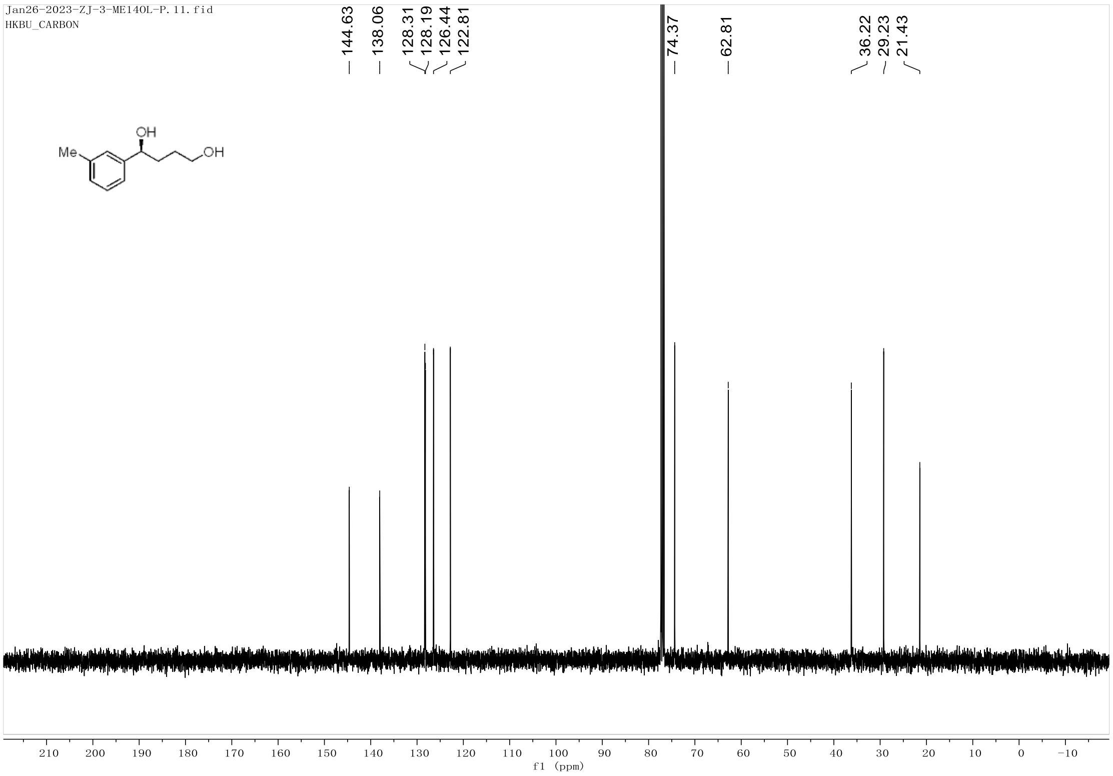

(*S*)-1-(p-tolyl)butane-1,4-diol (4d)

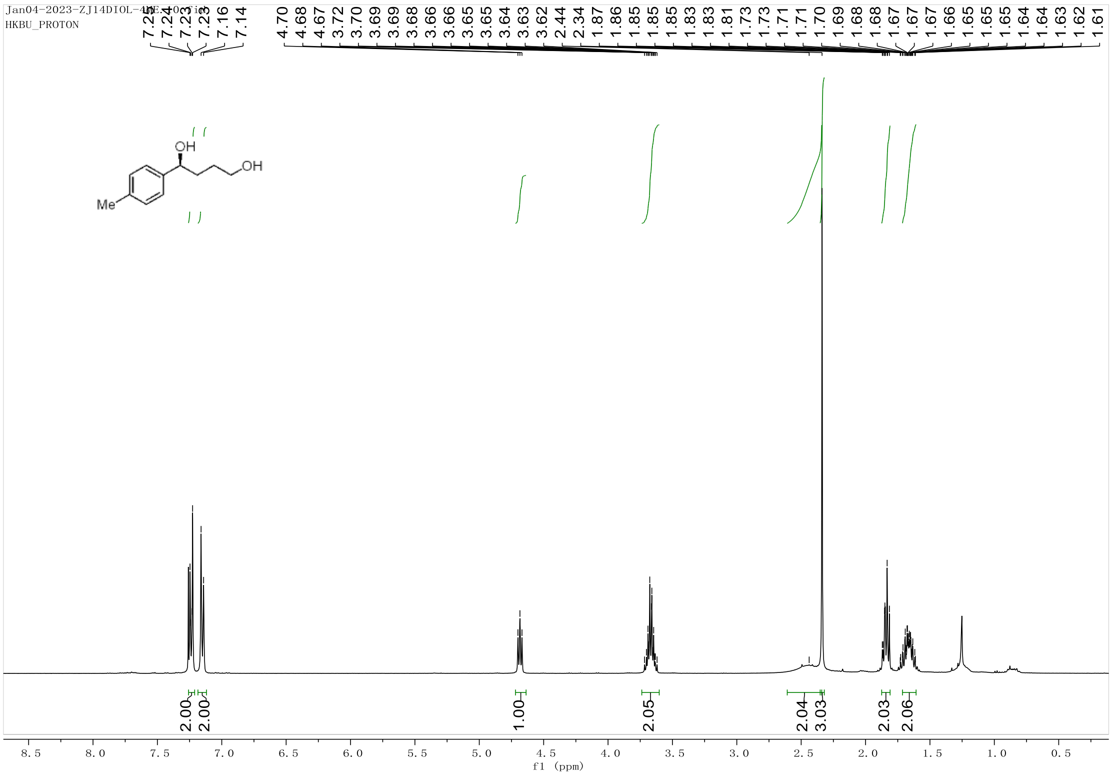

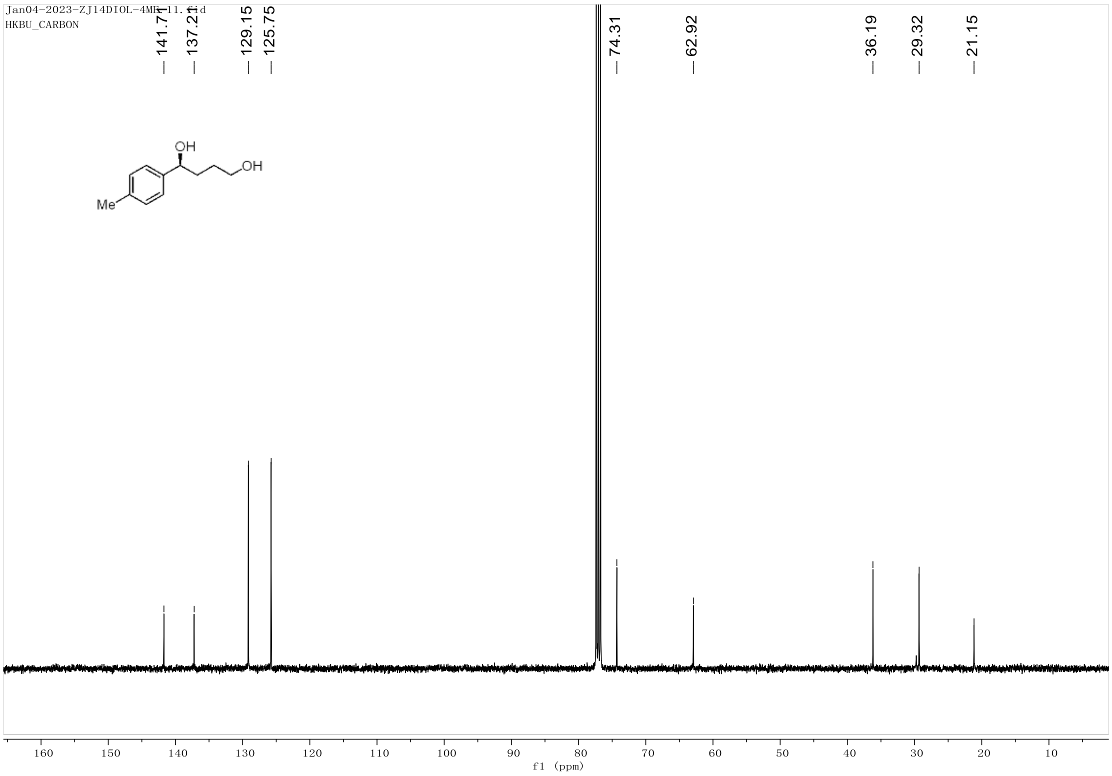

(*S*)-1-(4-(tert-butyl)phenyl)butane-1,4-diol (4e)

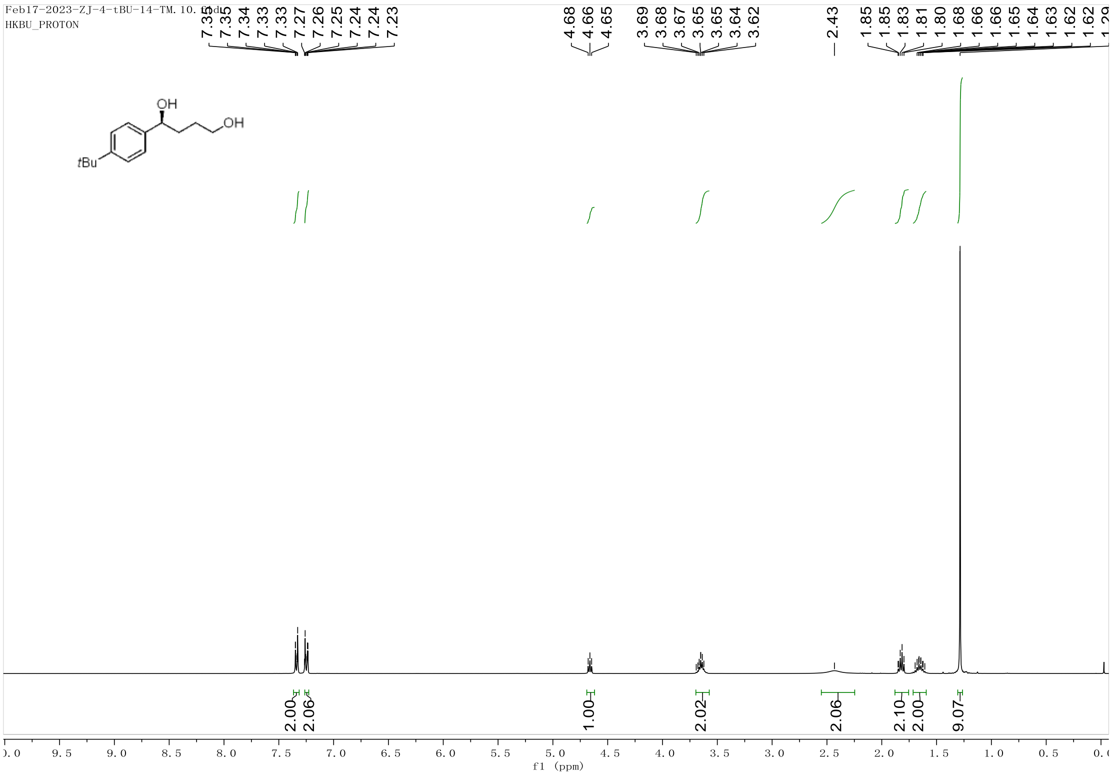

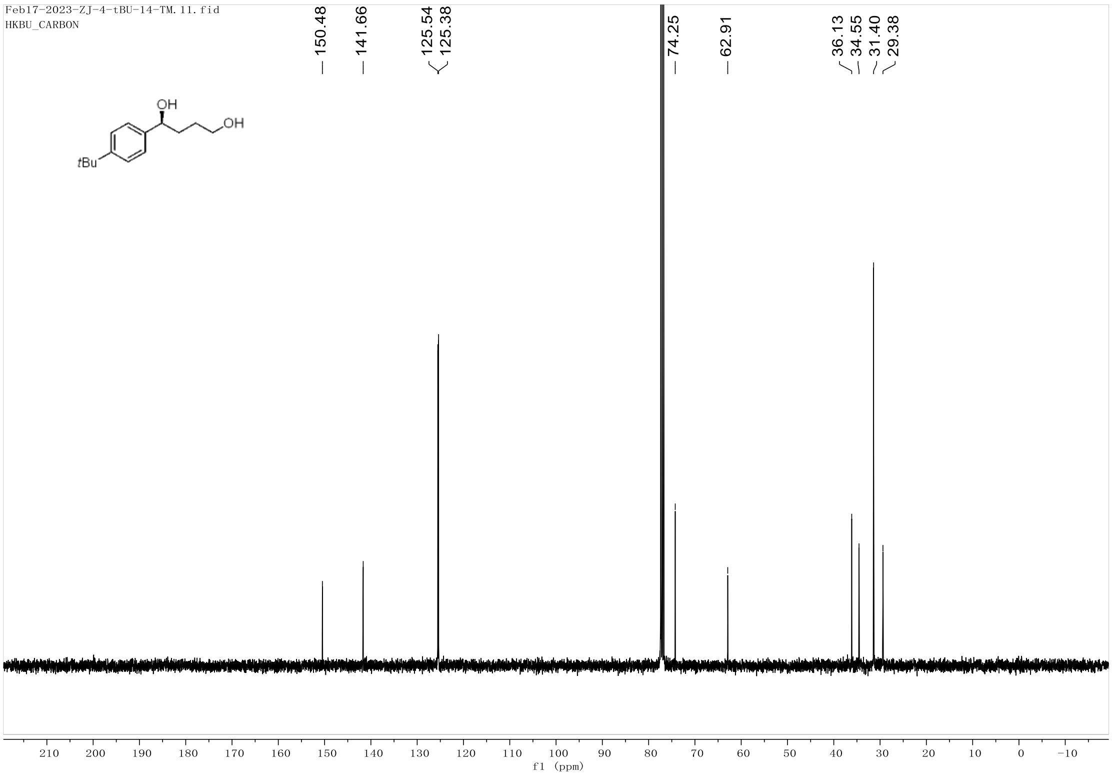

(*S*)-1-(3-methoxyphenyl)butane-1,4-diol (4f)

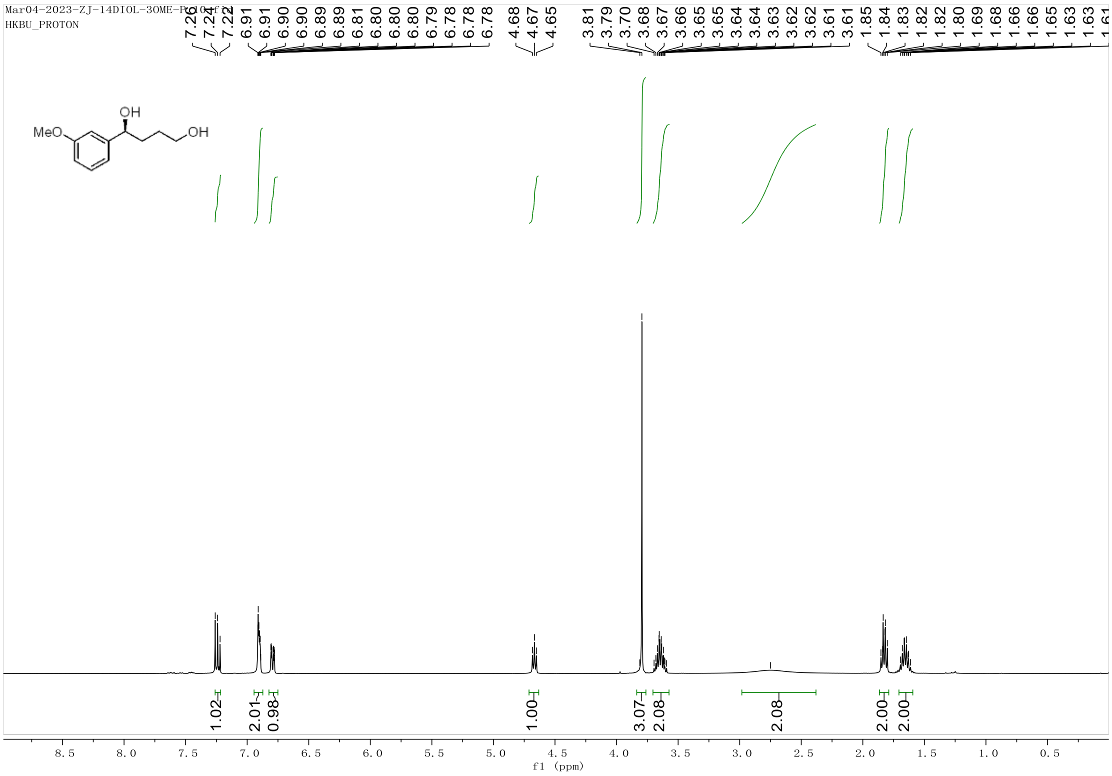

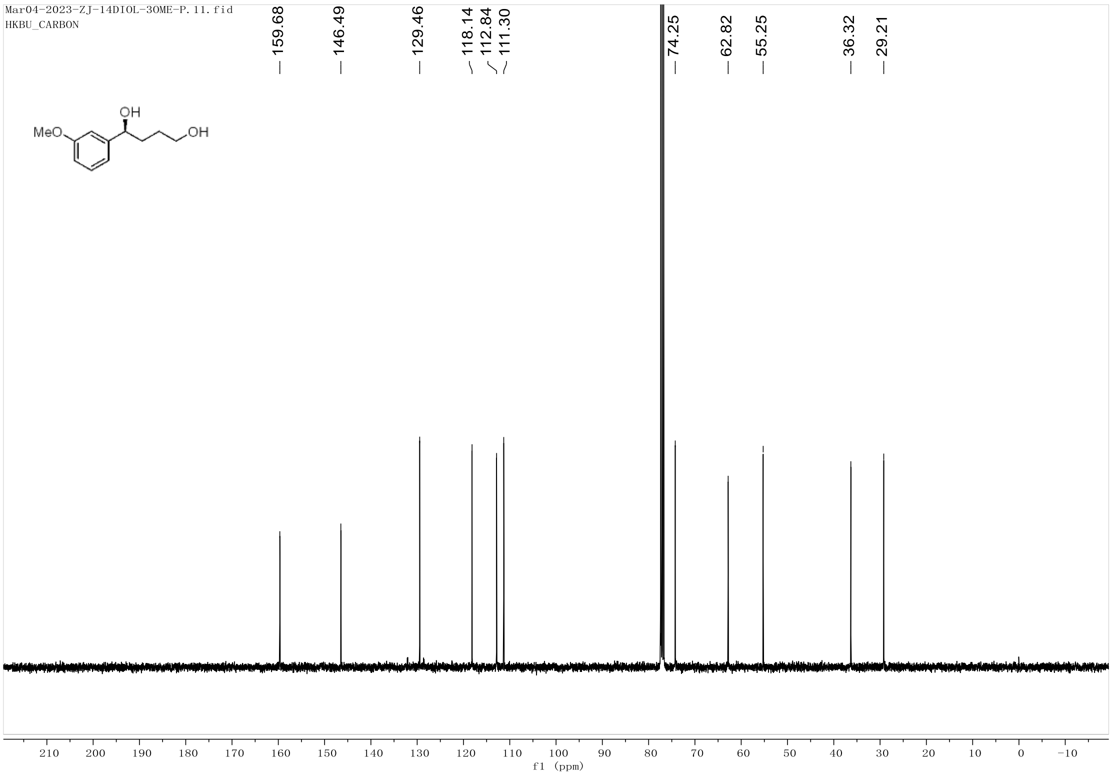

(*S*)-1-(4-methoxyphenyl)butane-1,4-diol (4g)

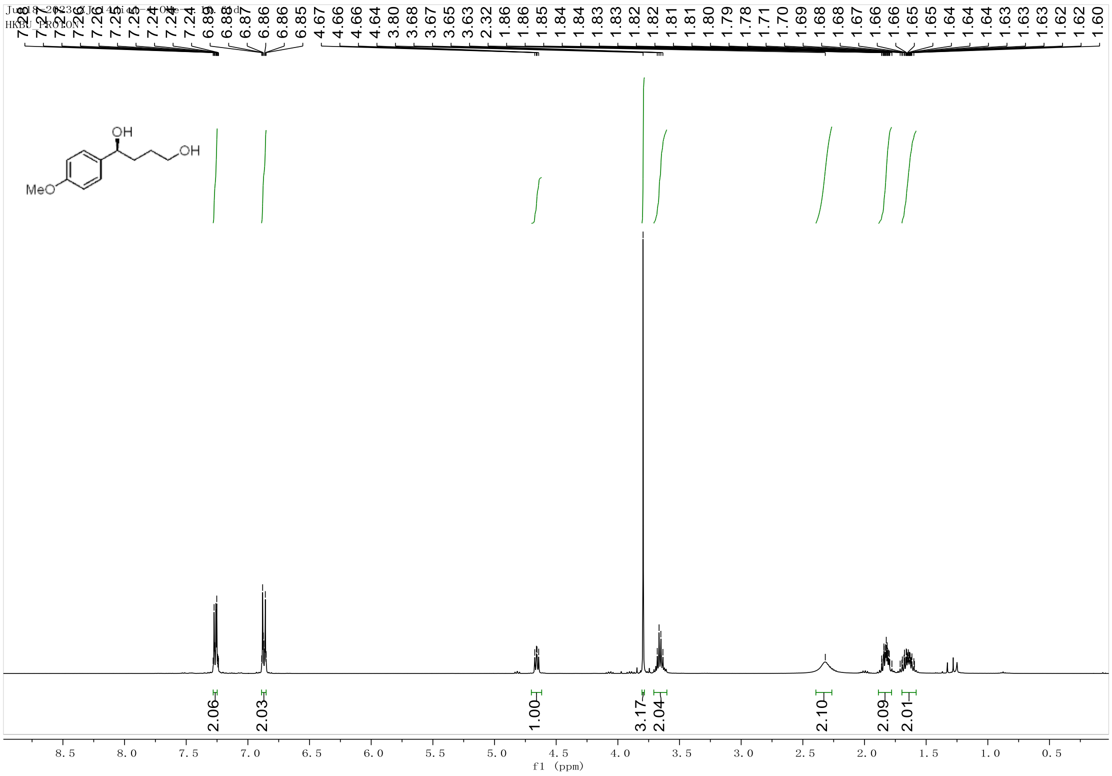

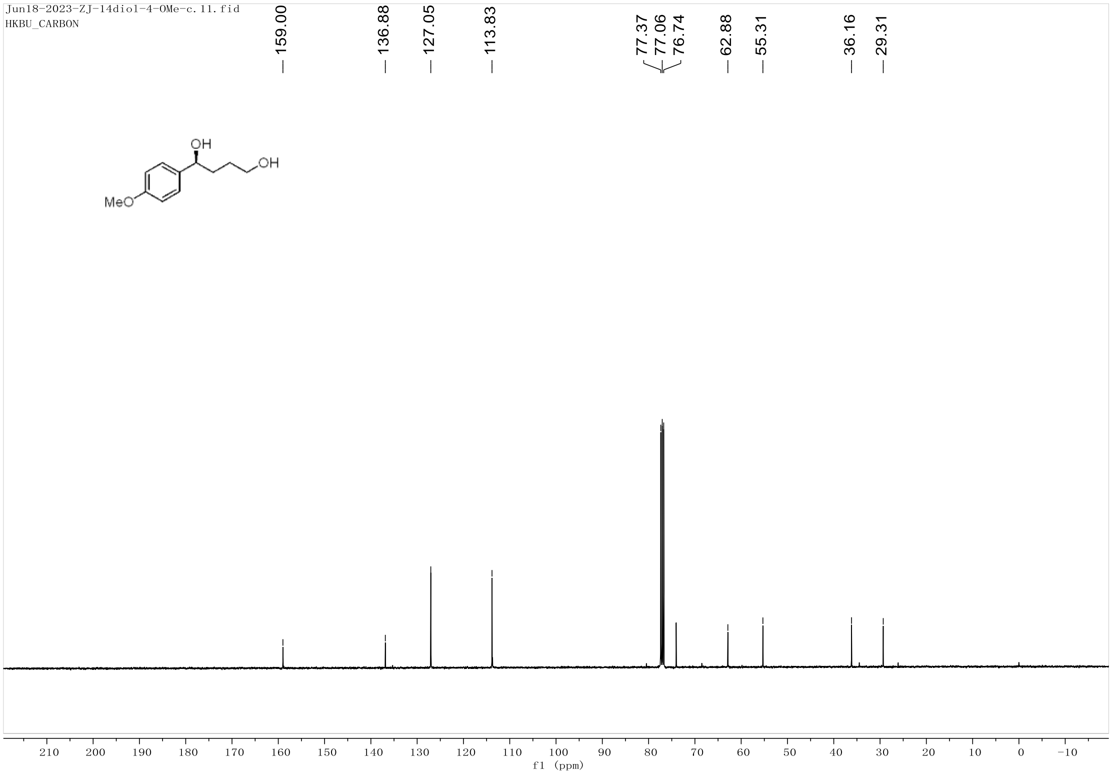

(*S*)-1-(4-(trifluoromethoxy)phenyl)butane-1,4-diol (4h)

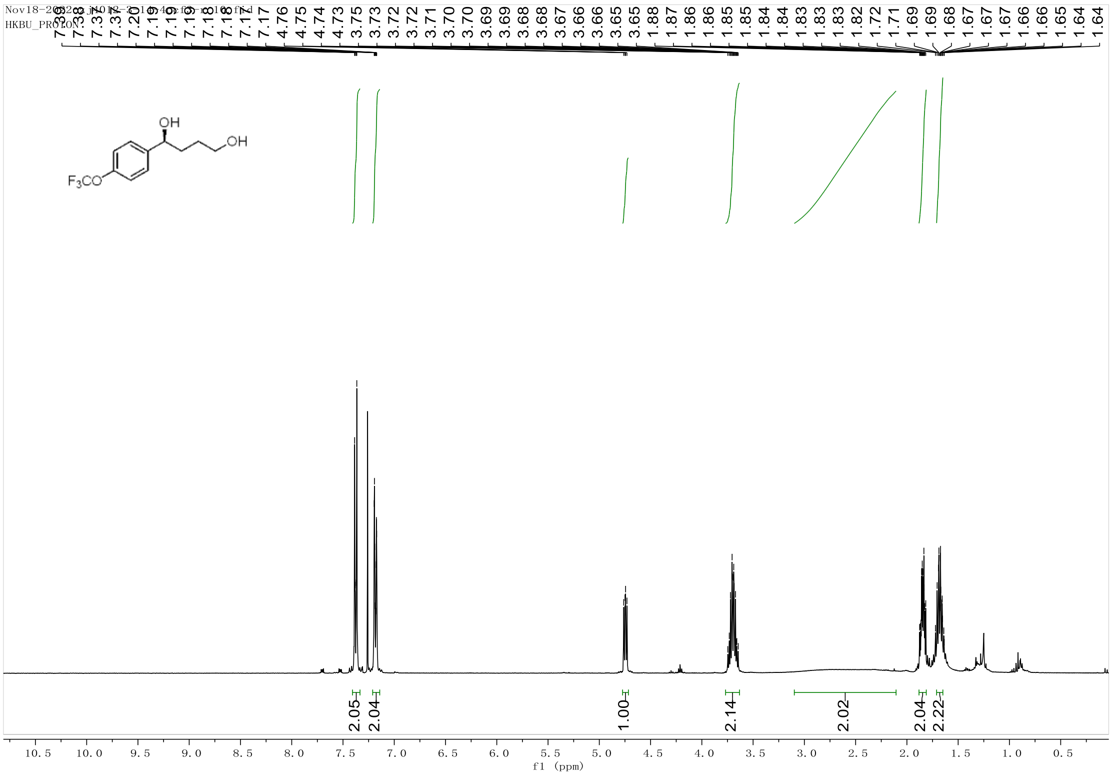

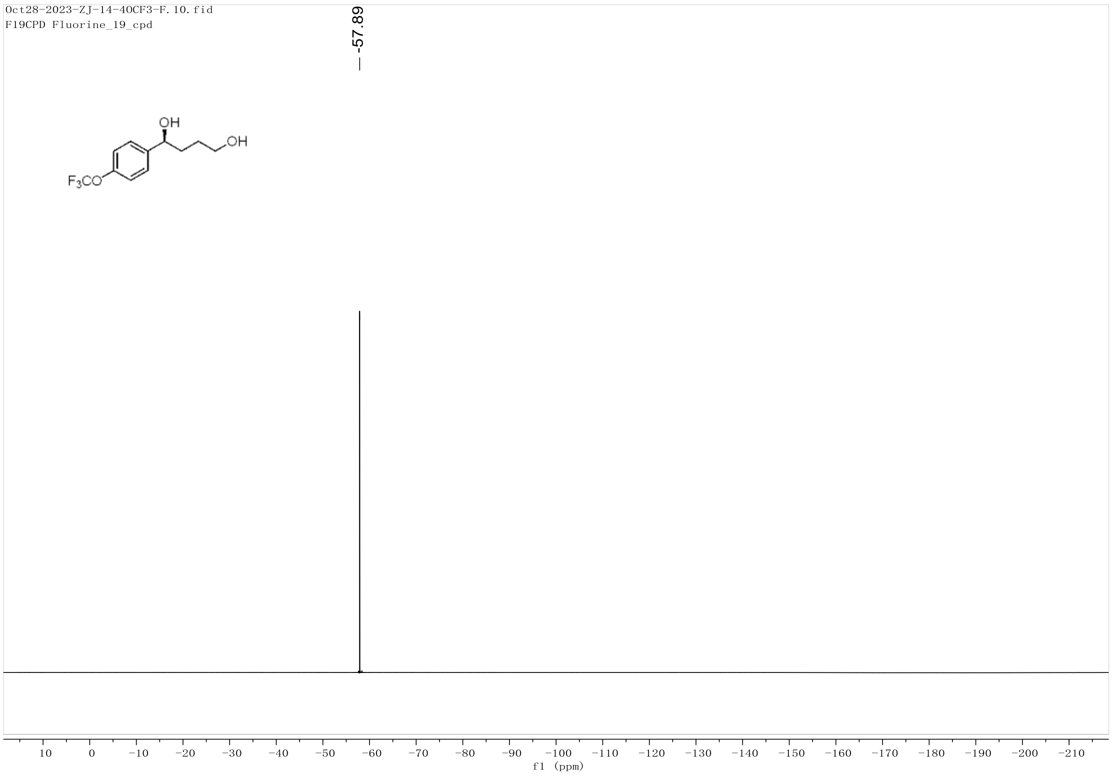

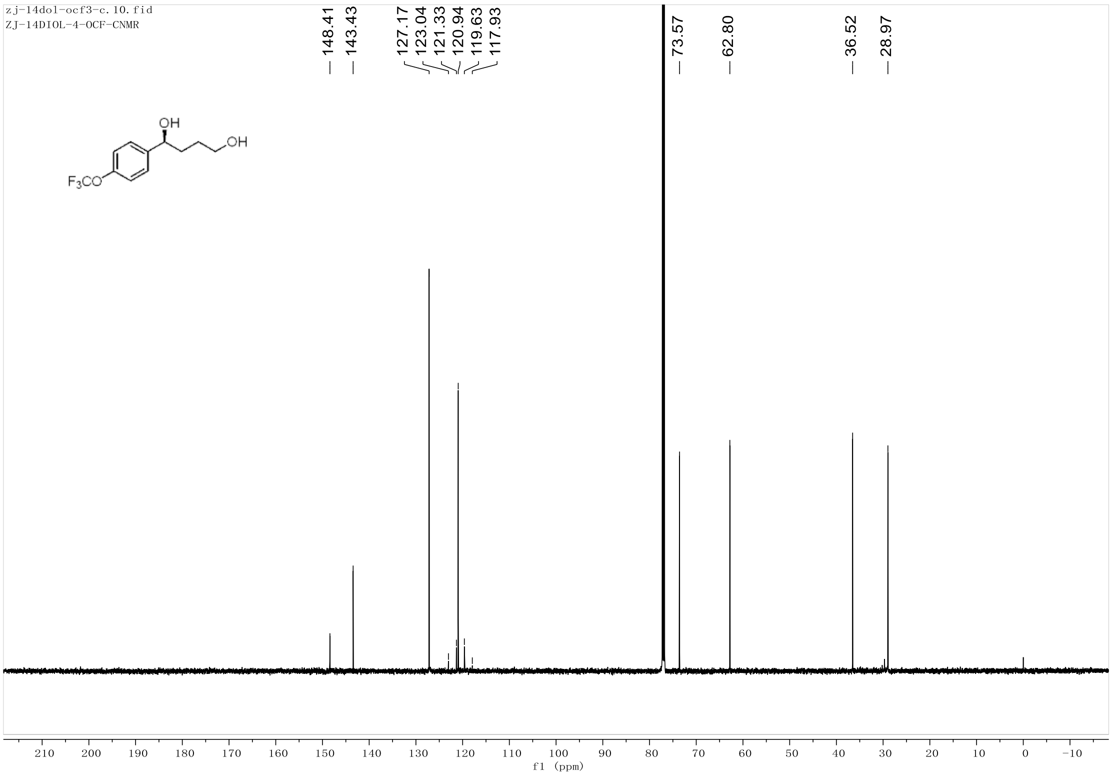

(*S*)-1-(4-(benzyloxy)phenyl)butane-1,4-diol (4i)

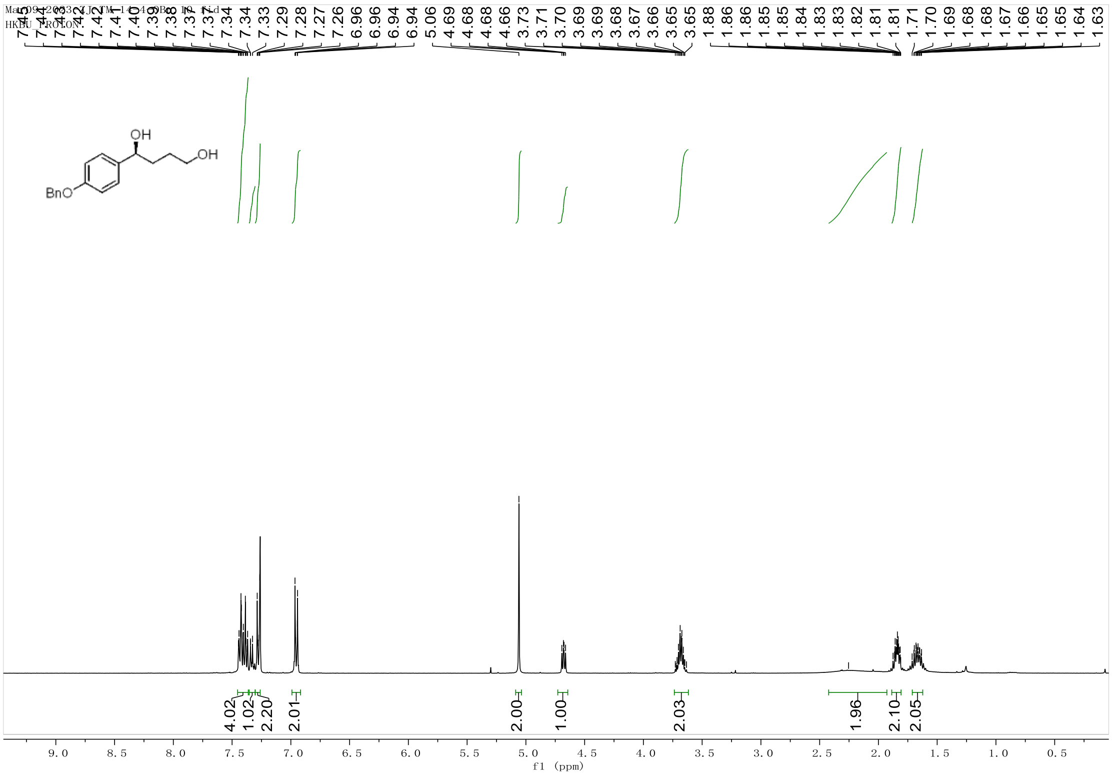

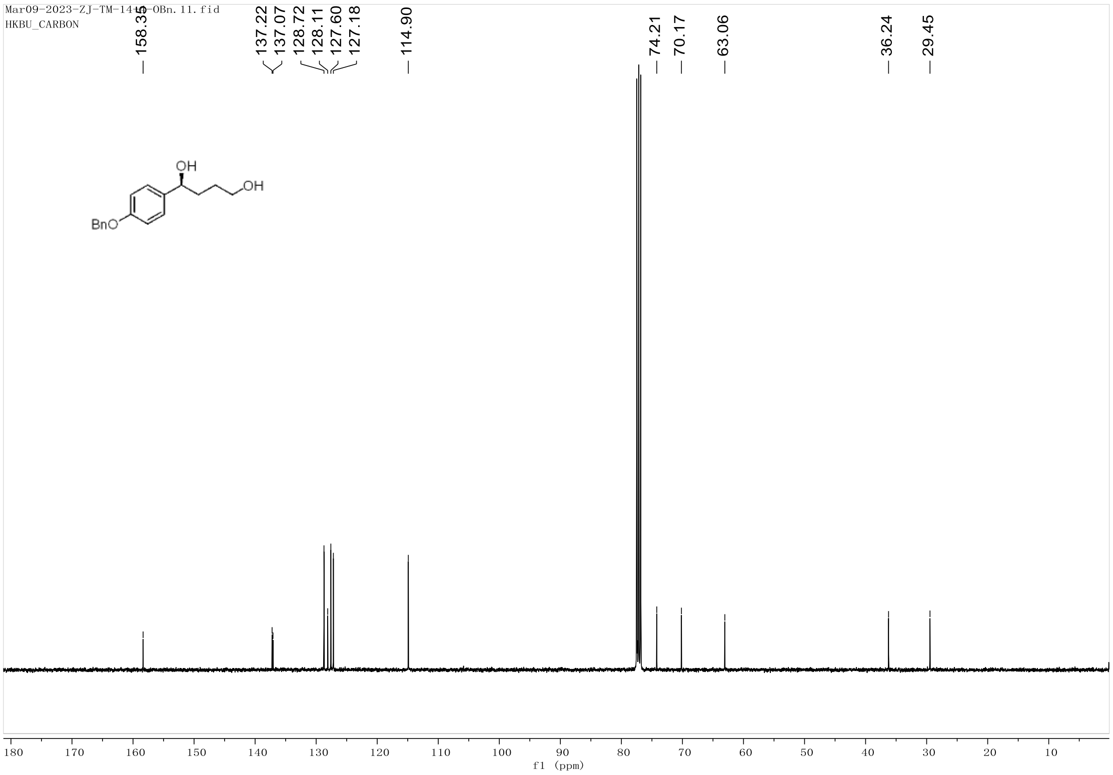

(*S*)-1-(2-fluorophenyl)butane-1,4-diol (4j)

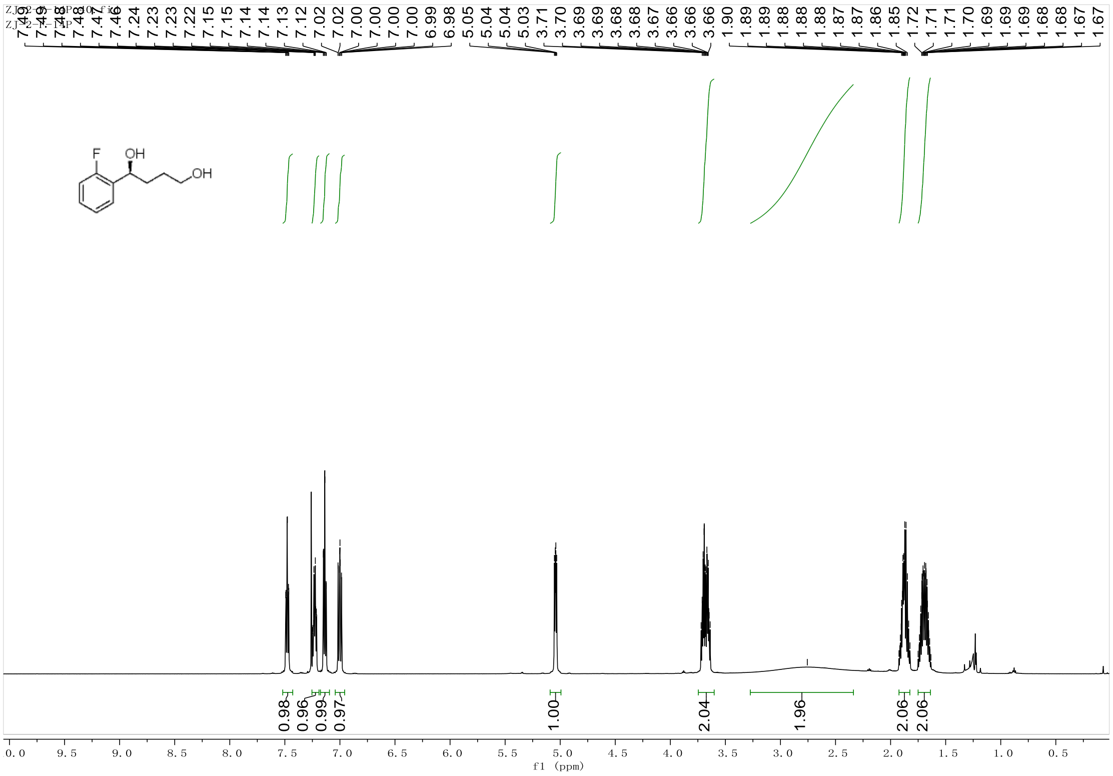

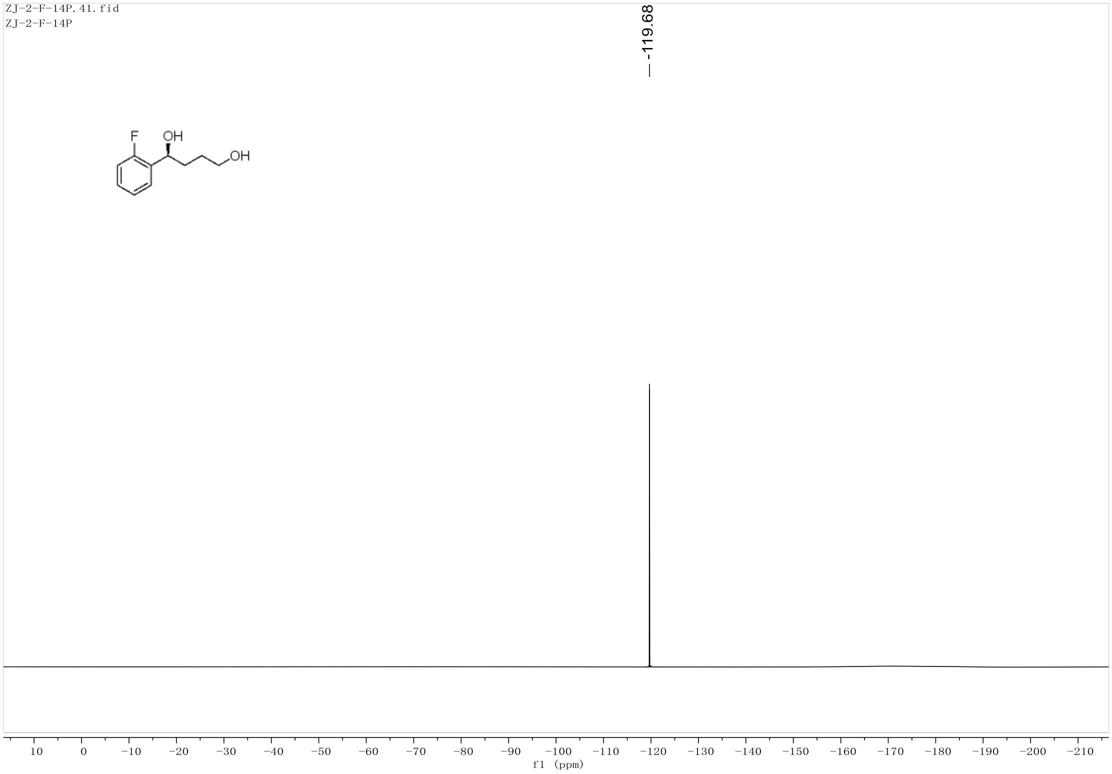
ZJ-2-F-14P.41.fid
ZJ-2-F-14P
-119.68
F
OH
OH
f1 (ppm)

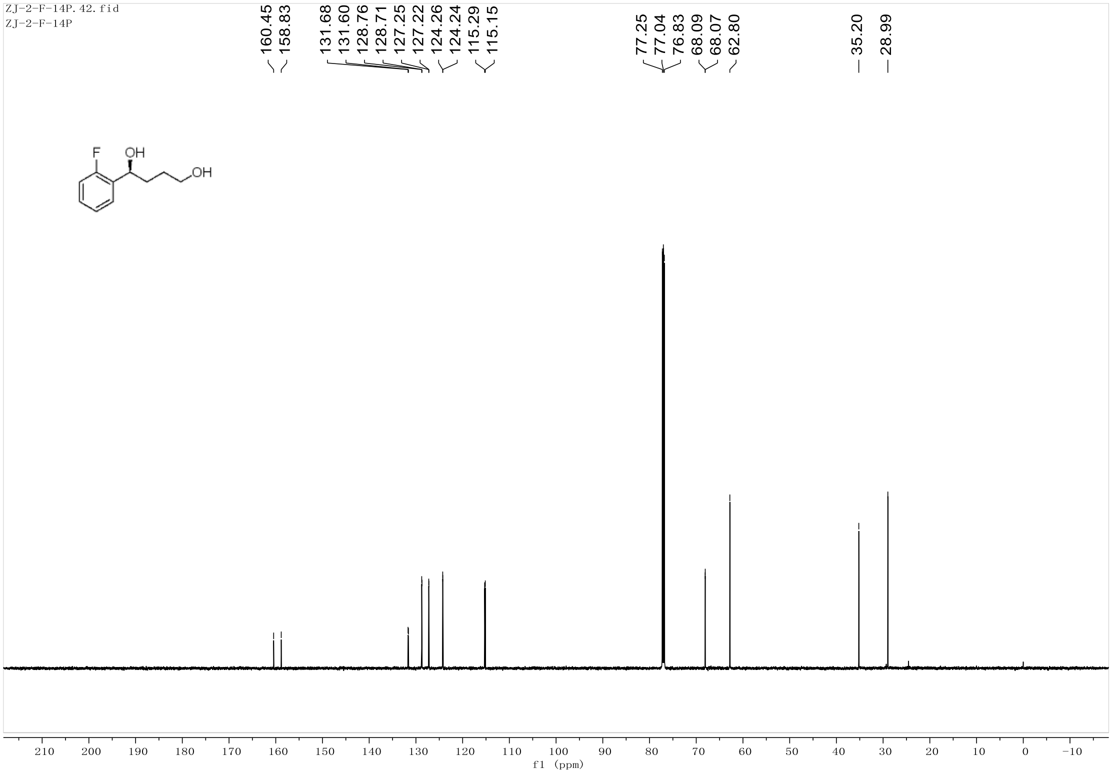
ZJ-2-F-14P.42.fid
ZJ-2-F-14P
160.45
158.83
131.68
131.60
128.76
128.71
127.25
127.22
124.26
124.24
115.29
115.15
77.25
77.04
76.83
68.09
68.07
62.80
35.20
28.99
F
OH
OH
f1 (ppm)

(*S*)-1-(4-fluorophenyl)butane-1,4-diol (4k)

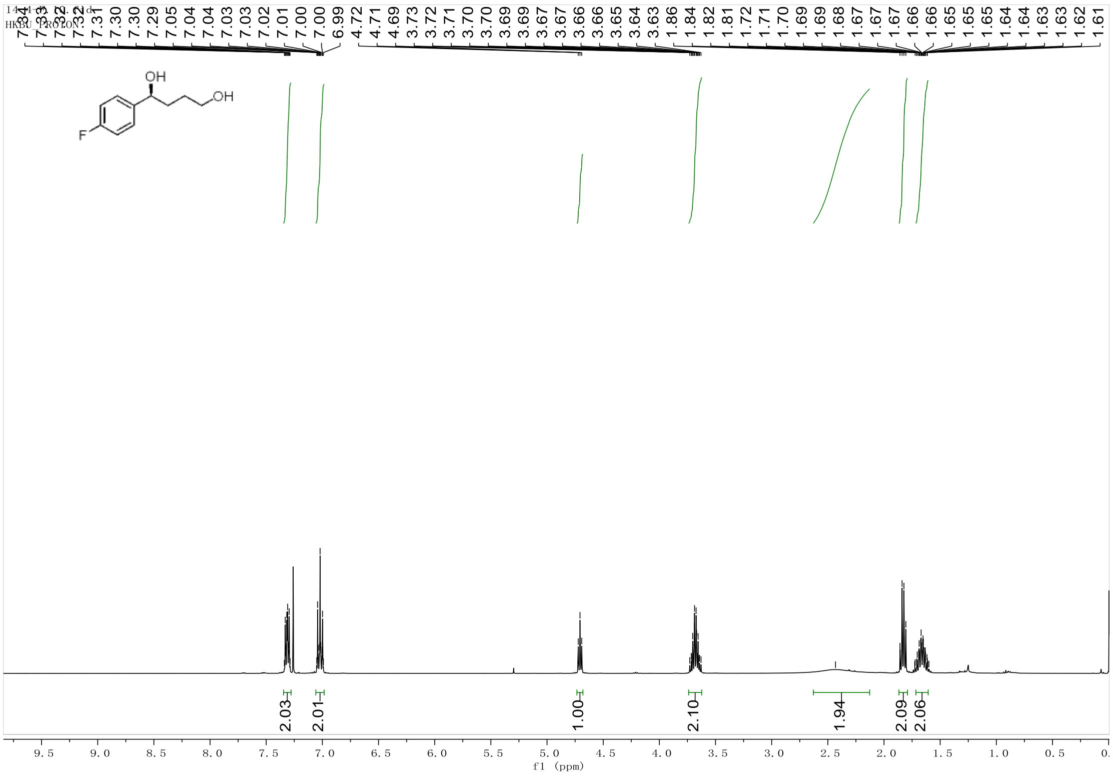

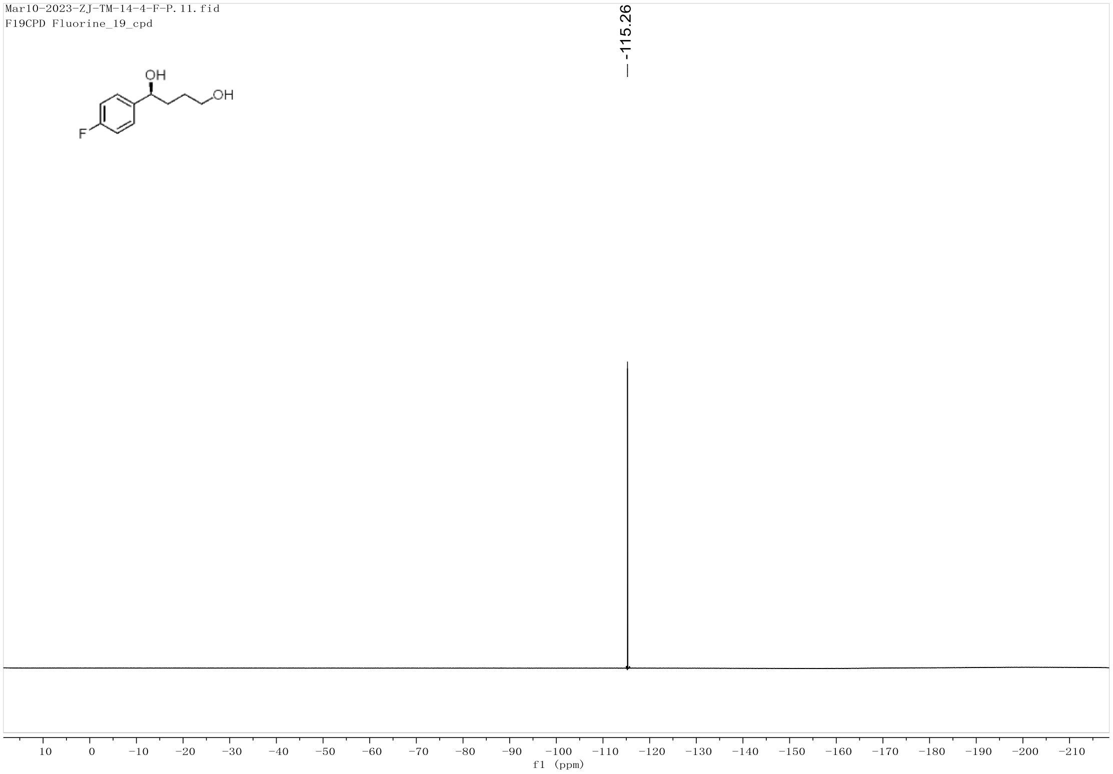

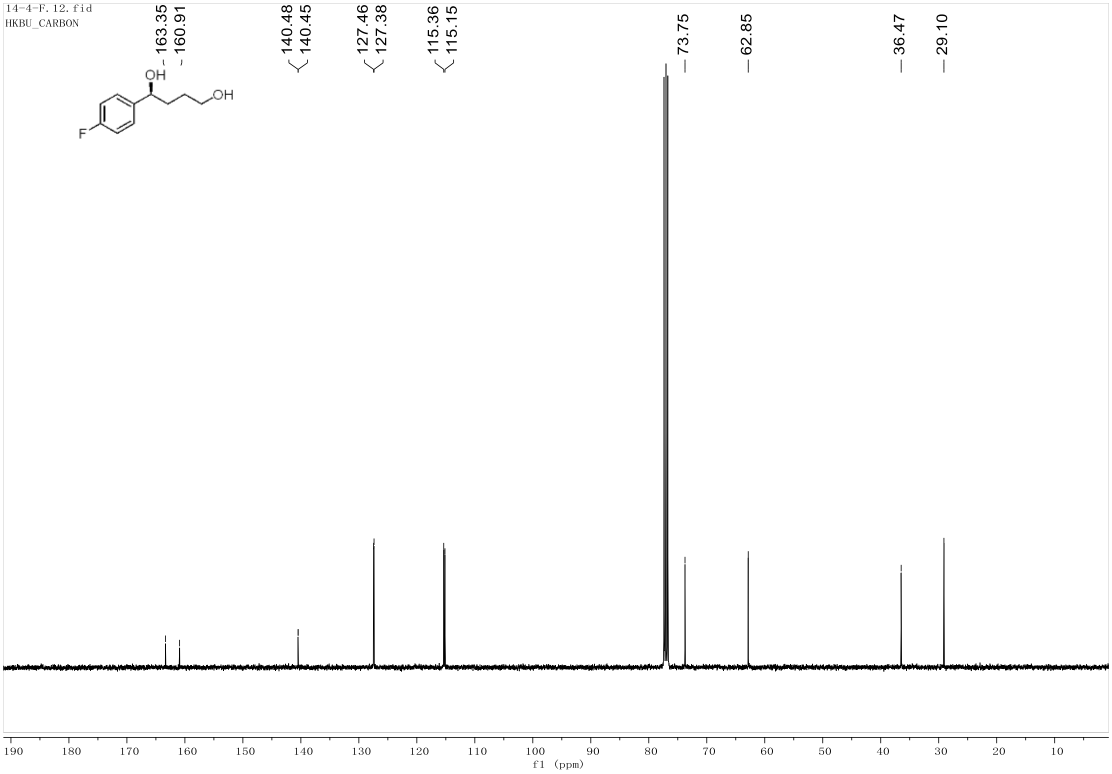

(*S*)-1-(4-(trifluoromethyl)phenyl)butane-1,4-diol (4l)

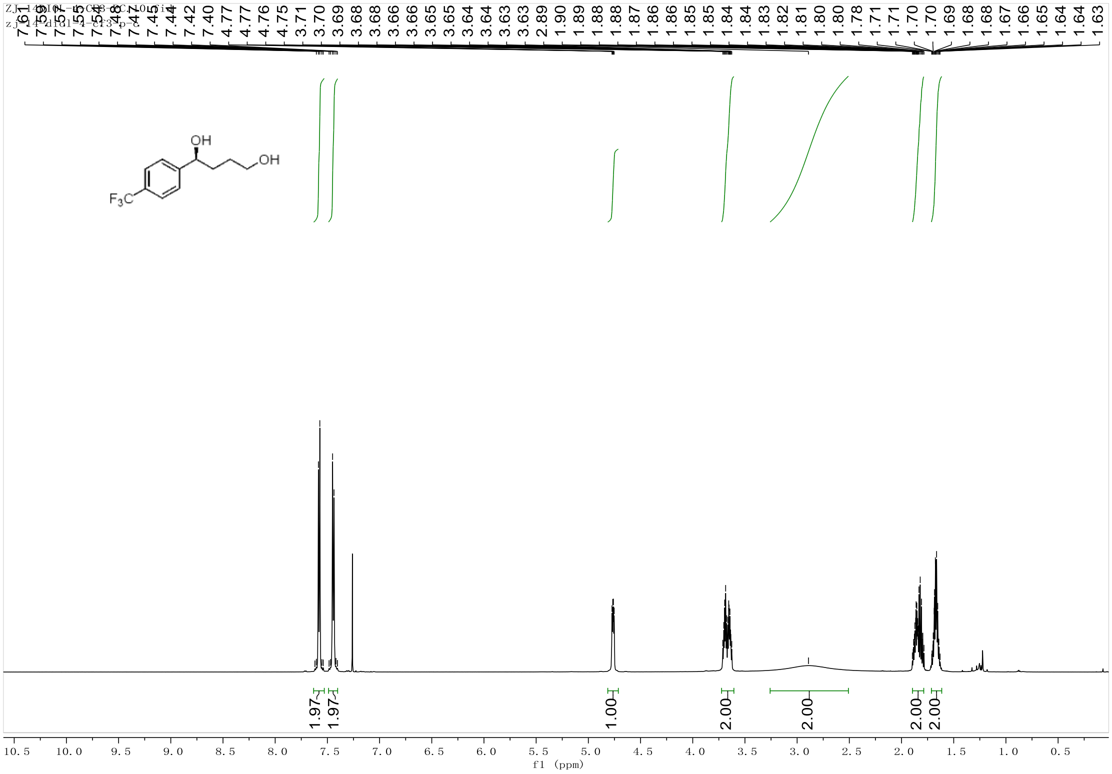

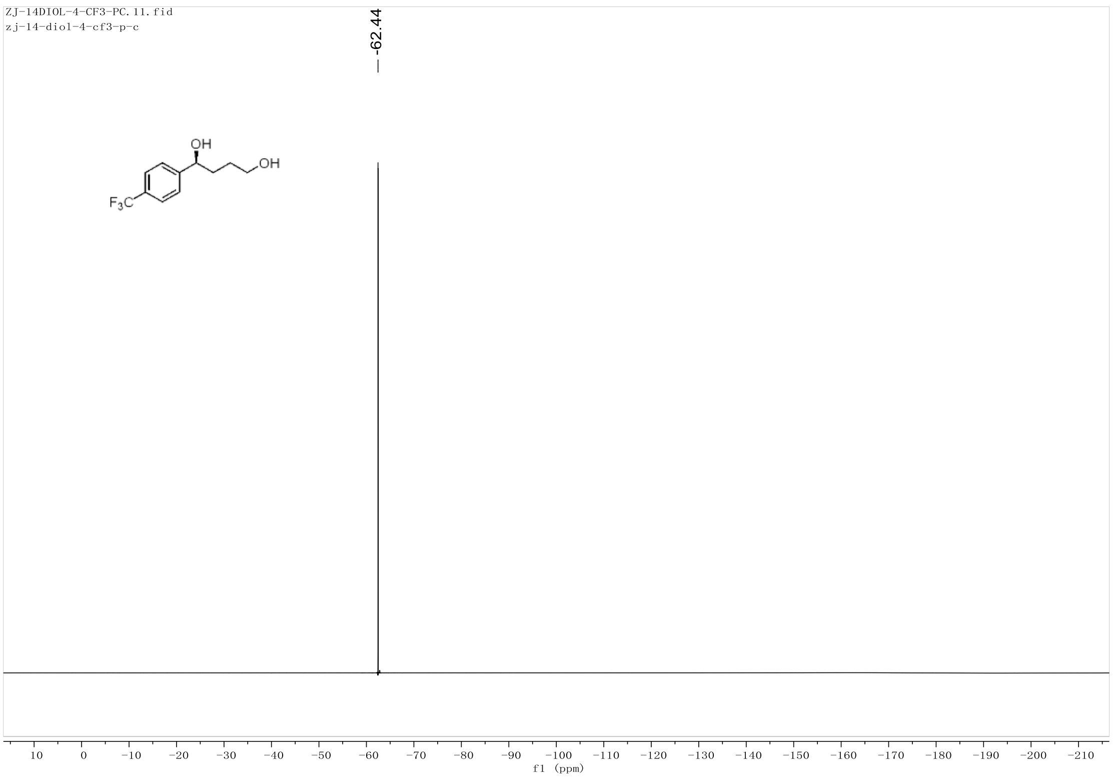
ZJ-14DIOL-4-CF3-PC.11.fid
zj-14-diol-4-cf3-p-c
-62.44
OH
OH
F3C
10 0 -10 -20 -30 -40 -50 -60 -70 -80 -90 -100 -110 -120 -130 -140 -150 -160 -170 -180 -190 -200 -210
f1 (ppm)

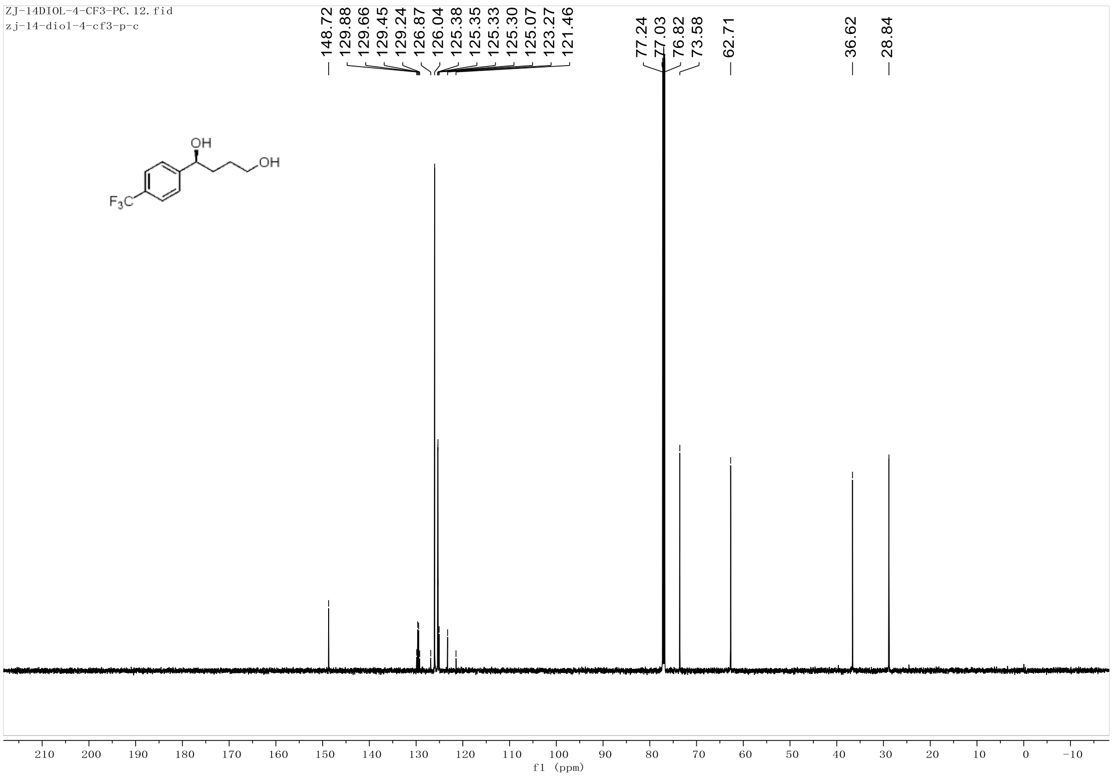
ZJ-14DIOL-4-CF3-PC.12.fid
zj-14-diol-4-cf3-p-c
148.72
129.88
129.66
129.45
129.24
126.87
126.04
125.38
125.35
125.33
125.30
125.07
123.27
121.46
77.24
77.03
76.82
73.58
62.71
36.62
28.84
OH
OH
F3C
210 200 190 180 170 160 150 140 130 120 110 100 90 80 70 60 50 40 30 20 10 0 -10
f1 (ppm)

(*S*)-1-(3-bromophenyl)butane-1,4-diol (4m)

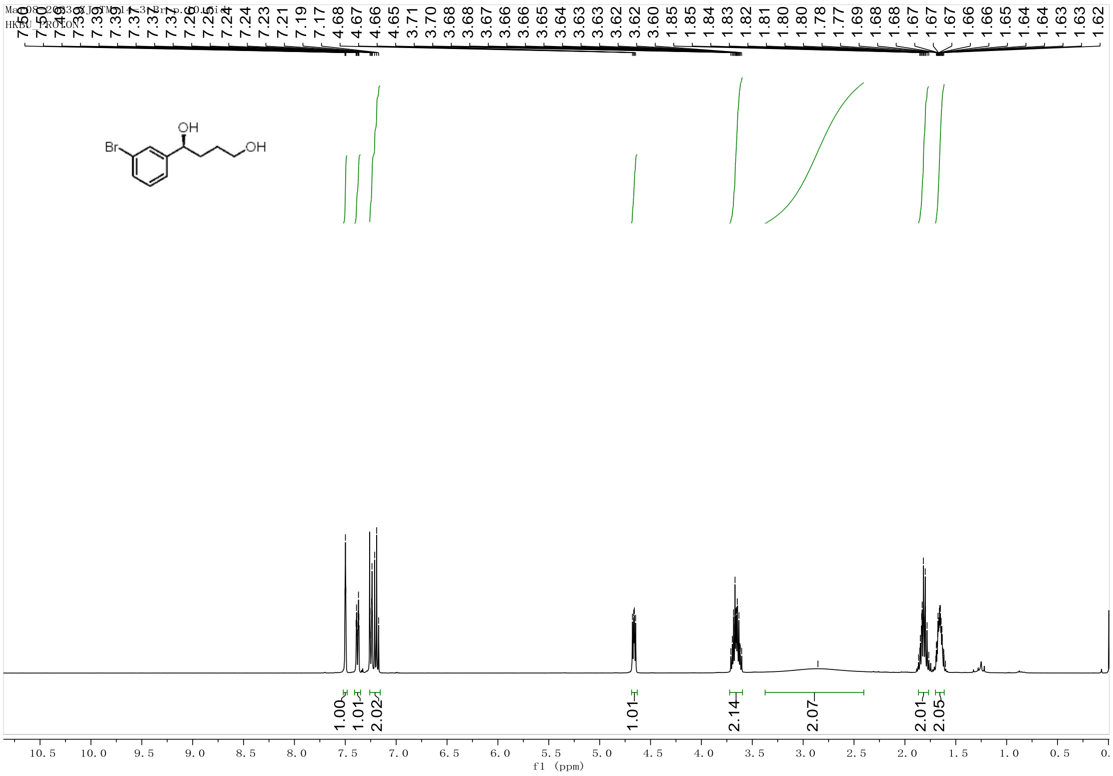

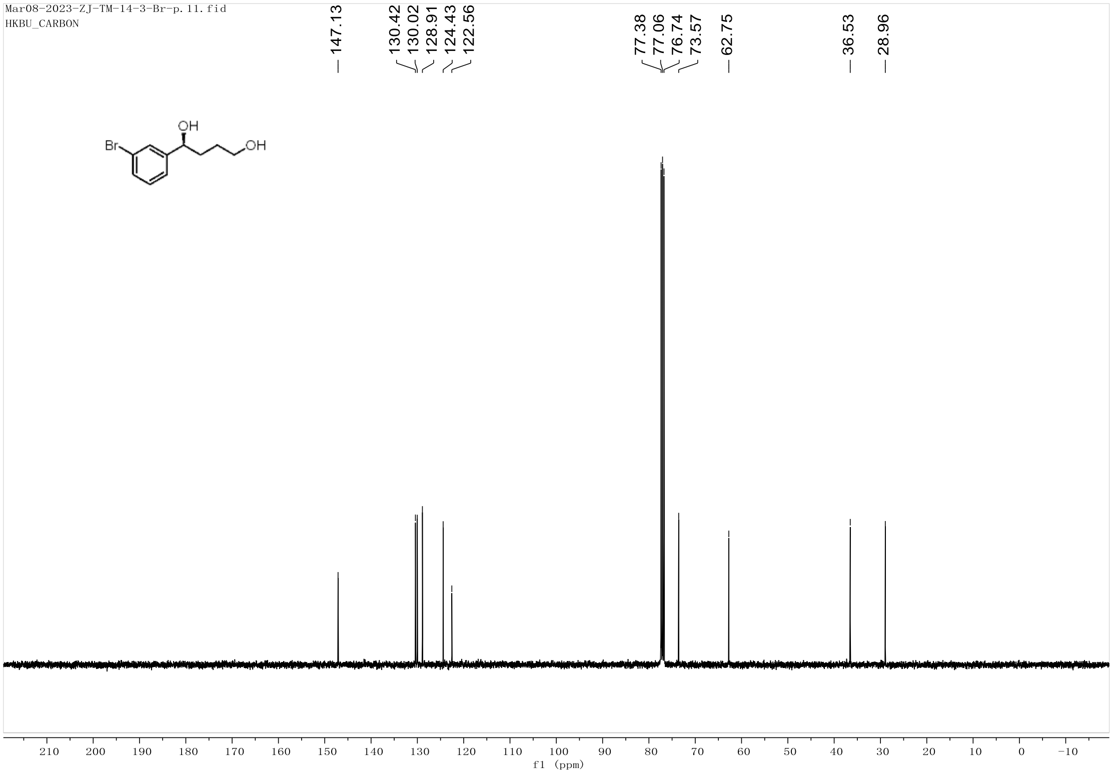

(*S*)-1-([1,1'-biphenyl]-4-yl)butane-1,4-diol (4n)

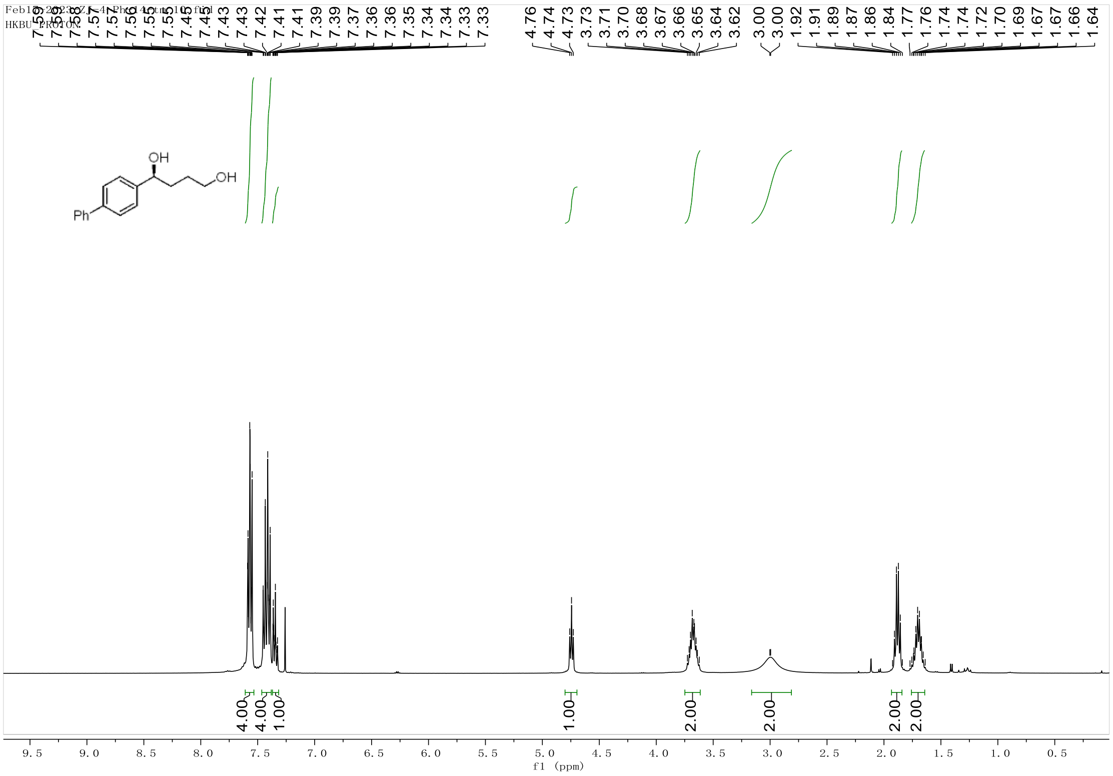

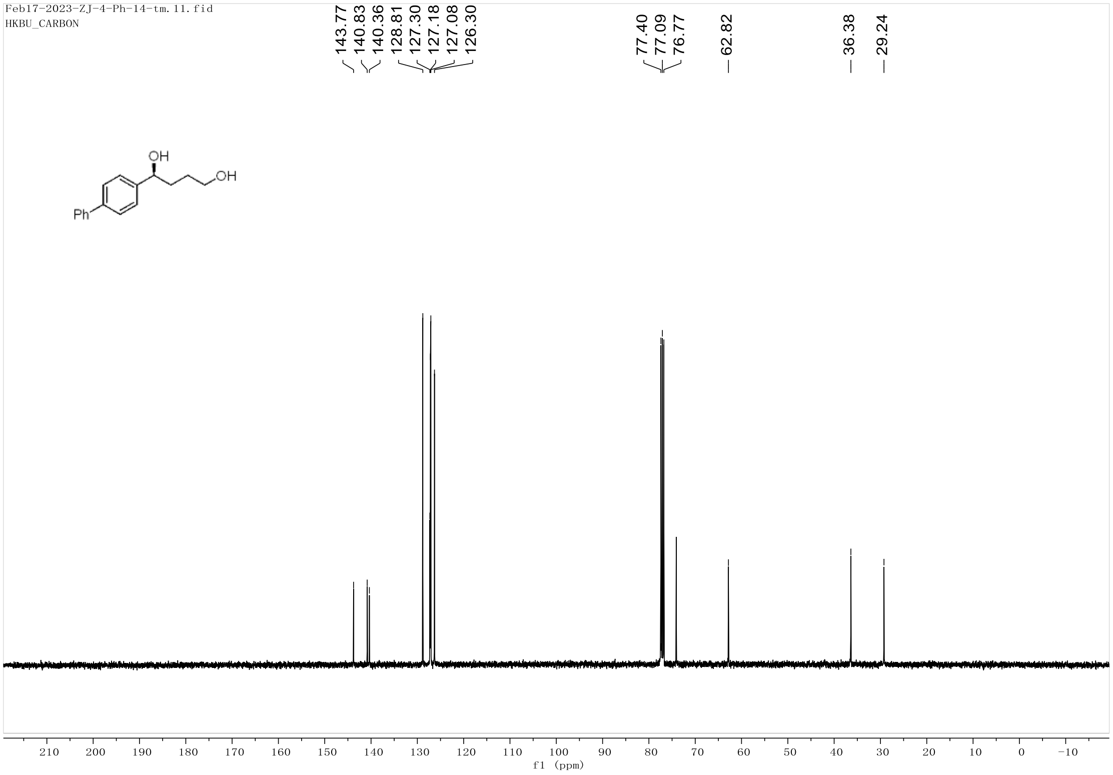

(*S*)-1-(naphthalen-1-yl)butane-1,4-diol (4o)

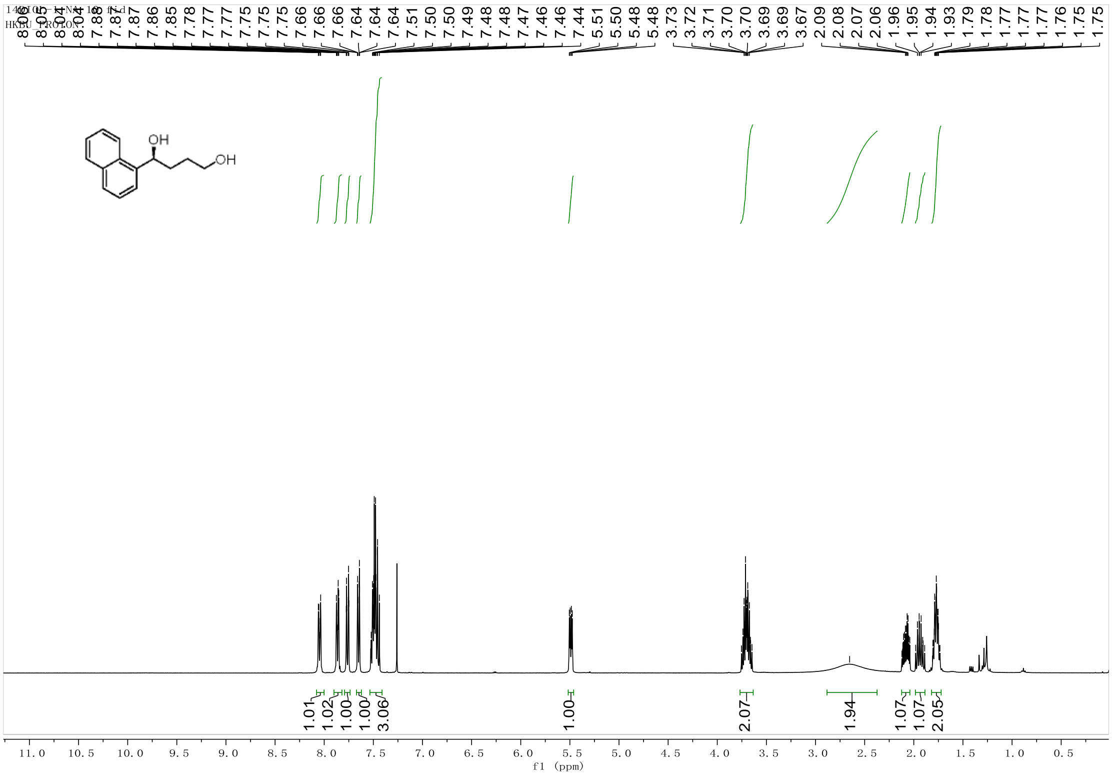

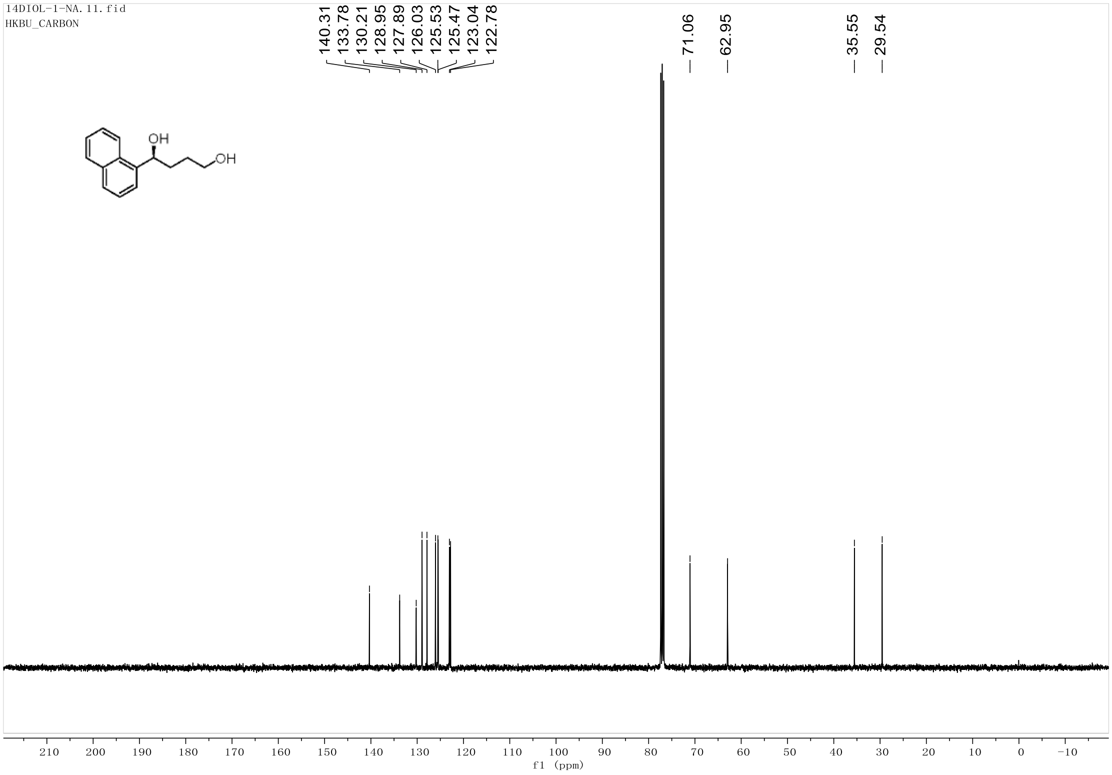

(*S*)-1-(naphthalen-2-yl)butane-1,4-diol (4p)

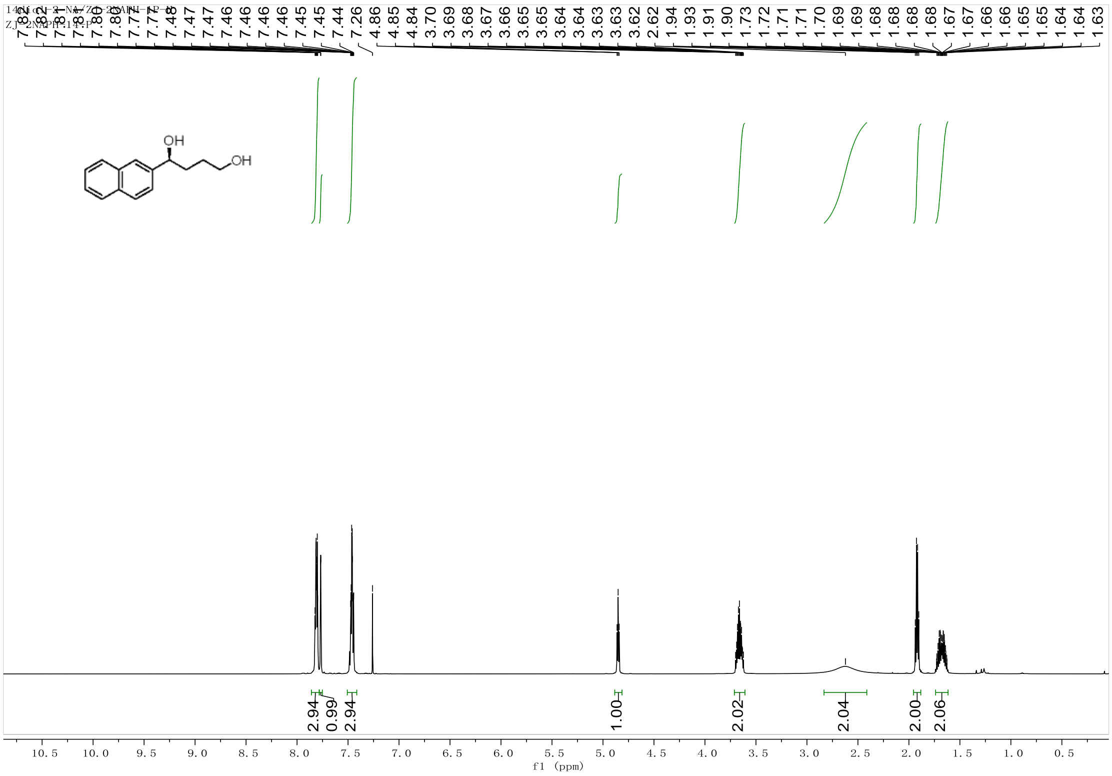

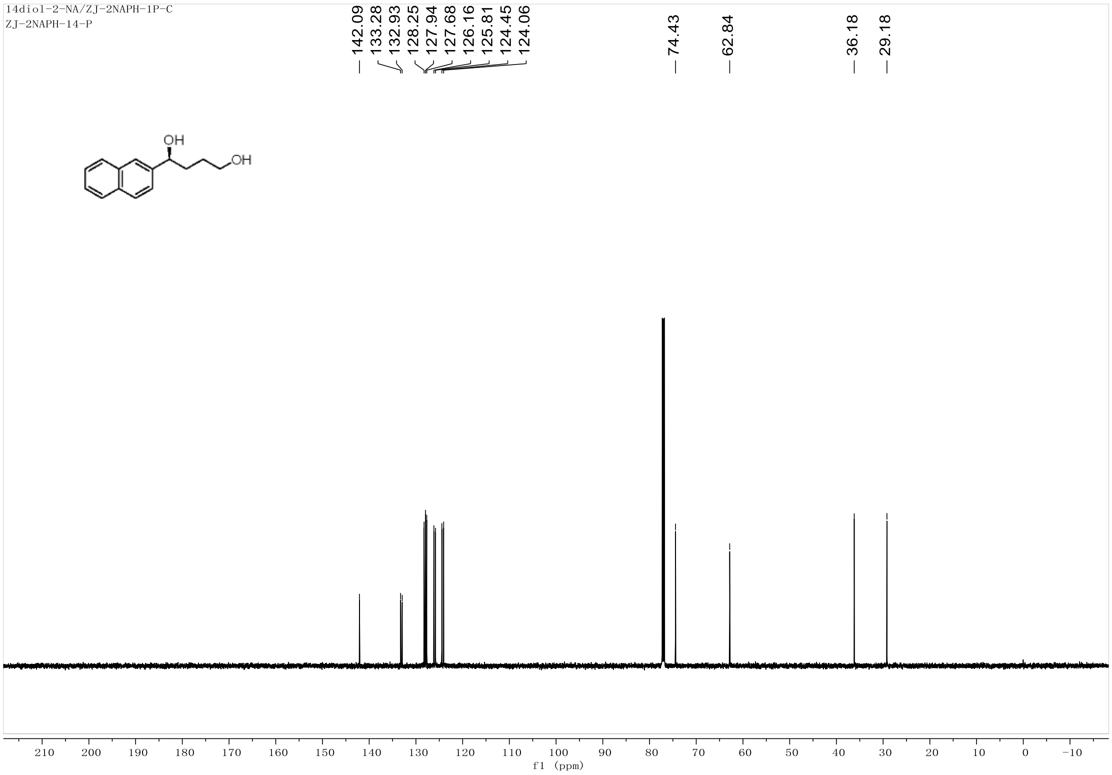

(*S*)-1-(benzofuran-3-yl)butane-1,4-diol (4q)

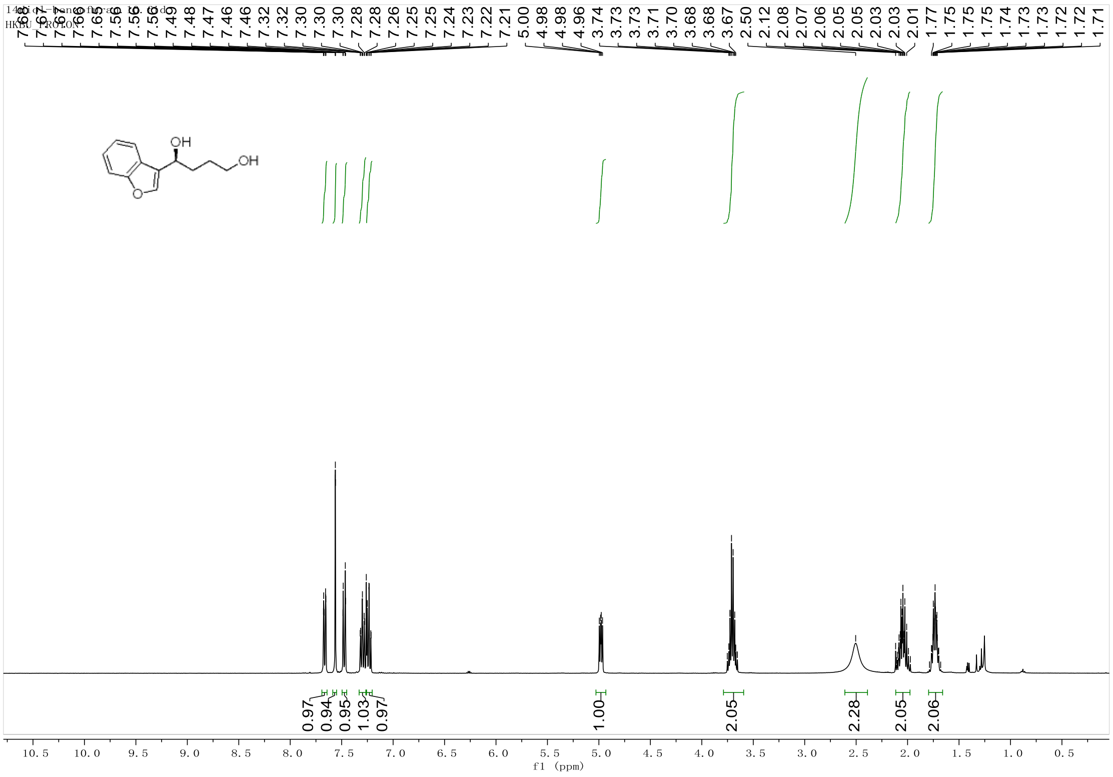

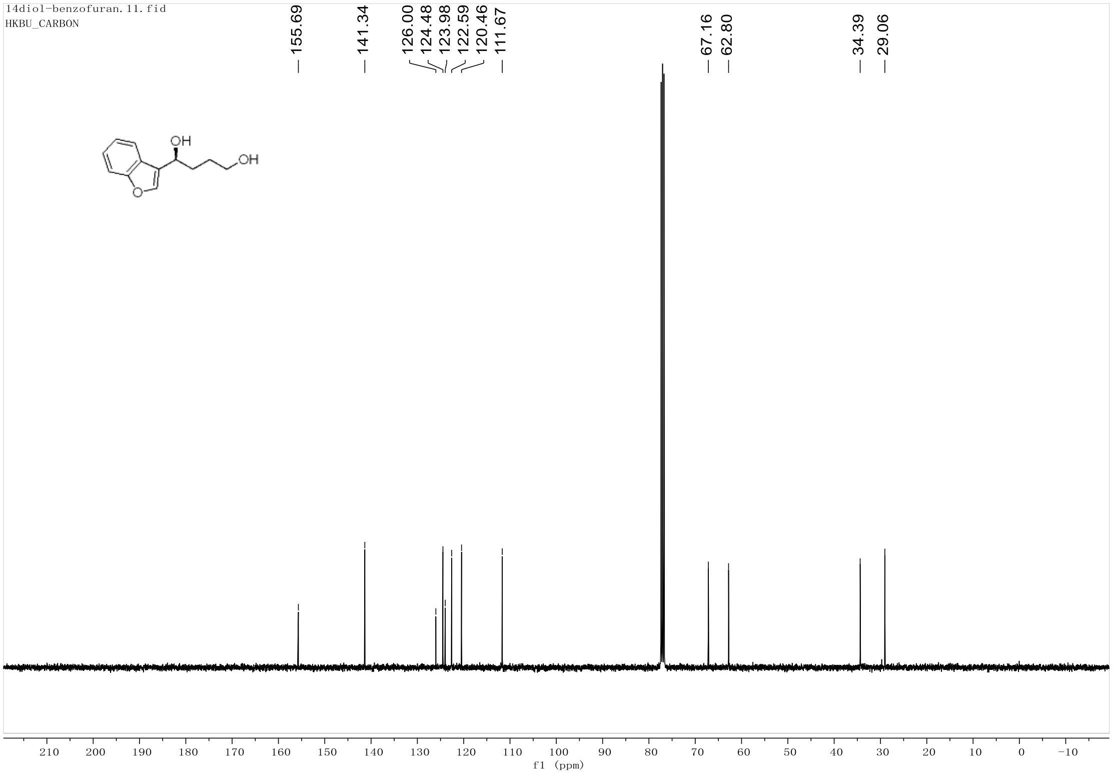

(*S*)-1-(3,4-difluorophenyl)butane-1,4-diol (4r)

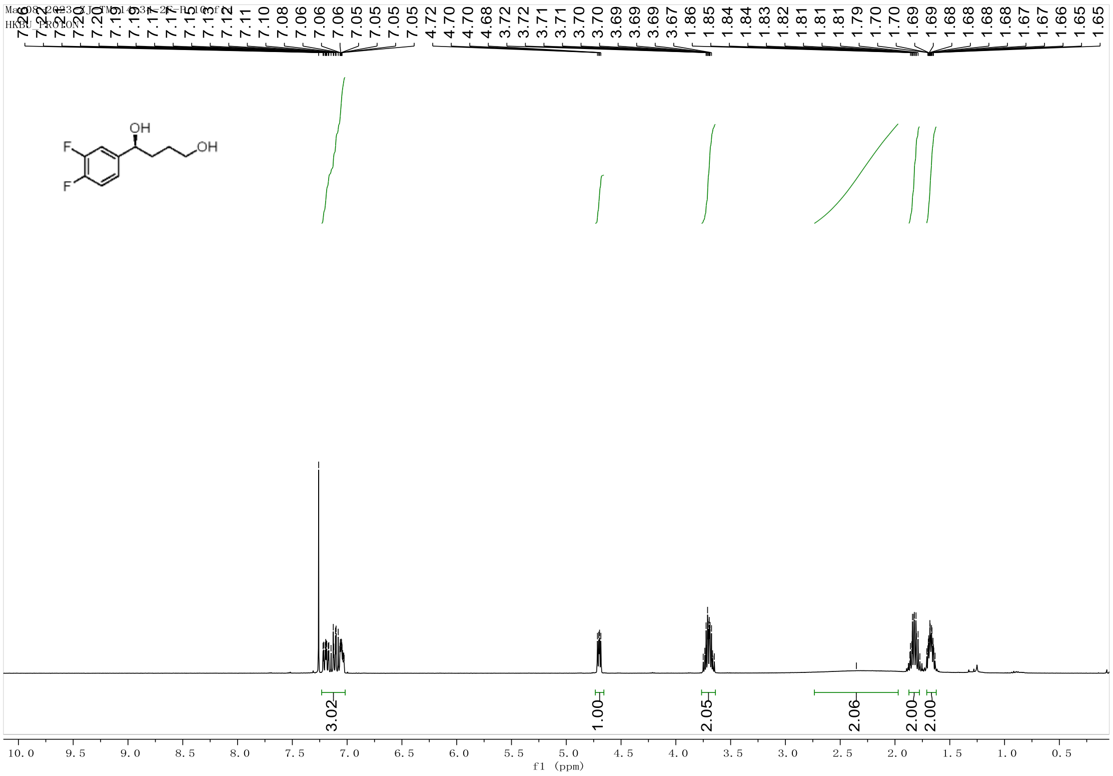

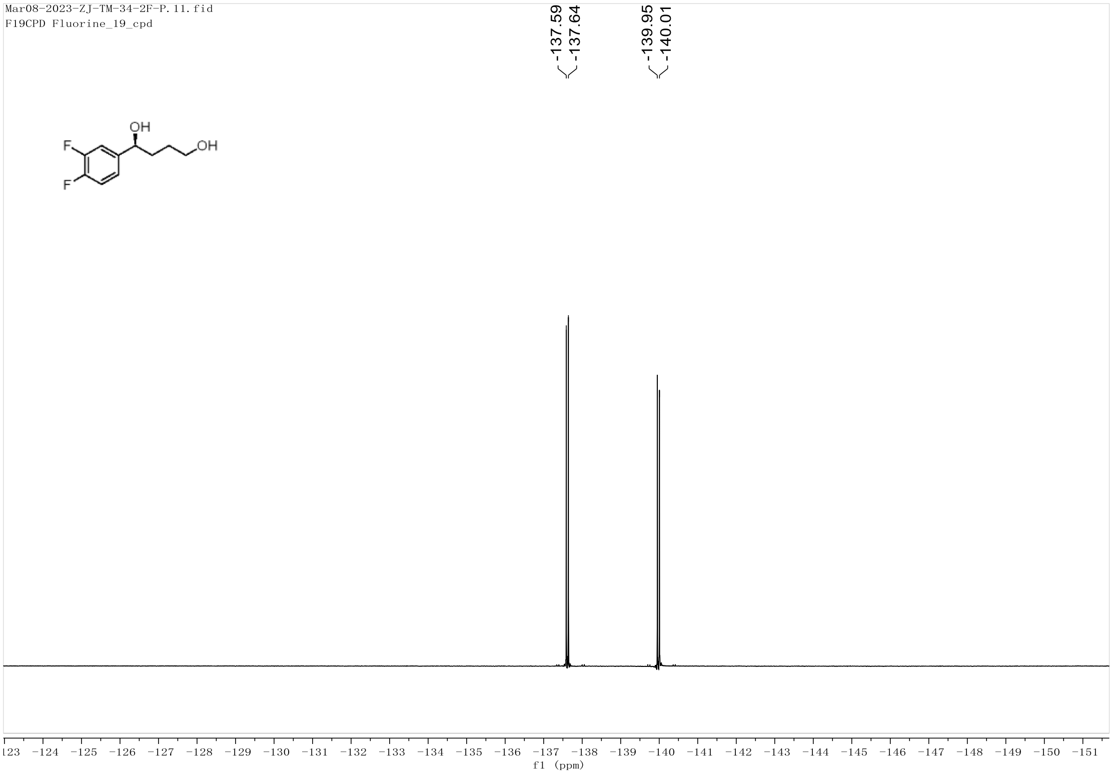

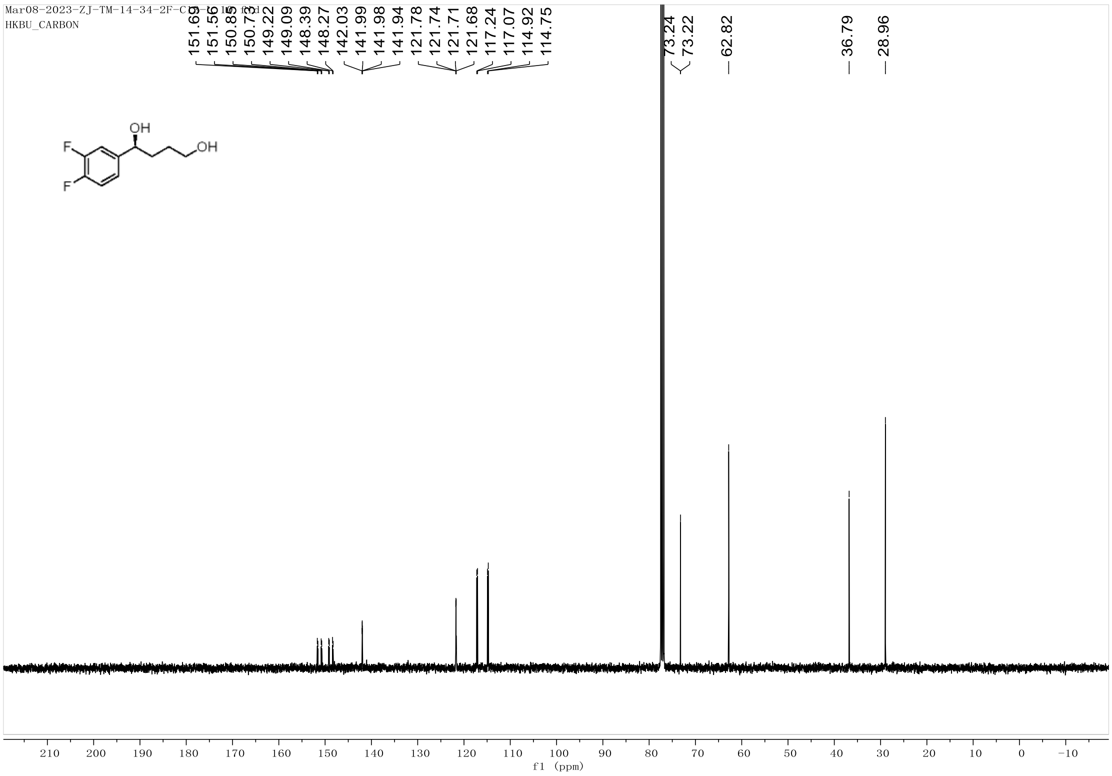

(*S*)-1-(3,4,5-trimethoxyphenyl)butane-1,4-diol (4s)

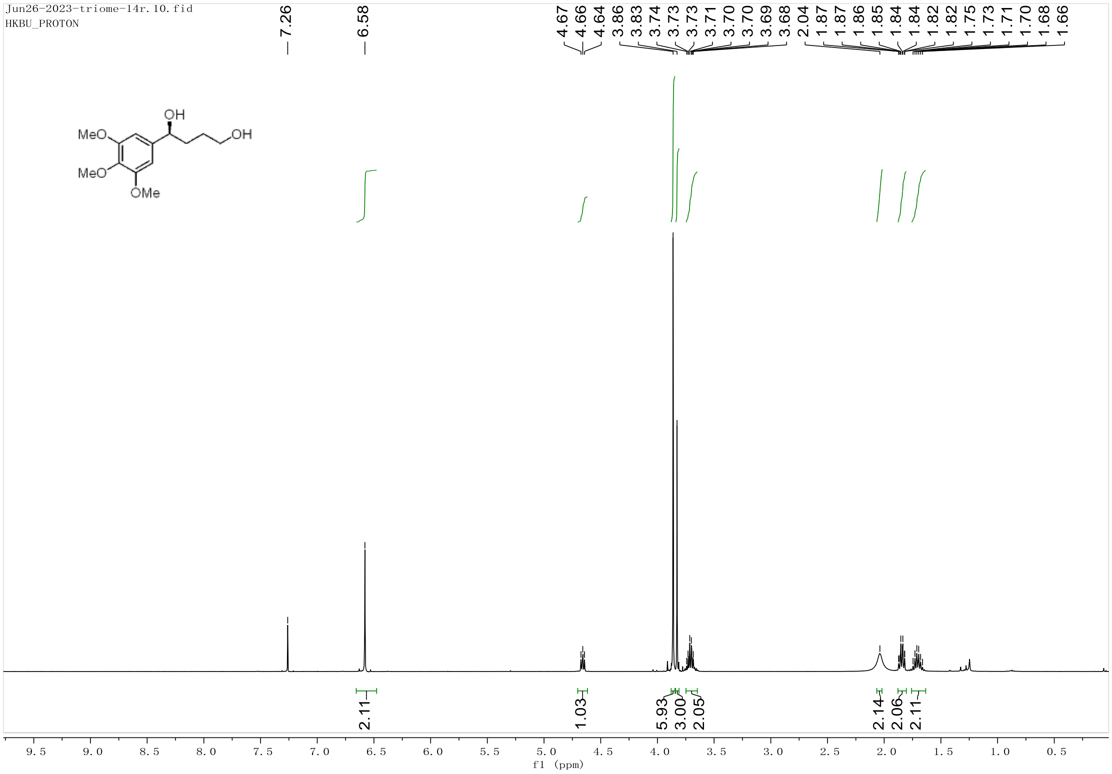

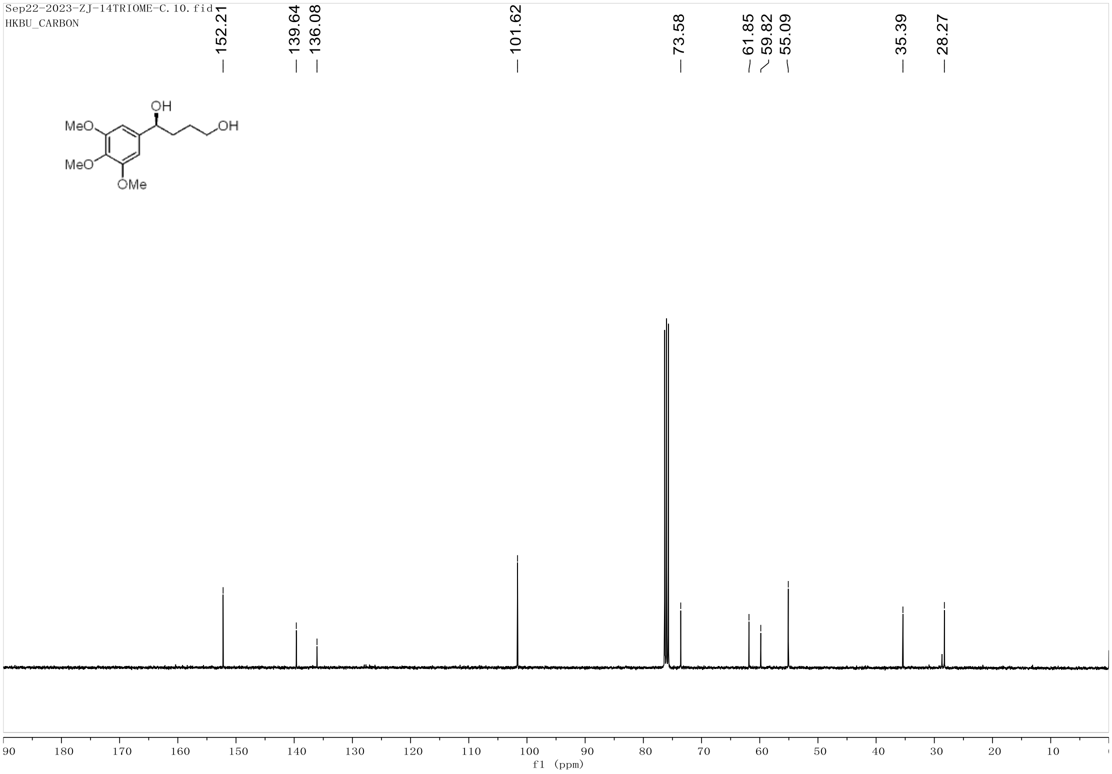

(*R*)-2,2'-(4-phenylbutane-1,3-diyl)bis(4,4,5,5-tetramethyl-1,3,2-dioxaborolane) (6a)

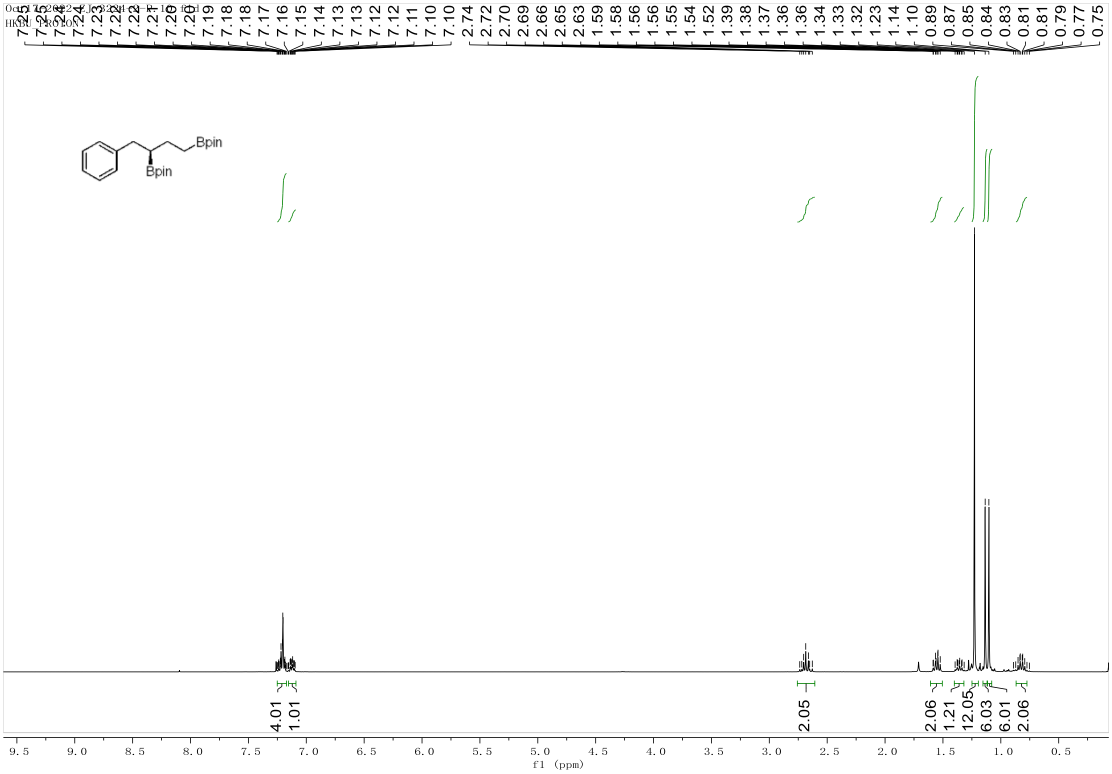

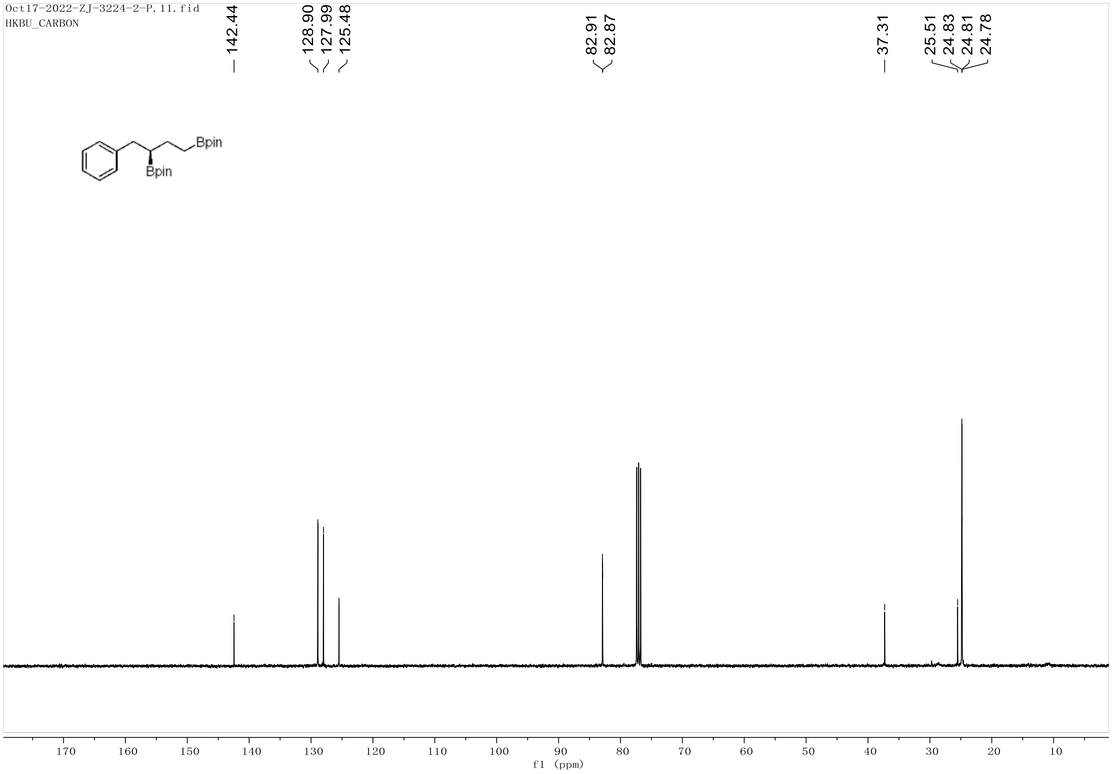

(*S*)-4-phenylbutane-1,3-diol (7a)

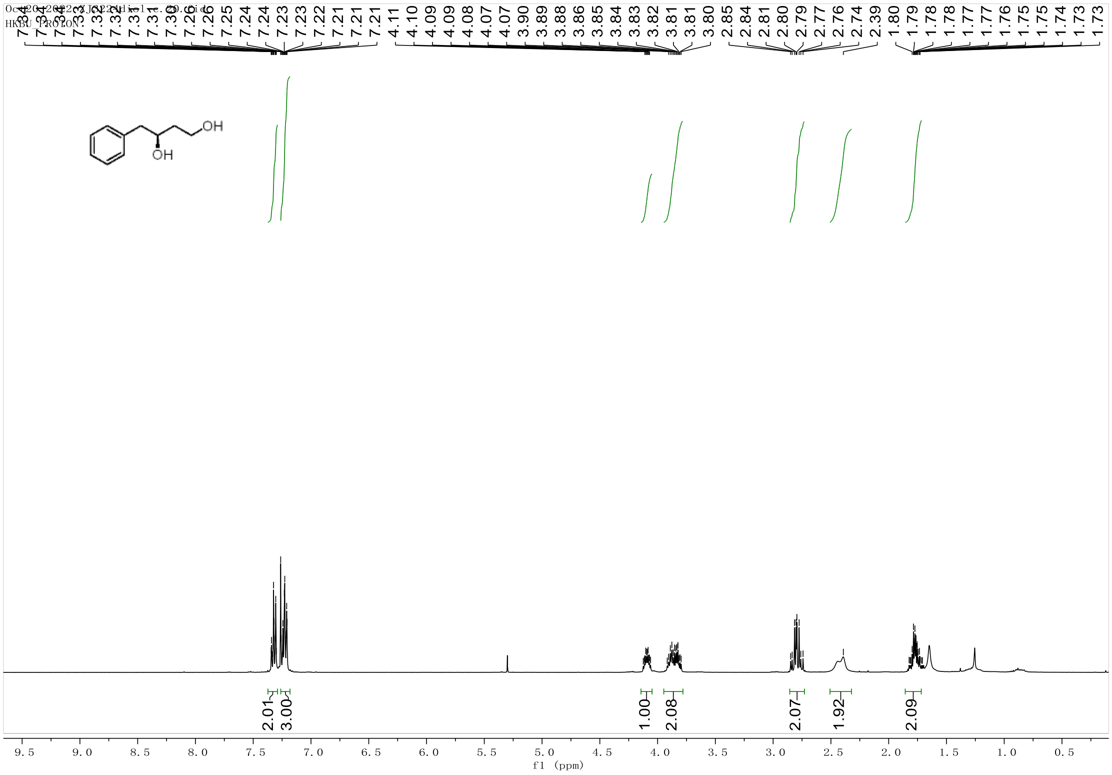

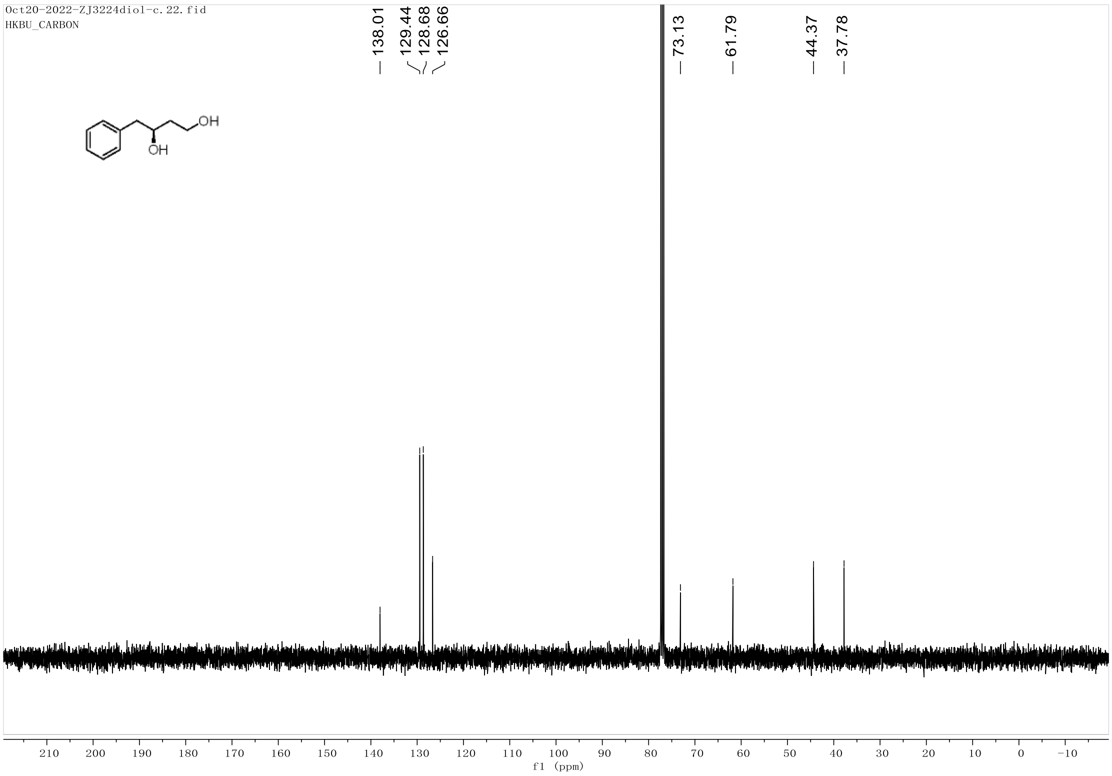

(*S*)-4-(o-tolyl)butane-1,3-diol (7b)

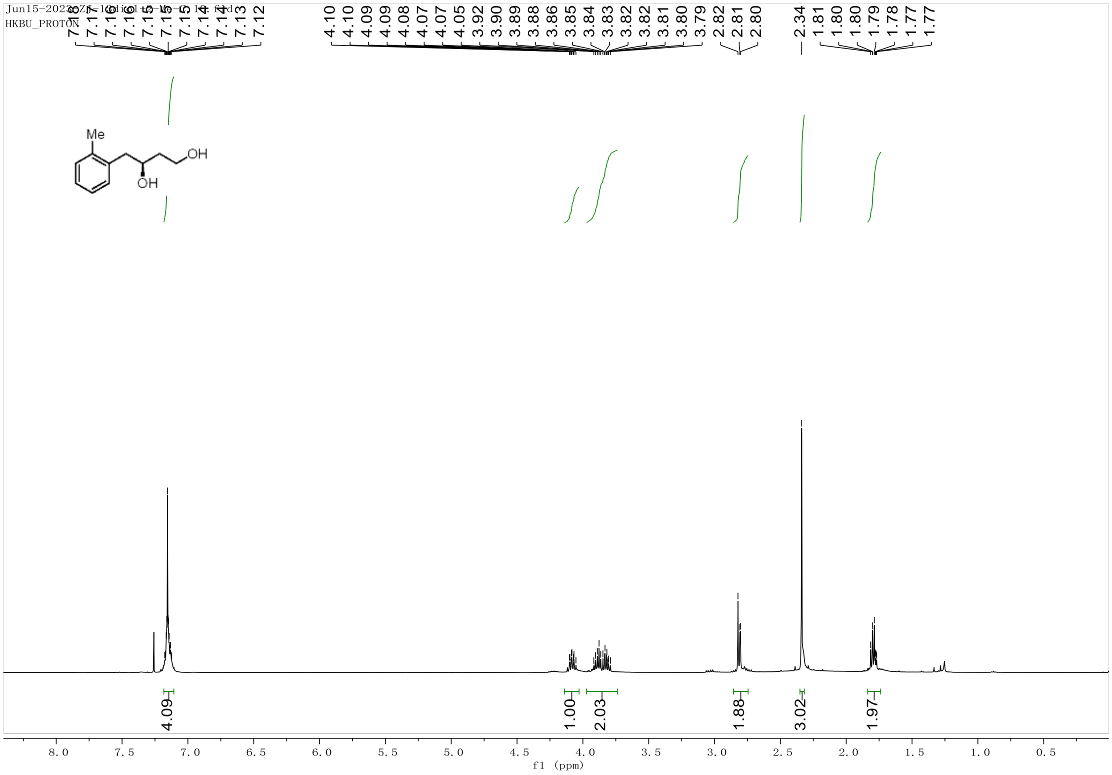

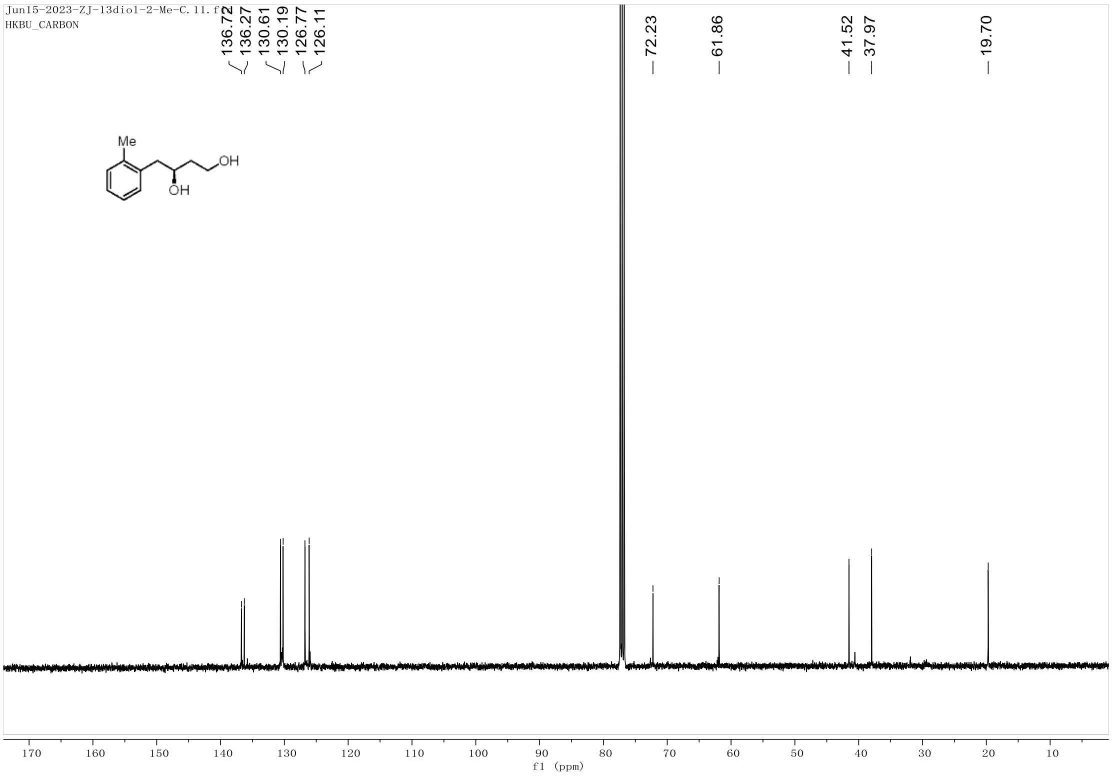

(*S*)-4-(4-(tert-butyl)phenyl)butane-1,3-diol (7c)

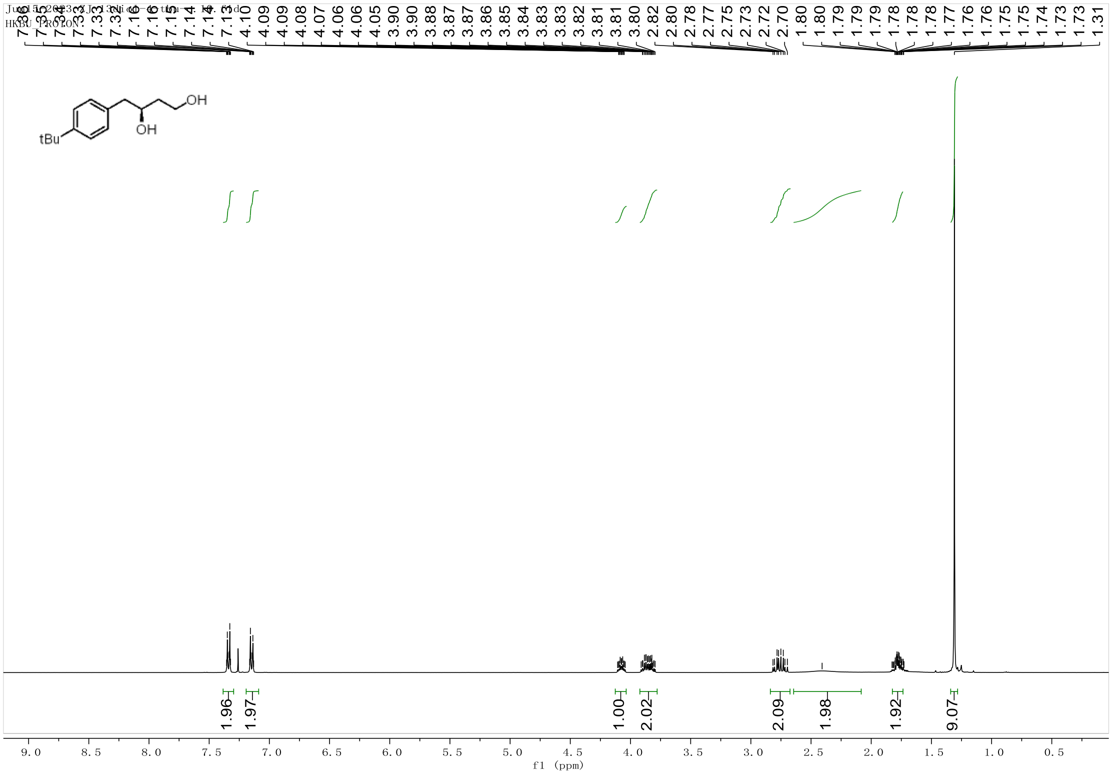

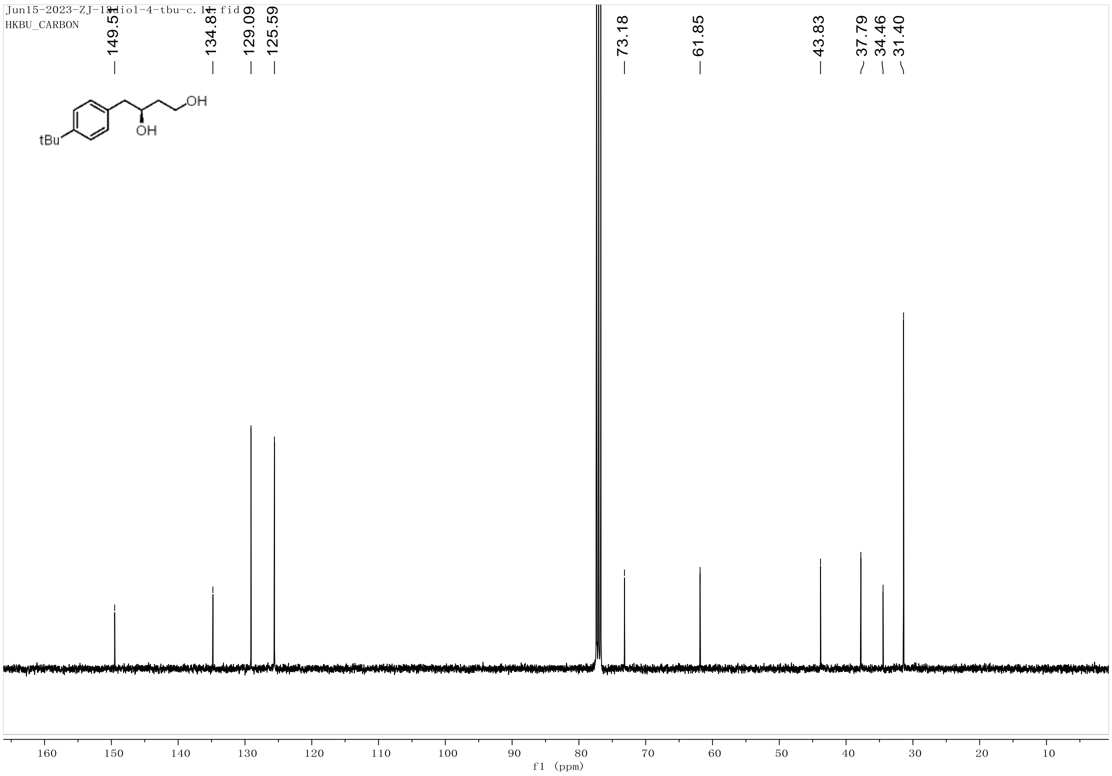

(*S*)-4-(3-methoxyphenyl)butane-1,3-diol (7d)

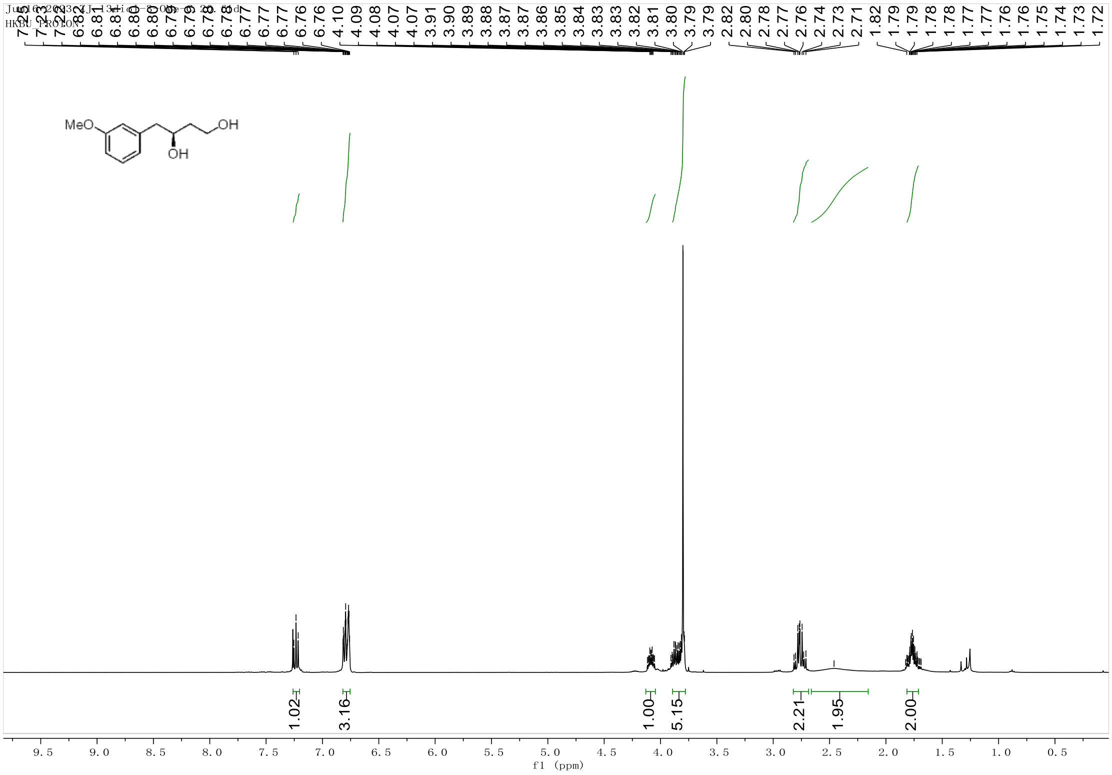

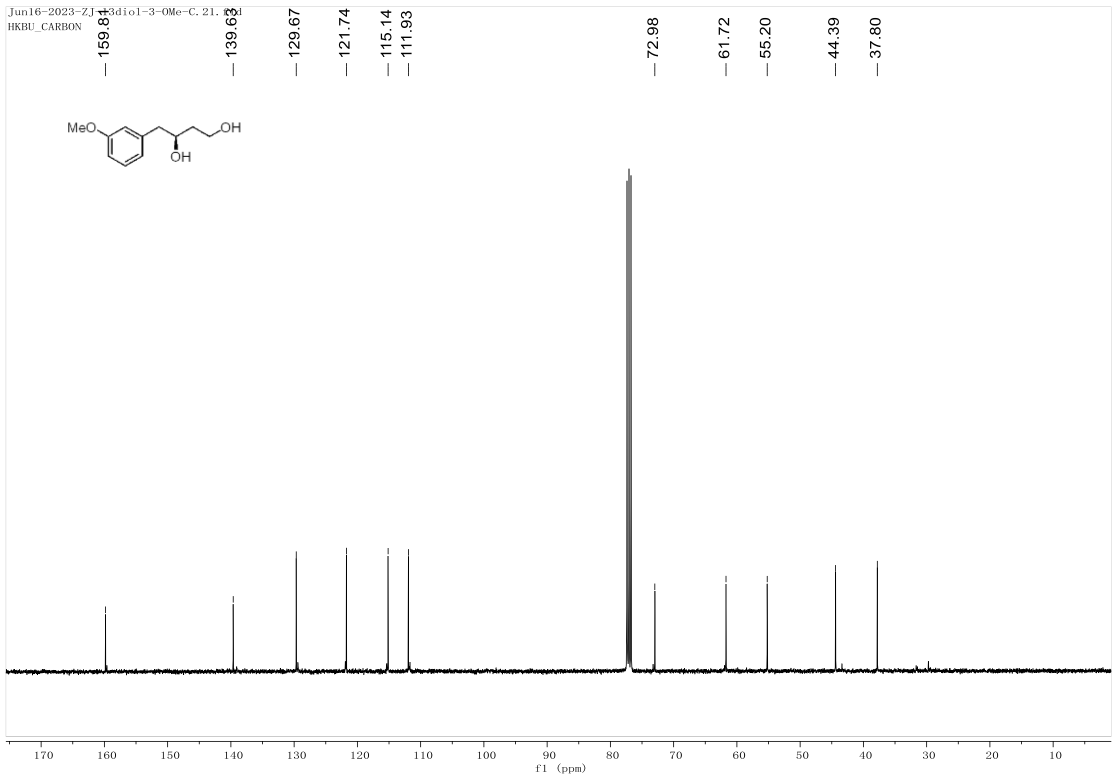

(*S*)-4-(4-(trifluoromethoxy)phenyl)butane-1,3-diol (7e)

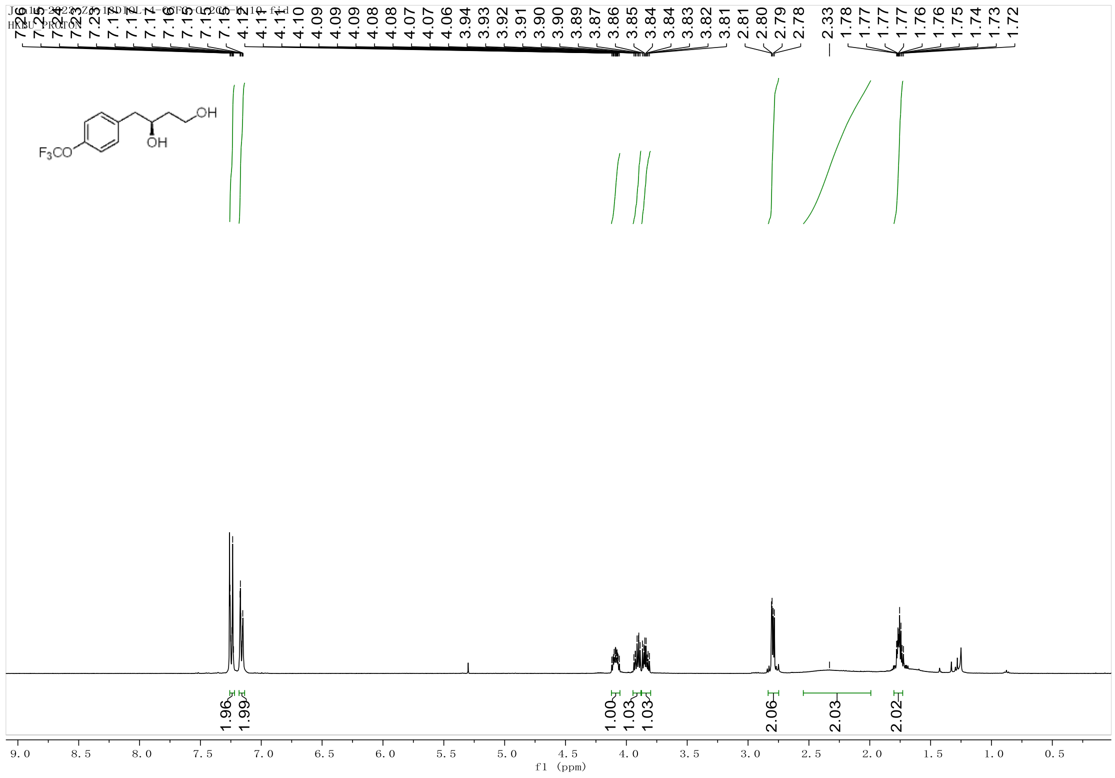

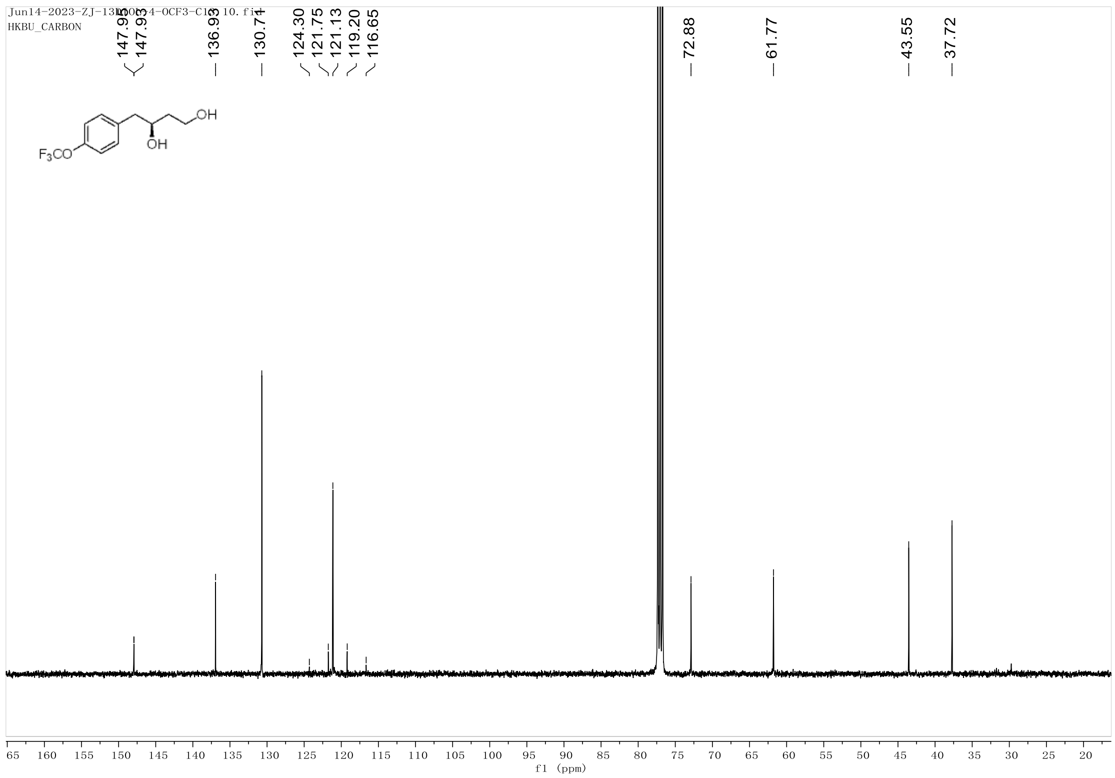
Jun14-2023-ZJ-13DIOL-4-OCF3-C13.10.fid
HKBU_CARBON
147.95
147.93
136.93
130.71
124.30
121.75
121.13
119.20
116.65
72.88
61.77
43.55
37.72
F3CO
OH
OH
f1 (ppm)

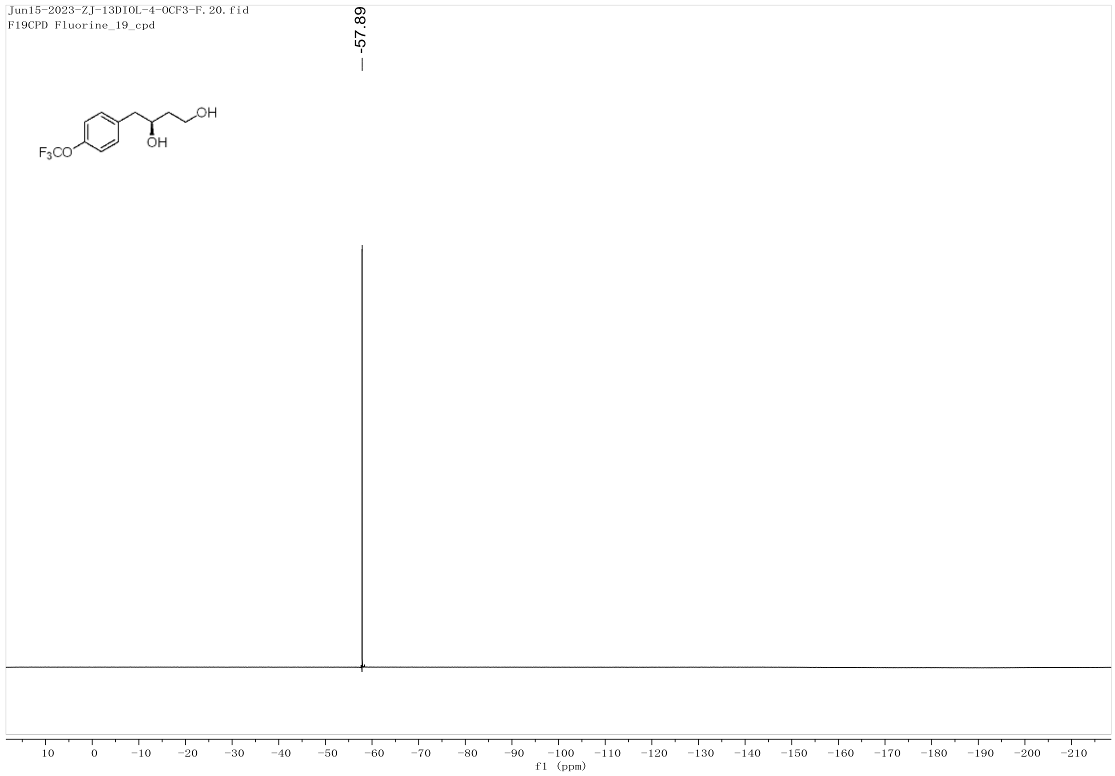
Jun15-2023-ZJ-13DIOL-4-OCF3-F.20.fid
F19CPD Fluorine_19_cpd
-57.89
F3CO
OH
OH
f1 (ppm)

(*S*)-4-(2-fluorophenyl)butane-1,3-diol (7f)

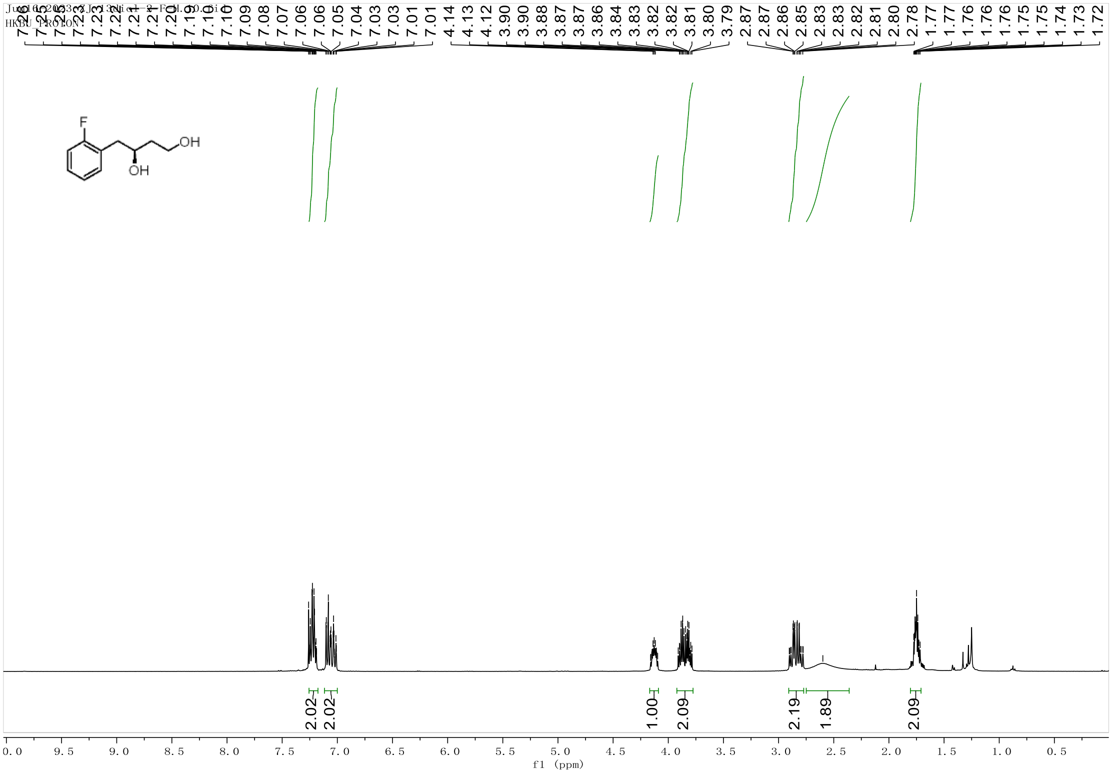

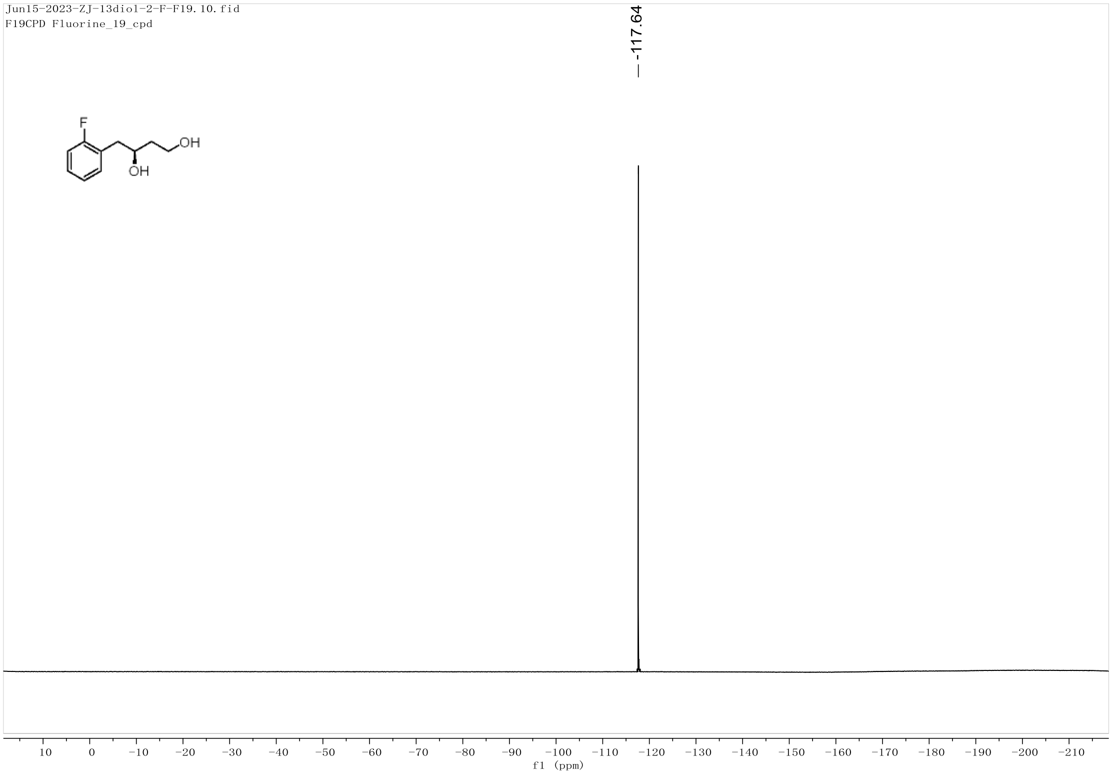

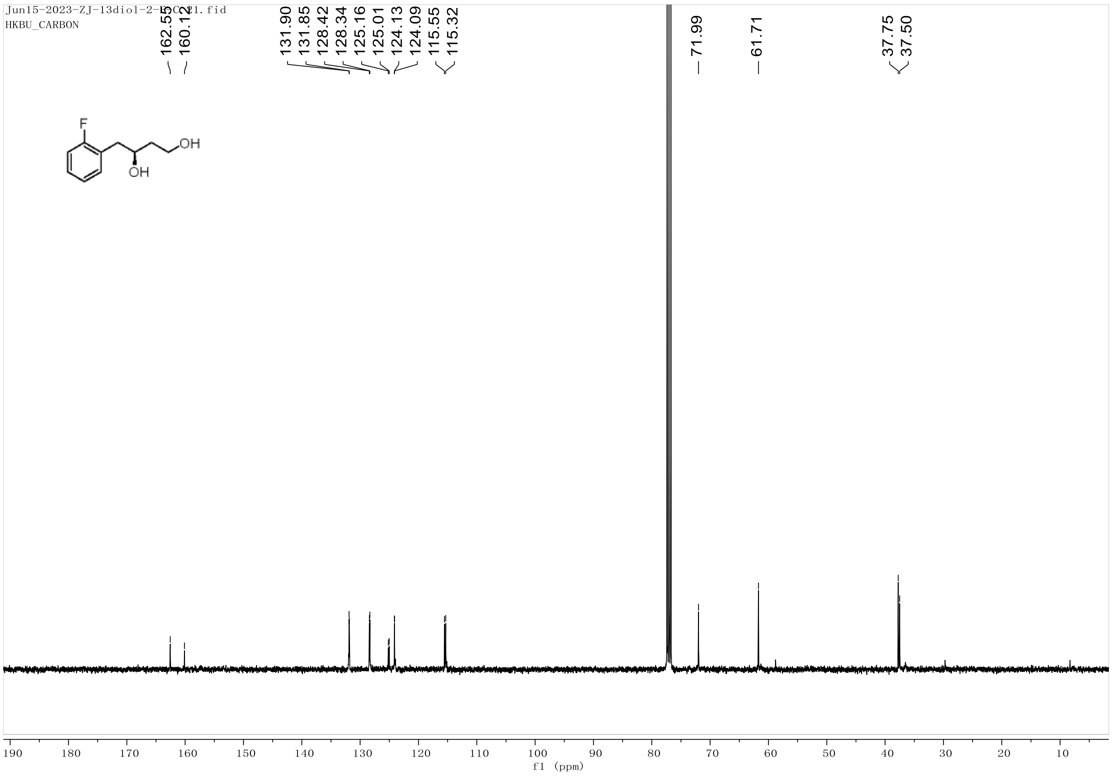

(*S*)-4-(4-fluorophenyl)butane-1,3-diol (7g)

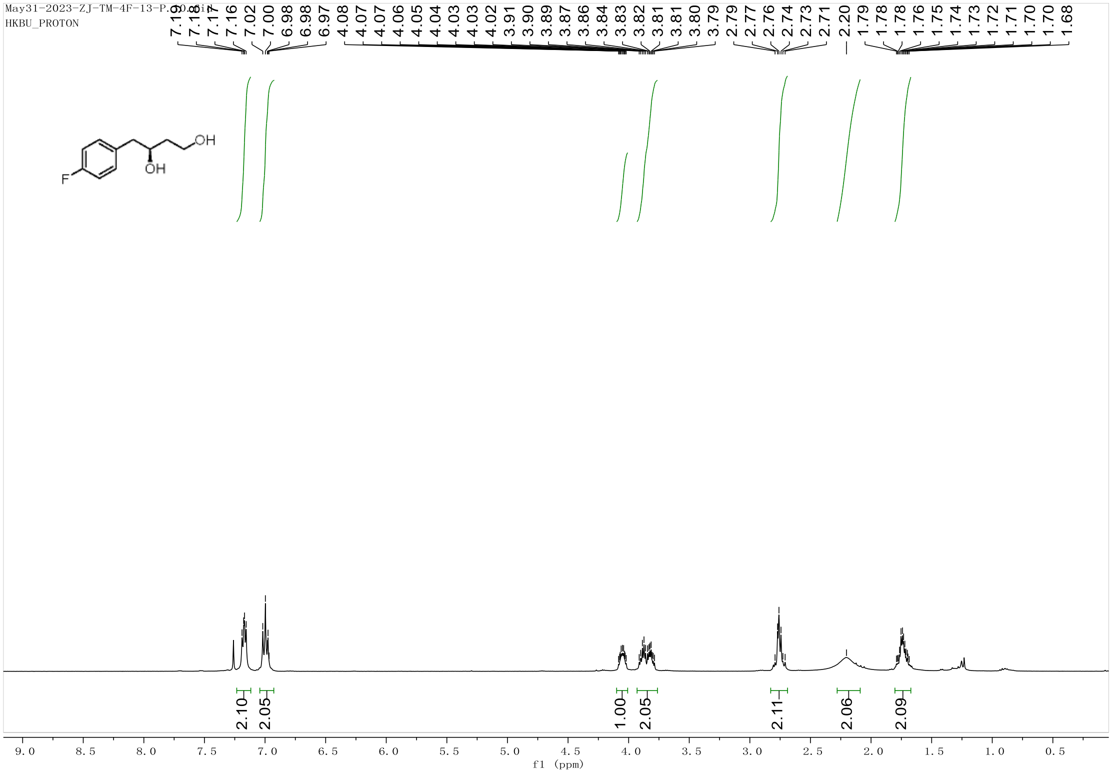

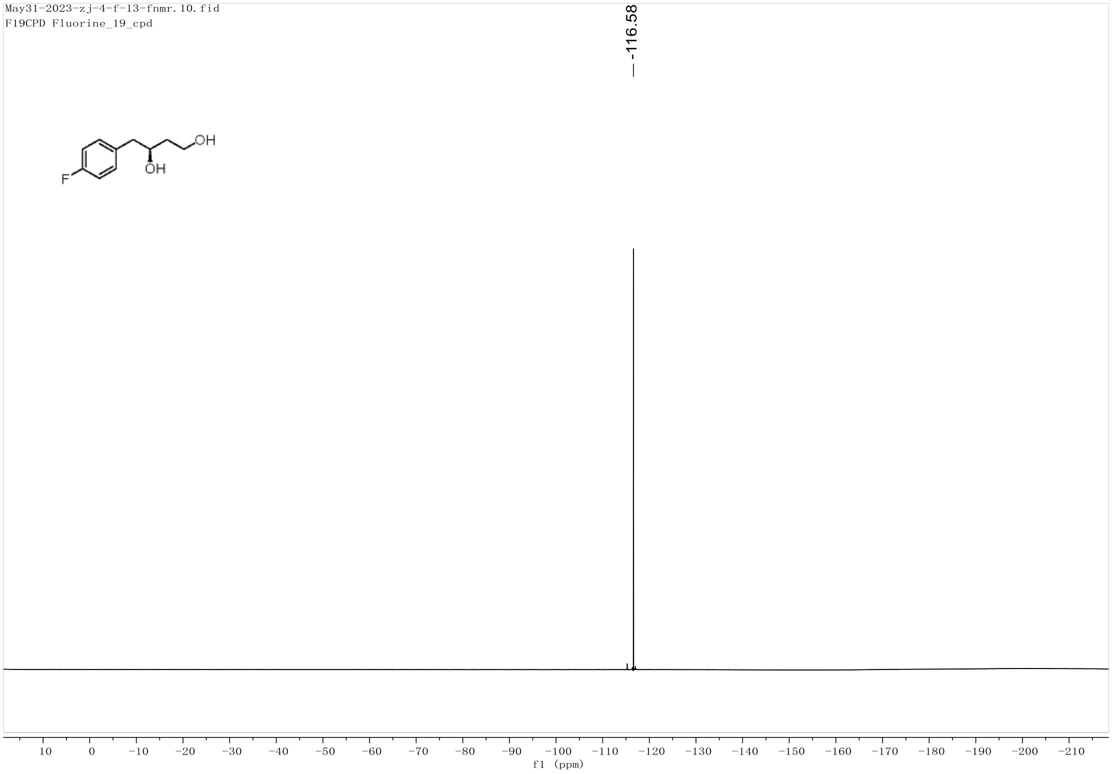
May31-2023-zj-4-f-13-fnmr.10.fid
F19CPD Fluorine_19_cpd
-116.58
OH
F
OH
10 0 −10 −20 −30 −40 −50 −60 −70 −80 −90 −100 −110 −120 −130 −140 −150 −160 −170 −180 −190 −200 −210
f1 (ppm)

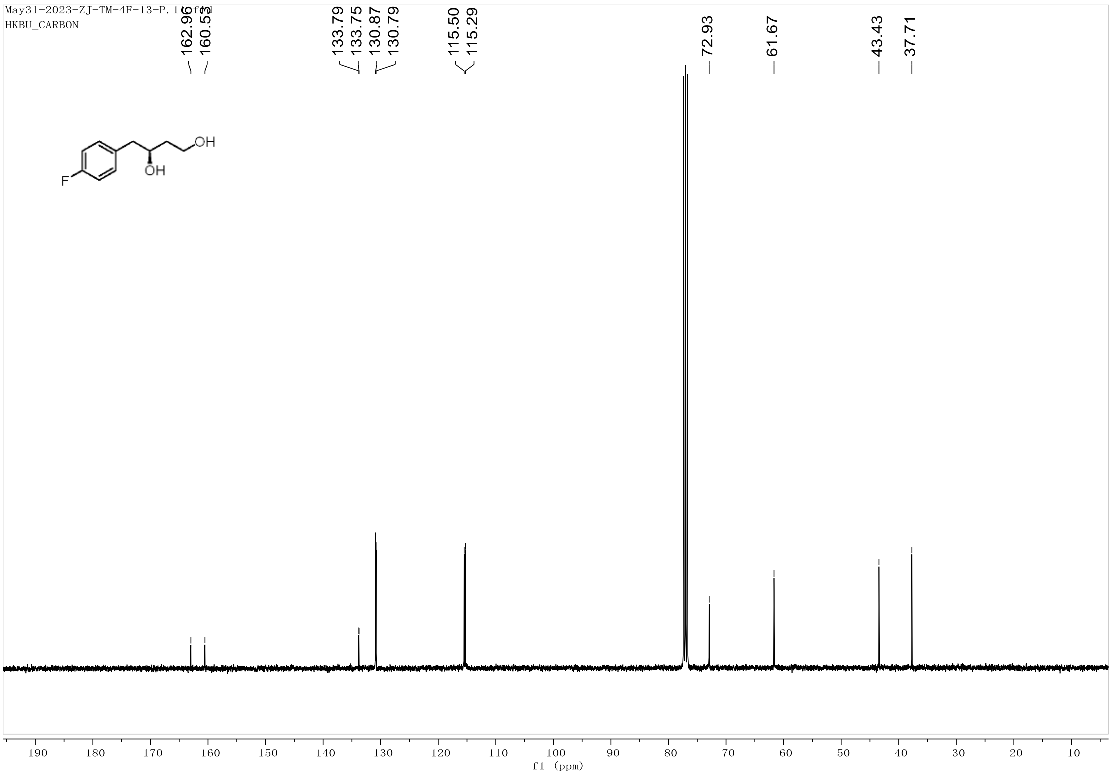
May31-2023-ZJ-TM-4F-13-P.1
HKBU_CARBON
162.96
160.53
133.79
133.75
130.87
130.79
115.50
115.29
72.93
61.67
43.43
37.71
OH
F
OH
190 180 170 160 150 140 130 120 110 100 90 80 70 60 50 40 30 20 10
f1 (ppm)

(*S*)-4-(3-bromophenyl)butane-1,3-diol (7h)

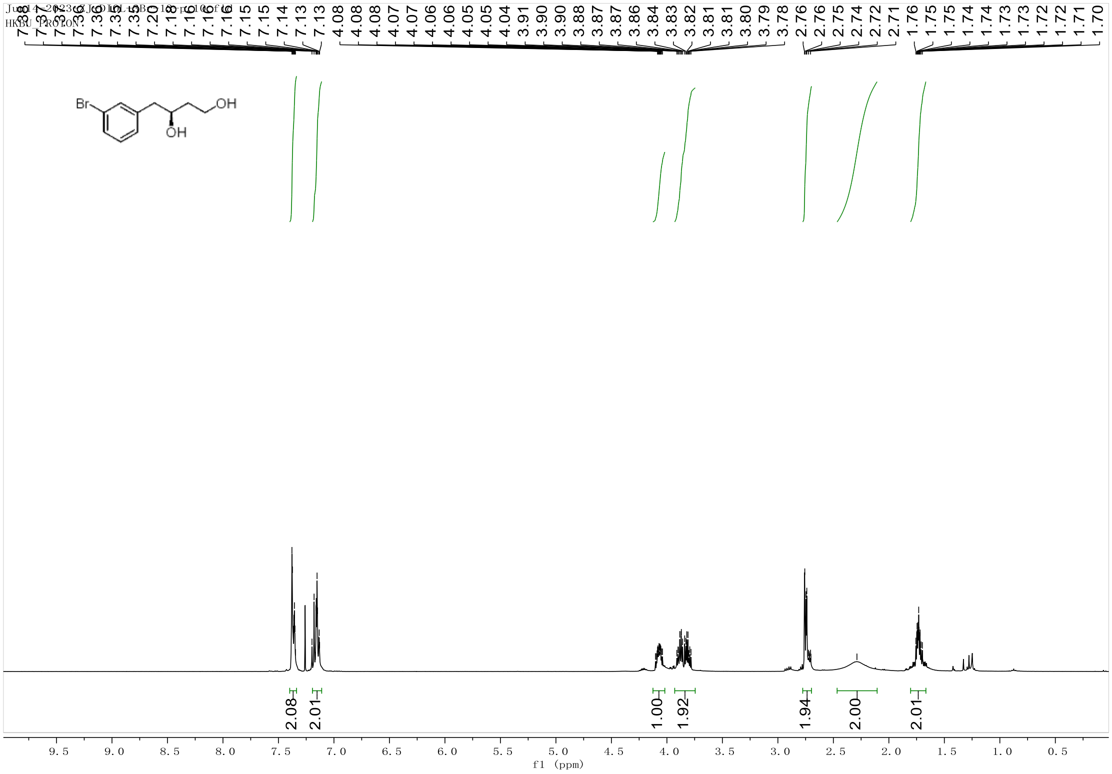

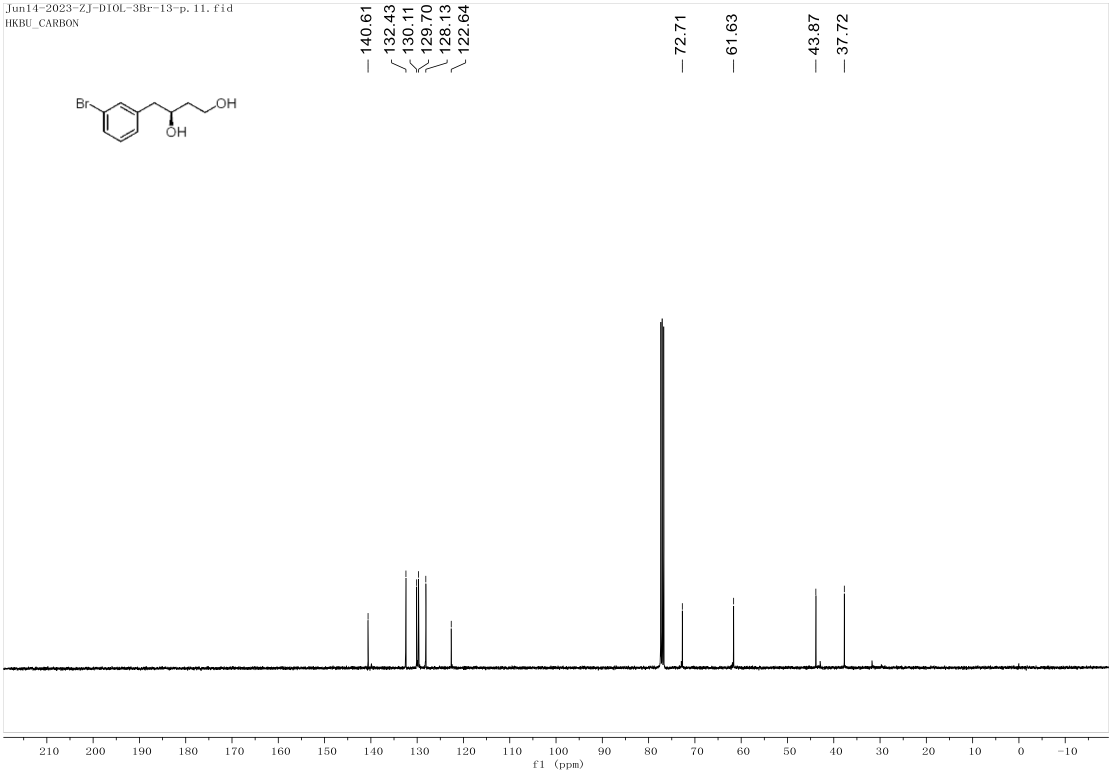

(*S*)-4-([1,1'-biphenyl]-4-yl)butane-1,3-diol (7i)

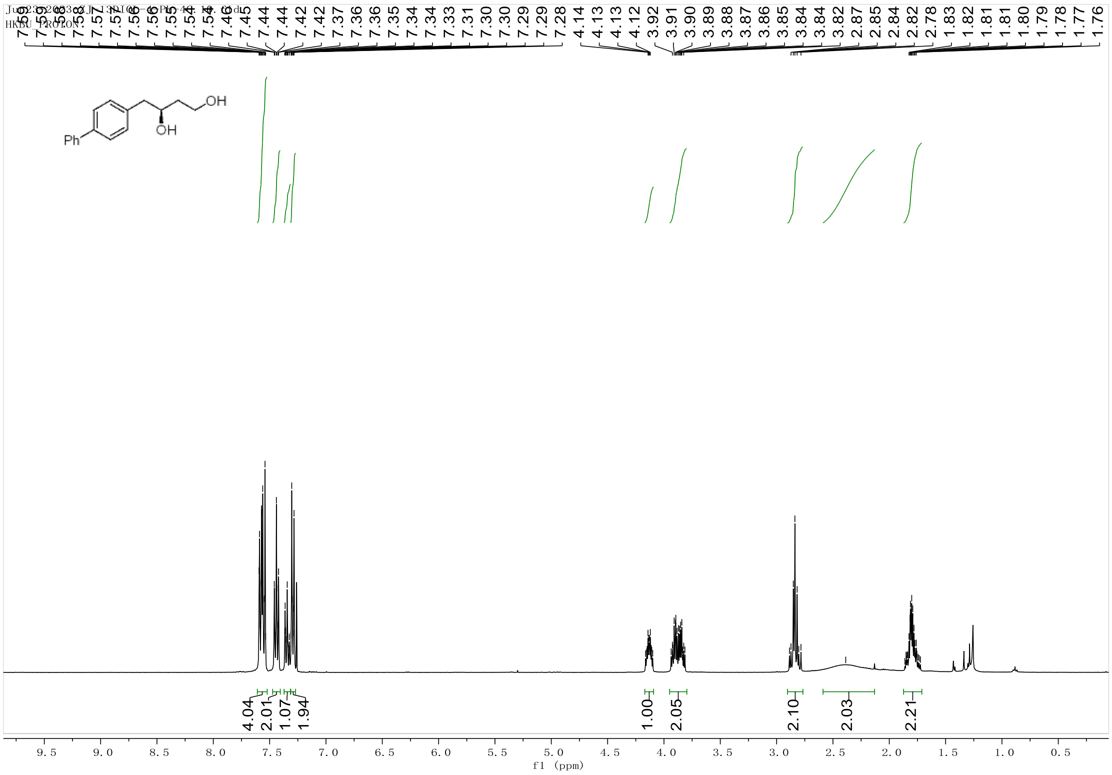

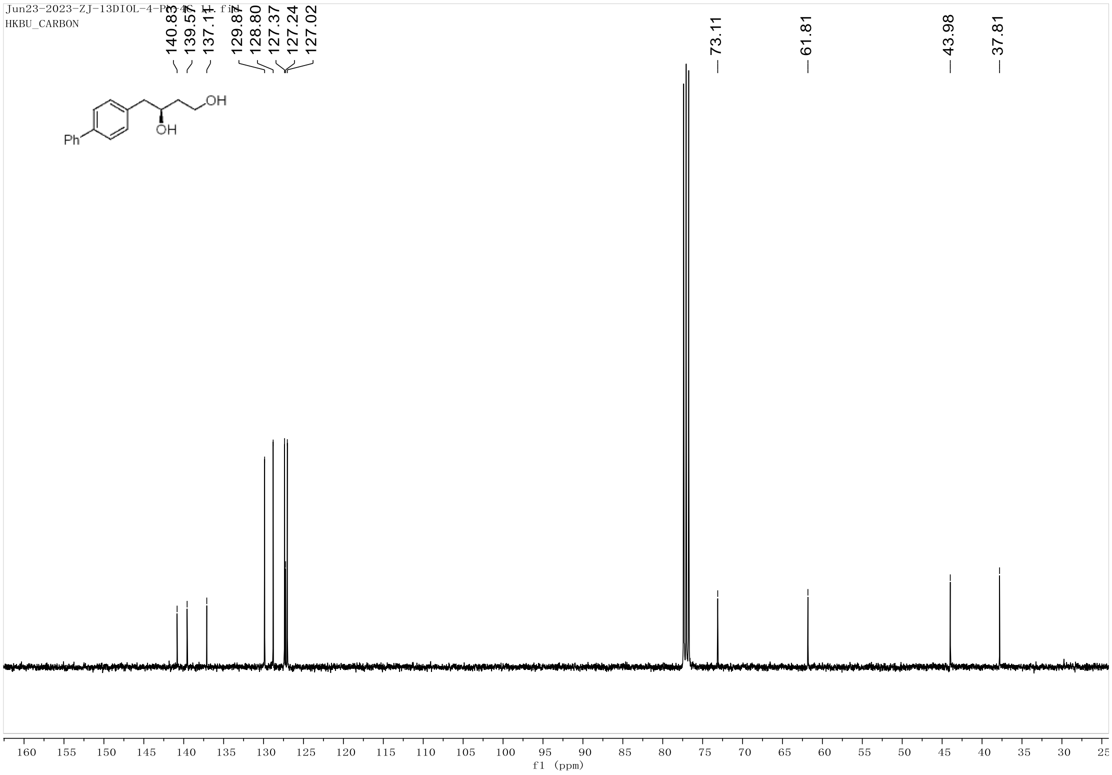

(*S*)-4-(naphthalen-1-yl)butane-1,3-diol (7j)

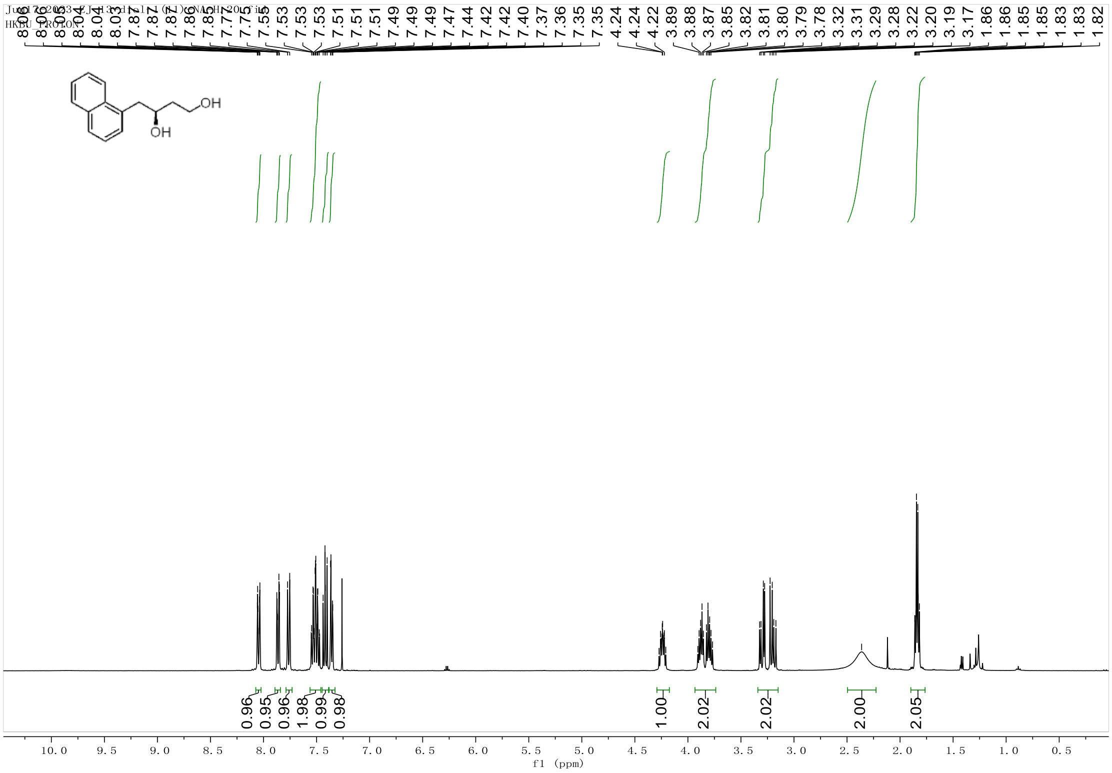

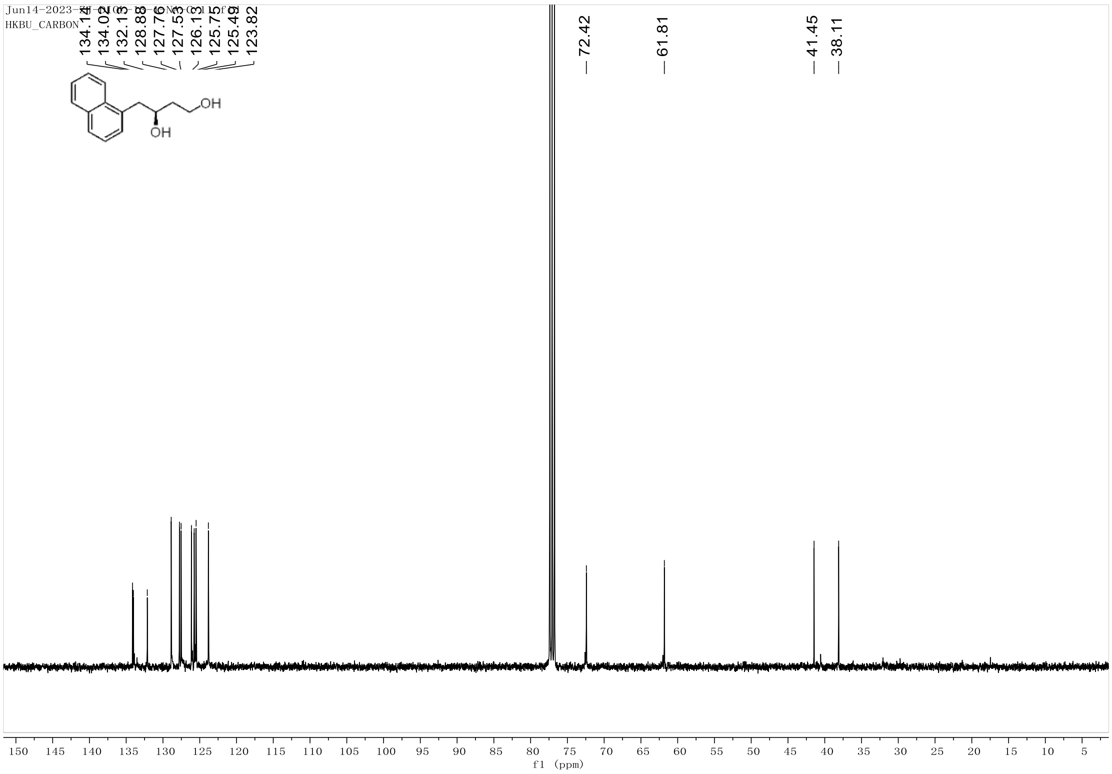

(*S*)-4-(naphthalen-2-yl)butane-1,3-diol (7k)

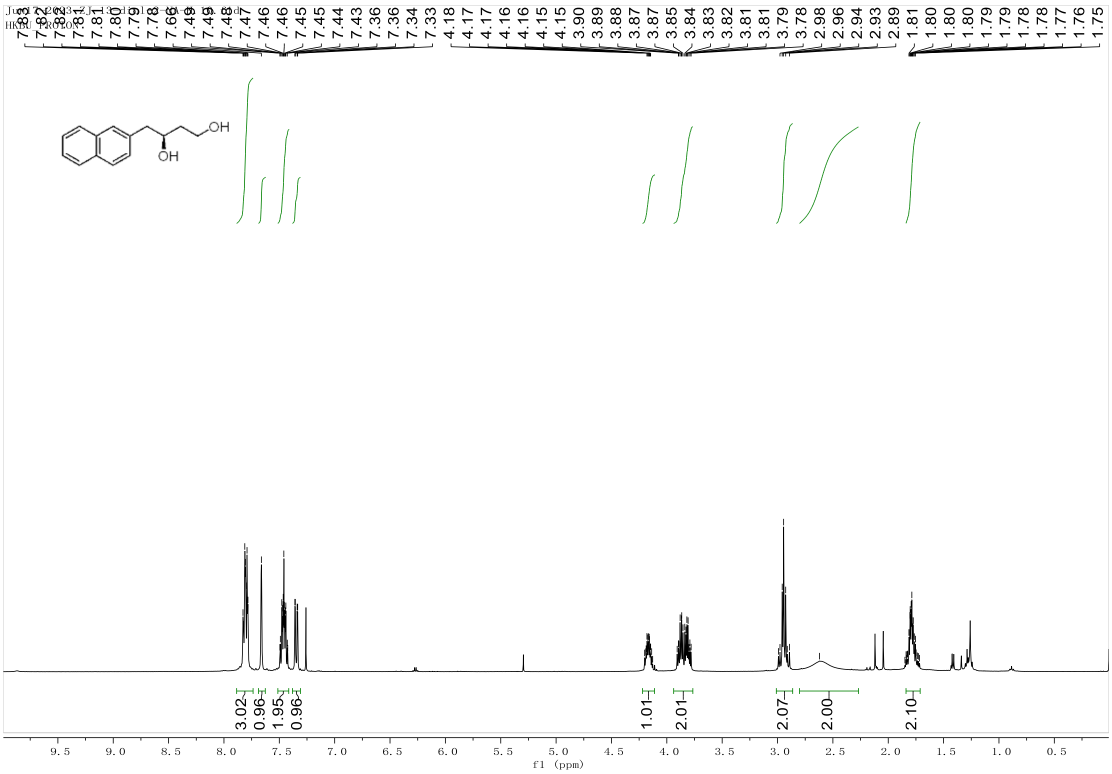

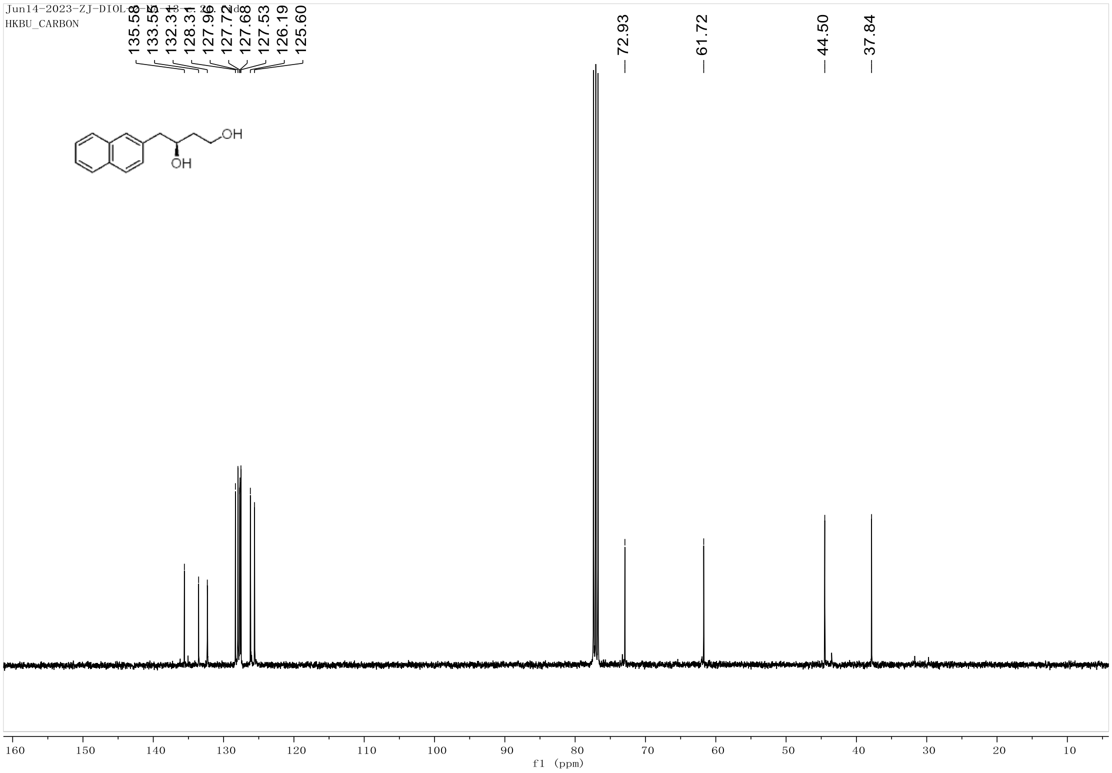

(*S*)-4-(benzofuran-3-yl)butane-1,3-diol (7l)

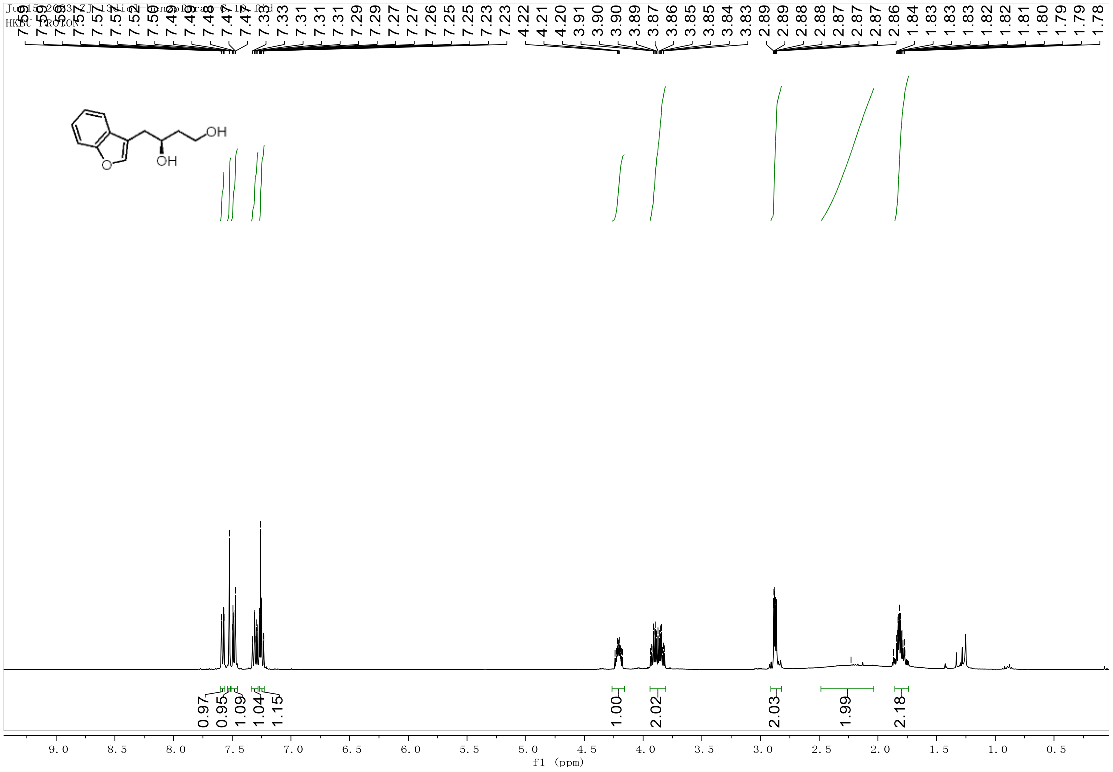

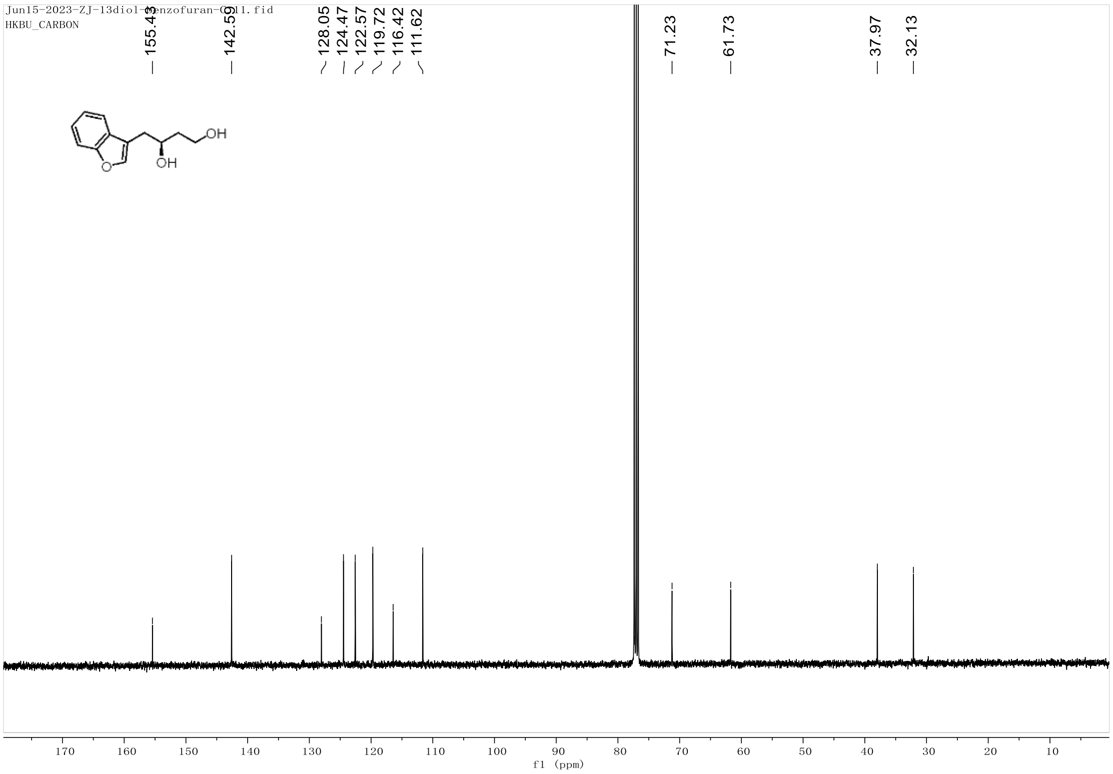

(*S*)-4-(3,4-difluorophenyl)butane-1,3-diol (7m)

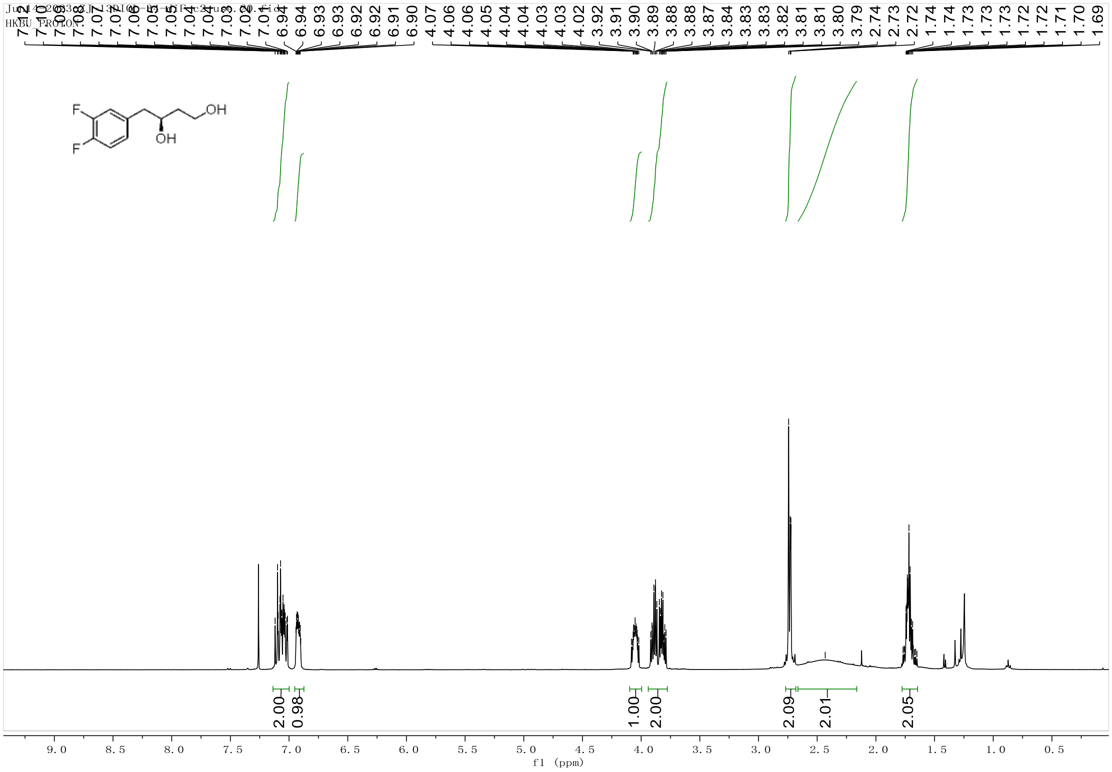

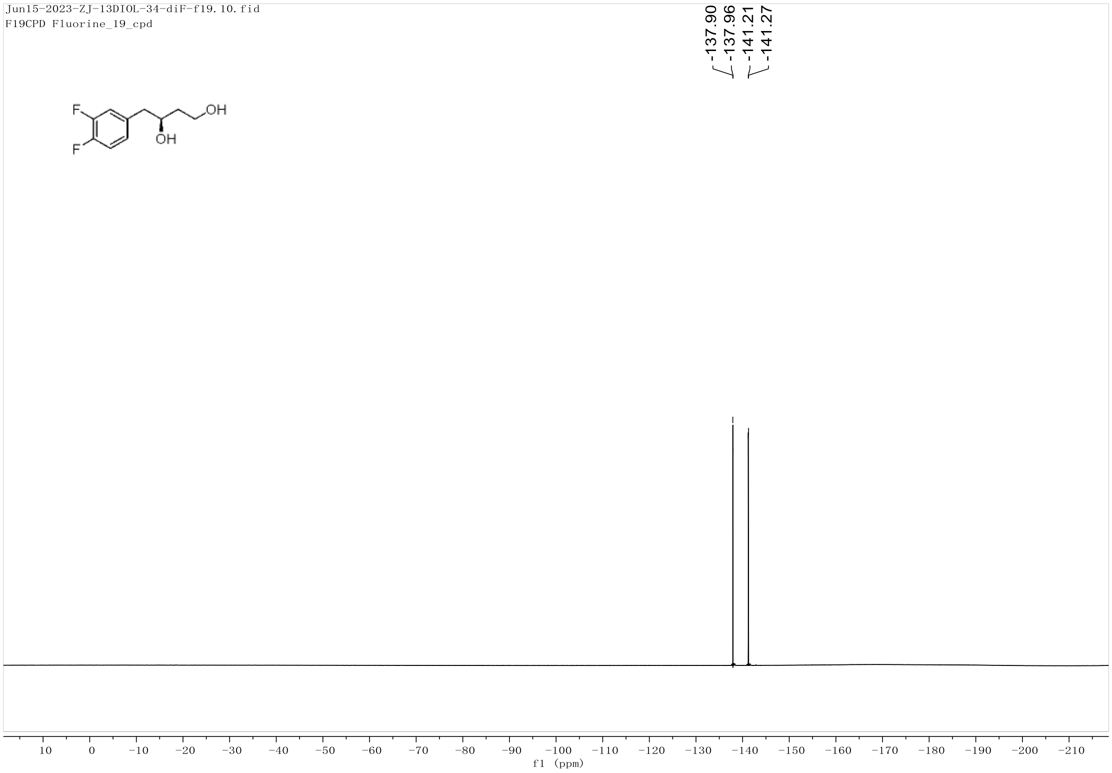

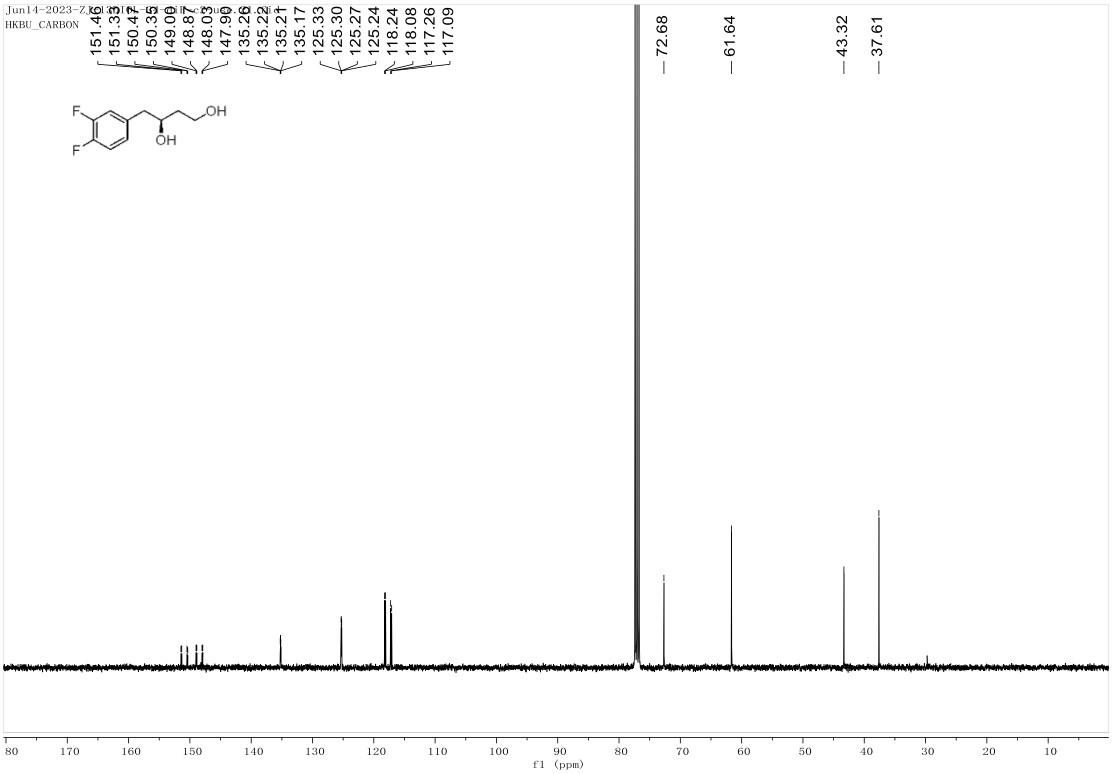

(*S*)-4-(3,4,5-trimethoxyphenyl)butane-1,3-diol (7n)

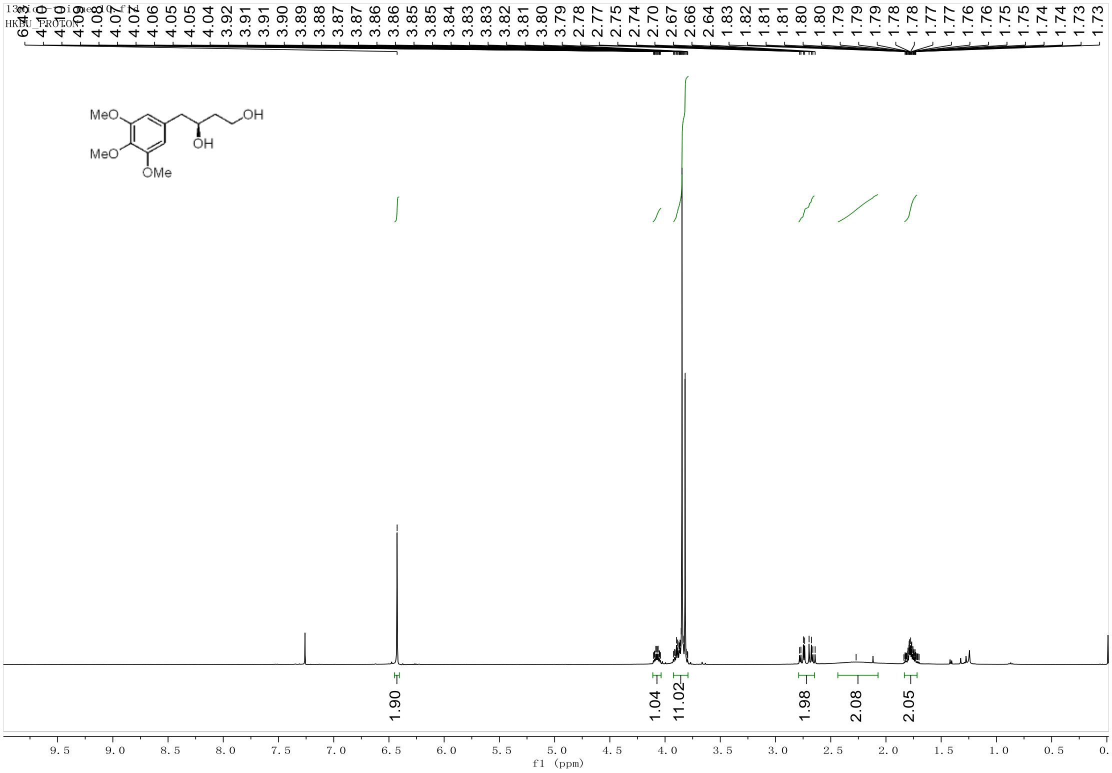

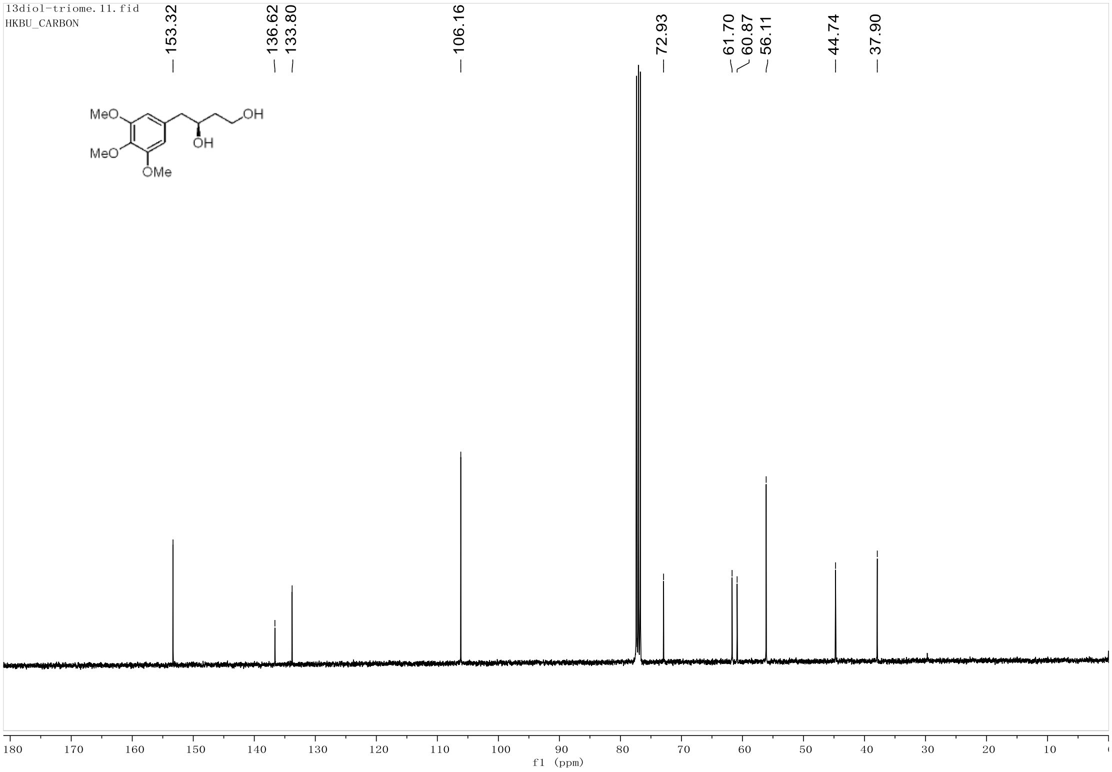

(*S*)-4-hydroxy-4-phenylbutyl diphenylphosphinate (8)

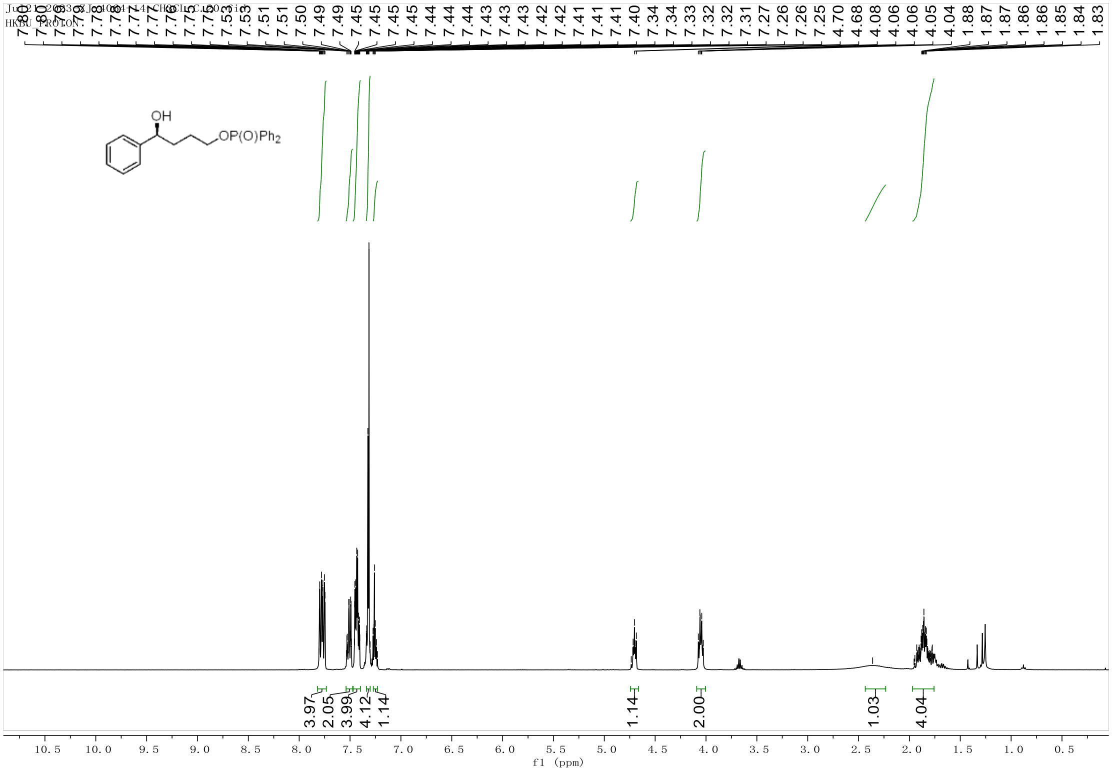

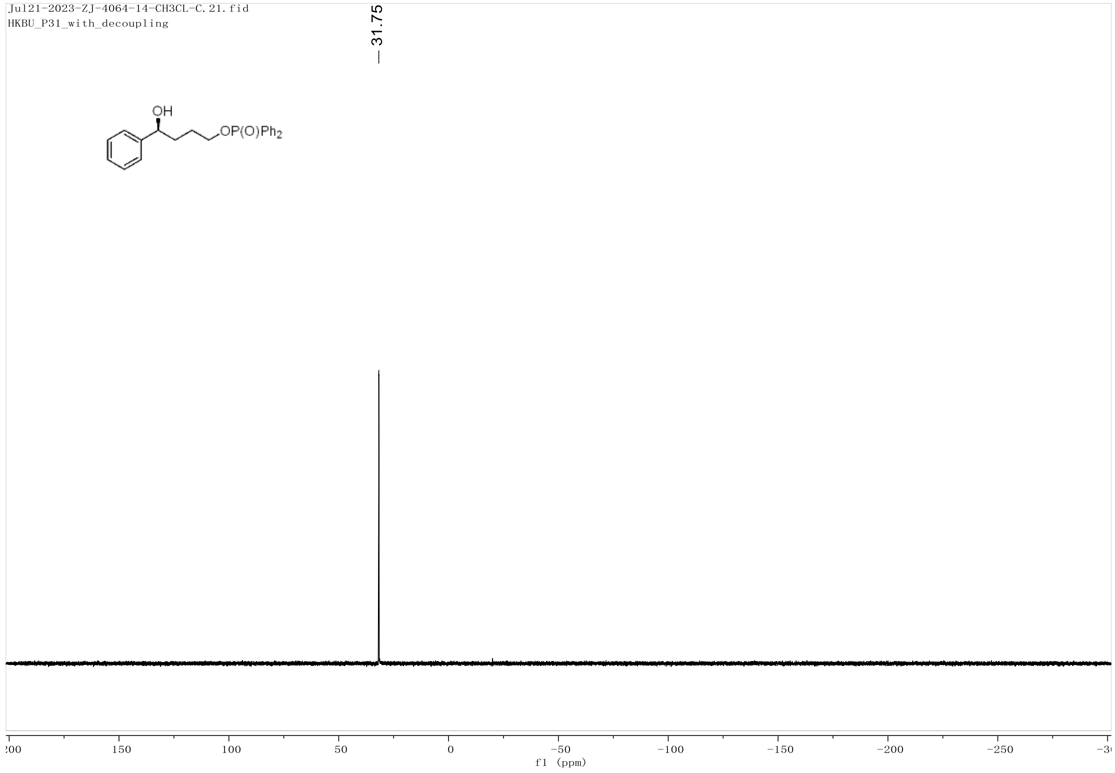
Jul21-2023-ZJ-4064-14-CH3CL-C.21.fid
HKBU_P31_with_decoupling
31.75
OH
OP(O)Ph2
200
150
100
50
0
−50
−100
−150
−200
−250
f1 (ppm)

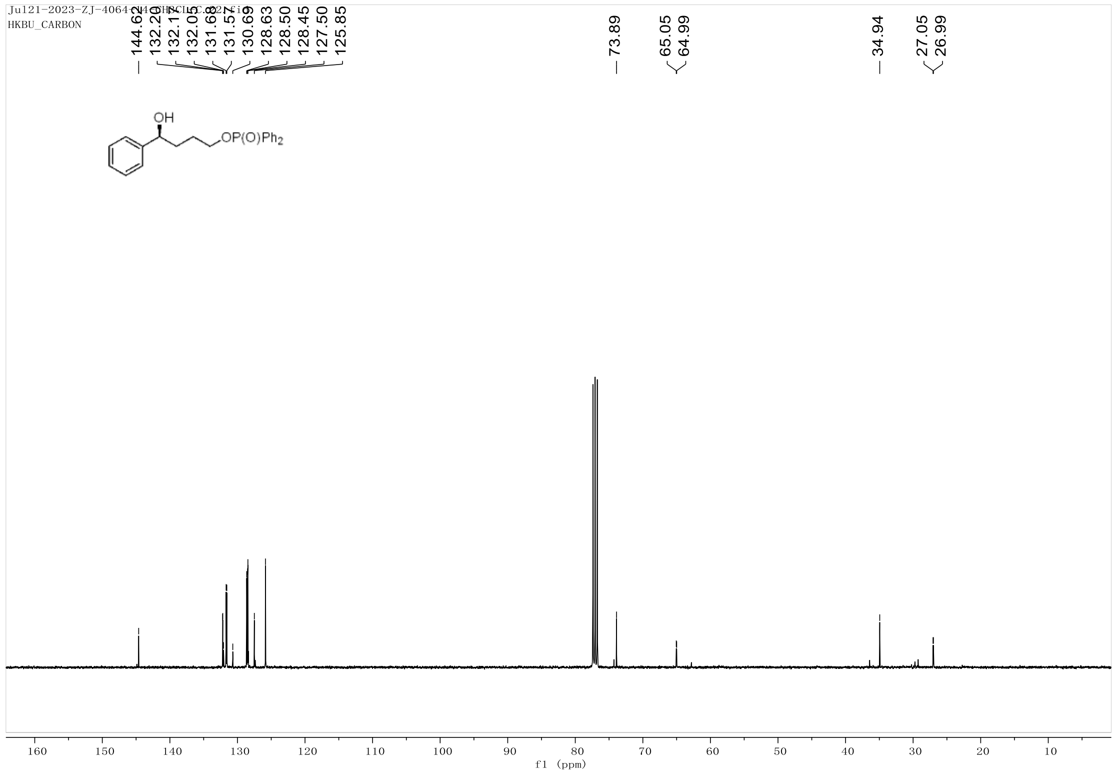
HKBU_CARBON
144.62
132.20
132.17
132.05
131.68
131.57
130.69
128.63
128.50
128.45
127.50
125.85
73.89
65.05
64.99
34.94
27.05
26.99
OH
OP(O)Ph2
160
150
140
130
120
110
100
90
80
70
60
50
40
30
20
10
f1 (ppm)

(*S*)-1-phenylbutane-1,4-diyl bis(diphenylphosphinate) (9)

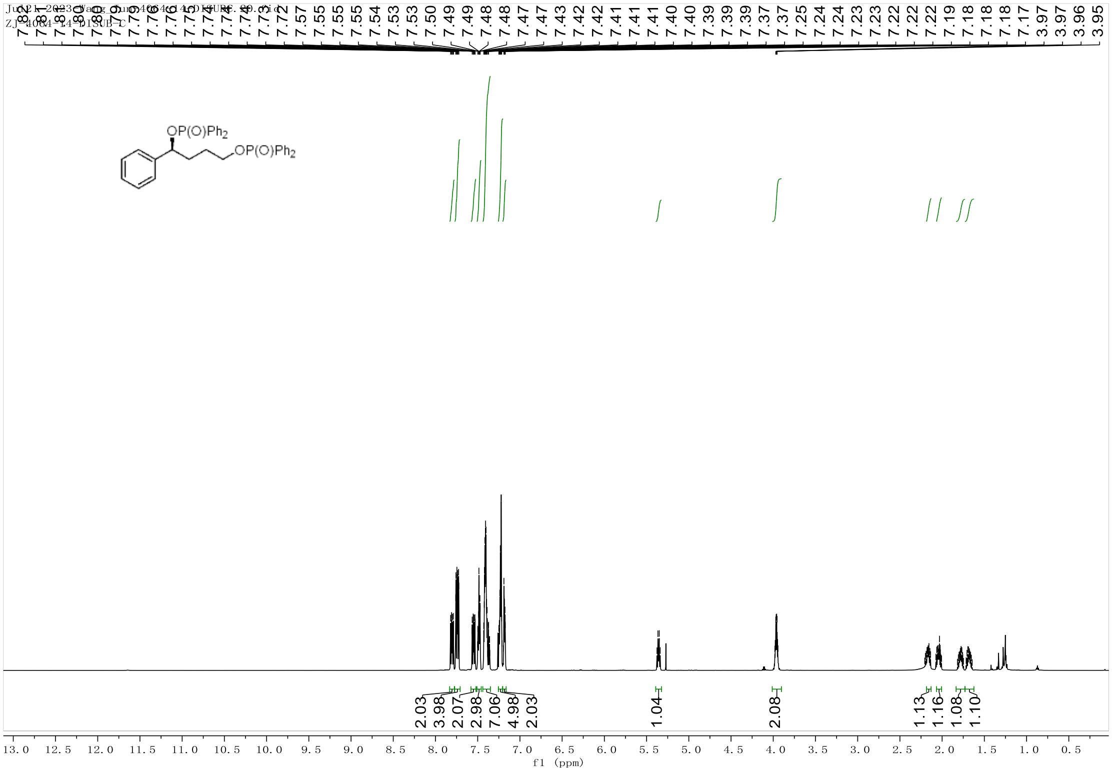

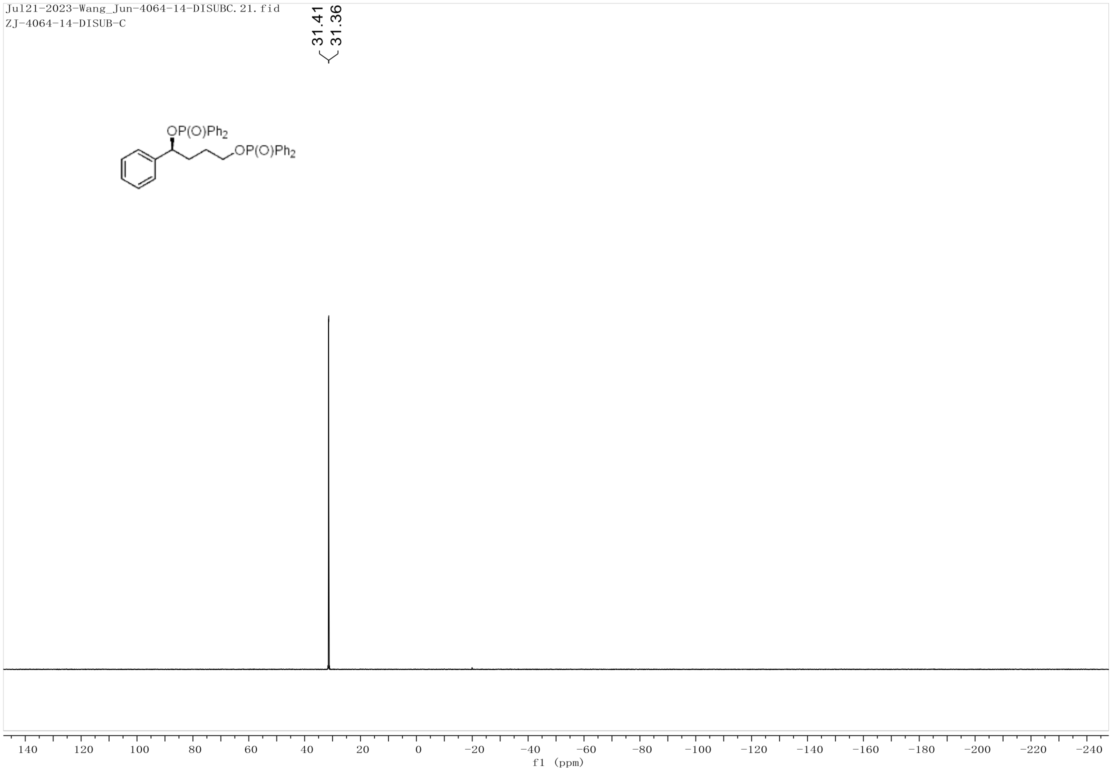

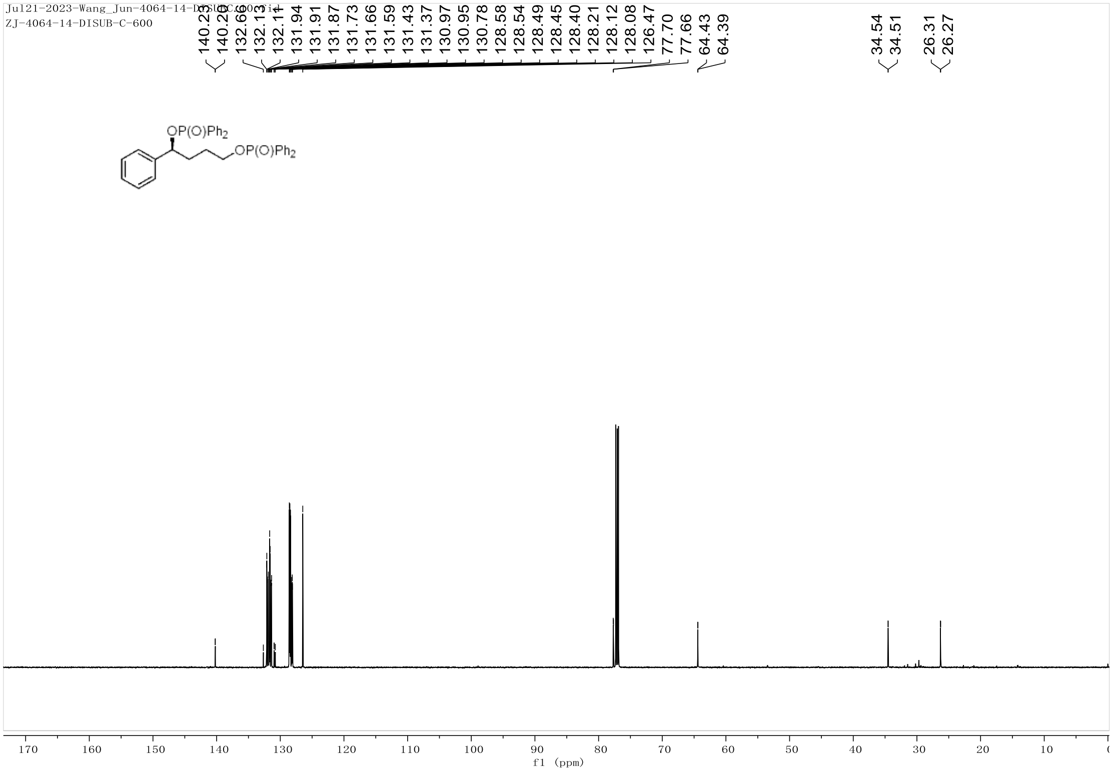

(*S*)-4-(4-methoxyphenyl)-1-phenylbutan-1-ol (10)

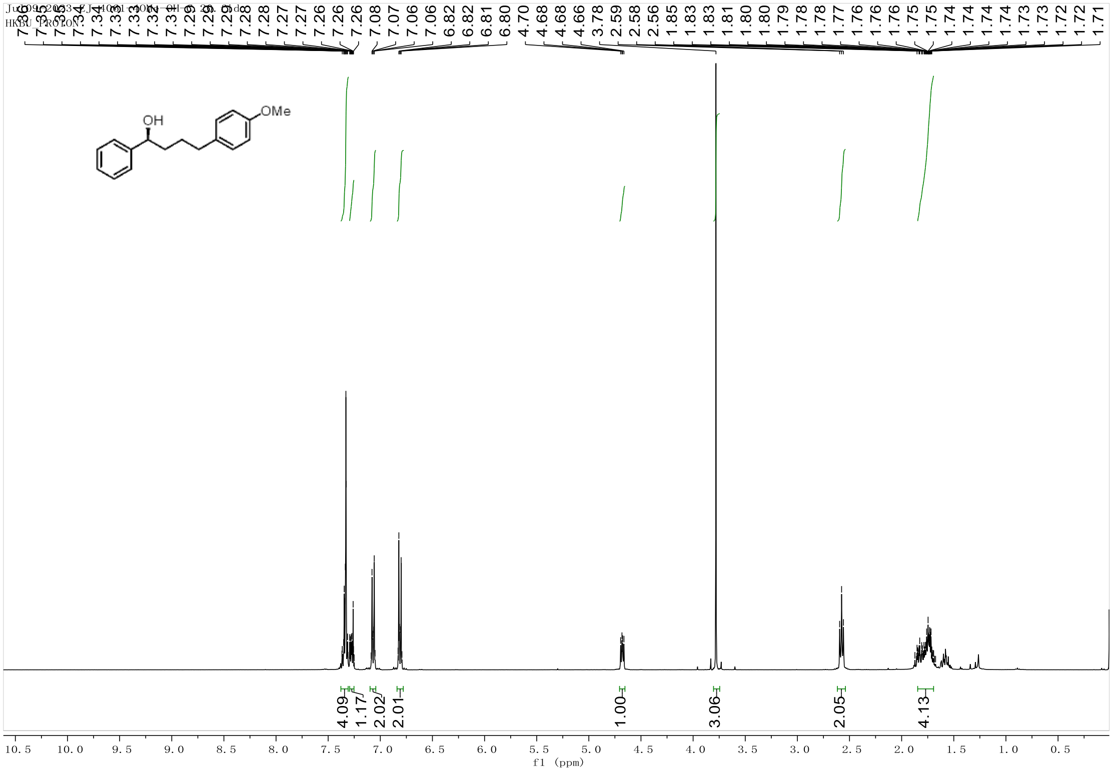

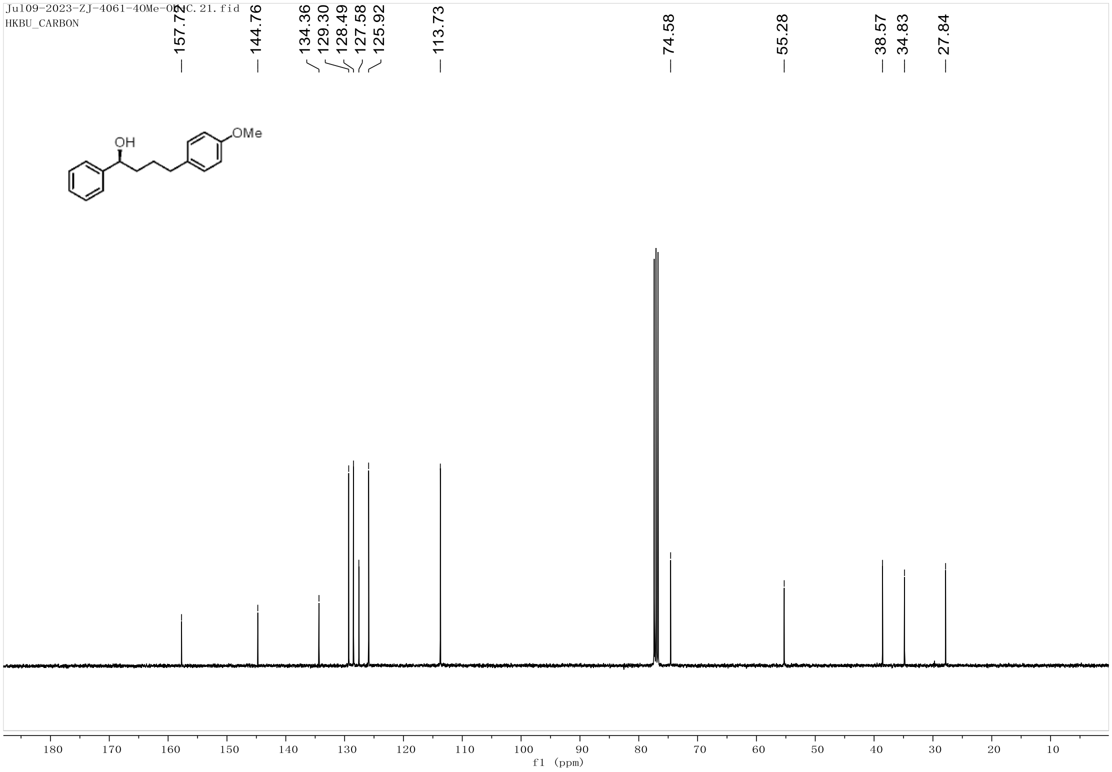

(*S*)-4-(3,5-dimethylphenyl)-1-phenylbutan-1-ol (11)

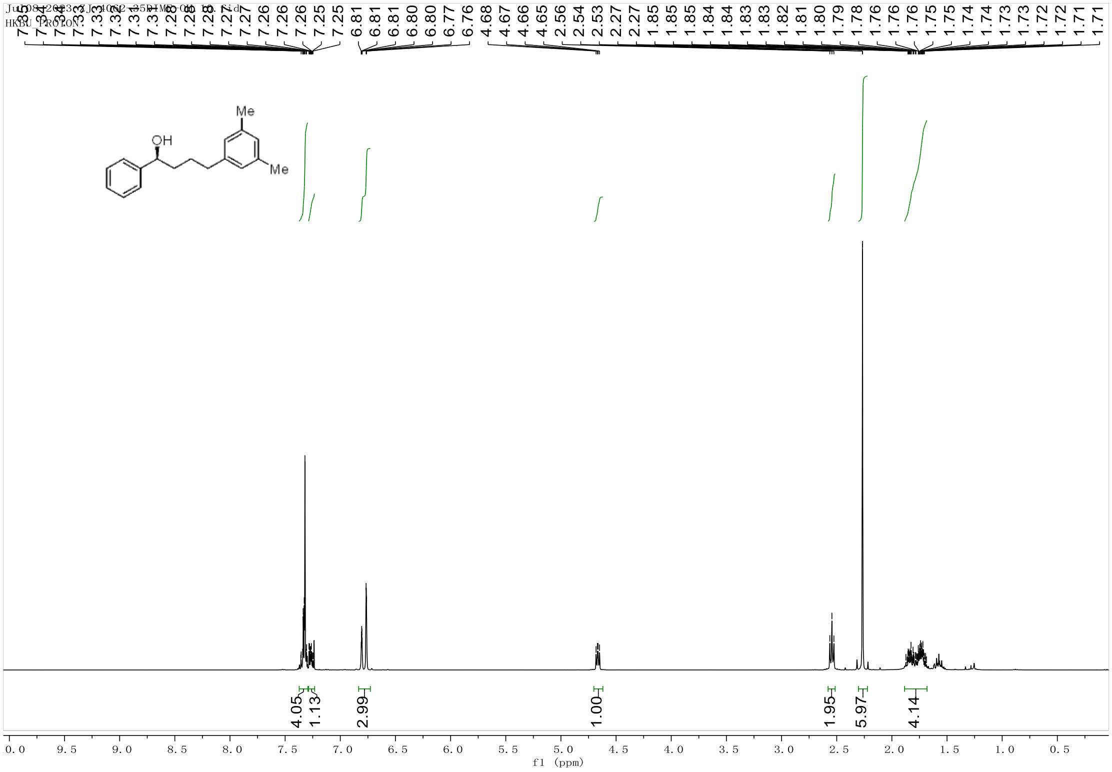

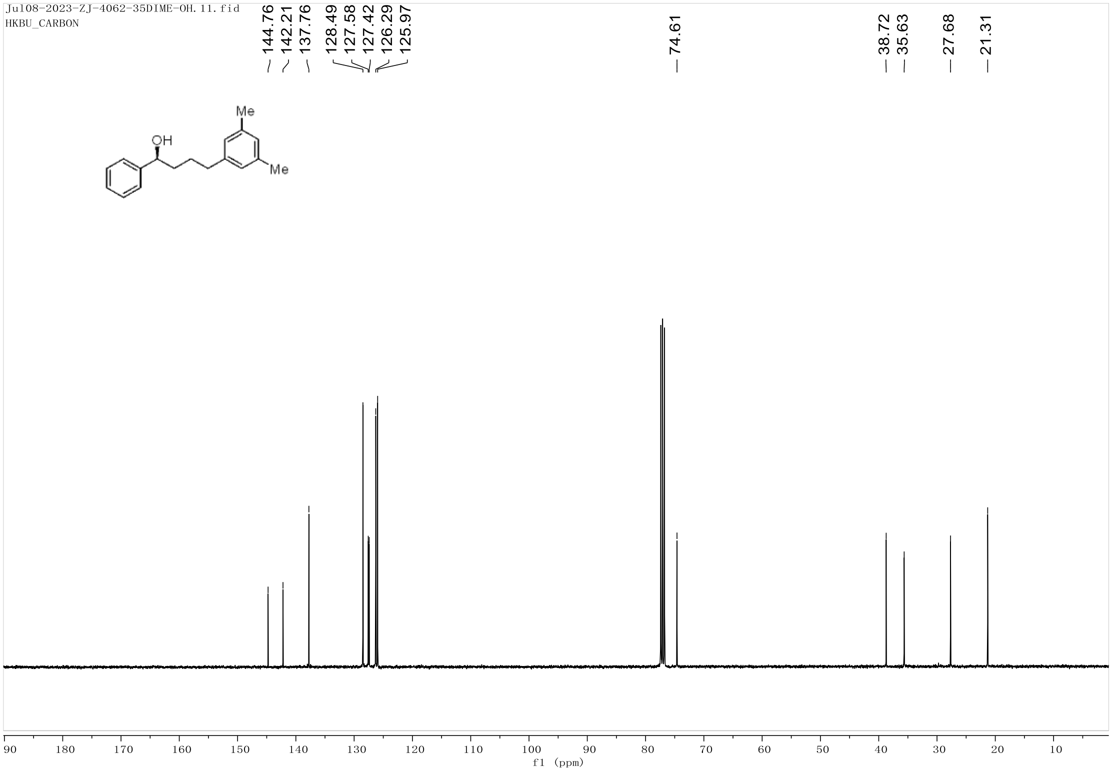

(*S*)-4-phenylbutane-1,3-diyl bis(diphenylphosphinate) (12)

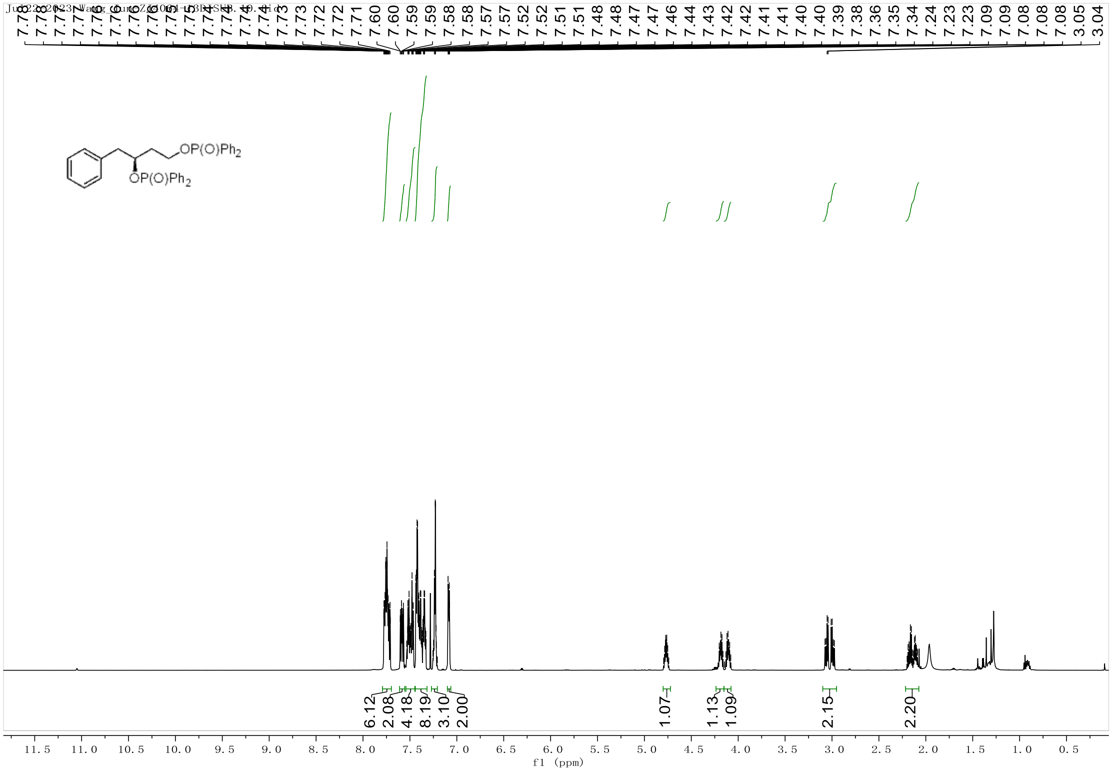

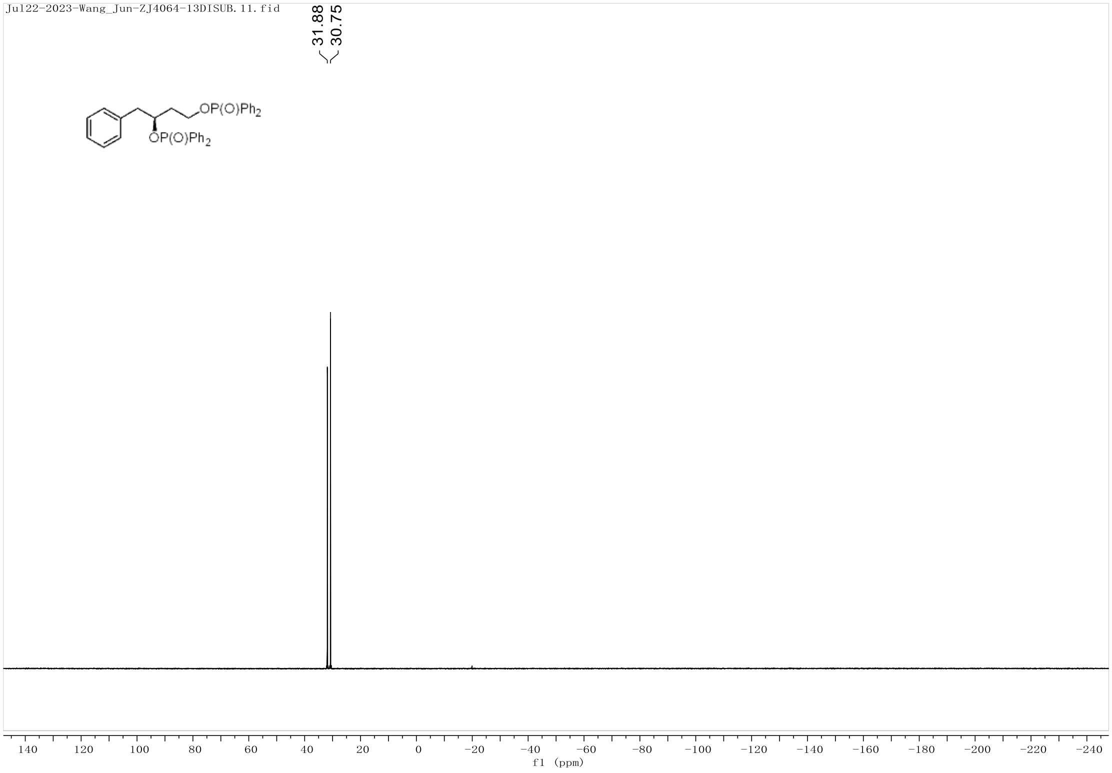
Jul22-2023-Wang_Jun-ZJ4064-13DISUB.11.fid
31.88
30.75
OP(O)Ph2
OP(O)Ph2
f1 (ppm)

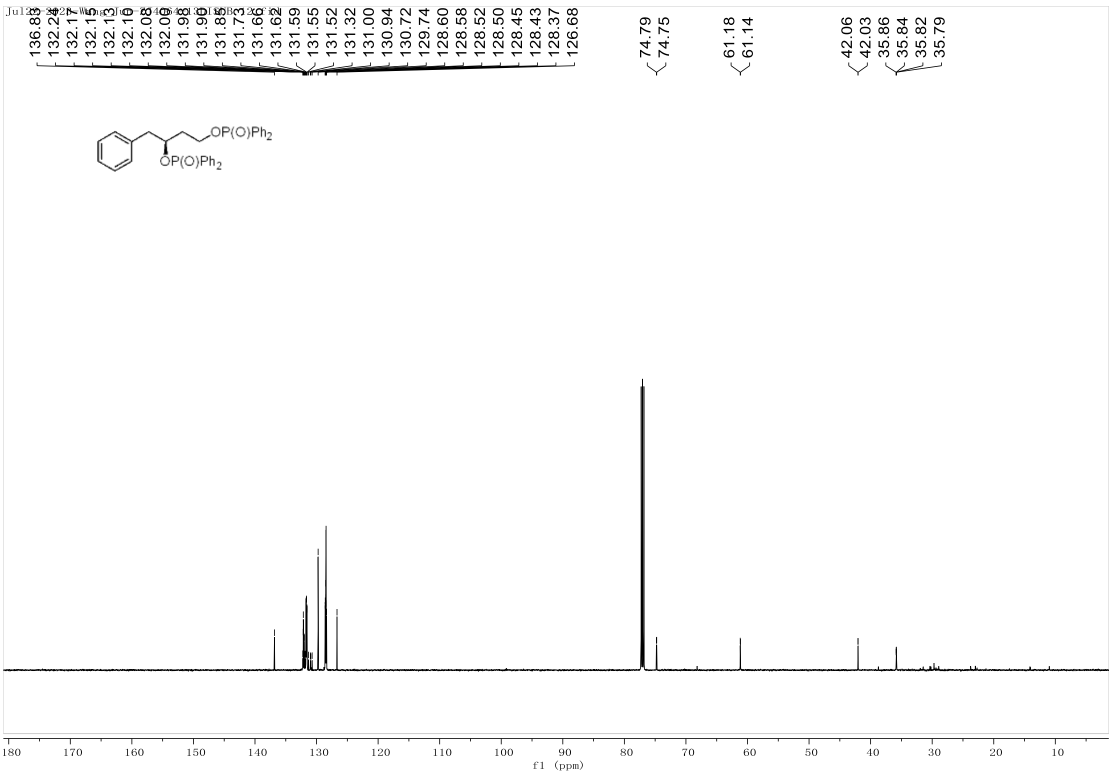
136.83
132.21
132.17
132.13
132.10
132.08
132.06
131.98
131.90
131.85
131.73
131.66
131.62
131.59
131.55
131.52
131.32
131.00
130.94
130.72
129.74
128.60
128.58
128.52
128.50
128.45
128.43
128.37
126.68
74.79
74.75
61.18
61.14
42.06
42.03
35.86
35.84
35.82
35.79
OP(O)Ph2
OP(O)Ph2
f1 (ppm)

(*R*)-4-(3,5-dimethylphenyl)-1-phenylbutan-2-ol (13)

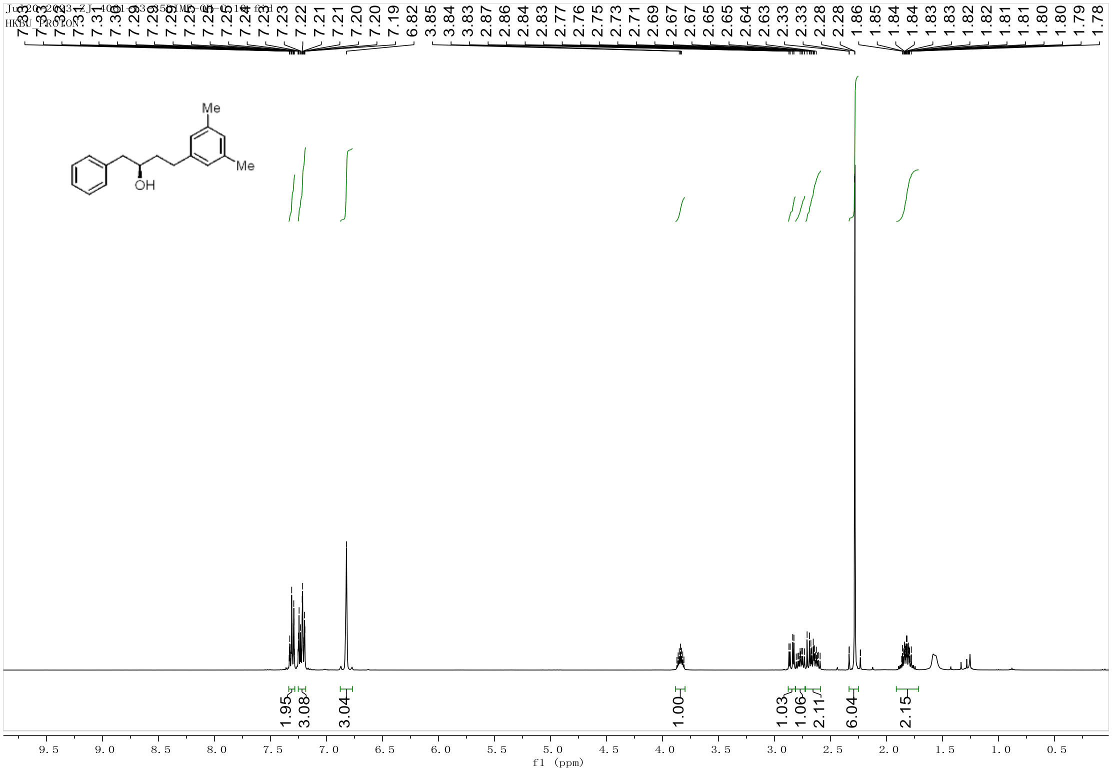

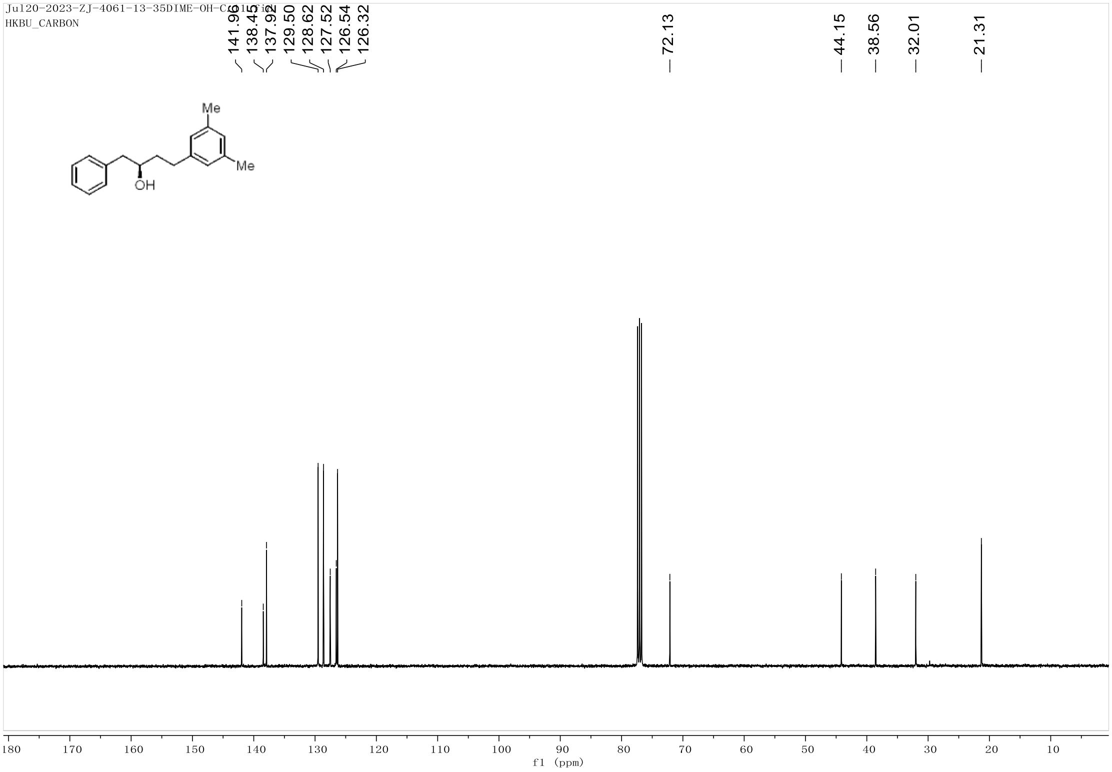

11. HPLC Spectrum

(*S*)-1-phenylbutane-1,4-diol (4a)

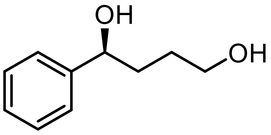

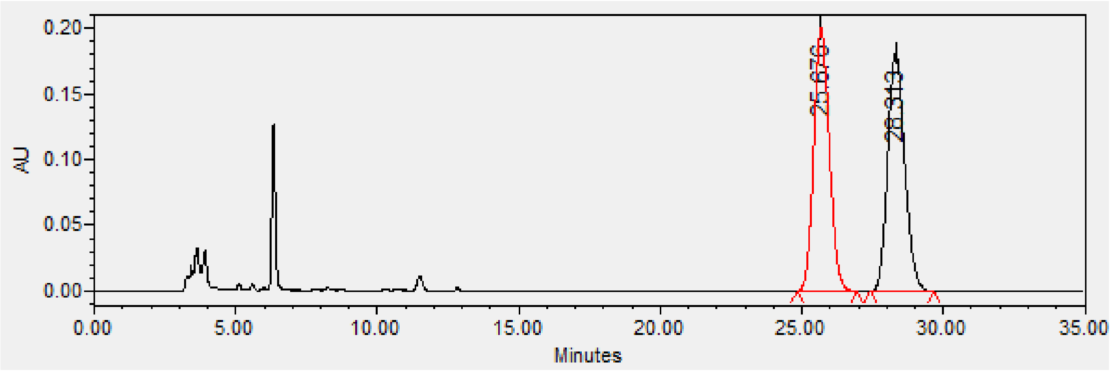

	Name	Retention Time (min)	Area (μV*sec)	% Area	Height (μV)	Int Type	Amount	Units	Peak Type	Peak Codes
1		25.676	7634169	50.11	200315	BB			Unknown	
2		28.313	7601479	49.89	181058	BB			Unknown	

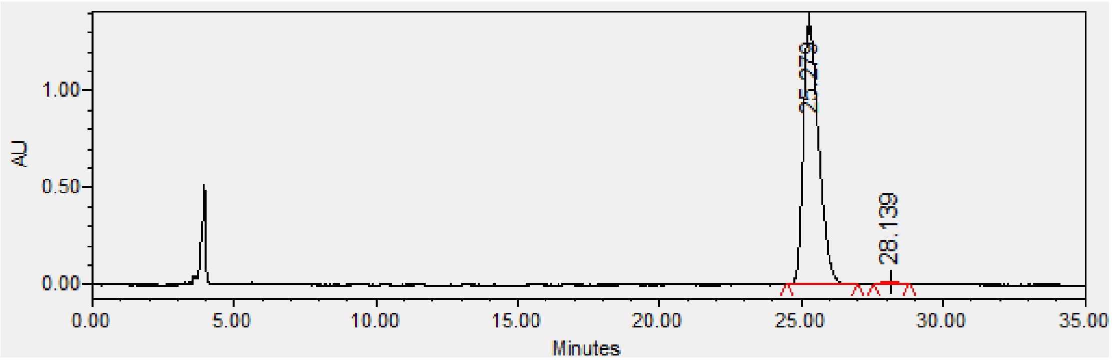

	Name	Retention Time (min)	Area (μV*sec)	% Area	Height (μV)	Int Type	Amount	Units	Peak Type	Peak Codes
1		25.279	51769190	98.93	1340830	Bb			Unknown	
2		28.139	560221	1.07	15308	bb			Unknown	

(*S*)-1-(o-tolyl)butane-1,4-diol (4b)

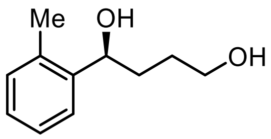

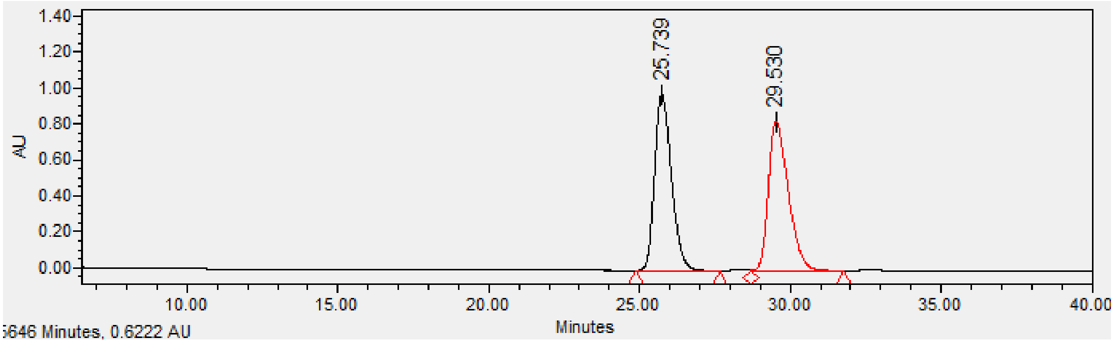

	Name	Retention Time (min)	Area (µV*sec)	% Area	Height (µV)	Int Type	Amount	Units	Peak Type	Peak Codes
1		25.739	38037507	50.05	982572	BB			Unknown	
2		29.530	37963558	49.95	828939	VB			Unknown	

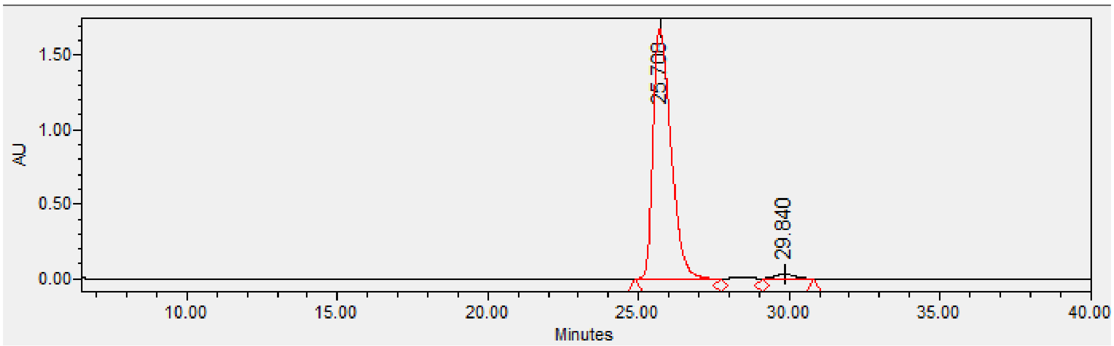

	Name	Retention Time (min)	Area (µV*sec)	% Area	Height (µV)	Int Type	Amount	Units	Peak Type	Peak Codes
1		25.708	69244631	98.13	1677469	BV			Unknown	
2		29.840	1319927	1.87	30548	VB			Unknown	

(*S*)-1-(m-tolyl)butane-1,4-diol (4c)

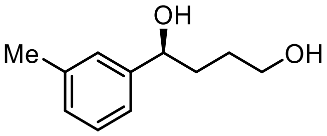

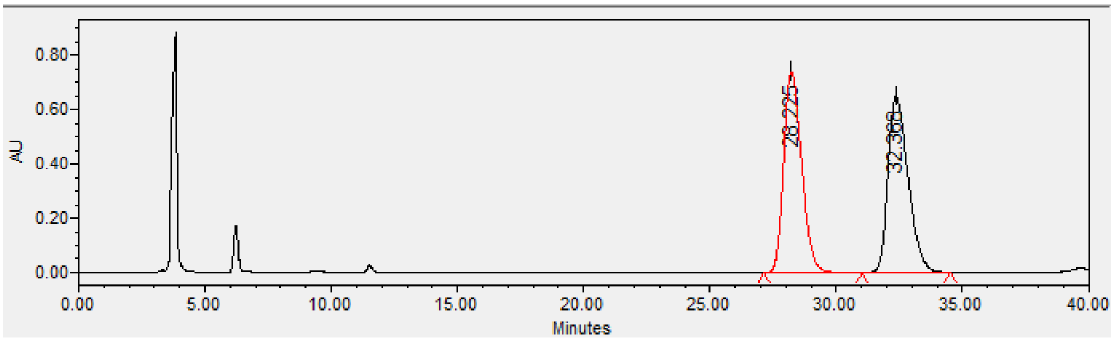

	Name	Retention Time (min)	Area (µV*sec)	% Area	Height (µV)	Int Type	Amount	Units	Peak Type	Peak Codes
1		28.225	36286133	50.22	737449	BB			Unknown	
2		32.368	35967334	49.78	647467	BB			Unknown	

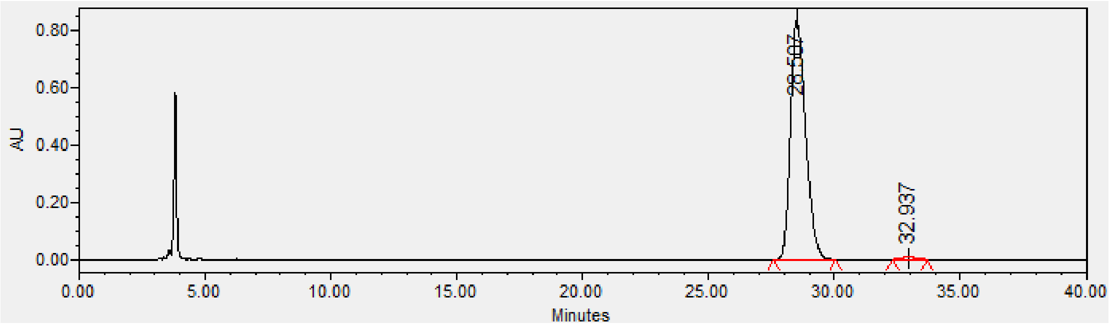

	Name	Retention Time (min)	Area (µV*sec)	% Area	Height (µV)	Int Type	Amount	Units	Peak Type	Peak Codes
1		28.507	34829099	99.09	834600	BB			Unknown	
2		32.937	318423	0.91	7815	bb			Unknown	

(*S*)-1-(p-tolyl)butane-1,4-diol (4d)

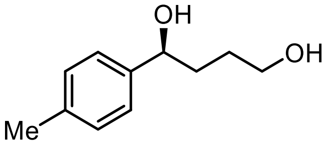

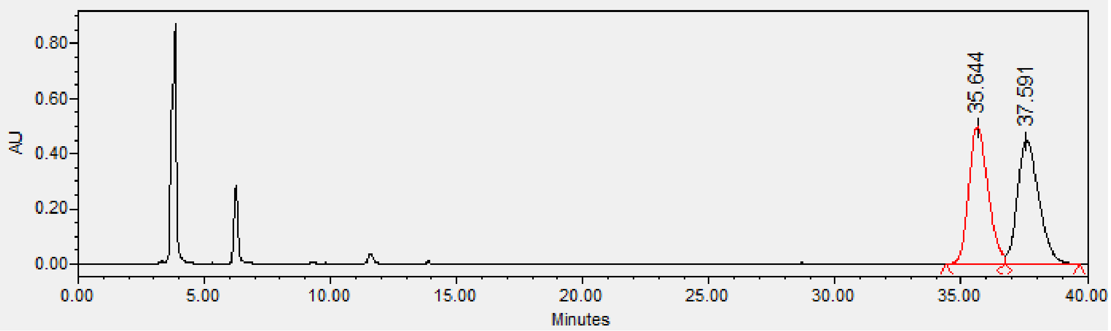

	Name	Retention Time (min)	Area (µV*sec)	% Area	Height (µV)	Int Type	Amount	Units	Peak Type	Peak Codes
1		35.644	26442024	50.03	497196	BV			Unknown	
2		37.591	26406454	49.97	447011	VB			Unknown	

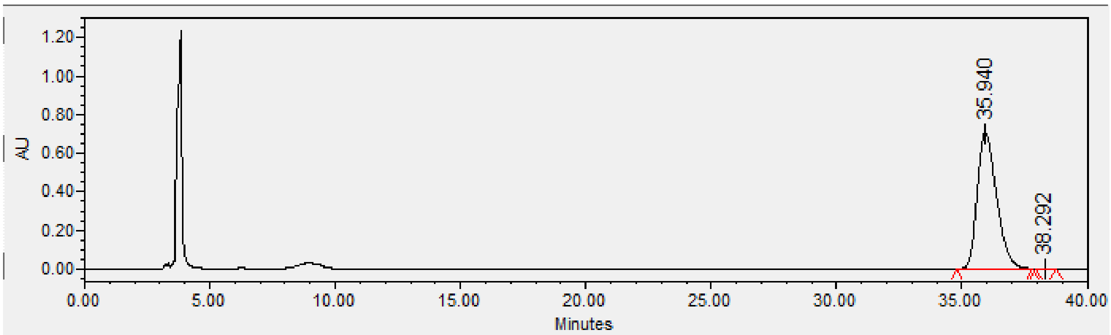

	Name	Retention Time (min)	Area (µV*sec)	% Area	Height (µV)	Int Type	Amount	Units	Peak Type	Peak Codes
1		35.940	37746486	99.95	705144	BB			Unknown	
2		38.292	18204	0.05	831	bb			Unknown	

(*S*)-1-(4-(tert-butyl)phenyl)butane-1,4-diol (4e)

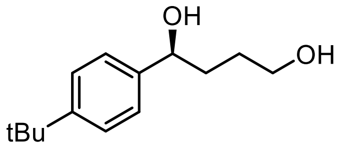

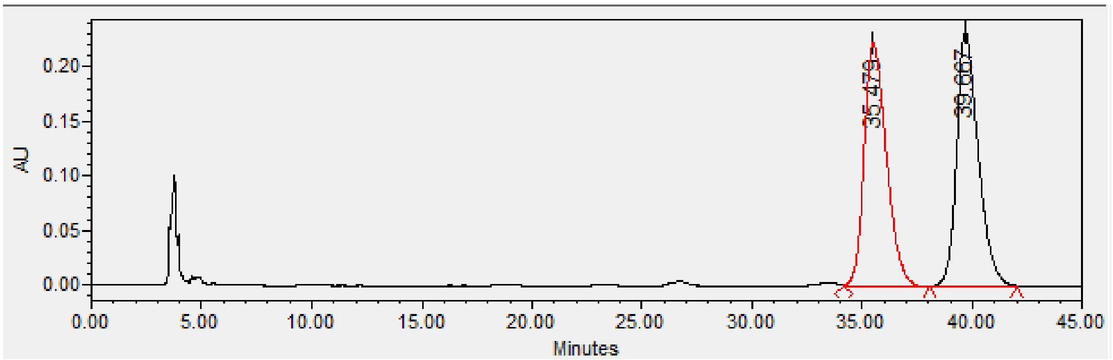

	Name	Retention Time (min)	Area (µV*sec)	% Area	Height (µV)	Int Type	Amount	Units	Peak Type	Peak Codes
1		35.479	15718323	49.72	223489	VB			Unknown	
2		39.667	15897742	50.28	233216	BB			Unknown	

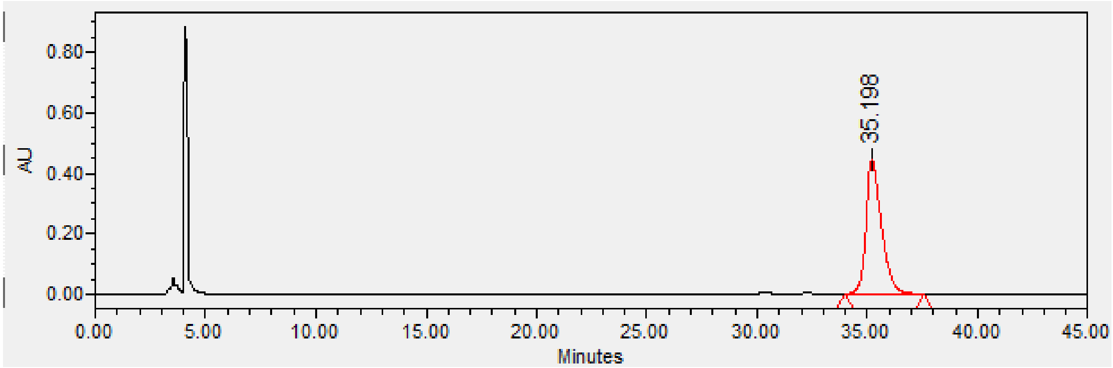

	Name	Retention Time (min)	Area (µV*sec)	% Area	Height (µV)	Int Type	Amount	Units	Peak Type	Peak Codes
1		35.198	21654372	100.00	447116	BB			Unknown	

(*S*)-1-(3-methoxyphenyl)butane-1,4-diol (4f)

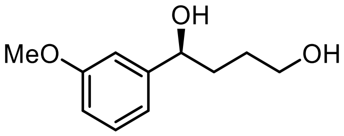

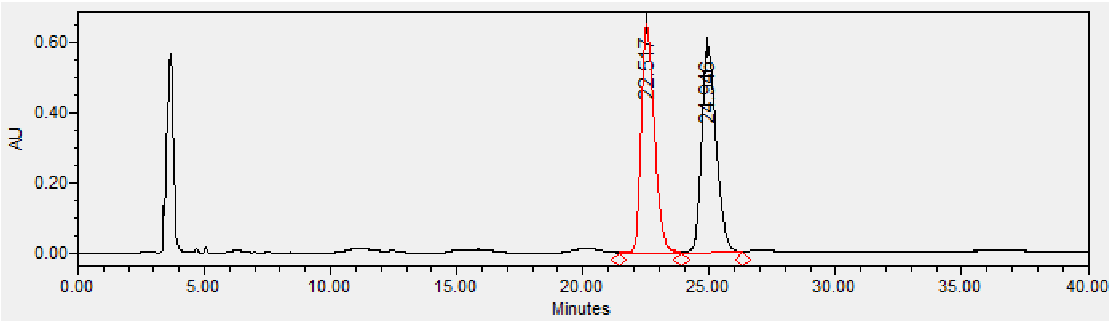

	Name	Retention Time (min)	Area (µV*sec)	% Area	Height (µV)	Int Type	Amount	Units	Peak Type	Peak Codes
1		22.517	23410342	49.97	648748	VV			Unknown	
2		24.946	23442858	50.03	579240	VV			Unknown	

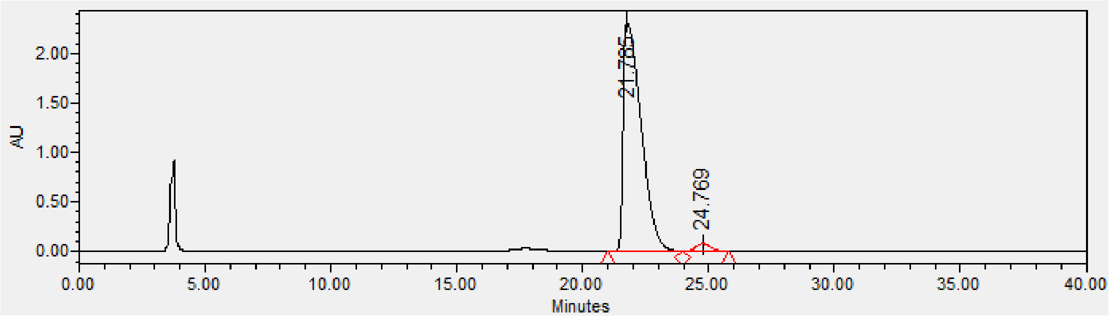

	Name	Retention Time (min)	Area (µV*sec)	% Area	Height (µV)	Int Type	Amount	Units	Peak Type	Peak Codes
1		21.785	114843311	97.32	2309927	BV			Unknown	
2		24.769	3156731	2.68	76920	Vb			Unknown	

(*S*)-1-(4-methoxyphenyl)butane-1,4-diol (4g)

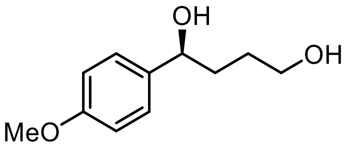

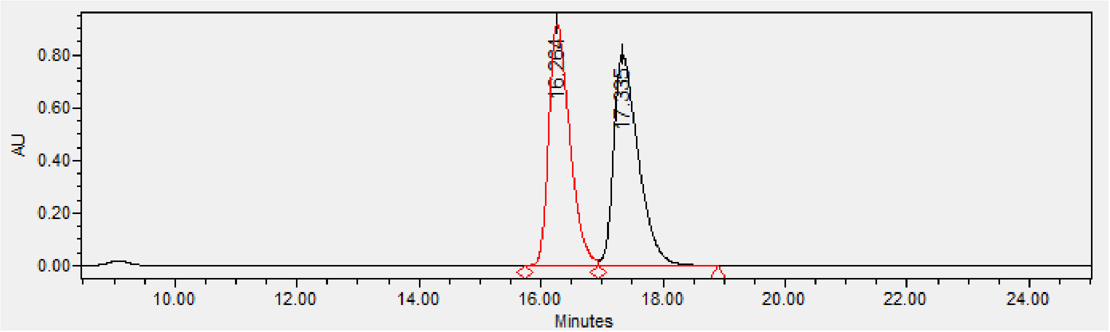

	Name	Retention Time (min)	Area (µV*sec)	% Area	Height (µV)	Int Type	Amount	Units	Peak Type	Peak Codes
1		16.264	21895245	49.38	915348	VV			Unknown	
2		17.335	22447023	50.62	800604	VB			Unknown	

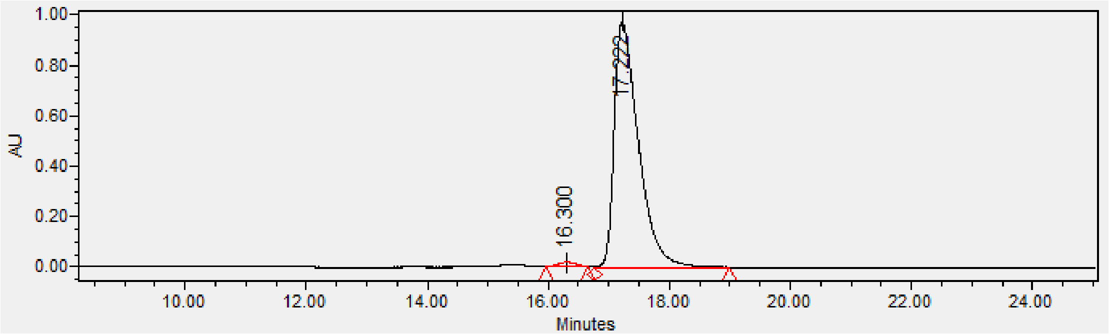

	Name	Retention Time (min)	Area (µV*sec)	% Area	Height (µV)	Int Type	Amount	Units	Peak Type	Peak Codes
1		16.300	368991	1.35	18493	bb			Unknown	
2		17.222	27056723	98.65	973679	VB			Unknown	

(*S*)-1-(4-(trifluoromethoxy)phenyl)butane-1,4-diol (4h)

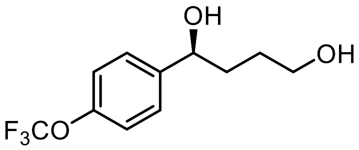

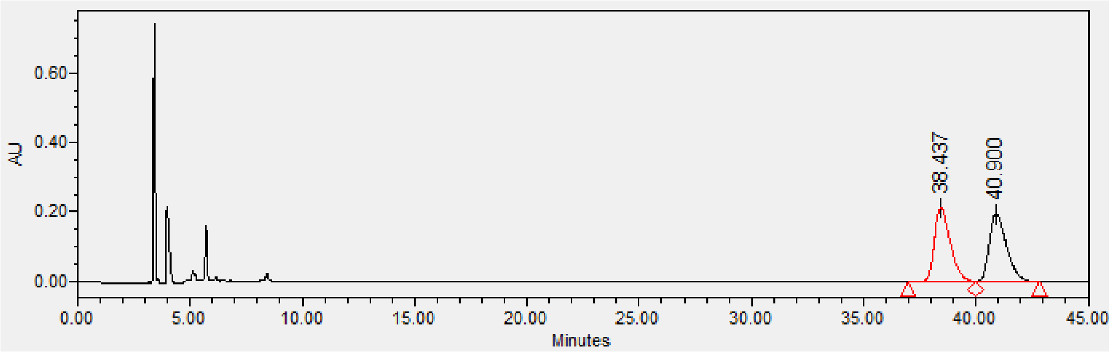

	Name	Retention Time (min)	Area (µV*sec)	% Area	Height (µV)	Int Type	Amount	Units	Peak Type	Peak Codes
1		38.437	10684931	50.25	214795	BV			Unknown	
2		40.900	10576981	49.75	197311	VB			Unknown	

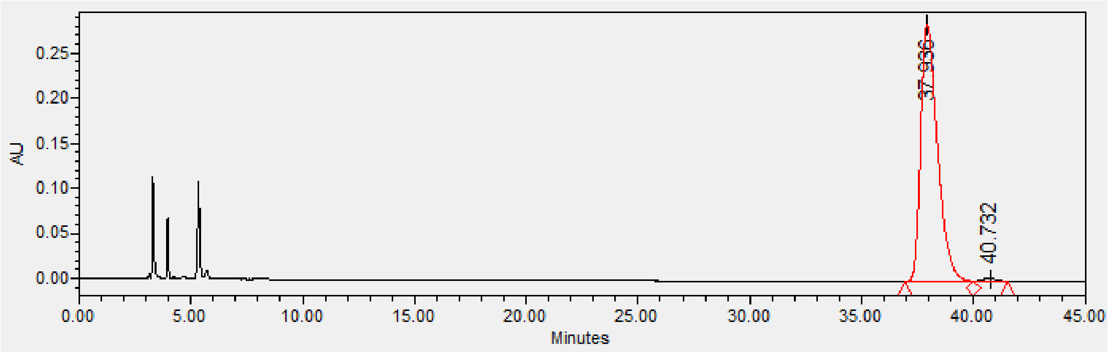

	Name	Retention Time (min)	Area (µV*sec)	% Area	Height (µV)	Int Type	Amount	Units	Peak Type	Peak Codes
1		37.936	14897287	98.85	284636	BV			Unknown	
2		40.732	172771	1.15	3554	VB			Unknown	

(*S*)-1-(4-(benzyloxy)phenyl)butane-1,4-diol (4i)

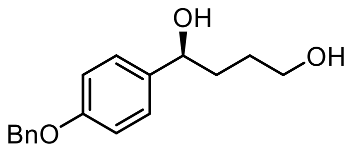

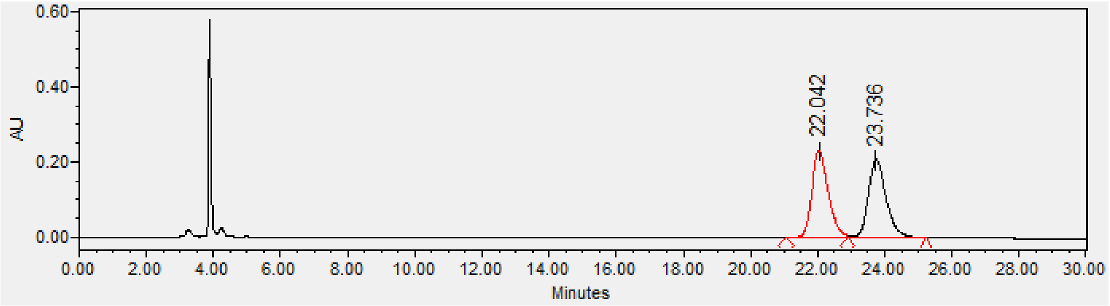

	Name	Retention Time (min)	Area (µV*sec)	% Area	Height (µV)	Int Type	Amount	Units	Peak Type	Peak Codes
1		22.042	7882573	50.55	231065	Vv			Unknown	
2		23.736	7712016	49.45	207873	vB			Unknown	

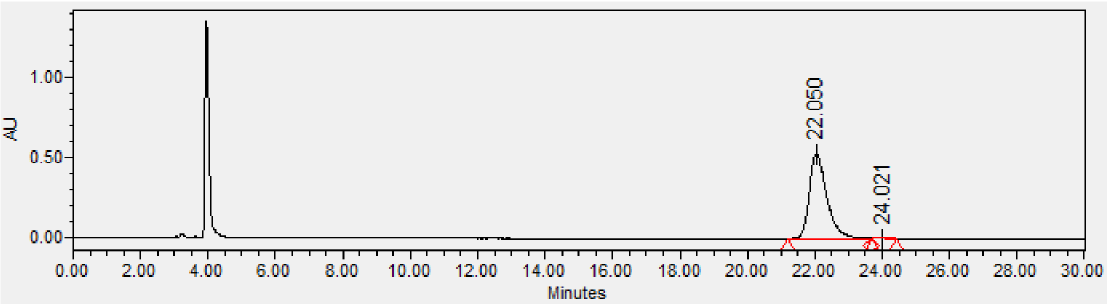

	Name	Retention Time (min)	Area (µV*sec)	% Area	Height (µV)	Int Type	Amount	Units	Peak Type	Peak Codes
1		22.050	18925381	99.85	528375	BV			Unknown	
2		24.021	28631	0.15	1255	bb			Unknown	

(*S*)-1-(2-fluorophenyl)butane-1,4-diol (4j)

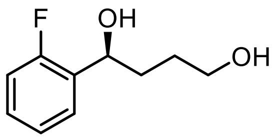

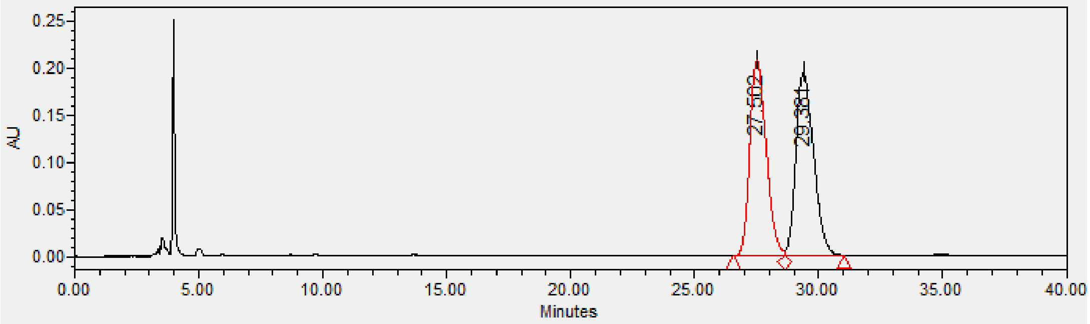

	Name	Retention Time (min)	Area (µV*sec)	% Area	Height (µV)	Int Type	Amount	Units	Peak Type	Peak Codes
1		27.502	9585268	49.77	208020	Bv			Unknown	
2		29.381	9672018	50.23	194461	vB			Unknown	

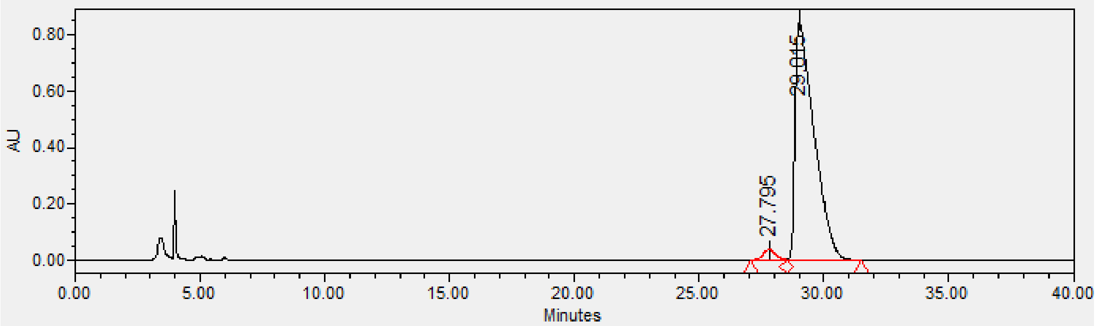

	Name	Retention Time (min)	Area (µV*sec)	% Area	Height (µV)	Int Type	Amount	Units	Peak Type	Peak Codes
1		27.795	1271197	2.77	36744	BV			Unknown	
2		29.015	44676044	97.23	847349	VB			Unknown	

(*S*)-1-(4-fluorophenyl)butane-1,4-diol (4k)

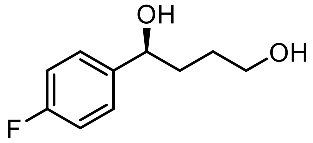

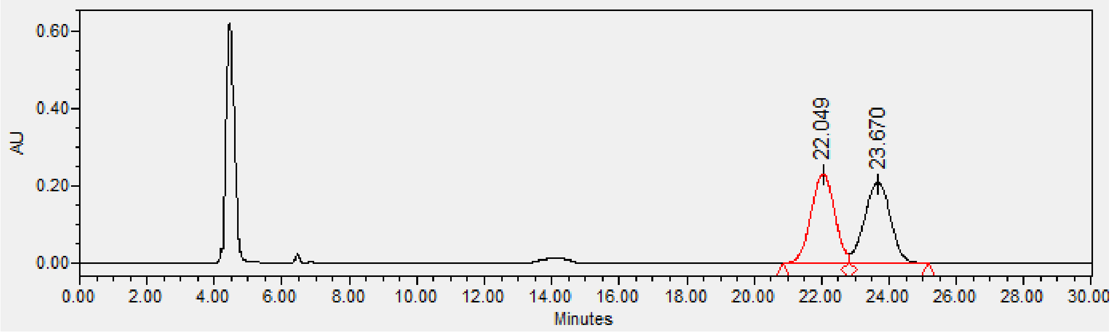

	Name	Retention Time (min)	Area (µV*sec)	% Area	Height (µV)	Int Type	Amount	Units	Peak Type	Peak Codes
1		22.049	11152098	50.16	229199	BV			Unknown	
2		23.670	11079179	49.84	207012	VB			Unknown	

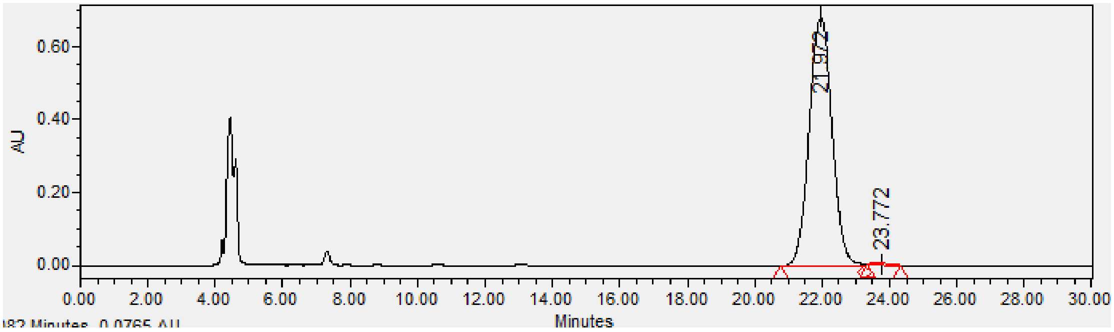

	Name	Retention Time (min)	Area (µV*sec)	% Area	Height (µV)	Int Type	Amount	Units	Peak Type	Peak Codes
1		21.972	32083857	99.73	680268	BV			Unknown	
2		23.772	87477	0.27	2612	bb			Unknown	I08

(*S*)-1-(4-(trifluoromethyl)phenyl)butane-1,4-diol (4l)

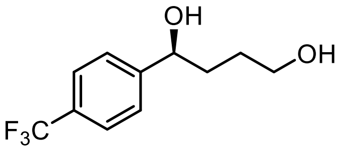

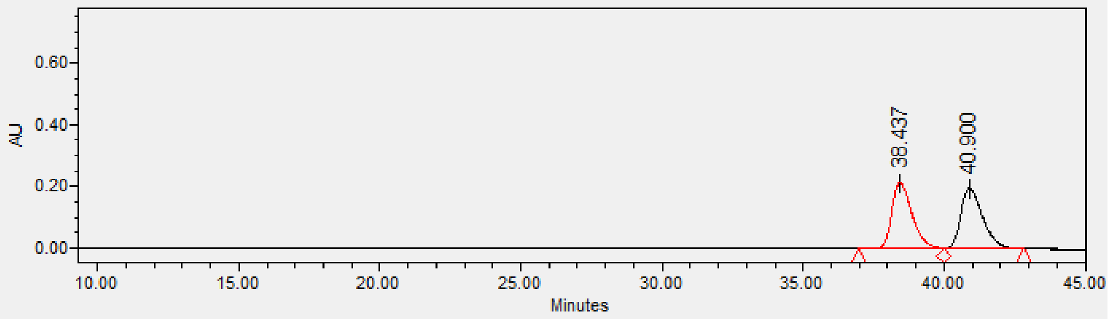

	Name	Retention Time (min)	Area (µV*sec)	% Area	Height (µV)	Int Type	Amount	Units	Peak Type	Peak Codes
1		38.437	10684931	50.25	214795	BV			Unknown	
2		40.900	10576981	49.75	197311	VB			Unknown	

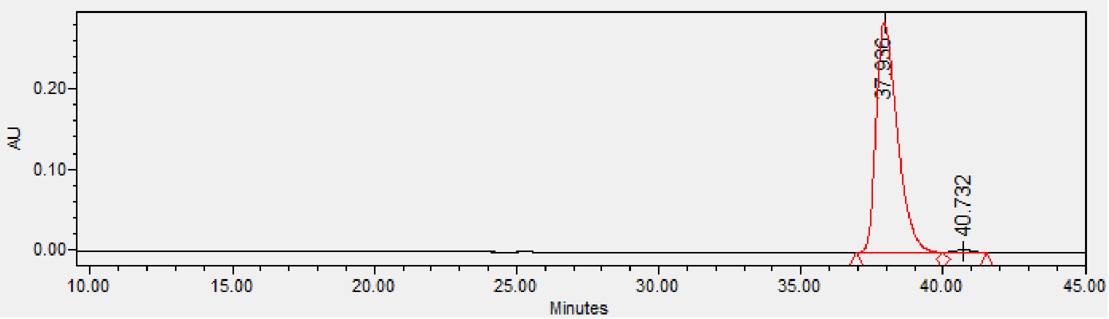

	Name	Retention Time (min)	Area (µV*sec)	% Area	Height (µV)	Int Type	Amount	Units	Peak Type	Peak Codes
1		37.936	14897287	98.85	284636	BV			Unknown	
2		40.732	172771	1.15	3554	VB			Unknown	

(*S*)-1-(3-bromophenyl)butane-1,4-diol (4m)

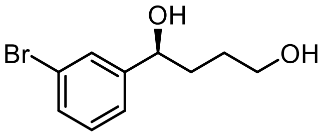

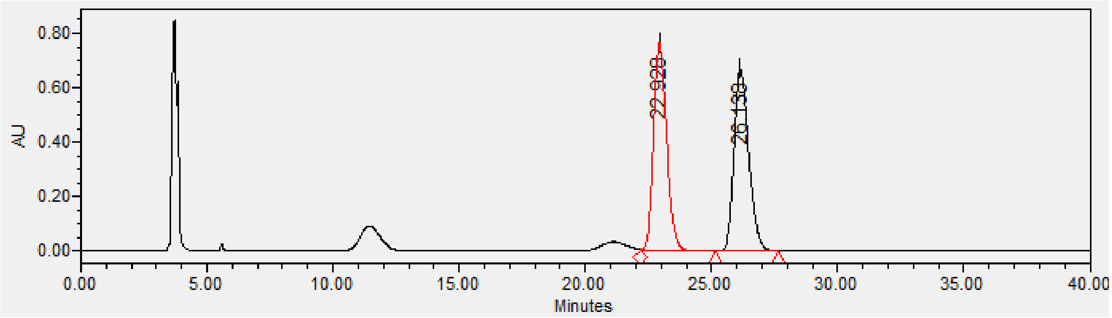

	Name	Retention Time (min)	Area (µV*sec)	% Area	Height (µV)	Int Type	Amount	Units	Peak Type	Peak Codes
1		22.928	27635039	50.40	762496	VB			Unknown	
2		26.138	27201288	49.60	665947	BB			Unknown	

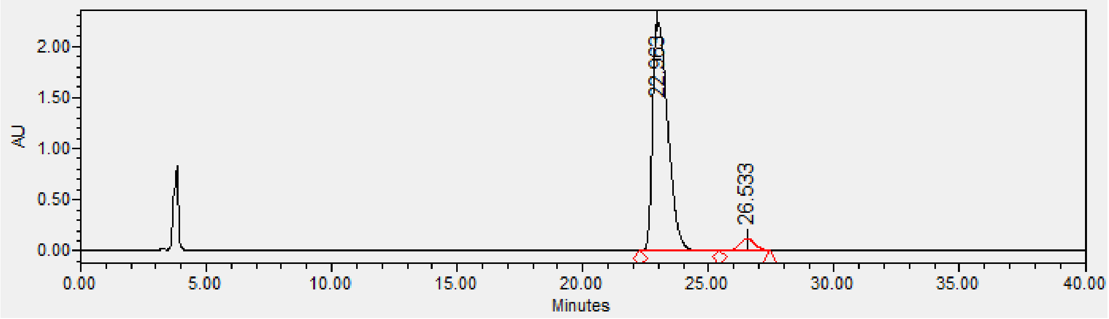

	Name	Retention Time (min)	Area (µV*sec)	% Area	Height (µV)	Int Type	Amount	Units	Peak Type	Peak Codes
1		22.963	95156726	95.20	2238641	VV			Unknown	
2		26.533	4795124	4.80	117005	Vb			Unknown	

(*S*)-1-([1,1'-biphenyl]-4-yl)butane-1,4-diol (4n)

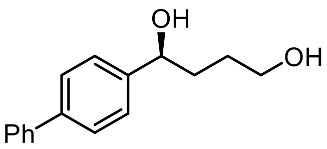

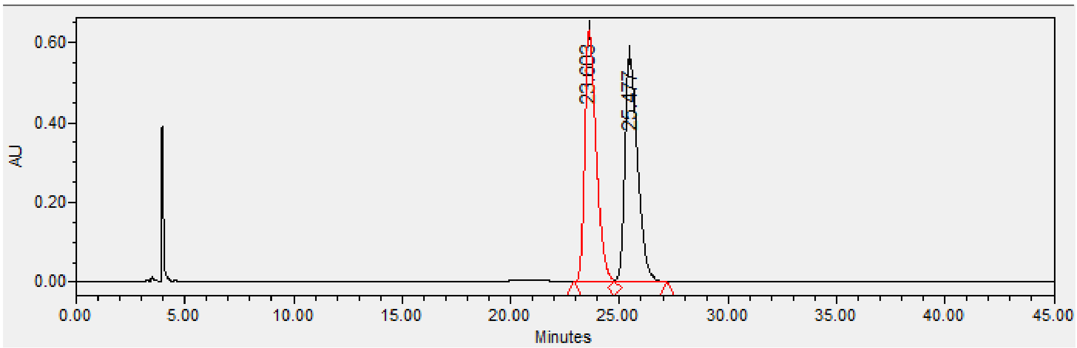

	Name	Retention Time (min)	Area (μV*sec)	% Area	Height (μV)	Int Type	Amount	Units	Peak Type	Peak Codes
1		23.603	23272163	50.00	630384	BV			Unknown	
2		25.477	23274269	50.00	565357	VB			Unknown	

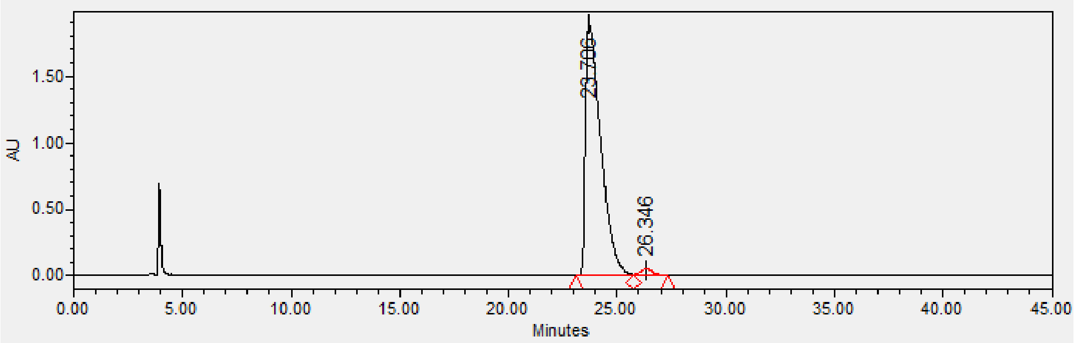

	Name	Retention Time (min)	Area (μV*sec)	% Area	Height (μV)	Int Type	Amount	Units	Peak Type	Peak Codes
1		23.706	92169926	97.83	1888848	BV			Unknown	
2		26.346	2042159	2.17	48145	Vb			Unknown	

(*S*)-1-(naphthalen-1-yl)butane-1,4-diol (4o)

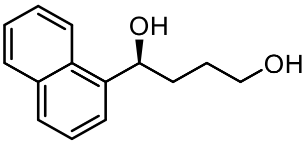

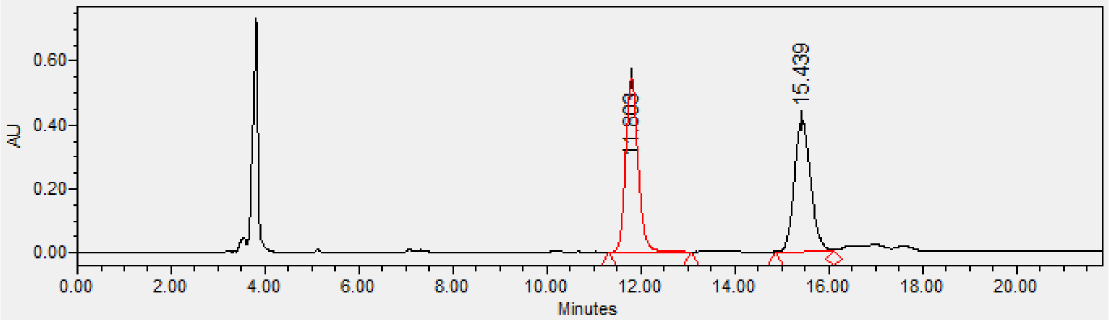

	Name	Retention Time (min)	Area (µV*sec)	% Area	Height (µV)	Int Type	Amount	Units	Peak Type	Peak Codes
1		11.803	13479585	50.04	728831	BB			Unknown	
2		15.439	13457990	49.96	552803	BV			Unknown	

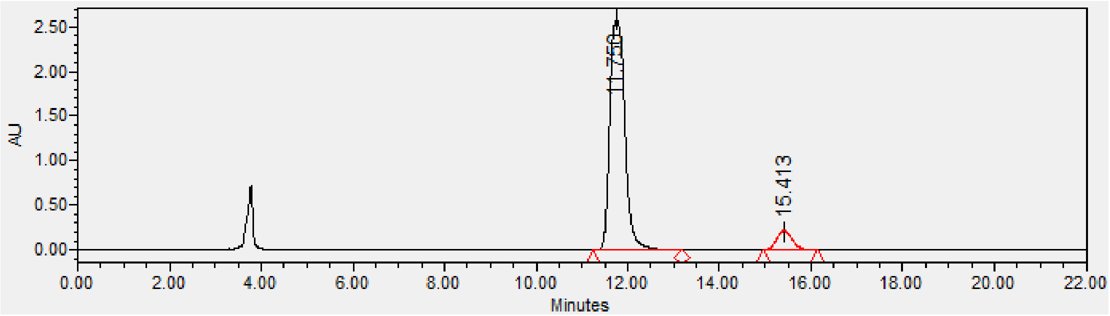

	Name	Retention Time (min)	Area (µV*sec)	% Area	Height (µV)	Int Type	Amount	Units	Peak Type	Peak Codes
1		11.750	58925606	92.03	2583224	BV			Unknown	
2		15.413	5101097	7.97	214608	bb			Unknown	

(*S*)-1-(naphthalen-2-yl)butane-1,4-diol (4p)

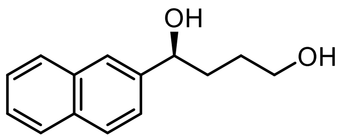

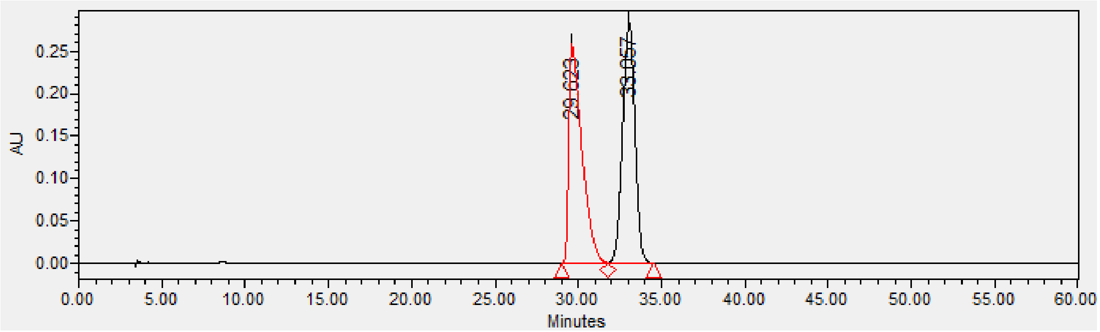

	Name	Retention Time (min)	Area (μV*sec)	% Area	Height (μV)	Int Type	Amount	Units	Peak Type	Peak Codes
1		29.623	14383731	49.78	257614	BV			Unknown	
2		33.057	14508316	50.22	283056	VB			Unknown	

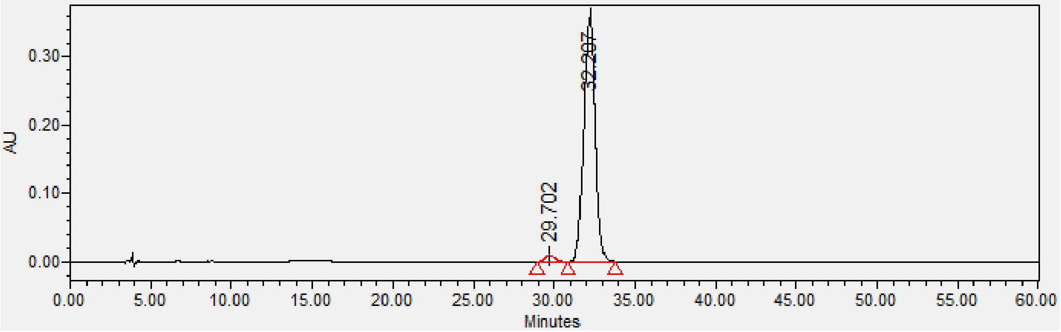

	Name	Retention Time (min)	Area (μV*sec)	% Area	Height (μV)	Int Type	Amount	Units	Peak Type	Peak Codes
1		29.702	425482	2.35	9807	BB			Unknown	
2		32.207	17689885	97.65	357027	BB			Unknown	

(*S*)-1-(benzofuran-3-yl)butane-1,4-diol (4q)

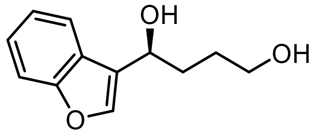

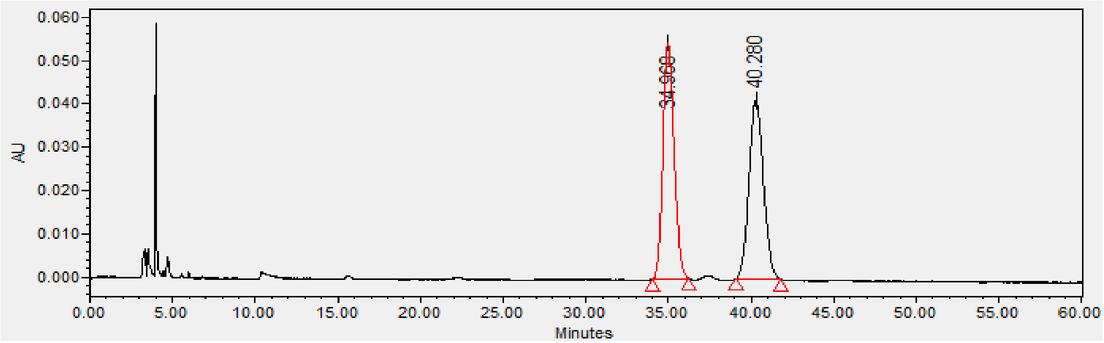

	Name	Retention Time (min)	Area (µV*sec)	% Area	Height (µV)	Int Type	Amount	Units	Peak Type	Peak Codes
1		34.968	2569096	50.41	54429	BB			Unknown	
2		40.280	2526820	49.59	41350	BB			Unknown	

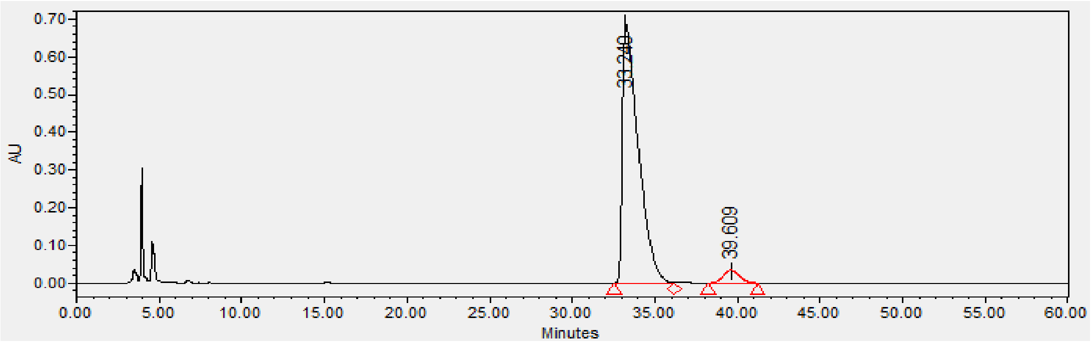

	Name	Retention Time (min)	Area (µV*sec)	% Area	Height (µV)	Int Type	Amount	Units	Peak Type	Peak Codes
1		33.240	46258380	95.27	686648	BV			Unknown	
2		39.609	2298749	4.73	34029	BB			Unknown	

(*S*)-1-(3,4-difluorophenyl)butane-1,4-diol (4r)

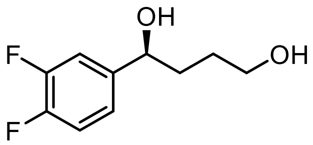

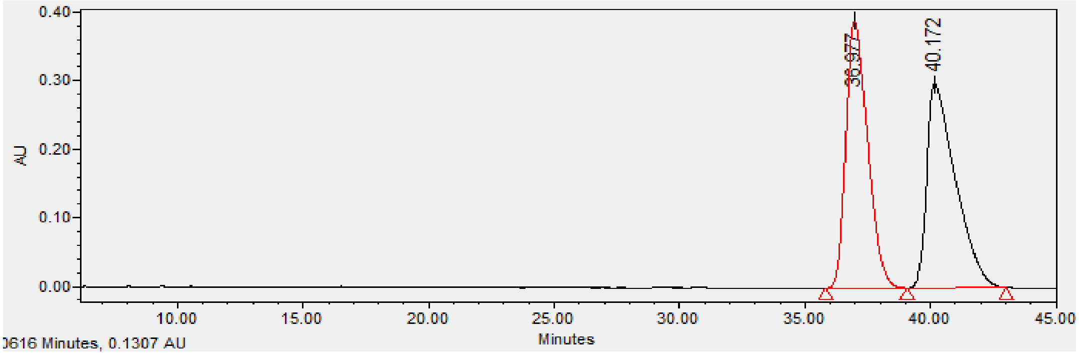

	Name	Retention Time (min)	Area (µV*sec)	% Area	Height (µV)	Int Type	Amount	Units	Peak Type	Peak Codes
1		36.977	23471925	49.78	388682	BB			Unknown	
2		40.172	23682340	50.22	297503	BB			Unknown	

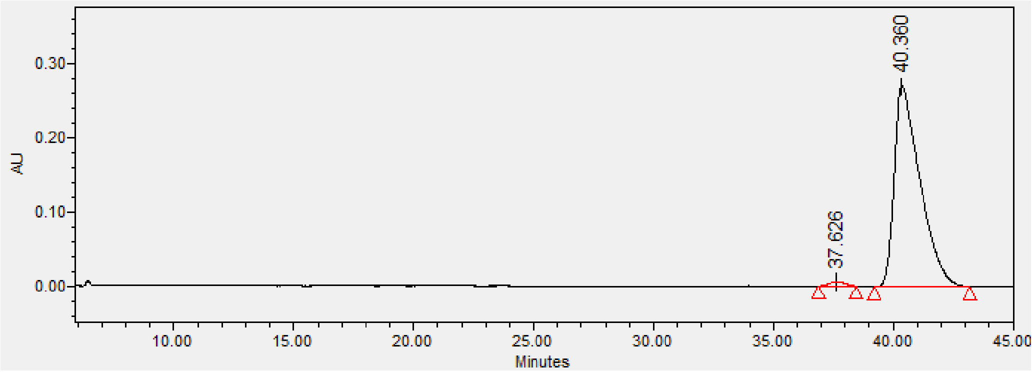

	Name	Retention Time (min)	Area (µV*sec)	% Area	Height (µV)	Int Type	Amount	Units	Peak Type	Peak Codes
1		37.626	294420	1.43	6478	bb			Unknown	
2		40.360	20229560	98.57	271129	BB			Unknown	

(*S*)-1-(3,4,5-trimethoxyphenyl)butane-1,4-diol (4s)

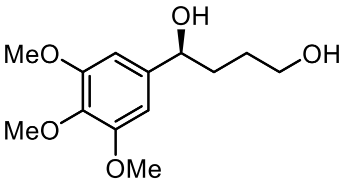

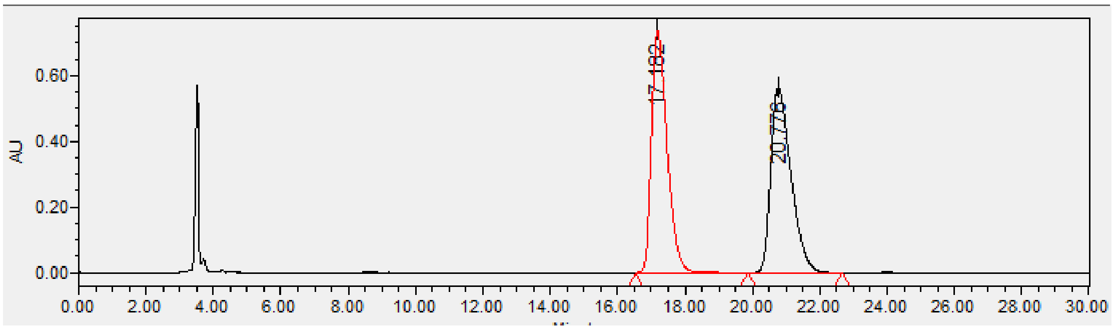

	Name	Retention Time (min)	Area (µV*sec)	% Area	Height (µV)	Int Type	Amount	Units	Peak Type	Peak Codes
1		17.182	23839195	50.32	738602	BB			Unknown	
2		20.776	23531859	49.68	565441	BB			Unknown	

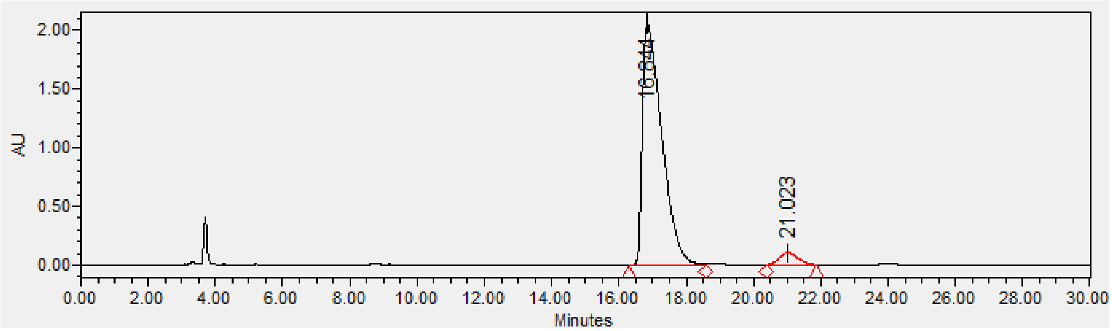

	Name	Retention Time (min)	Area (µV*sec)	% Area	Height (µV)	Int Type	Amount	Units	Peak Type	Peak Codes
1		16.844	82544086	95.34	2053129	BV			Unknown	
2		21.023	4030136	4.66	104942	vb			Unknown	

(*S*)-4-phenylbutane-1,3-diol (7a)

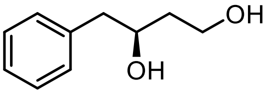

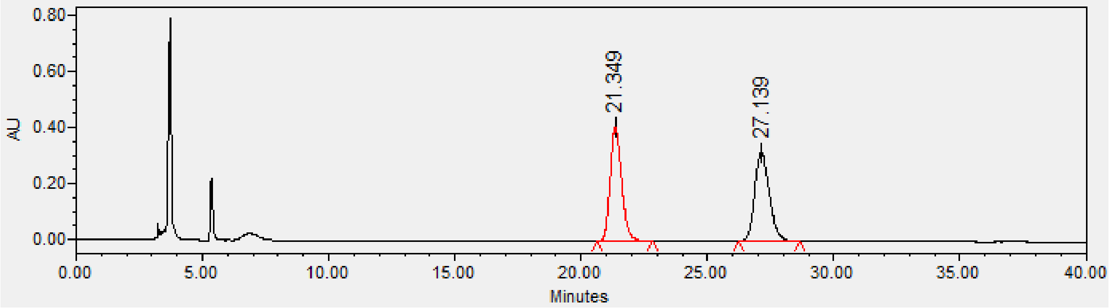

	Name	Retention Time (min)	Area (μV*sec)	% Area	Height (μV)	Int Type	Amount	Units	Peak Type	Peak Codes
1		21.349	12873302	49.94	406902	BB			Unknown	
2		27.139	12906807	50.06	319866	BB			Unknown	

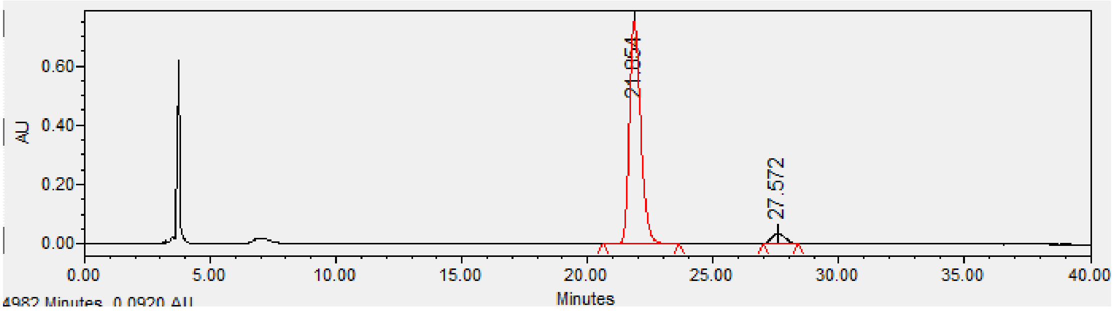

	Name	Retention Time (min)	Area (μV*sec)	% Area	Height (μV)	Int Type	Amount	Units	Peak Type	Peak Codes
1		21.854	23426936	94.89	749269	bB			Unknown	
2		27.572	1260367	5.11	34631	bb			Unknown	

(*S*)-4-(o-tolyl)butane-1,3-diol (7b)

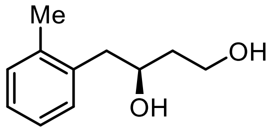

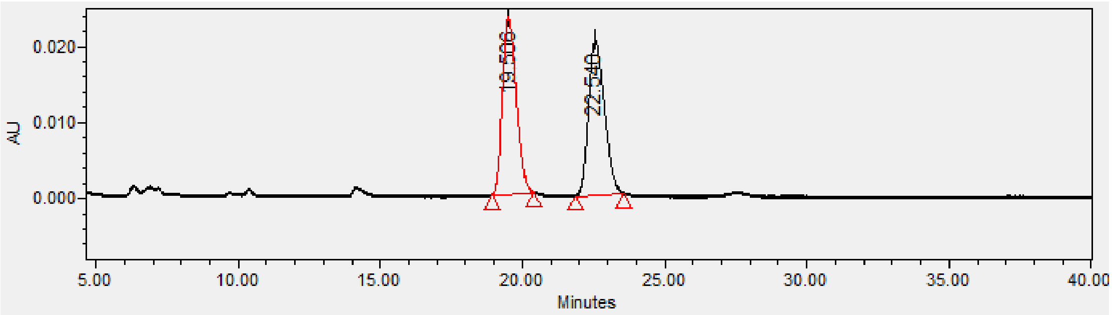

	Name	Retention Time (min)	Area (µV*sec)	% Area	Height (µV)	Int Type	Amount	Units	Peak Type	Peak Codes
1		19.506	796099	49.89	23397	BB			Unknown	
2		22.540	799460	50.11	20487	BB			Unknown	

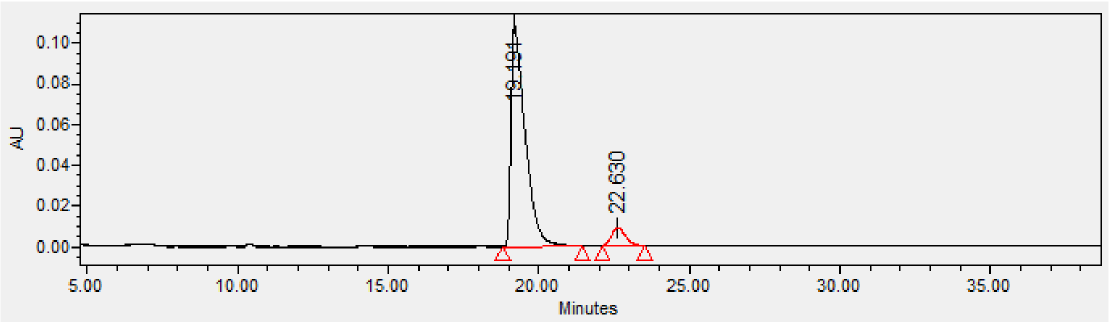

	Name	Retention Time (min)	Area (µV*sec)	% Area	Height (µV)	Int Type	Amount	Units	Peak Type	Peak Codes
1		19.191	3576312	92.36	108806	Bb			Unknown	
2		22.630	295973	7.64	9133	Bb			Unknown	

(*S*)-4-(4-(tert-butyl)phenyl)butane-1,3-diol (7c)

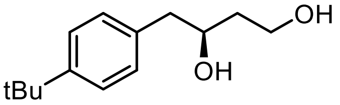

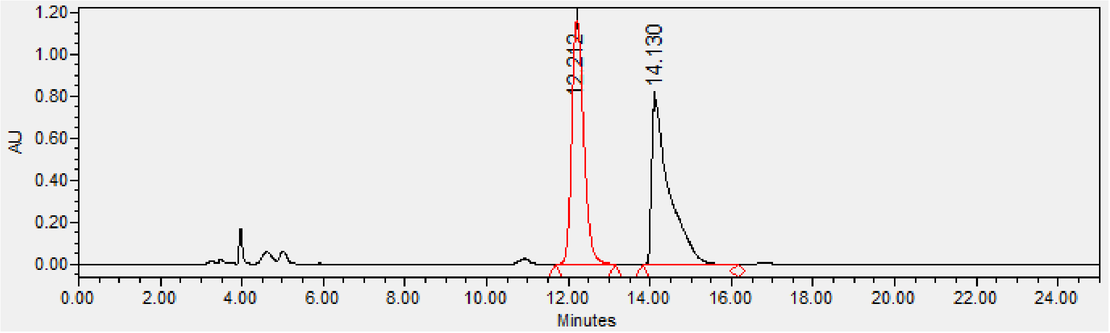

	Name	Retention Time (min)	Area (µV*sec)	% Area	Height (µV)	Int Type	Amount	Units	Peak Type	Peak Codes
1		12.212	23664244	50.07	1163761	BB			Unknown	
2		14.130	23600185	49.93	783084	BV			Unknown	

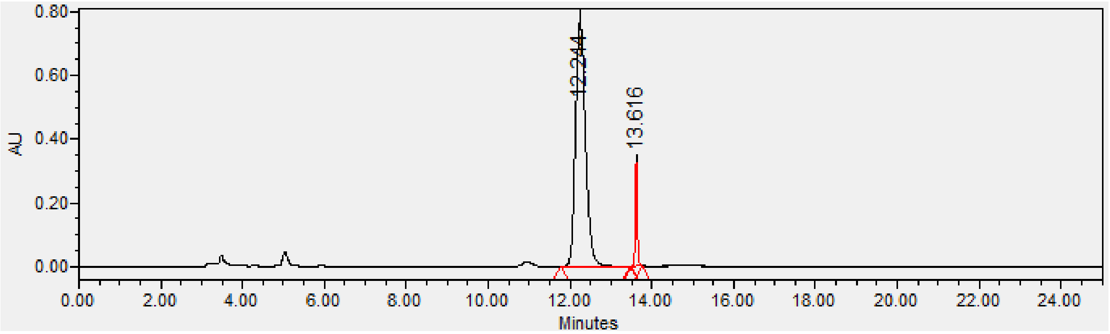

	Name	Retention Time (min)	Area (µV*sec)	% Area	Height (µV)	Int Type	Amount	Units	Peak Type	Peak Codes
1		12.244	12995363	92.41	770110	BB			Unknown	
2		13.616	1066835	7.59	323249	bb			Unknown	

(*S*)-4-(3-methoxyphenyl)butane-1,3-diol (7d)

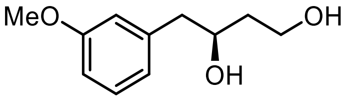

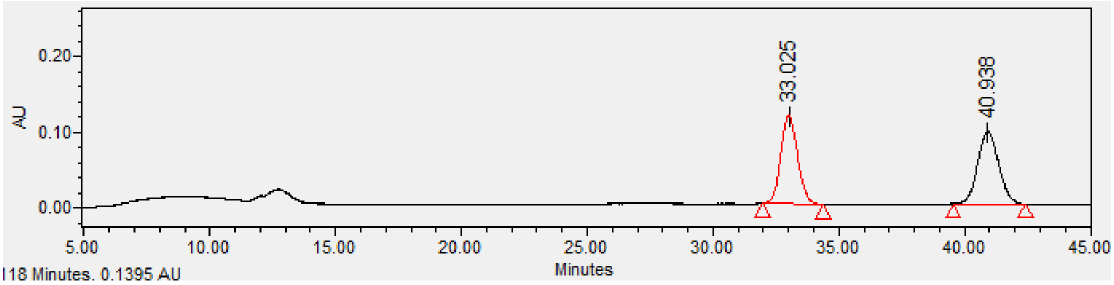

	Name	Retention Time (min)	Area (µV*sec)	% Area	Height (µV)	Int Type	Amount	Units	Peak Type	Peak Codes
1		33.025	5451516	49.37	117110	BB			Unknown	
2		40.938	5591570	50.63	95933	BB			Unknown	

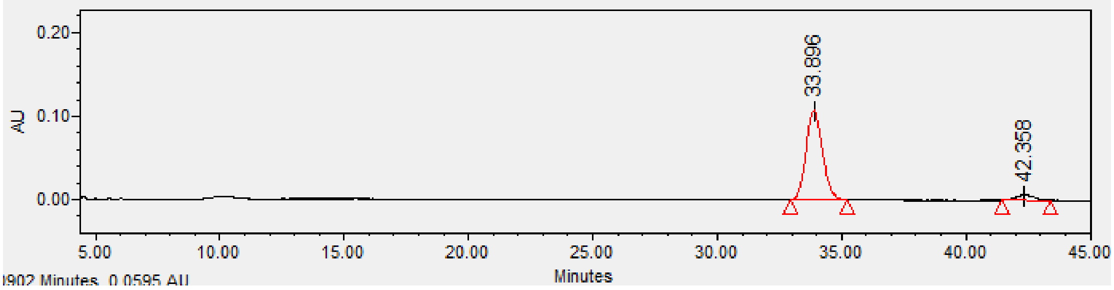

	Name	Retention Time (min)	Area (µV*sec)	% Area	Height (µV)	Int Type	Amount	Units	Peak Type	Peak Codes
1		33.896	4908209	93.48	106733	BB			Unknown	
2		42.358	342614	6.52	6423	BB			Unknown	

(*S*)-4-(4-(trifluoromethoxy)phenyl)butane-1,3-diol (7e)

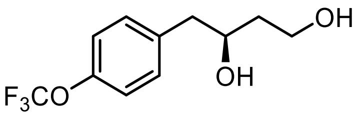

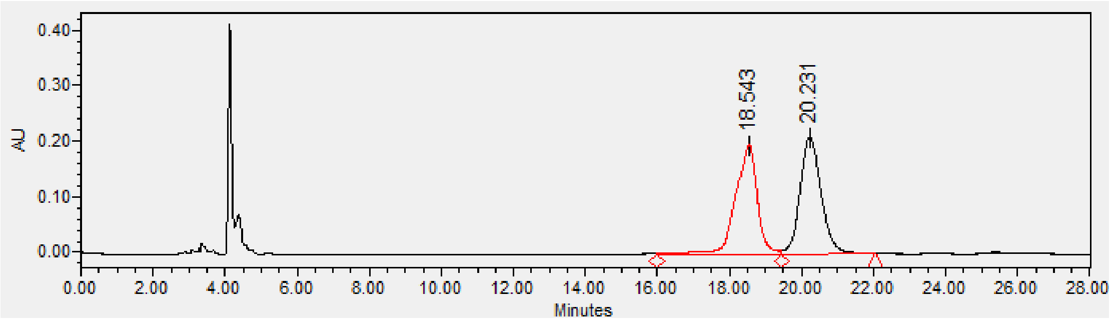

	Name	Retention Time (min)	Area (µV*sec)	% Area	Height (µV)	Int Type	Amount	Units	Peak Type	Peak Codes
1		18.543	8237249	49.10	197192	VV			Unknown	
2		20.231	8539096	50.90	209360	VB			Unknown	

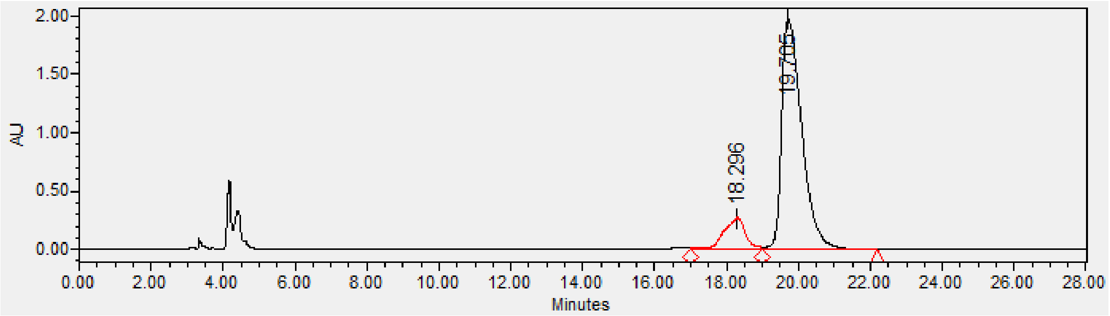

	Name	Retention Time (min)	Area (µV*sec)	% Area	Height (µV)	Int Type	Amount	Units	Peak Type	Peak Codes
1		18.296	11419884	12.93	271015	Vv			Unknown	
2		19.705	76905910	87.07	1965141	vB			Unknown	

(*S*)-4-(2-fluorophenyl)butane-1,3-diol (7f)

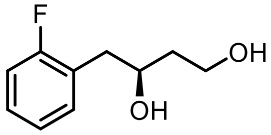

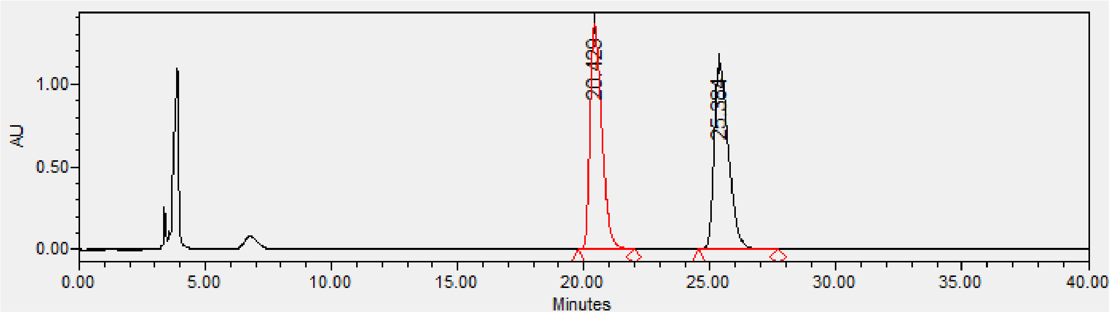

	Name	Retention Time (min)	Area (µV*sec)	% Area	Height (µV)	Int Type	Amount	Units	Peak Type	Peak Codes
1		20.429	44153936	49.89	1362458	BV			Unknown	
2		25.384	44343516	50.11	1121904	BV			Unknown	

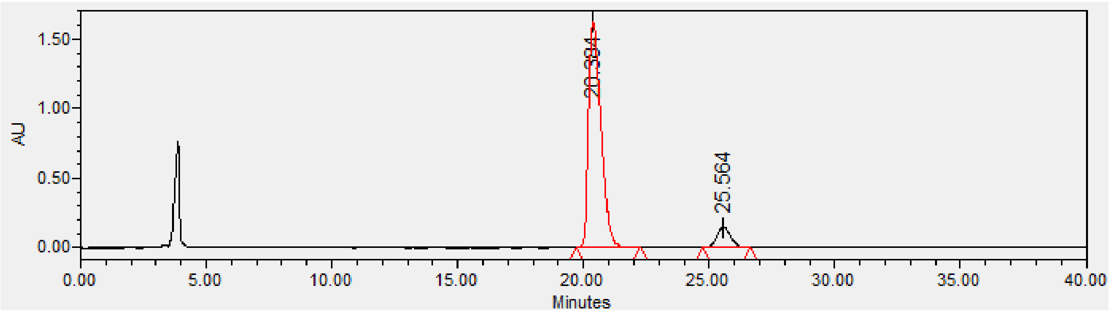

	Name	Retention Time (min)	Area (µV*sec)	% Area	Height (µV)	Int Type	Amount	Units	Peak Type	Peak Codes
1		20.384	55500867	90.71	1623446	Bb			Unknown	
2		25.564	5682084	9.29	147910	Bb			Unknown	

(*S*)-4-(4-fluorophenyl)butane-1,3-diol (7g)

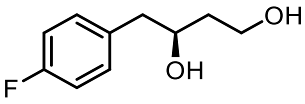

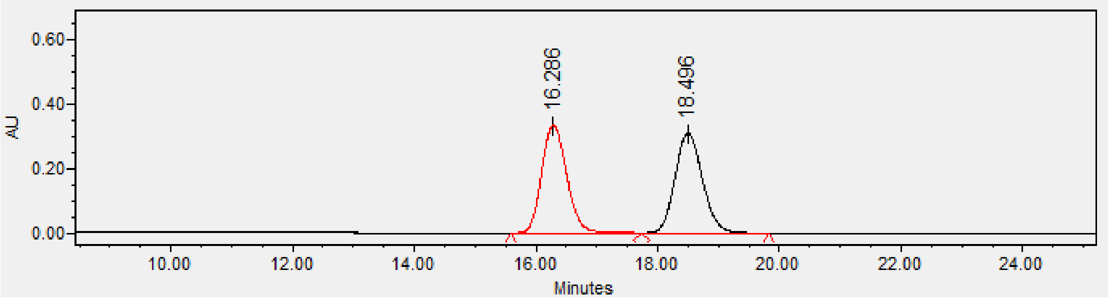

	Name	Retention Time (min)	Area (µV*sec)	% Area	Height (µV)	Int Type	Amount	Units	Peak Type	Peak Codes
1		16.286	9642642	49.56	335096	BV			Unknown	
2		18.496	9814826	50.44	310994	VB			Unknown	

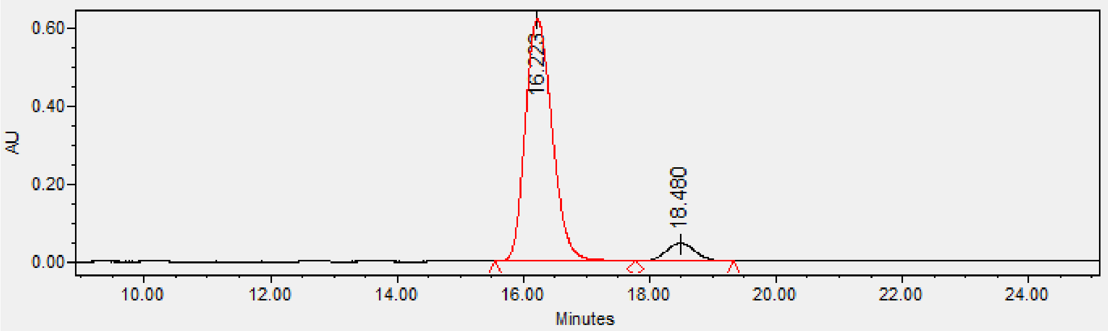

	Name	Retention Time (min)	Area (µV*sec)	% Area	Height (µV)	Int Type	Amount	Units	Peak Type	Peak Codes
1		16.223	18508463	92.88	623450	BV			Unknown	
2		18.480	1419684	7.12	45522	bb			Unknown	

(*S*)-4-(3-bromophenyl)butane-1,3-diol (7h)

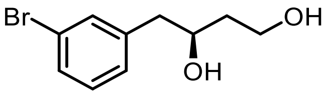

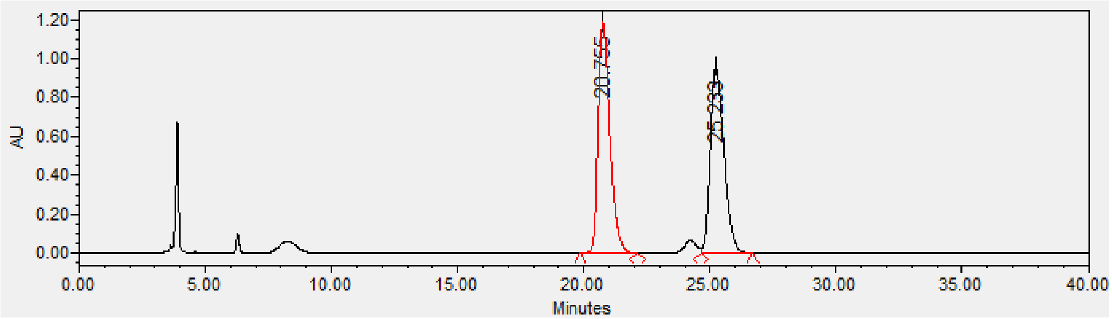

	Name	Retention Time (min)	Area (μV*sec)	% Area	Height (μV)	Int Type	Amount	Units	Peak Type	Peak Codes
1		20.755	37749718	51.26	1184841	BV			Unknown	
2		25.233	35895134	48.74	956686	VB			Unknown	

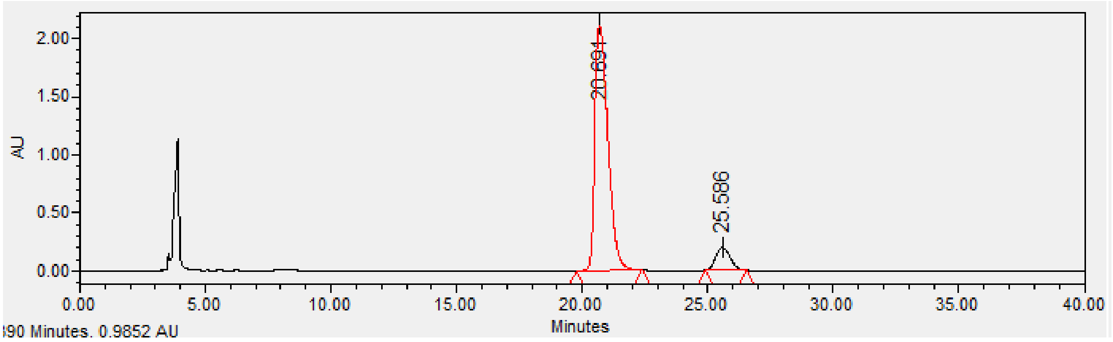

	Name	Retention Time (min)	Area (μV*sec)	% Area	Height (μV)	Int Type	Amount	Units	Peak Type	Peak Codes
1		20.691	76344216	90.58	2111944	Bb			Unknown	
2		25.586	7941582	9.42	200638	bb			Unknown	

(*S*)-4-([1,1'-biphenyl]-4-yl)butane-1,3-diol (7i)

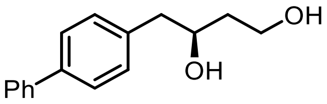

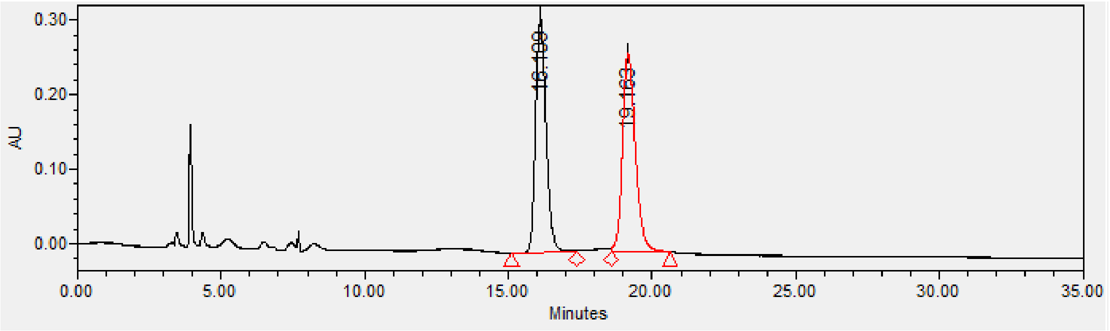

	Name	Retention Time (min)	Area (µV*sec)	% Area	Height (µV)	Int Type	Amount	Units	Peak Type	Peak Codes
1		16.109	8012525	49.18	315524	bv			Unknown	
2		19.163	8278162	50.82	266142	vb			Unknown	

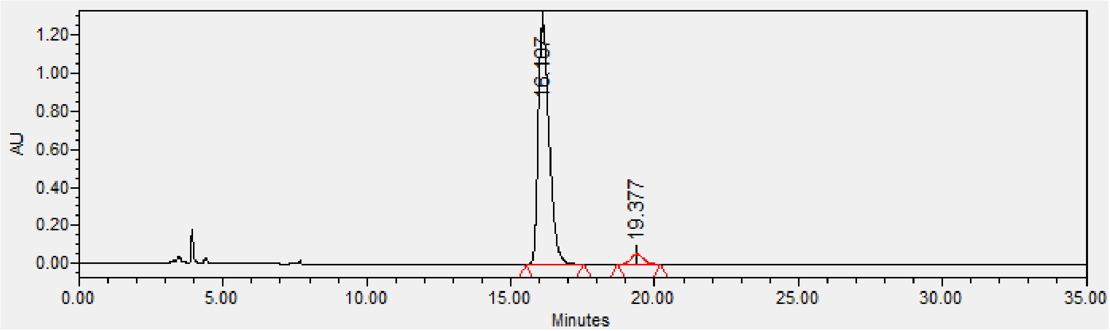

	Name	Retention Time (min)	Area (µV*sec)	% Area	Height (µV)	Int Type	Amount	Units	Peak Type	Peak Codes
1		16.107	33667643	95.47	1264848	BB			Unknown	
2		19.377	1597719	4.53	53427	Bb			Unknown	

(*S*)-4-(naphthalen-1-yl)butane-1,3-diol (7j)

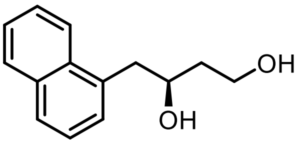

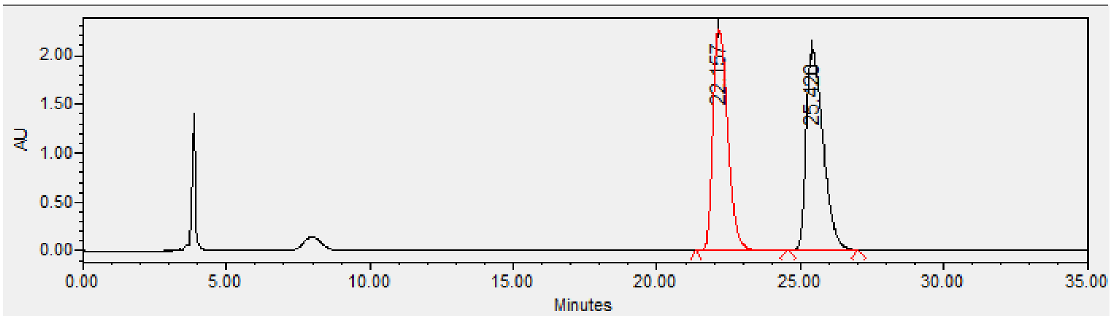

	Name	Retention Time (min)	Area (μV*sec)	% Area	Height (μV)	Int Type	Amount	Units	Peak Type	Peak Codes
1		22.157	81420621	49.11	2267385	bb			Unknown	
2		25.420	84387690	50.89	2045724	bb			Unknown	

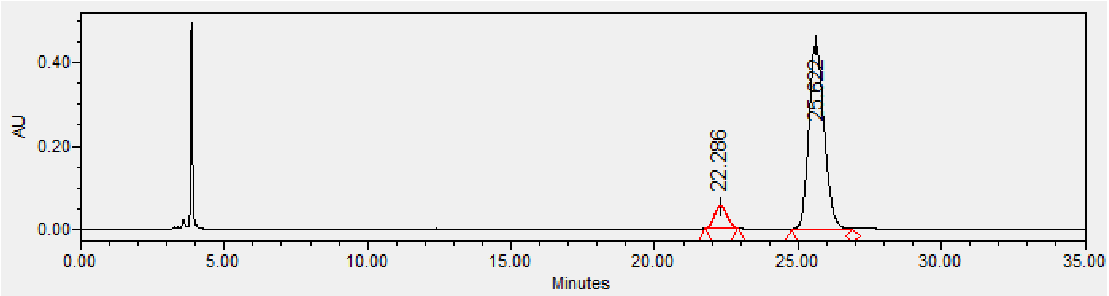

	Name	Retention Time (min)	Area (μV*sec)	% Area	Height (μV)	Int Type	Amount	Units	Peak Type	Peak Codes
1		22.286	1631644	9.01	53662	bb			Unknown	
2		25.622	16470207	90.99	440739	BV			Unknown	

(*S*)-4-(naphthalen-2-yl)butane-1,3-diol (7k)

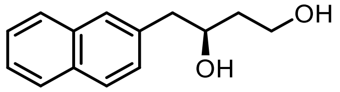

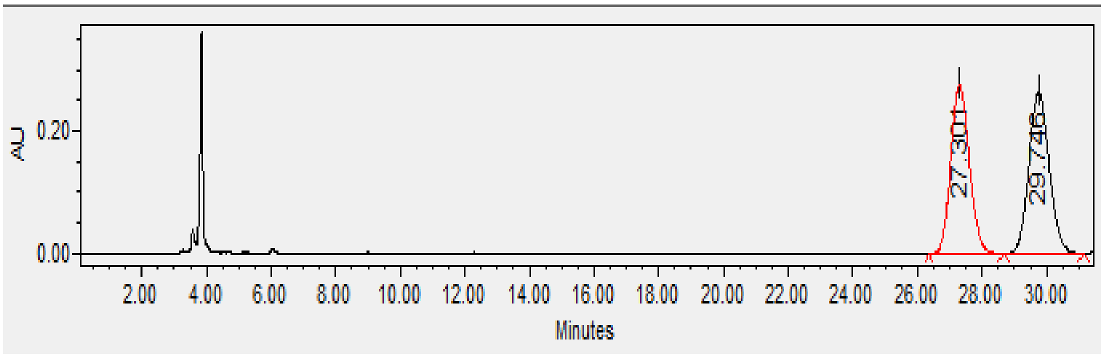

	Name	Retention Time (min)	Area (μV*sec)	% Area	Height (μV)	Int Type	Amount	Units	Peak Type	Peak Codes
1		27.301	10995756	48.76	276202	BV			Unknown	
2		29.746	11553078	51.24	264052	VV			Unknown	

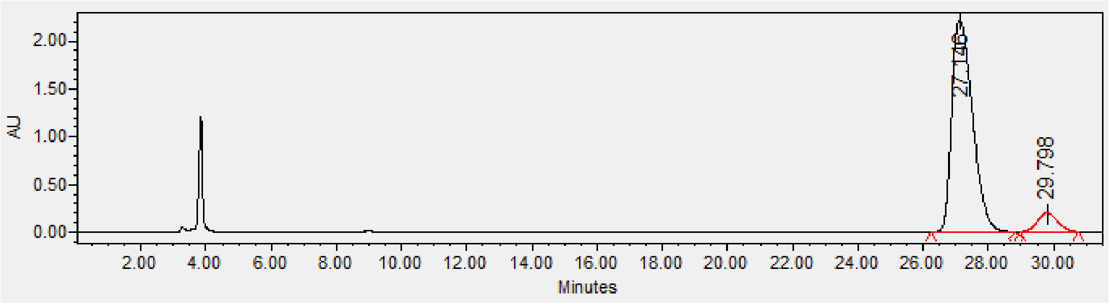

	Name	Retention Time (min)	Area (μV*sec)	% Area	Height (μV)	Int Type	Amount	Units	Peak Type	Peak Codes
1		27.146	98851633	92.11	2221187	BV			Unknown	
2		29.798	8465477	7.89	197127	bb			Unknown	

(*S*)-4-(benzofuran-3-yl)butane-1,3-diol (7l)

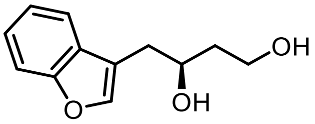

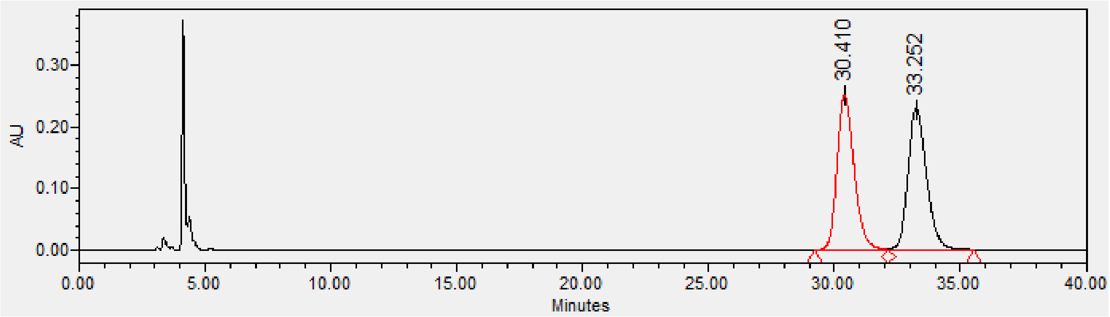

	Name	Retention Time (min)	Area (µV*sec)	% Area	Height (µV)	Int Type	Amount	Units	Peak Type	Peak Codes
1		30.410	11859942	50.30	250860	BV			Unknown	
2		33.252	11717463	49.70	228939	VB			Unknown	

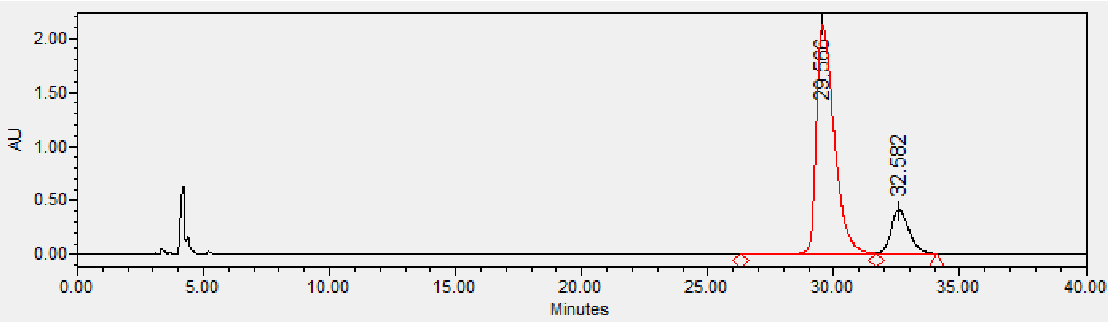

	Name	Retention Time (min)	Area (µV*sec)	% Area	Height (µV)	Int Type	Amount	Units	Peak Type	Peak Codes
1		29.566	104890780	83.85	2119363	Vv			Unknown	
2		32.582	20207738	16.15	408721	vb			Unknown	

(*S*)-4-(3,4-difluorophenyl)butane-1,3-diol (7m)

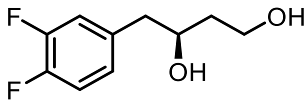

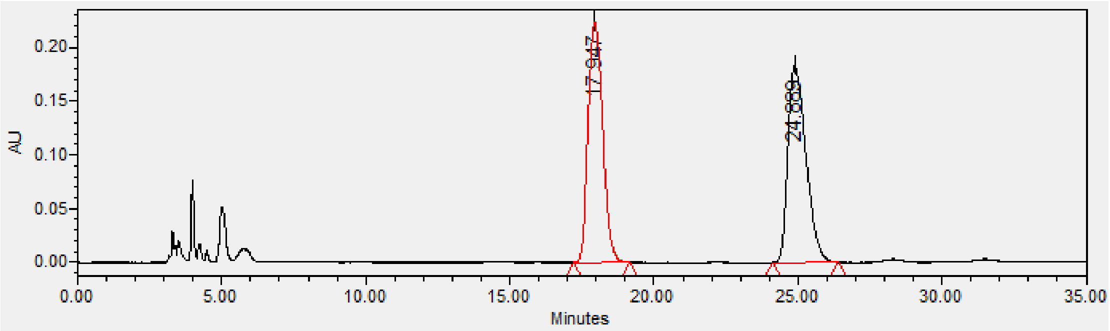

	Name	Retention Time (min)	Area (µV*sec)	% Area	Height (µV)	Int Type	Amount	Units	Peak Type	Peak Codes
1		17.947	7922282	49.83	223662	BB			Unknown	
2		24.889	7976718	50.17	183284	BB			Unknown	

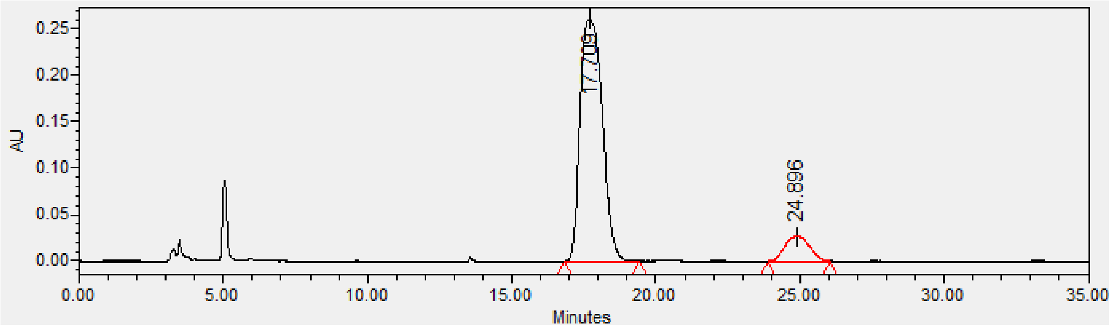

	Name	Retention Time (min)	Area (µV*sec)	% Area	Height (µV)	Int Type	Amount	Units	Peak Type	Peak Codes
1		17.709	13925802	89.51	260311	BB			Unknown	
2		24.896	1632691	10.49	27069	BB			Unknown	

(*S*)-4-(3,4,5-trimethoxyphenyl)butane-1,3-diol (7n)

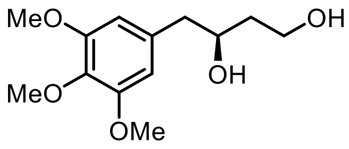

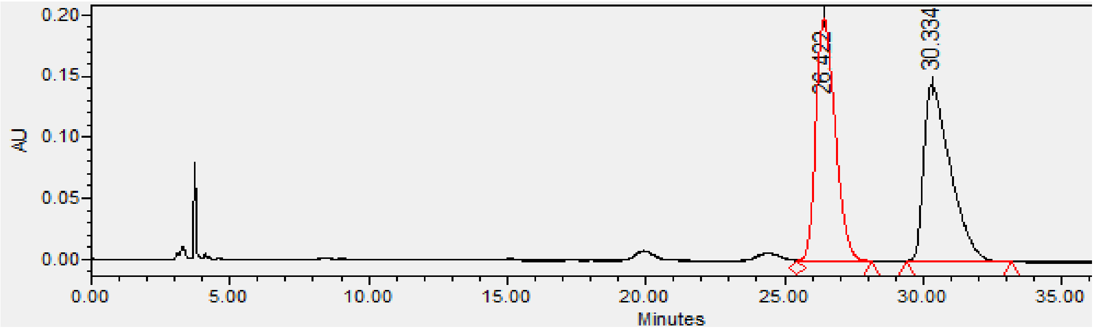

	Name	Retention Time (min)	Area (µV*sec)	% Area	Height (µV)	Int Type	Amount	Units	Peak Type	Peak Codes
1		26.422	9649820	49.73	199381	VB			Unknown	
2		30.334	9754536	50.27	144770	BB			Unknown	

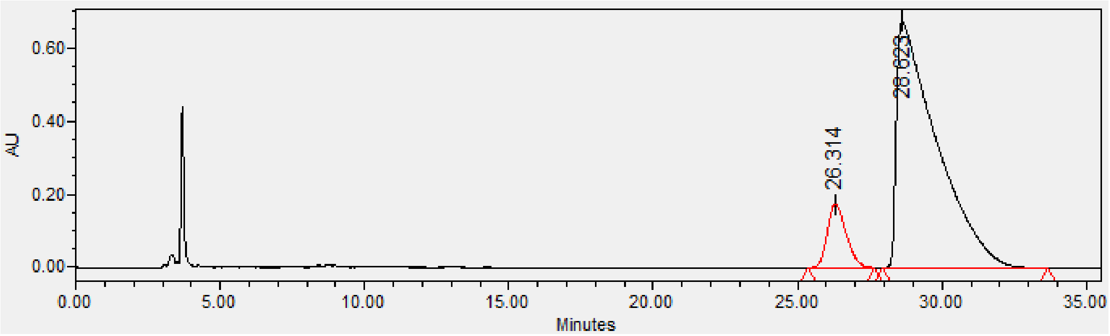

	Name	Retention Time (min)	Area (µV*sec)	% Area	Height (µV)	Int Type	Amount	Units	Peak Type	Peak Codes
1		26.314	7838826	10.72	173086	bb			Unknown	
2		28.623	65274360	89.28	673123	BB			Unknown	

(*S*)-4-hydroxy-4-phenylbutyl diphenylphosphinate (8)

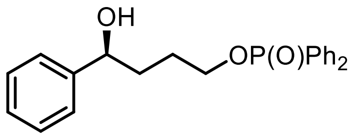

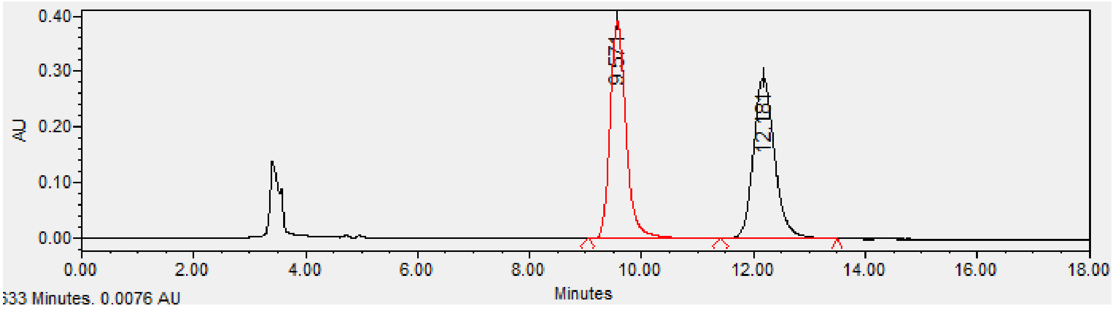

	Name	Retention Time (min)	Area (µV*sec)	% Area	Height (µV)	Int Type	Amount	Units	Peak Type	Peak Codes
1		9.571	7817941	50.42	392229	VV			Unknown	
2		12.181	7687261	49.58	290921	VB			Unknown	

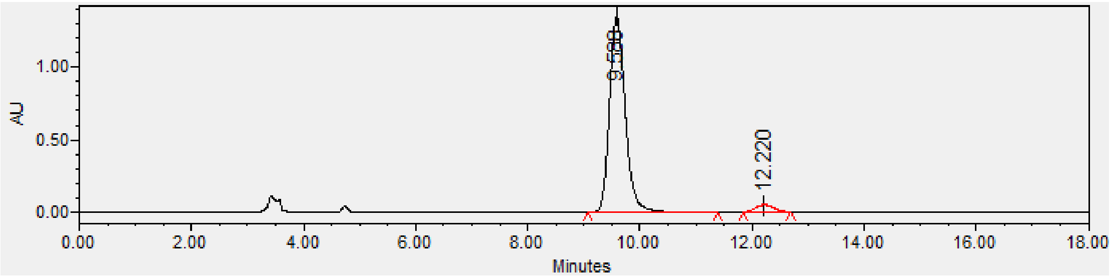

	Name	Retention Time (min)	Area (µV*sec)	% Area	Height (µV)	Int Type	Amount	Units	Peak Type	Peak Codes
1		9.588	26608243	95.67	1349525	Bb			Unknown	
2		12.220	1204112	4.33	51389	bb			Unknown	

(*S*)-1-phenylbutane-1,4-diyl bis(diphenylphosphinate) (9)

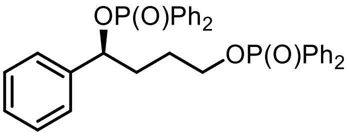

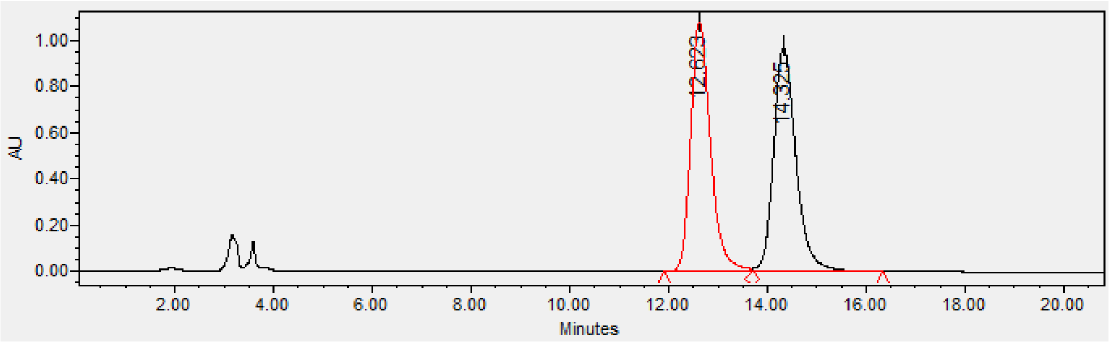

	Name	Retention Time (min)	Area (µV*sec)	% Area	Height (µV)	Int Type	Amount	Units	Peak Type	Peak Codes
1		12.623	30215961	49.71	1079723	BV			Unknown	
2		14.325	30571258	50.29	971721	VB			Unknown	

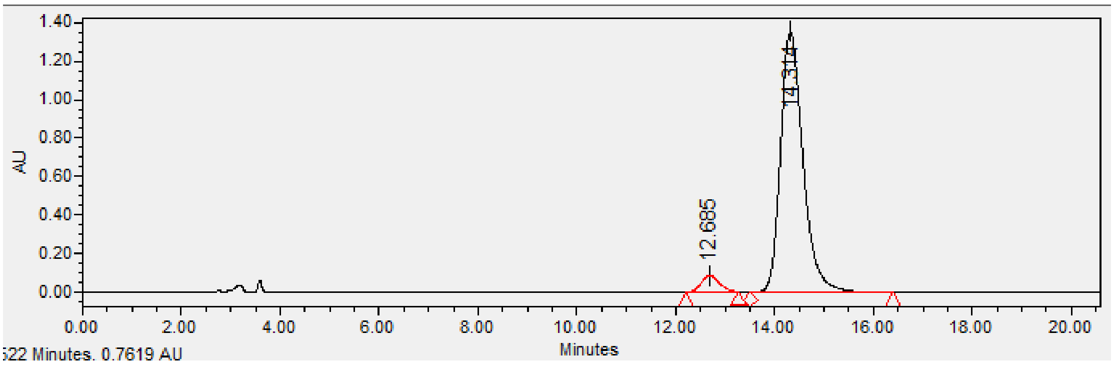

	Name	Retention Time (min)	Area (µV*sec)	% Area	Height (µV)	Int Type	Amount	Units	Peak Type	Peak Codes
1		12.685	2240157	4.97	86291	bb			Unknown	
2		14.314	42801905	95.03	1353427	VB			Unknown	

(*S*)-4-(4-methoxyphenyl)-1-phenylbutan-1-ol (10)

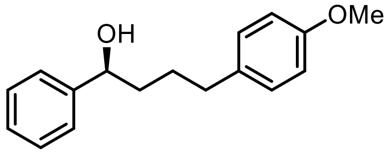

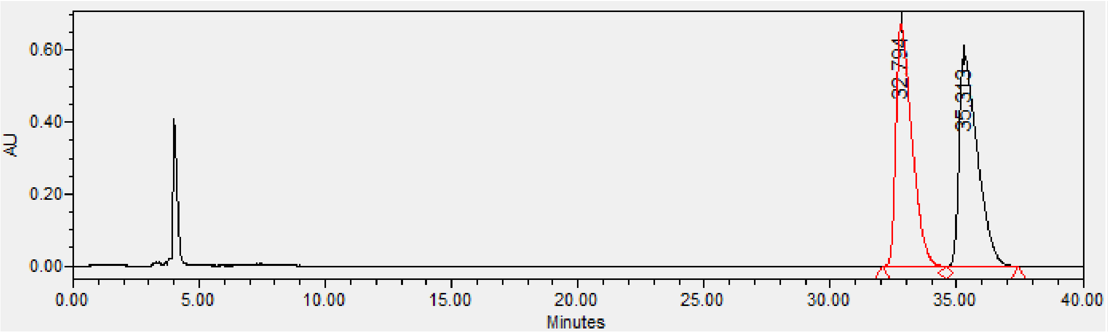

	Name	Retention Time (min)	Area (µV*sec)	% Area	Height (µV)	Int Type	Amount	Units	Peak Type	Peak Codes
1		32.794	29993119	49.98	677379	BV			Unknown	
2		35.313	30012245	50.02	587164	VB			Unknown	

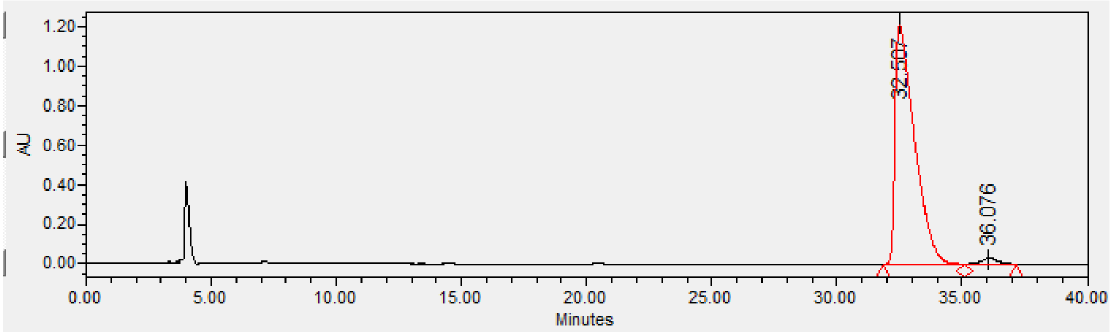

	Name	Retention Time (min)	Area (µV*sec)	% Area	Height (µV)	Int Type	Amount	Units	Peak Type	Peak Codes
1		32.507	65036307	98.23	1213070	BV			Unknown	
2		36.076	1171121	1.77	26426	bb			Unknown	

(*S*)-4-(3,5-dimethylphenyl)-1-phenylbutan-1-ol (11)

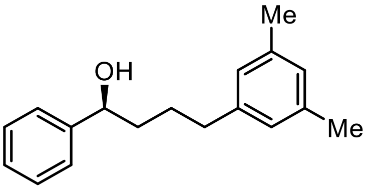

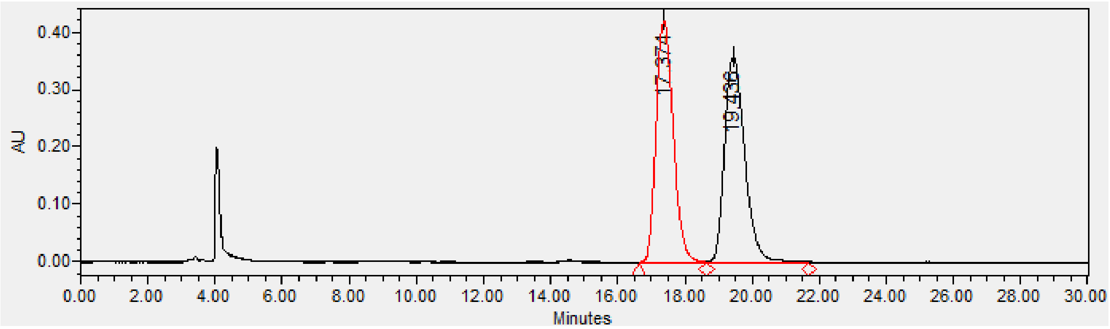

	Name	Retention Time (min)	Area (µV*sec)	% Area	Height (µV)	Int Type	Amount	Units	Peak Type	Peak Codes
1		17.374	14996948	50.17	420645	BV			Unknown	
2		19.436	14894838	49.83	357846	VV			Unknown	

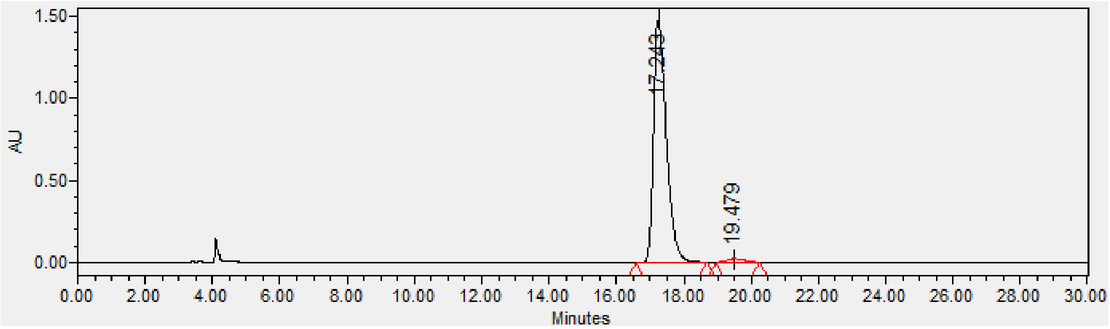

	Name	Retention Time (min)	Area (µV*sec)	% Area	Height (µV)	Int Type	Amount	Units	Peak Type	Peak Codes
1		17.243	39056547	98.05	1477310	Bb			Unknown	
2		19.479	775860	1.95	23071	bb			Unknown	

(*S*)-4-phenylbutane-1,3-diyl bis(diphenylphosphinate) (12)

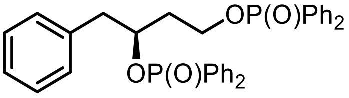

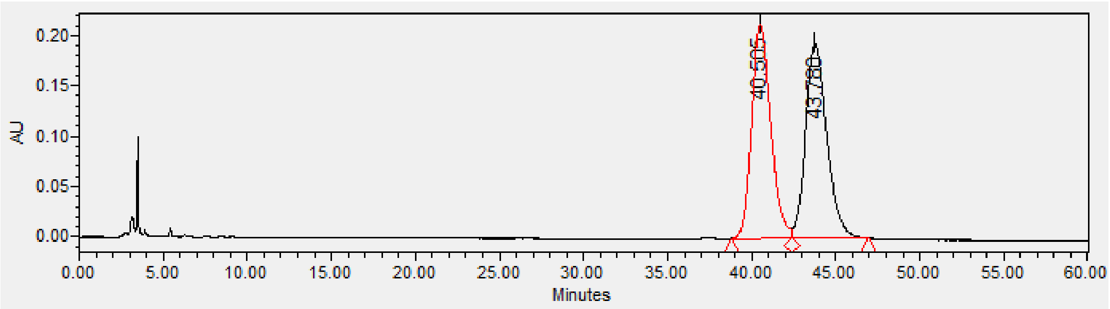

	Name	Retention Time (min)	Area (µV*sec)	% Area	Height (µV)	Int Type	Amount	Units	Peak Type	Peak Codes
1		40.505	16833357	49.69	212938	Bv			Unknown	
2		43.780	17042378	50.31	193908	vB			Unknown	

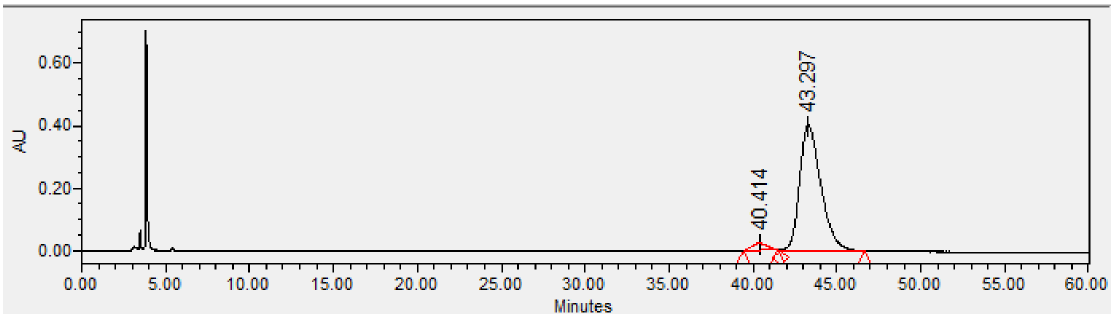

	Name	Retention Time (min)	Area (µV*sec)	% Area	Height (µV)	Int Type	Amount	Units	Peak Type	Peak Codes
1		40.414	1373001	3.70	21437	bb			Unknown	
2		43.297	35749457	96.30	403059	VB			Unknown	

(*R*)-4-(3,5-dimethylphenyl)-1-phenylbutan-2-ol (13)

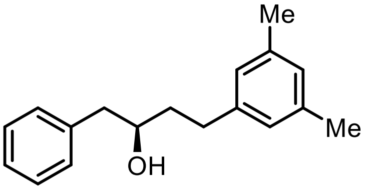

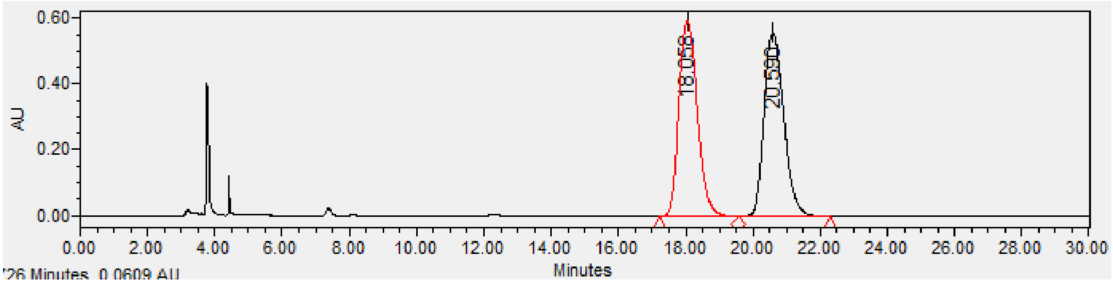

	Name	Retention Time (min)	Area (μV*sec)	% Area	Height (μV)	Int Type	Amount	Units	Peak Type	Peak Codes
1		18.058	22775269	49.91	594107	BV			Unknown	
2		20.590	22853760	50.09	558730	VB			Unknown	

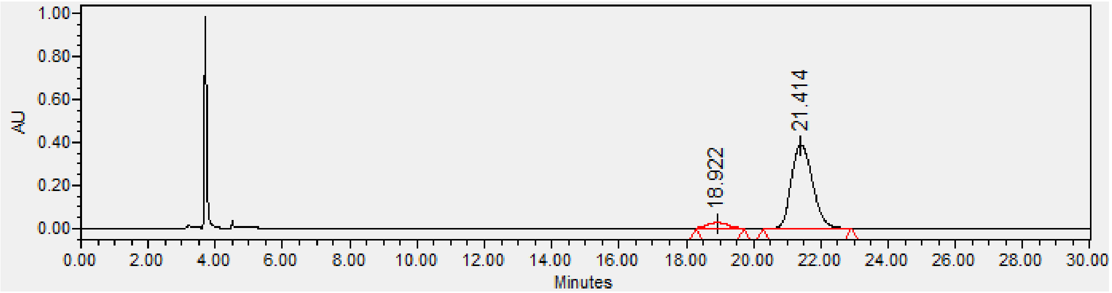

	Name	Retention Time (min)	Area (μV*sec)	% Area	Height (μV)	Int Type	Amount	Units	Peak Type	Peak Codes
1		18.922	994300	5.54	25022	bb			Unknown	
2		21.414	16953265	94.46	388183	BB			Unknown	

12. Reference

[1] (a) Y. Wang, M. E. Muratore, Z. Rong, A. M. Echavarren, *Angew. Chem. Int. Ed.* **2014**, *53*, 14022-14026; (b) W. L. Xing, R. Shang, G. Z. Wang, Y. Fu, *Chem. Commun.* **2019**, *55*, 14291-14294; (c) G. Q. Cui, J. C. Dai, Y. Li, Y. B. Li, D. D. Hu, K. J. Bian, J. Sheng, X. S. Wang, *Org. Lett.* **2021**, *23*, 7503-7507; (d) H.-D. Zuo, S.-S. Zhu, W. -J. Hao, S. -C. Wang, S.-J. Tu, B. Jiang, *ACS Catal.* **2021**, *11*, 6010-6019; (e) J. Zhou, Q. Yang, C. S. Lee, J. Wang, *Angew. Chem. Int. Ed.* **2022**, *61*, e202202160.

[2] S. Z. Nie, R. T. Davison, V. M. Dong, *J. Am. Chem. Soc.* **2018,** *140*, 16450-16454.

[3] (a) L. Liu, X. Bao, H. Xiao, J. Li, F. Ye, C. Wang, Q. Cai, S. Fan, *J. Org. Chem.* **2019**, *84*, 423-434; (b) Z. Q. Zhang, C. T. Yang, L. J. Liang, B. Xiao, X. Lu, J. H. Liu, Y. Y. Sun, T. B. Marder, Y. Fu, *Org. Lett.* **2014**, *16*, 6342-6345.

[4] M. J. Frisch, et al. Gaussian 09; Revision D.01; Gaussian, Inc.: Wallingford, CT, **2013**.

[5] J.-D. Chai, M. Head-Gordon, *Phys. Chem. Chem. Phys.* **2008**, *10*, 6615-6620.

[6] W. R. Wadt, P. J. Hay, *J. Chem. Phys.* **1985**, *82*, 284-298.

[7] A. W. Ehlers, M. Bohme, S. Dapprich, A. Gobbi, A. Hollwarth, V. Jonas, K. F. Kohler, R. Stegmann, A. Veldkamp, G. Frenking, *Chem. Phys. Lett*. **1993**, *208*, 111-114.

[8] (a) K. Fukui, *Acc. Chem. Res.* **1981**, *14*, 363-368; (b) C. Gonzalez, H. B. Schlegel, *J. Chem. Phys.* **1989**, *90*, 2154-2161.

[9] L. Falivene, R. Credendino, A. Poater, A. Petta, L. Serra, R. Oliva, V. Scarano, L. Cavallo. *Organometallics* **2016**, *35*, 2286-2293.

13. Cartesian coordinates

ts_Cu_L17_E

Cu	0.44602800	-0.68261000	-1.15723000
P	-1.54421700	-1.35384900	-0.12768100
P	1.26216900	1.26200600	-0.20201100
C	1.59996000	-1.84024500	-2.70246600
C	0.72340100	-2.22437600	-3.82471600
C	0.21453400	-0.85491900	-3.42439500
C	1.65523800	-2.44554000	-1.38993700
H	1.02787700	-3.32453700	-1.24378400
C	2.82624000	-2.35948700	-0.53325500
C	2.89636000	-3.15871700	0.63365800
C	3.94182400	-1.52171200	-0.77355900
H	0.55894100	-0.02327200	-4.03475900
H	-0.84478300	-0.79316200	-3.17826400
H	0.04062900	-3.04463300	-3.59744400
H	1.17576600	-2.33599300	-4.81059200
C	3.97423800	-3.09846100	1.50586400
H	2.07496100	-3.84047500	0.84659600
C	5.02276000	-1.47223100	0.09799300
H	3.96182100	-0.87921400	-1.64820600
C	5.05362300	-2.24922200	1.25604200
H	3.97503700	-3.73125800	2.39094400
H	5.84713700	-0.80067700	-0.13022400
H	5.89844400	-2.20514400	1.93671900
O	-1.59813600	1.45834000	0.32879800
C	-3.02998600	-0.29465600	-0.30628400
C	-4.29575700	-0.72005200	-0.71196700
H	-4.47922400	-1.77626900	-0.88188200
C	-5.31302500	0.20743400	-0.91870500
H	-6.29205500	-0.13073300	-1.24377700
C	-5.08596800	1.56547100	-0.71204400
H	-5.89494900	2.26778100	-0.88337300
C	-3.84141100	2.02597700	-0.28237200
C	-2.84517500	1.07213700	-0.09279400
C	0.22395500	2.76732400	-0.38807500
C	0.70555900	3.99828200	-0.83903900
H	1.76504700	4.12004500	-1.03908600
C	-0.16704300	5.05995200	-1.05345200
H	0.21785500	6.00800200	-1.41585700
C	-1.52993200	4.91566800	-0.80803000
H	-2.19143400	5.75740800	-0.98408500
C	-2.04677000	3.71186700	-0.33271500
C	-1.14593000	2.66483200	-0.14125200
C	-3.50461200	3.47561000	0.06267900
C	-4.45348000	4.46492600	-0.61954400
H	-4.40423800	4.38276900	-1.71018700
H	-4.21149400	5.49338500	-0.33536900
H	-5.48605900	4.29070100	-0.30274000
C	-3.61993500	3.64400500	1.59757400
H	-2.95676600	2.94931100	2.12243700
H	-4.64772600	3.44804100	1.92250000
H	-3.34835600	4.66546800	1.88617600
C	-1.33588400	-1.47360300	1.68363400
C	-0.06920000	-1.81503800	2.16417100
H	0.75473800	-1.95529600	1.46932300
C	0.14452800	-1.96218300	3.53055600
H	1.13514400	-2.22308000	3.88833400
C	-0.89773600	-1.74738500	4.42700100
H	-0.72715800	-1.85093600	5.49473400
C	-2.16013800	-1.39346000	3.95456100
H	-2.97435100	-1.22147300	4.65279900
C	-2.38162900	-1.26237100	2.58733900
H	-3.36907100	-0.99406700	2.22136300
C	-2.16735300	-3.00916000	-0.60941300
C	-2.35734500	-3.26441700	-1.97356800
H	-2.16756300	-2.48117900	-2.70303400
C	-2.79199400	-4.51126900	-2.40831100
H	-2.93889000	-4.69175700	-3.46903500
C	-3.02658400	-5.52740300	-1.48379400
H	-3.35681500	-6.50521800	-1.82191500
C	-2.83145800	-5.28581600	-0.12783900
H	-3.00958100	-6.07435900	0.59741700
C	-2.40777700	-4.03242200	0.30978700
H	-2.25968900	-3.85470100	1.37028000
C	1.58767700	1.16337200	1.59849600
C	0.85336500	1.87282600	2.54997700
H	0.08065400	2.56967900	2.24118200
C	1.10949000	1.69579800	3.90764600
H	0.52524100	2.24724900	4.63851400
C	2.11164200	0.82599400	4.32576400
H	2.31093800	0.69436500	5.38547900
C	2.85032600	0.11901600	3.37955600

H	3.63051200	-0.57271900	3.68353800
C	2.57965700	0.27283600	2.02598000
H	3.14812100	-0.30150000	1.30188500
C	2.85695600	1.80558900	-0.92009900
C	3.01456100	1.63889600	-2.30058600
H	2.20286300	1.20301700	-2.87756300
C	4.20323100	2.00038600	-2.92476600
H	4.31705400	1.85993100	-3.99556400
C	5.25299700	2.52239500	-2.17160000
H	6.18828000	2.79036100	-2.65415500
C	5.10161700	2.69538700	-0.79852800
H	5.91710900	3.10254600	-0.20806600
C	3.90692600	2.34495700	-0.17436300
H	3.79890700	2.47707100	0.89751100
H	2.46085900	-1.24962100	-3.00898300

ts_Cu_L17_Z

Cu	-0.44204100	-0.85233400	-1.04791400
P	-1.30264800	1.17160600	-0.20834800
P	1.67565100	-1.30201300	-0.15506400
C	-1.09063400	-2.75231700	-2.04558800
C	-1.76915100	-2.56843100	-3.33943400
C	-0.92262700	-1.30877700	-3.21114700
C	-1.55571600	-2.71635500	-0.67797000
H	-0.95167400	-3.33643700	-0.01824200
C	-2.90837800	-2.46380000	-0.22107000
C	-3.34975400	-3.02015000	1.00216800
C	-3.82170700	-1.61198200	-0.88267700
H	-0.03010500	-1.27238000	-3.83171700
H	-1.51089800	-0.39426800	-3.23028300
H	-2.84542400	-2.41500400	-3.30925500
H	-1.49201400	-3.26879700	-4.12742800
C	-4.60077200	-2.73361000	1.53080300
H	-2.67904100	-3.68451900	1.54351000
C	-5.07558400	-1.33122000	-0.35288700
H	-3.52172000	-1.10716300	-1.79502700
C	-5.48148000	-1.88138300	0.86166100
H	-4.89477600	-3.18441000	2.47618000
H	-5.73018900	-0.64925700	-0.88989500
H	-6.45884000	-1.65537800	1.27699400
O	1.58261500	1.51172400	0.21059000
C	-0.33018600	2.72045100	-0.44184600
C	-0.88942900	3.93010800	-0.86256400
H	-1.96037400	4.00403400	-1.01602800
C	-0.08249100	5.03538900	-1.10791100
H	-0.53063700	5.96417300	-1.44639600
C	1.29479200	4.95893500	-0.92266400
H	1.90409200	5.83392700	-1.12270400
C	1.88997800	3.78201300	-0.47391200
C	1.05194400	2.68941000	-0.24743500
C	3.08546600	-0.16676200	-0.44795900
C	4.35558900	-0.53095200	-0.89662100
H	4.58897100	-1.57840600	-1.05915100
C	5.31349700	0.44552500	-1.15462600
H	6.29616500	0.15515400	-1.51318000
C	5.02258700	1.79230800	-0.95498300
H	5.78632700	2.53405600	-1.16342500
C	3.77150500	2.19222600	-0.48549500
C	2.83474600	1.19020700	-0.24872000
C	3.37400800	3.62599000	-0.14086400
C	4.23871100	4.65354100	-0.87699600
H	4.13674200	4.55991800	-1.96298700
H	5.29331300	4.52933400	-0.61411200
H	3.96490500	5.67215200	-0.58644900
C	3.54844200	3.81946900	1.38530100
H	2.94515500	3.09877100	1.94626000
H	3.23819600	4.82934300	1.67575500
H	4.59807000	3.67865900	1.66589500
C	-1.54690400	1.07861300	1.60647200
C	-2.50112800	0.17188900	2.08461600
H	-3.09921000	-0.40953800	1.39136300
C	-2.69935900	0.01416200	3.45071000
H	-3.45080500	-0.68979200	3.79597400
C	-1.93207800	0.74261500	4.35747500
H	-2.07928000	0.61281100	5.42586800
C	-0.97262600	1.63416500	3.88844100
H	-0.36735800	2.20302600	4.58803700
C	-0.78417400	1.80834700	2.51944100
H	-0.04148700	2.51888500	2.17151500
C	-2.94253500	1.70046700	-0.84710400
C	-3.11381000	1.70750900	-2.23624100
H	-2.28436700	1.43447600	-2.88246500
C	-4.33075600	2.06999200	-2.80156500

H	-4.44805100	2.06298500	-3.88108800
C	-5.39760500	2.43309400	-1.98182300
H	-6.35286900	2.70636900	-2.42010600
C	-5.23108100	2.44723000	-0.60063800
H	-6.05514700	2.73553700	0.04525400
C	-4.01013400	2.08757900	-0.03507900
H	-3.89793600	2.09673300	1.04369600
C	1.64363600	-1.41016300	1.67453200
C	2.78616000	-1.21777900	2.45889200
H	3.73684700	-0.98910100	1.98493700
C	2.70998200	-1.31421900	3.84465600
H	3.60145400	-1.15908500	4.44562900
C	1.49361300	-1.60763500	4.45766800
H	1.43406900	-1.68085500	5.53989900
C	0.35446000	-1.80239100	3.68277100
H	-0.59990800	-2.01491300	4.15303200
C	0.42595200	-1.69852500	2.29682300
H	-0.46896900	-1.82698800	1.69390200
C	2.36494000	-2.91242600	-0.69078800
C	2.47360000	-3.13489600	-2.07039000
H	2.16305600	-2.35637000	-2.76395400
C	2.96769300	-4.34007400	-2.55610600
H	3.05261400	-4.49737800	-3.62724100
C	3.34260300	-5.34700900	-1.66798300
H	3.72152500	-6.29198900	-2.04598000
C	3.22431200	-5.13952400	-0.29747200
H	3.51056000	-5.92234300	0.39872200
C	2.74167200	-3.92684000	0.19139700
H	2.65645800	-3.77360200	1.26271700
H	-0.13293800	-3.25646700	-2.15367600

ts_Cu_L16_E

C	3.75854800	3.48766200	-0.46547500
C	2.85005300	4.21325900	-1.17902300
C	1.54282400	3.70994800	-1.40995600
C	1.13031200	2.45493700	-0.85360300
C	2.16647500	1.64429500	-0.25149300
C	3.41945100	2.18441900	-0.04854100
H	1.01994800	5.40228600	-2.64268100
H	4.75430100	3.87372800	-0.27198800
H	3.10932000	5.18586600	-1.58859300
C	0.65912200	4.46876300	-2.21882400
C	-0.26351000	2.10666100	-0.99702600
H	4.19351400	1.57907200	0.40972300
C	-1.06643300	2.86426900	-1.82820900
C	-0.60764900	4.02688200	-2.47555100
H	-2.10983400	2.58965300	-1.94682800
H	-1.27708800	4.58866400	-3.11904100
P	1.91246700	-0.13995700	0.12722600
P	-1.17362700	0.91443000	0.09372900
C	3.57691300	-0.77139300	0.55085500
C	4.58407700	-0.97961000	-0.39815500
C	3.82242900	-1.08878400	1.88931800
C	5.82023900	-1.48266800	-0.00842700
H	4.39520900	-0.75416600	-1.44403500
C	5.06327600	-1.58628300	2.28054300
H	3.03163500	-0.95866400	2.62377600
C	6.06208400	-1.78228800	1.33171500
H	6.59533100	-1.64593900	-0.75139500
H	5.24444300	-1.82822200	3.32342800
H	7.02734000	-2.17845300	1.63321600
C	1.60876600	-0.85910400	-1.53382000
C	1.88407000	-0.20792400	-2.73977800
C	1.03005700	-2.13251600	-1.56142300
C	1.57792700	-0.82211000	-3.95180300
H	2.32381600	0.78521700	-2.73788000
C	0.73220300	-2.74812900	-2.77217200
H	0.76746400	-2.62203200	-0.62901600
C	1.00167700	-2.09058000	-3.97031900
H	1.78354800	-0.30427900	-4.88408700
H	0.25529100	-3.72333800	-2.76689000
H	0.75655300	-2.56123400	-4.91807800
C	-2.50441200	0.32129500	-1.02144300
C	-3.85813800	0.61067300	-0.84252200
C	-2.11736900	-0.51404100	-2.07460700
C	-4.80908600	0.07043700	-1.70730200
H	-4.17730500	1.25917900	-0.03241000
C	-3.06565800	-1.04626300	-2.93661700
H	-1.07013100	-0.75742600	-2.21780400
C	-4.41628400	-0.75714300	-2.75408800
H	-5.86033500	0.29970800	-1.55814500
H	-2.74716100	-1.70476800	-3.73870000
H	-5.16029900	-1.18057800	-3.42247100

C	-2.02688800	2.04657300	1.26393100
C	-1.89432400	3.43666000	1.26834400
C	-2.80893600	1.44243000	2.25834600
C	-2.54167200	4.20397400	2.23564500
H	-1.28217700	3.93219100	0.52260700
C	-3.47114700	2.20910700	3.20895000
H	-2.89620900	0.35975300	2.28740800
C	-3.33589500	3.59641400	3.20151500
H	-2.42182700	5.28344800	2.22892300
H	-4.08252800	1.72247600	3.96309700
H	-3.84241100	4.19795400	3.95042300
Cu	0.06325300	-0.58811200	1.36848300
C	-0.75477400	-1.77582100	3.03782500
C	0.02130300	-1.56543500	4.27486300
C	0.07062800	-0.21646600	3.58916300
C	-0.38727600	-2.60423600	1.90688400
H	0.50612500	-3.21372900	2.04495800
C	-1.37130300	-3.13734800	0.97433800
C	-1.04946100	-4.26444000	0.18467300
C	-2.65909700	-2.59268400	0.77548200
H	-0.63417900	0.53417700	3.93781000
H	1.07114100	0.17385200	3.40177200
H	0.97668600	-2.09257400	4.30262000
H	-0.50595400	-1.64054100	5.22603200
C	-1.93329800	-4.78327500	-0.75373200
H	-0.07971300	-4.73963000	0.32476100
C	-3.54321500	-3.11909700	-0.15432600
H	-2.96589500	-1.71367500	1.33715100
C	-3.19205900	-4.21555200	-0.94042200
H	-1.63699500	-5.65320900	-1.33666500
H	-4.51302100	-2.64568500	-0.28456500
H	-3.88369400	-4.61709200	-1.67490100
H	-1.80912500	-1.52665400	3.14572200

ts_Cu_L15_E

Cu	-0.10713500	0.36668100	-1.55509900
P	-1.92600400	0.04664000	-0.13724800
P	1.01496300	0.87756800	0.44784000
C	-1.10336700	3.34295900	3.02108000
C	-2.40584200	2.88852800	2.84053700
C	-2.66957100	1.87390900	1.92435100
C	-1.63438500	1.29780500	1.18416500
C	-0.31038100	1.73945700	1.38923300
C	-0.06088600	2.76750600	2.29967700
H	-0.89650500	4.14466400	3.72381700
H	-3.22230500	3.33180300	3.40282300
H	-3.69195100	1.54077000	1.77208400
H	0.95573600	3.12606800	2.43834900
C	2.39971000	2.06892500	0.43201100
C	2.35302100	3.05722000	-0.55908200
C	3.48682300	2.03114400	1.30849100
C	3.36057100	4.01019700	-0.65253500
H	1.52464300	3.06528400	-1.26392600
C	4.50072400	2.98134300	1.20591900
H	3.54742000	1.25523200	2.06540900
C	4.43691100	3.97354700	0.23182200
H	3.31249000	4.77469000	-1.42220900
H	5.34317300	2.94344900	1.89031300
H	5.22888000	4.71273000	0.15573500
C	1.48980000	-0.44197200	1.62497000
C	1.74518200	-1.71487200	1.11321300
C	1.56051400	-0.23260400	3.00825800
C	2.08837600	-2.75898300	1.96717400
H	1.65877700	-1.89951700	0.04936400
C	1.90224800	-1.27729200	3.85935000
H	1.33683300	0.74535300	3.42546400
C	2.16990400	-2.54259100	3.33784500
H	2.28271600	-3.73986600	1.54534000
H	1.95589600	-1.10560300	4.93061600
H	2.43470800	-3.35822400	4.00493600
C	-3.72634500	0.17149700	-0.43303500
C	-4.17899100	1.26857300	-1.17684700
C	-4.65081200	-0.76821700	0.02884800
C	-5.53424500	1.43230800	-1.43795600
H	-3.46488200	2.00239700	-1.54344200
C	-6.00788000	-0.60768200	-0.24305000
H	-4.31134600	-1.62626300	0.60131600
C	-6.45138400	0.49185400	-0.97175800
H	-5.87473000	2.28978200	-2.01062300
H	-6.71952600	-1.34415300	0.11832900
H	-7.51001400	0.61613100	-1.17945400
C	-1.68596800	-1.56852300	0.68335500
C	-1.27164800	-2.63559600	-0.12068700

C	-1.83104700	-1.76029200	2.05987700
C	-1.02821600	-3.88376600	0.44189400
H	-1.09767400	-2.46950500	-1.18089600
C	-1.57125600	-3.00593600	2.62257300
H	-2.12391600	-0.92954000	2.69606200
C	-1.17594300	-4.06889500	1.81379900
H	-0.69327800	-4.70118900	-0.18937500
H	-1.66785100	-3.14407700	3.69512100
H	-0.96639600	-5.03764800	2.25784600
C	0.78325100	0.43274600	-3.57201100
C	-0.10233100	1.09483300	-4.54620300
C	-0.41119500	1.81880500	-3.25133600
C	0.66147500	-0.91958600	-3.07124300
H	-0.09556700	-1.53834200	-3.55237900
C	1.78572100	-1.63285700	-2.48702100
C	1.70840000	-3.03309200	-2.30435500
C	2.98488700	-1.01651000	-2.05487600
H	0.03100900	2.80563600	-3.13527800
H	-1.45671500	1.75644400	-2.95042800
H	-0.91541200	0.47184100	-4.92111200
H	0.37003900	1.66480200	-5.34591300
C	2.73861000	-3.75749600	-1.71864500
H	0.81000100	-3.55153000	-2.63469700
C	4.01370500	-1.74702200	-1.47539200
H	3.10425300	0.06028300	-2.14597600
C	3.90644700	-3.12529400	-1.29234700
H	2.62738800	-4.83276600	-1.59657900
H	4.91043300	-1.22558600	-1.14954200
H	4.71142800	-3.68989800	-0.83234400
H	1.74724900	0.92501100	-3.46011400

ts_Cu_L11_E

C	1.06244600	-0.45821800	2.05832200
C	-0.01548000	-1.53955400	1.88756400
H	1.77088200	-0.71172700	2.85435200
H	0.59167800	0.48903500	2.34832100
H	0.42978300	-2.48572600	1.55600900
H	-0.52332300	-1.71681500	2.84222000
P	1.92050000	-0.11150000	0.44852600
P	-1.22660100	-0.99907500	0.57781600
C	2.74027900	1.48759000	0.77464100
C	2.04346500	2.64223100	0.40178100
C	3.98614300	1.60457500	1.39757500
C	2.58146400	3.89881800	0.66291300
H	1.08474500	2.54946500	-0.10406900
C	4.52666700	2.86271900	1.64583100
H	4.53754600	0.71132700	1.67942200
C	3.82403400	4.00999600	1.28191700
H	2.03256400	4.78972900	0.37203800
H	5.49743500	2.94878200	2.12520900
H	4.24982100	4.99017000	1.47576500
C	3.28778900	-1.32133300	0.35017800
C	4.09791900	-1.27426200	-0.79315400
C	3.51612500	-2.32591300	1.29394800
C	5.12138500	-2.19675100	-0.97609100
H	3.92440300	-0.50525500	-1.54288600
C	4.53480200	-3.25755200	1.10473900
H	2.90057800	-2.39556600	2.18561800
C	5.34070300	-3.19378600	-0.02715800
H	5.74268300	-2.14343600	-1.86507900
H	4.69797000	-4.03321600	1.84725000
H	6.13320400	-3.92146800	-0.17369700
C	-2.27897100	0.15751800	1.53097300
C	-2.03221100	1.52412300	1.37400900
C	-3.26351400	-0.26265700	2.43205500
C	-2.74892600	2.45822400	2.11775000
H	-1.30225100	1.86253200	0.64380000
C	-3.98072800	0.67148800	3.17079000
H	-3.48080000	-1.32093900	2.54844800
C	-3.72131900	2.03297900	3.01634500
H	-2.56056800	3.51578300	1.96482500
H	-4.74641600	0.33817700	3.86542100
H	-4.28834100	2.76027900	3.59032800
C	-2.25406200	-2.47989700	0.28610300
C	-2.49183000	-3.47068400	1.24485700
C	-2.81287000	-2.62297900	-0.98855500
C	-3.28376200	-4.57372900	0.93854600
H	-2.05550400	-3.38755000	2.23635300
C	-3.61313300	-3.72000400	-1.29211800
H	-2.60277600	-1.87335100	-1.74738500
C	-3.84926600	-4.69683900	-0.32803800
H	-3.45877500	-5.33813600	1.68993000
H	-4.04457600	-3.81766600	-2.28379600

H	-4.46709100	-5.55768300	-0.56611900
Cu	0.17254300	-0.19390300	-1.08146600
C	-0.26571600	0.16683100	-3.20448200
C	0.73265700	-0.47943000	-4.07748200
C	0.64521700	-1.42240700	-2.89629000
C	-0.10666800	1.39807600	-2.46025200
H	0.81730500	1.94612900	-2.64434200
C	-1.23685200	2.20020700	-2.01733100
C	-1.02994400	3.54329900	-1.62668600
C	-2.56581200	1.72563200	-1.92378600
H	0.03250100	-2.31057800	-3.02888000
H	1.59182000	-1.60676000	-2.38739500
H	1.68016600	0.05602000	-4.15634800
H	0.39387800	-0.84190000	-5.04820600
C	-2.06653800	4.34236000	-1.16033800
H	-0.02838800	3.96232300	-1.71078500
C	-3.59905700	2.53076400	-1.46673600
H	-2.78880000	0.69383900	-2.18432200
C	-3.36709400	3.84812400	-1.07325200
H	-1.85622100	5.37066400	-0.87425700
H	-4.60129100	2.11461700	-1.39986500
H	-4.17813200	4.47199900	-0.71055300
H	-1.28199100	-0.17862500	-3.38447200
